# Identification of a five-immune gene model as an independent prognostic factor in hepatocellular carcinoma

**DOI:** 10.1186/s12885-021-08012-2

**Published:** 2021-03-16

**Authors:** Haitao Chen, Yueying Li, Shu-Yuan Xiao, Jianchun Guo

**Affiliations:** 1 Department of Orthopedic Surgery, Zhongnan Hospital of Wuhan University, Wuhan, 430071 China; 2 Department of Pathology, Zhongnan Hospital of Wuhan University, Wuhan, 430071 China; 3 Wuhan University Center for Pathology and Molecular Diagnostics, Wuhan, 430071 China; 4 Department of Pathology, University of Chicago Medicine, Chicago, IL USA

**Keywords:** Immune gene, Prognosis, Risk model, Hepatocellular carcinoma, Pathology

## Abstract

**Background:**

Hepatocellular carcinoma (HCC) is a common malignant tumor with a poor prognosis. We aimed to identify a new prognostic model of HCC based on differentially expressed (DE) immune genes.

**Methods:**

The DE immune genes were identified based on an analysis of 374 cases of HCC and 50 adjacent non-tumor specimens from the Cancer Genome Atlas (TCGA) database. Univariate Cox analysis, Lasso regression, and multivariate Cox analysis were used to construct the model based on the training group. Survival analysis and the receiver operating characteristic (ROC) curves were used to evaluate model performance. The testing group and the entire group were subsequently used for validation of the model.

**Results:**

A five-immune gene model consisted of HSPA4, ISG20L2, NDRG1, EGF, and IL17D was identified. Based on the model, the overall survival was significantly different between the high-risk and low-risk groups (*P* = 7.953e-06). The AUCs for the model at 1- and 3-year were 0.849 and 0.74, respectively. The reliability of the model was confirmed using the validation groups. The risk score was identified as an independent prognostic parameter and closely related to the content of immune cells from human HCC specimens.

**Conclusion:**

We identified a five-immune gene model that can be used as an independent prognostic marker for HCC.

**Supplementary Information:**

The online version contains supplementary material available at 10.1186/s12885-021-08012-2.

## Background

Hepatocellular carcinoma (HCC) is common malignancy worldwide, being the third leading cause of cancer-associated death globally. Its morbidity and mortality continue to rise, causing more than 600,000 deaths annually [1, 2]. The symptoms and signs of early HCC are hard to notice, making its diagnosis often delayed, which is partly related to the poor prognosis [3–5]. In addition, due to host variabilities, individuals with the same pathological stage may still have significant differences in the overall survival (OS) [6]. Therefore, it is important to identify relevant genes and develop a molecular model that can better predict the prognosis of HCC.

In recent years, immunotherapy has become an important approach for HCC treatment [3]. A variety of strategies including cancer vaccines, adoptive cellular therapy, and immune checkpoint blockade (ICB) [7, 8], have been explored. Several immune checkpoint inhibitors, such as anti-PD-L1, anti-CTLA-4, and anti-PD-1 monoclonal antibodies, have displayed therapeutic effects for HCC [9, 10], both in the induction and maintenance of treatments [11, 12].

Previous studies have suggested that immune-related genes (or immune genes) may be related to the prognosis and response of HCC patients to immunotherapy [13]. Dong et al. [14] have shown that high expressions of STAT5A, STAT5B, and STAT6 are associated with improved prognosis in HCC patients. In the current study, we aim to further identify a prognostic model for HCC based on immune genes and to examine its clinical significance.

## Methods

### Basic information

Clinical information and expression data were obtained from The Cancer Genome Atlas (TCGA) database. Cancer-related transcription factors (TFs) and immune genes were available in the Cistrome database [15] and the ImmPort database [16], respectively. After screening of the cancer samples, we randomly divided the 343 tumor samples into the training and testing groups using the “caret” package of R software (version 4.0.3).

### Detection of the differentially expressed (DE) immune genes

The Wilcoxon signed-rank test was applied to identify DE genes and DE immune genes with R software. False discovery rate (FDR) < 0.05 and Log_2_(fold change [FC]) > 1 were set as the cut-offs. The DE genes and immune genes were presented in the volcano plot and heatmap using the “gplots” package and “Pheatmap” package.

### Function enrichment analyses of the DE immune genes

Gene Ontology (GO) analysis and the Kyoto Encyclopedia of Genes and Genomes (KEGG) analysis were analyzed with the Database for Annotation, Visualization, and Integrated Discovery (*DAVID*) (v6.8) and the “ClusterProfiler” R package, respectively. GO terms, including cellular component, biological process, and molecular function were considered significantly enriched when Bonferroni correction < 0.001 and FDR < 0.05, and p.adjust < 0.001 was set as the cut-offs for KEGG terms. The “Pathview” package was used to detect the dysregulated genes enriched in the pathways.

### Consensus clustering analysis

For the analysis of the DE immune genes in HCC, tumor samples from TCGA were divided into clusters using the “ConsensusClusterPlus” package. Principal component analysis (PCA) was applied to validate the reliability of clustering with the “ggplot2” package. Survival analysis was performed to stratify the clusters using the “survival” package.

### Construction of a DE immune genes-TFs network

We constructed the DE TFs based on the DE genes and cancer-related TFs. A co-expression network was established with the WGCNA package. Scale free topology model and mean connectivity was used to screen the optimal soft threshold power. We set the abline at 0.95. Subsequently, the optimal soft threshold power was used to create an adjacency matrix. The interaction pairs were identified when the weight score was more than 0.3. Meanwhile, Spearman correlation analysis was performed to estimate the correlation between DE immune genes and DE TFs. Correlation coefficient > 0.6 and *p* < 0.001 were set as thresholds to find more robust interaction pairs. In addition, the protein-protein interaction (PPI) network was established using the STRING database and the hub genes were identified with the Cytohubba plug-in. The qualified interactions were imported to Cytoscape (v3.7.2) to construct the DE immune genes-TFs network.

### Establishment of a prognostic risk model

A risk model was developed with the training group, and this model was established with published methodologies [17, 18]. Univariate analysis was performed with the “survival” package, and the genes with *p* < 0.05 were identified as survival-related. Lasso regression was applied by the “glmnet” R package with the number of lambda = 1000 to eliminate collinear or correlated genes. Lambda.min was set as the cutoff point to bring minimum mean cross-validated error, and qualified genes were selected based on the lambda.min for further analysis. Subsequently, multivariate analysis was performed to screen for the ultimate immune genes. Gene and protein expression levels were verified in the Oncomine database (https://www.oncomine.org) [19] and Human Protein Atlas (HPA) (https://www.proteinatlas.org/) [20]. In addition, the survival analysis of these genes was conducted. Based on the resulting immune genes, a risk score was calculated according to the following formula:

$$ \mathrm{Risk}\kern0.5em \mathrm{score}={\sum}_{\mathrm{i}-1}^{\mathrm{N}}\left(\mathrm{Expression}\ast \mathrm{Coef}\right) $$

N and Coef represented gene number and coefficient value, respectively. In the training cohort, we divided HCC patients into high-risk and low-risk groups according to the median of the risk scores. A low-risk score correlates with good survival for HCC patients. A Kaplan-Meier analysis was employed to compare the survival rates between the two groups. The “SurvivalROC” package was applied to perform receiver operating characteristic (ROC) analysis. It has been reported that an area under the ROC (AUC) > 0.60 is considered suitable for prediction. Risk curves and the heatmap of risk genes were also utilized to assess this model. Besides, this model was validated by the testing group and the entire group using survival analysis, ROC analysis, risk curves, and the heatmap of risk genes.

### The prognostic value of the risk model

The risk score and other clinical parameters, including age, sex, histological grade, and the pathological stage, were evaluated with univariate and multivariate Cox analyses. Indicators were considered independent prognostic factors for *p* < 0.05 in both analyses.

### Correlation between the model and the clinical parameters

To evaluate the clinical utility of the model, the correlation between the risk score of the model and other clinical parameters were analyzed. Patients were separated into two subgroups according to age (> = 60 and < 60 years old), sex, pathologic grade (G1&2 for well-differentiated versus G3&4 for poor-differentiated), and stage (stage I&II versus stage III&IV).

### Evaluation of immune cell infiltration

The CIBERSORT (R scrip v 1.03) was applied to calculate the distribution of 22 types of infiltrating immune cells between the normal and cancer tissues based on the transcriptome profiles [21]. After calculation and filtration with *p* < 0.05, the proportions of different immune cells were exhibited in a violin plot. Meanwhile, to explore the role of the model in the reflection of the immune microenvironment in HCC patients, we downloaded the immune infiltrate data from Tumor Immune Estimation Resource [22]. Subsequently, the correlation between the risk score and the content of immune cells was also assessed using the Pearson correlation coefficient test.

## Results

### Data download

**Fig. 1:**
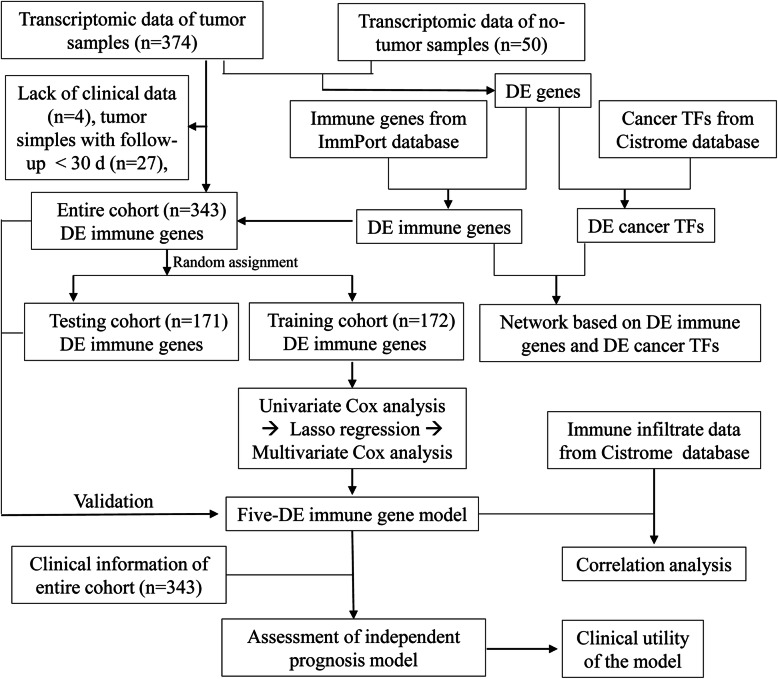
The workflow for the study. DE, differentially expressed; TFs, transcription factors

Data for 374 tumors and 50 normal controls were downloaded from the TCGA data portal. Information of the normal control samples and their corresponding tumor samples are shown in Table S1. Three tumor samples lacking clinical data and 28 tumor samples with follow-up time less than 30 days were excluded. Clinical information of the remaining 343 HCC patients is listed in Table 1. We randomly divided the 343 tumor samples into training and testing groups (Table 2). The workflow of the study is shown in Fig. 1.

**Table 1:** Clinical information of the 343 HCC patients in the entire cohort

Clinical Traits	Variable	N (Total = 343)	Percentage (%)
Survival status	Alive	220	64.1
	Dead	123	35.9
Age (years)	< 60	157	45.8
	> = 60	186	54.2
Sex	Female	110	32.1
	Male	233	67.9
Grade	G1	53	15.5
	G2	165	48.1
	G3	113	32.9
	G4	12	3.5
Pathological stage	Stage I	166	48.4
	Stage II	80	23.3
	Stage III	94	27.4
	Stage IV	3	0.9

*HCC* hepatocellular carcinoma

**Table 2:** Grouping of the HCC patients

Clinical Traits	Variable	Training Cohort	Testing Cohort	Entire Cohort
Survival status	Alive	106 (33.8%)	104 (30.3%)	220 (64.1%)
	Dead	66 (16.4%)	67 (19.5%)	123 (35.9%)

*HCC* hepatocellular carcinoma

### Detection of the DE immune genes

**Fig. 2:**
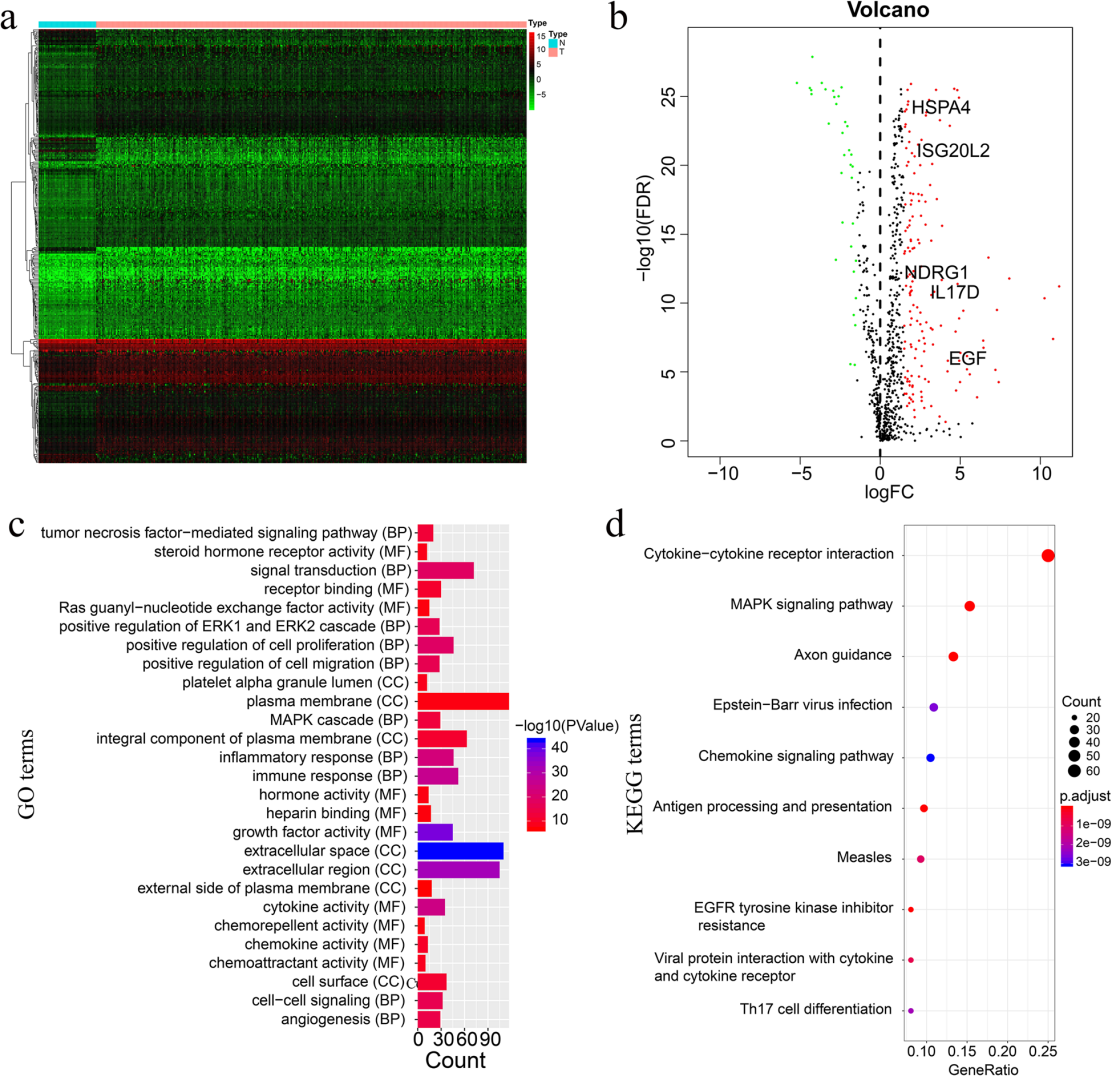
Detection of the differentially expressed (DE) immune genes. **a** The heatmap showing the DE genes. **b** The heatmap showing the DE immune genes. **c**, **d** Gene ontology (GO) and Kyoto Encyclopedia of Genes and Genomes (KEGG) analysis showing the DE immune genes

In total, 5388 DE genes (325 down-regulated and 5063 up-regulated) and 325 DE immune genes (59 down-regulated and 266 up-regulated) were identified (Table S2 and Table S3), which are shown in the volcano map and heatmap (Supplementary Figure 1a, b and Fig. 2a, b).

### GO and KEGG analyses of the DE immune genes

A total of 68 GO terms, comprising 14 molecular function terms, seven cellular component terms, and 47 biological process terms, were detected. Also, 65 enriched pathways were discovered. The significantly enriched GO terms “Inflammatory response”, “immune response”, and “growth factor activity”, and the enriched KEGG terms included “cytokine-cytokine receptor interaction”, “antigen processing and presentation”, “MAPK signaling pathway” (Table S4 and Table S5). The top 10 GO terms and pathway terms are depicted in Fig. 2c and Fig. 2d, respectively. The dysregulated genes in the top 10 pathways are shown in Supplementary Figure 2.

### Consensus clustering analysis

**Fig. 3:**
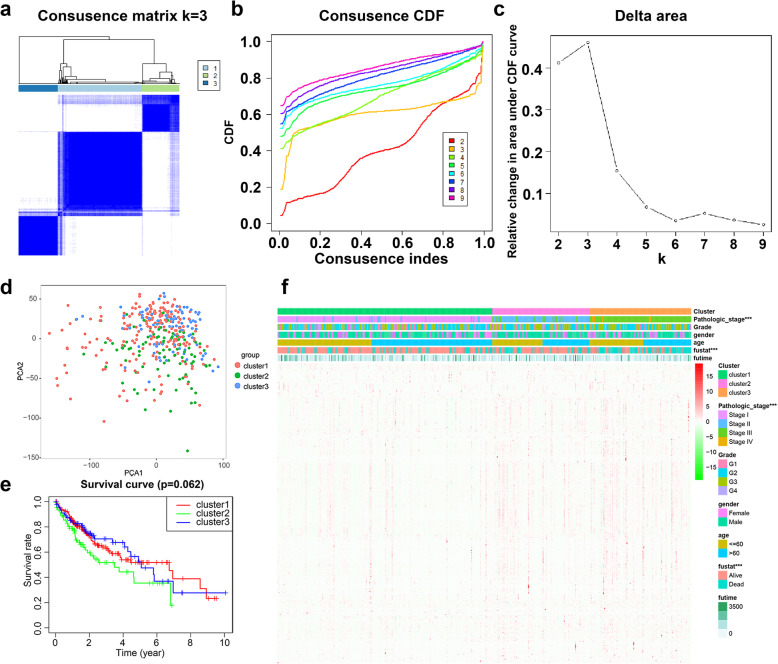
The expression of differentially expressed (DE) immune genes in consensus clustering subgroups of hepatocellular carcinoma (HCC). **a** Consensus clustering matrix for k = 3. **b**, **c** Consensus clustering cumulative distribution function for k = 2–9. **d** Principal component analysis of the three clusters. **e** Survival analysis of patients in three subgroups. **f** Heat map of clinicopathological features of the three subtypes

The 343 HCC patients were clustered into three subgroups (Fig. 3a-c). PCA was further applied to demonstrate the distinction of gene expression levels among the three subgroups (Fig. 3d). However, survival analysis showed no significant differences among the three subgroups (Fig. 3e). We further compared the clinicopathological characters of the three subgroups and found that there is a significant difference in pathologic stage and status (Fig. 3f).

### Construction of a DE immune genes-TFs network

**Fig. 4:**
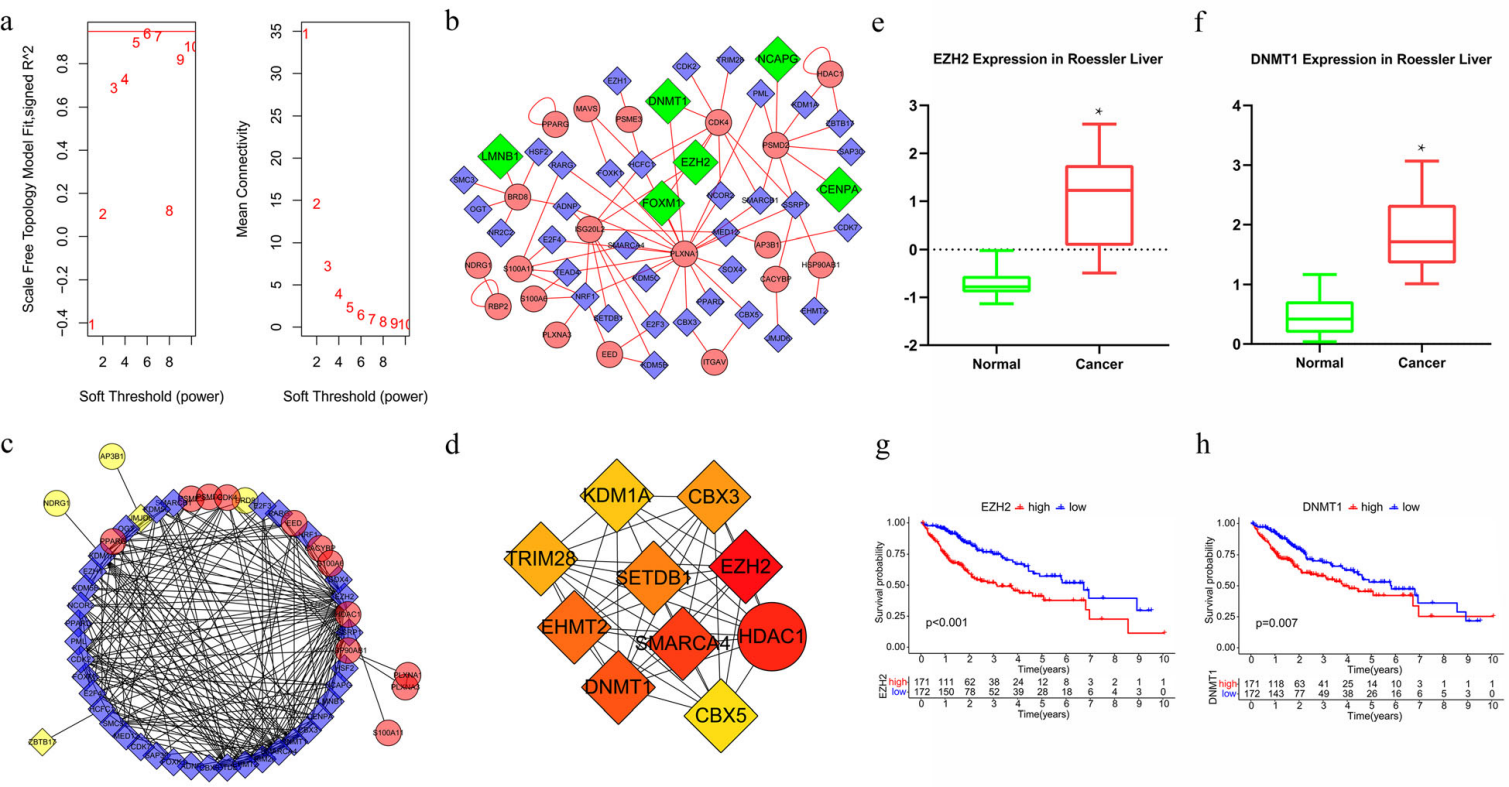
Construction of a differentially expressed (DE) immune genes-transcription factors (TFs) network. **a** The optimal soft threshold power was identified based on scale free topology model and mean connectivity. **b** The DE immune genes-TFs pairs were detected based on correlation analysis. **c** A protein-protein interaction (PPI) network. **d** Ten hub genes were detected based on the sub-PPI network. **e**, **f** The expression of EZH2 and DNMT1. **g**, **h** The survival analysis of EZH2 and DNMT1

A total of 318 tumor-related TFs were available in the Cistrome database, among which 117 DE TFs (9 down-regulated and 108 up-regulated) were detected (Table S6). The volcano map and heatmap of the DE TFs are presented in Supplementary Figure 1c and Supplementary Figure 1d. Six was chosen as the optimal soft threshold power based on scale free topology model and mean connectivity (Fig. 4a). A total of 10 genes were identified to construct the co-expression network. Meanwhile, 81 positive DE immune genes-TFs pairs were detected based on correlation analysis, involving 19 DE up-regulated immune genes and 40 DE TFs (Fig. 4b). Among them, six overlapping genes were identified, including EZH2, DNMT1, NCAPG, LMNB1, FOXM1, and CENPA (Supplementary Figure 3a). Besides, we created a PPI network and found 10 hub genes (Fig. 4c, d). Finally, we identified two common genes, namely EZH2 and DNMT1 (Supplementary Figure 3b). Both of them are high-hazard genes, and their high expression reflects poor survival (Fig. 4e-h).

### Construction of the model

**Fig. 5:**
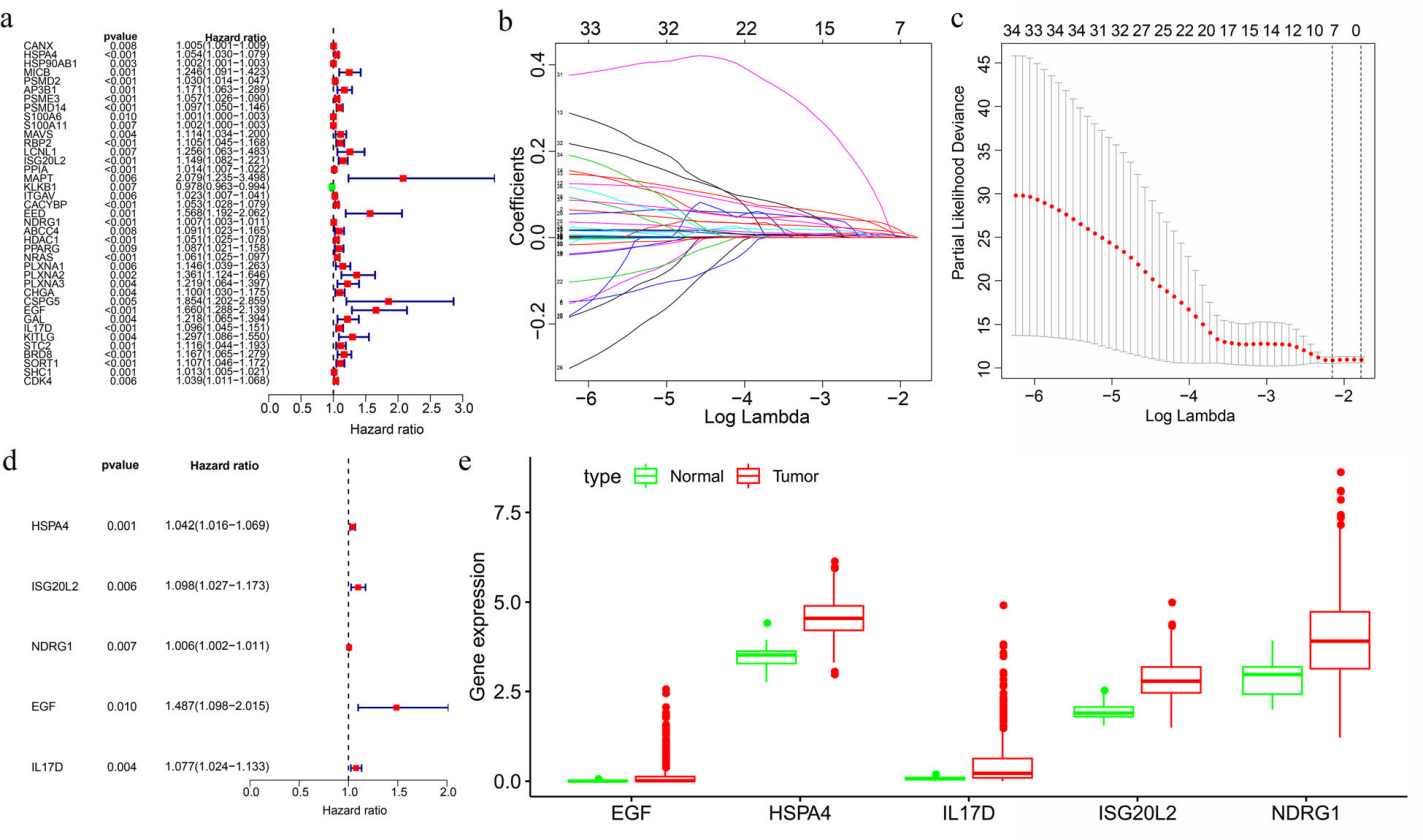
Identification of a model in the training group. **a** Screening of potential prognostic genes with univariate analysis. **b**, **c** Further analysis of possible prognostic genes with Lasso regression. **d** Identification of optimal immune genes via multivariate analysis. **e** The expression level of the optimal immune genes of the model

**Fig. 6:**
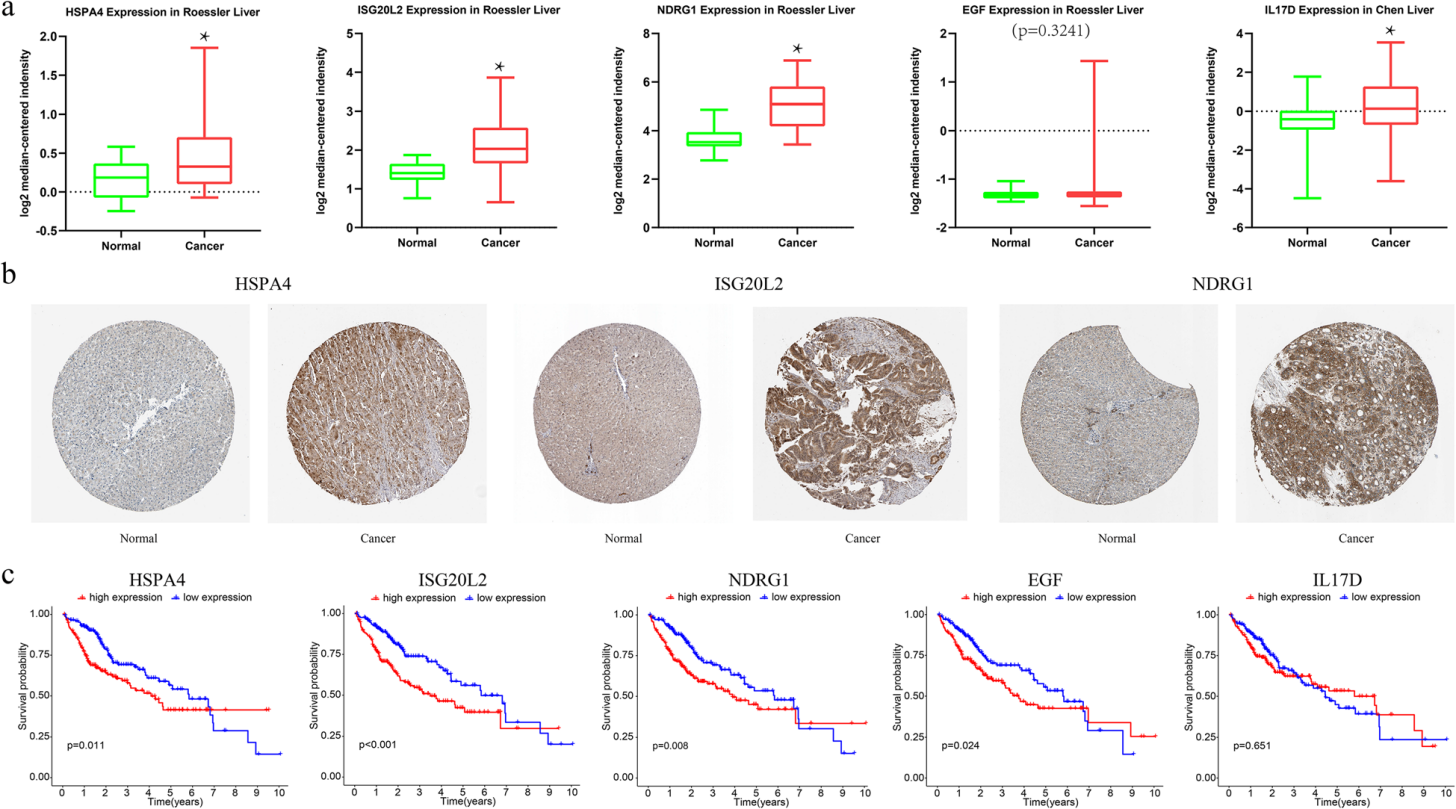
Validation of five immunes genes in the model. **a** The expression level of the immune genes in Oncomine database. **b** The protein level of the immune genes in Human Protein Atlas database. **c** Survival analysis of the immune genes based on TCGA database

At first, 39 immune genes were screened with univariate Cox analysis (Fig. 5a). Seven suitable prognostic immune genes were analyzed using Lasso regression (Fig. 5b and c). Five of them were obtained, including HSPA4, ISG20L2, NDRG1, EGF, and IL17D, all of which are high hazard genes (Fig. 5d and e, Table 3). Analysis of gene expression of these immune genes in the Oncomine database revealed a high expression level of these genes in HCC (Fig. 6a). Likewise, the protein expression level of HSPA4, ISG20L2, and NDRG1 is significantly higher in HCC (Fig. 6b). Besides, these immune genes showed association with poor survival of the patients (Fig. 6c).

**Table 3:** The expression of five immune genes in HCC patients

Gene	Mean (Normal)	Mean (HCC)	logFC	FDR
EGF	0.0050615	0.24447286	5.593952	1.57E-05
HSPA4	10.409368	24.4210186	1.230241	1.16E-24
IL17D	0.0544136	0.78016648	3.841742	2.18E-12
NDRG1	6.8347972	25.5636733	1.903125	1.52E-11
ISG20L2	2.8538117	6.7903827	1.250603	2.37E-23

*HCC* hepatocellular carcinoma

**Fig. 7:**
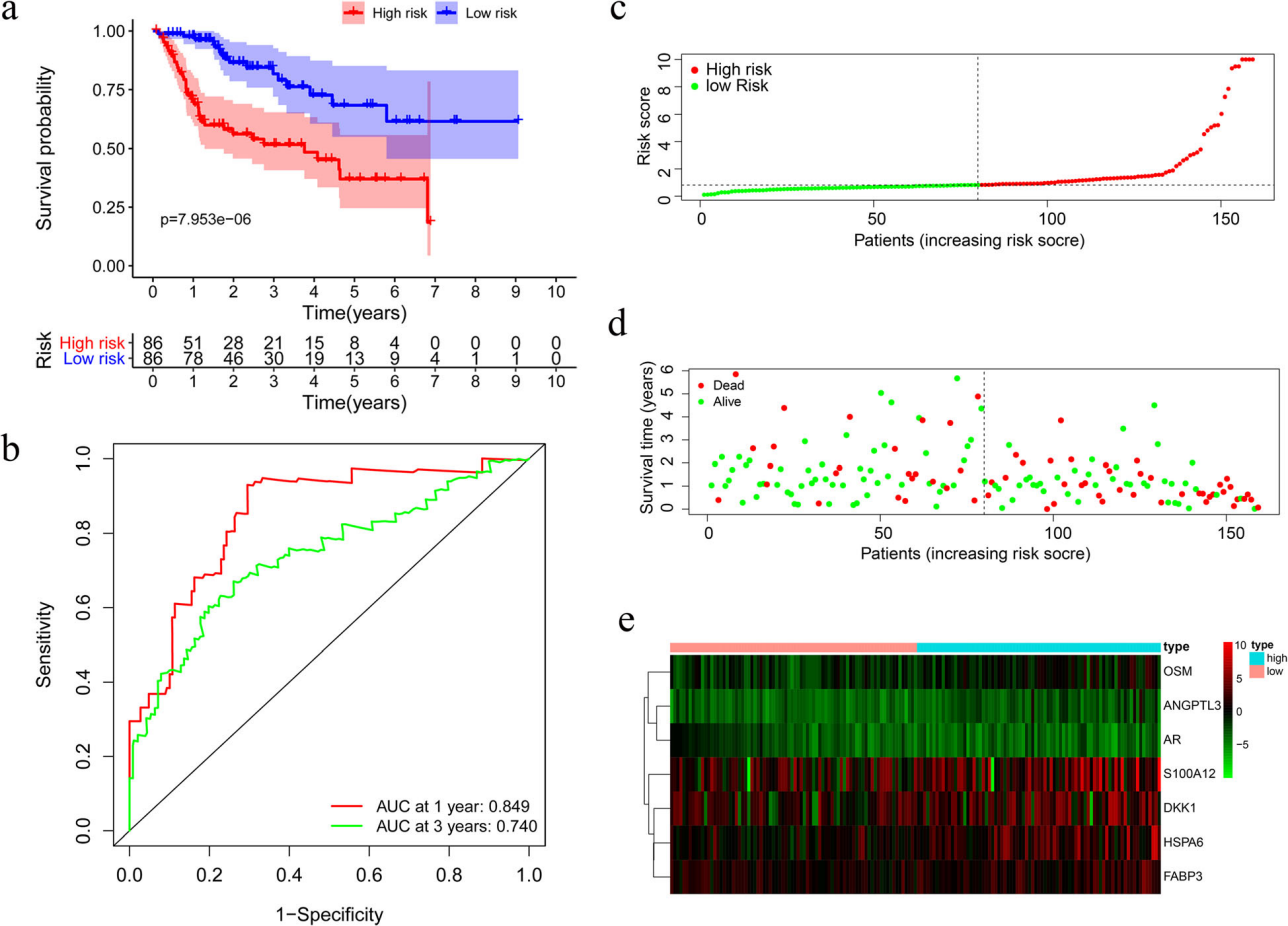
Model assessment in the training group. **a** Survival analysis between the high-risk and low-risk subgroups. **b** The time-dependent receiver operating characteristic (ROC) curve for the prognostic risk model at 1 and 3 years. **c** The risk score distribution of HCC patients. **d** Survival status scatter plots of hepatocellular carcinoma (HCC) patients. **e** Heatmap of the risk immune genes

The risk score = (0.0412 × HSPA4 expression level) + (0.0932 × ISG20L2 expression level) + (0.0062 × NDRG1 expression level) + (0.3969 × EGF expression level) + (0.0746 × IL17D expression level). All the HCC patients were classified into a high-risk group (*n* = 71) and a low-risk group (*n* = 71) based on the median risk score. OS differs significantly between the two groups (*P* = 7.953e-06) (Fig. 7a). The AUCs for the model at 1 and 3 years of overall survival are 0.849 and 0.74, respectively (Fig. 7b). Besides, we assessed the risk scores of HCC patients and examined their distribution in Fig. 7c. The survival status of HCC patients is shown on the dot plot (Fig. 7d). The heatmap shows the expression patterns of the prognostic immune genes between the two groups (Fig. 7e).

### Validation of the model

**Fig. 8:**
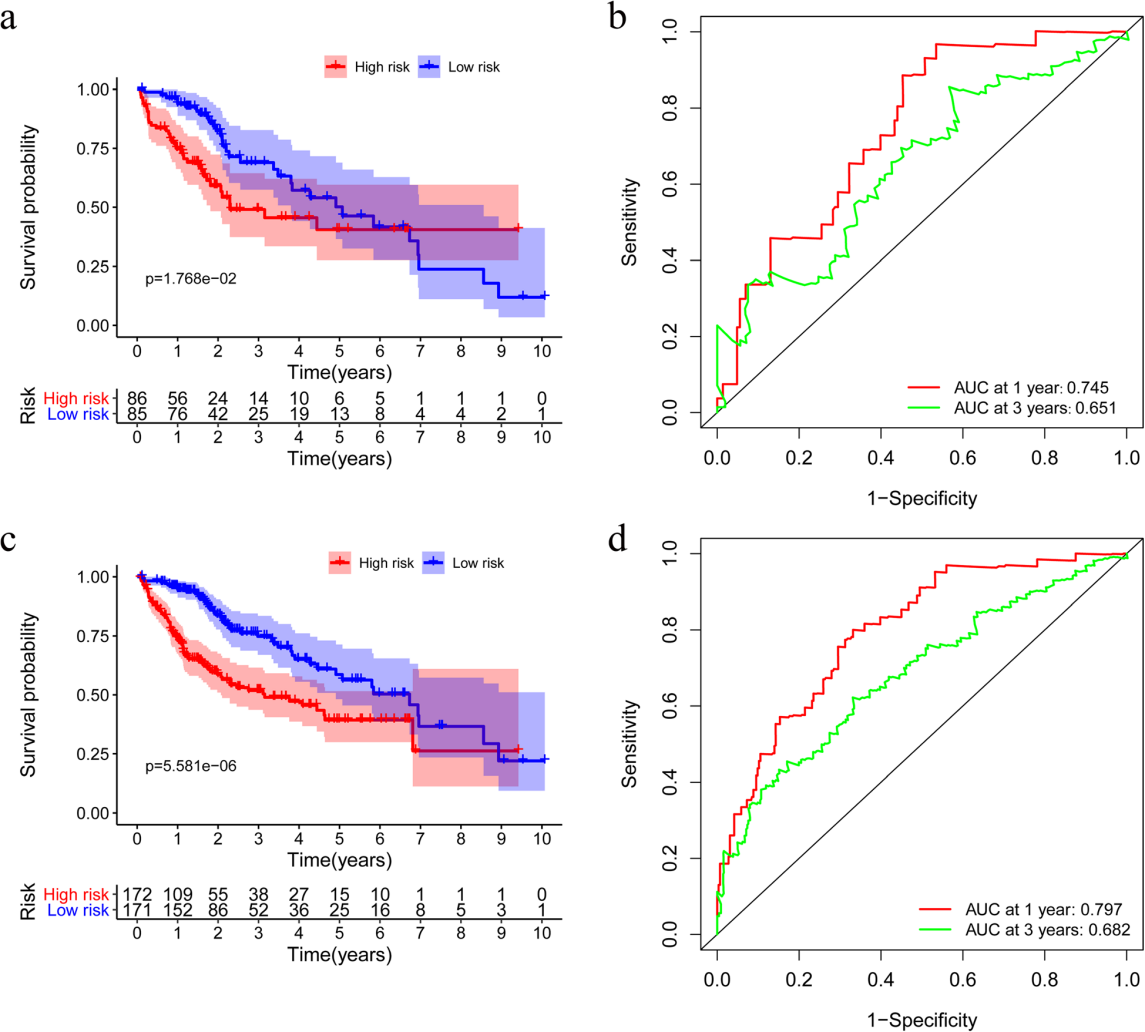
Validation of the prognostic model. **a** Overall survival (OS) of high-risk and low-risk patients in the testing group. **b** The time-dependent receiver operating characteristic (ROC) curve analysis of the testing group. **c** OS in the entire group. **d** The ROC curve analysis in the entire group

The risk score of each patient was determined in the testing and entire groups and subsequently classified into two subgroups. The survival curves are significantly different between the high-risk and low-risk subgroups in the two cohorts (*p* < 0.05) (Fig. 8a and c). Furthermore, the AUCs at 1- and 3-year in the testing cohort are 0.745 and 0.651, respectively (Fig. 8b), and those in the entire cohort are 0.797 and 0.682, respectively (Fig. 8d).

### The prognostic value of the model

**Fig. 9:**
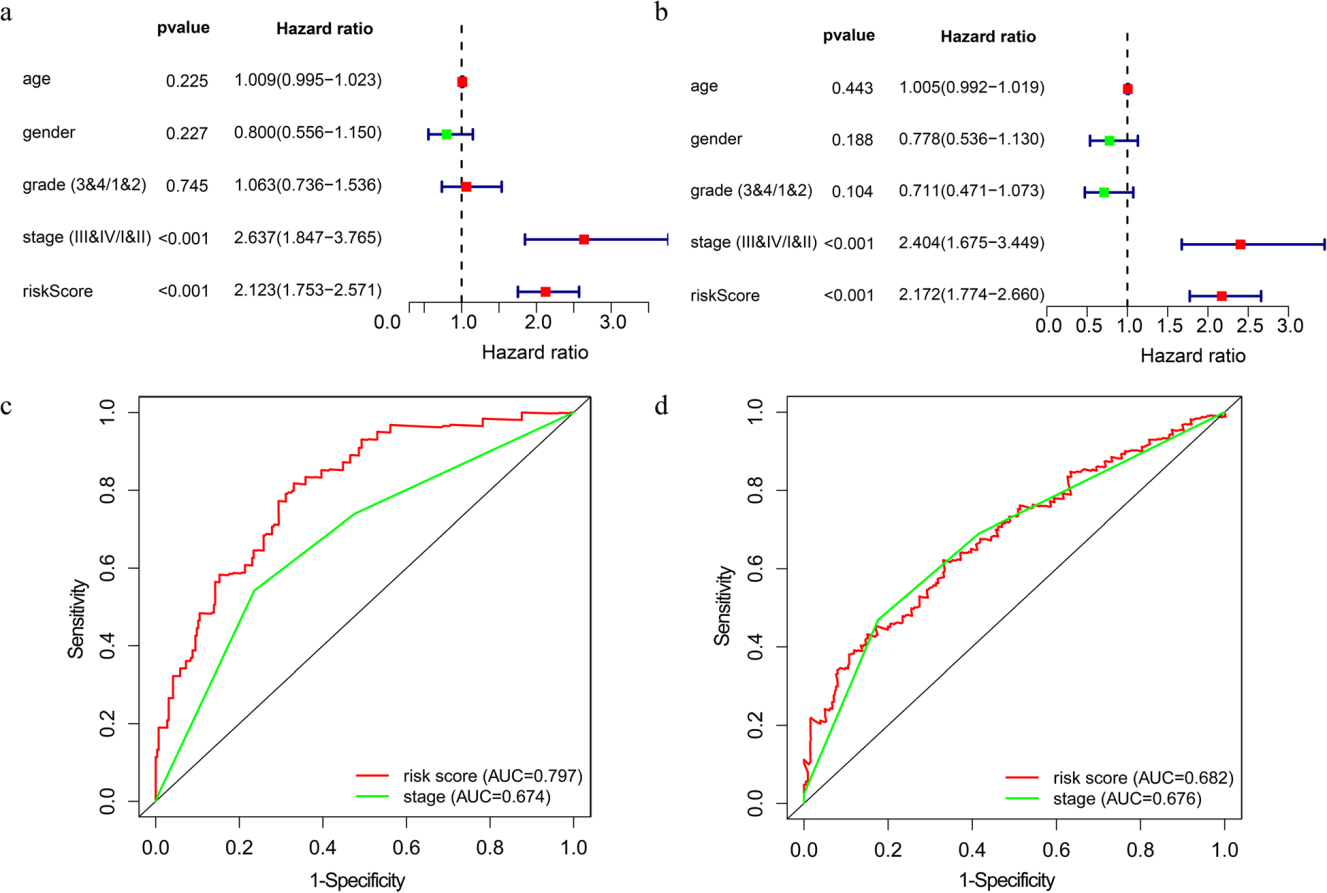
The prognostic value of the model. **a**, **b** Univariate and multivariate analyses of the entire group. **c**, **d** The time-dependent receiver operating characteristic (ROC) curve for two independent prognostic factors in the entire group at one year and three years

For the entire group, the risk score and the pathological stage were found to be closely associated with OS (*p* < 0.001) (Fig. 9a and b). These results suggest that the model, as well as the pathological stage, are independent prognostic factors. Interestingly, further comparison demonstrated that the risk score is more accurate in predicting OS at one and 3 years, as compared with the pathological stage (Fig. 9c and d).

### Correlation between the model and the clinical parameters

**Fig. 10:**
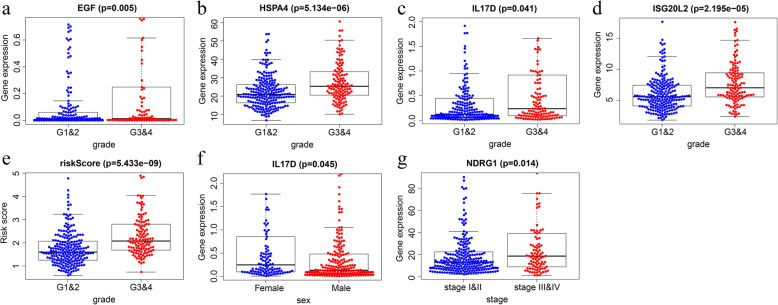
Clinical utility of the model in the entire group. **a-d** The correlation of *EGF, HSPA4, IL17D, and ISG20L2* expression with the histological grade. **e** The relationship of the risk score with the histological grade. **f** The correlation of *IL17D* expression with sex. **g** The association of *NDRG1* expression with the pathological stage

In the entire group, the values of several factors (EGF, HSPA4, IL17D, ISG20L2, and the risk score) are positively related to the histological grade of HCC (*p* < 0.05) (Fig. 10a–e). The expression level of IL17D was higher in females than in males (*p* < 0.05) (Fig. 10f). Besides, as NDRG expression increased, the value of the pathological stage decreased (*p* < 0.05) (Fig. 10g).

### Evaluation of immune cell infiltration

**Fig. 11:**
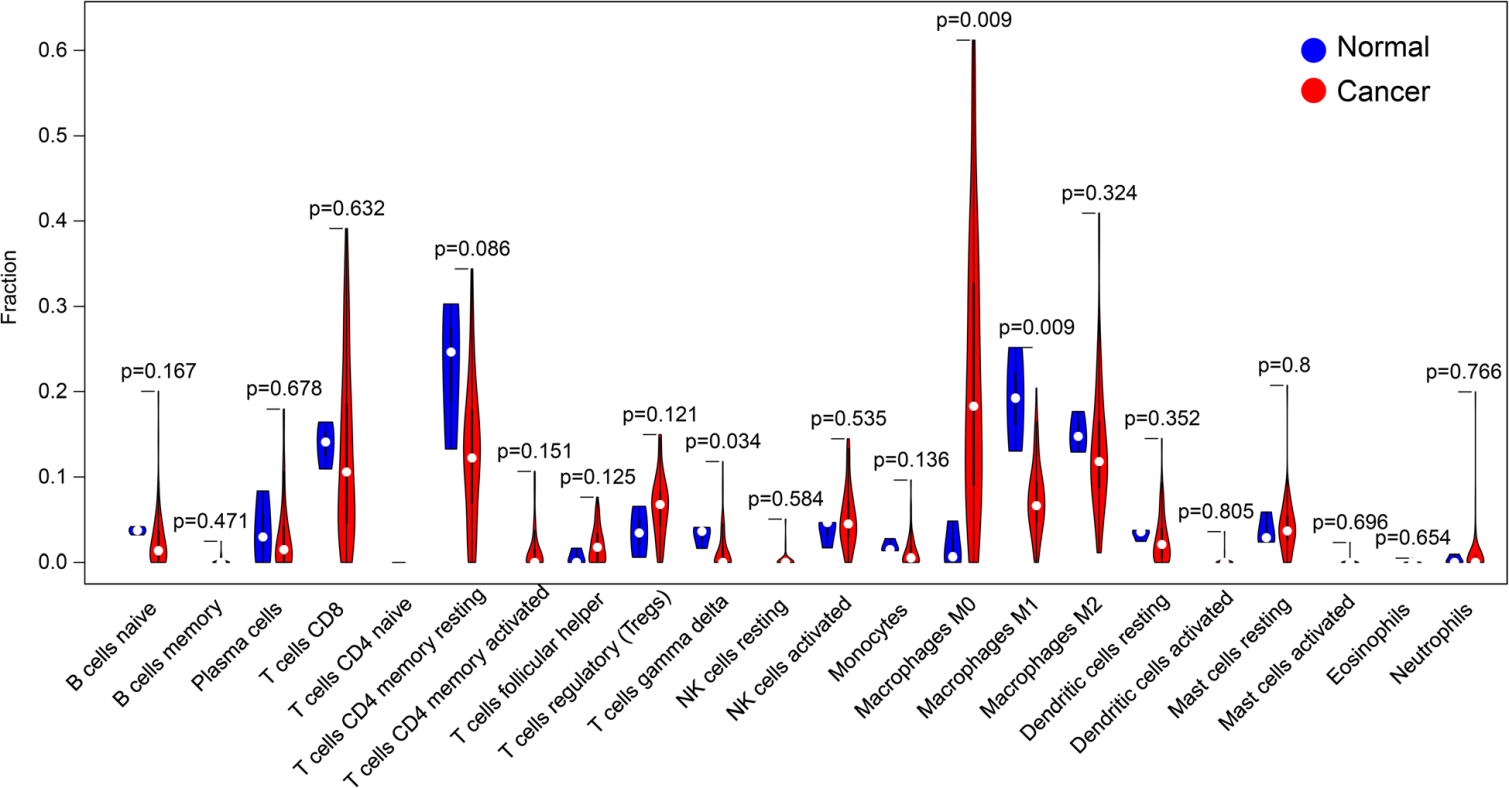
Comparisons of abundance of infiltrating immune cells between normal and cancer tissues

**Fig. 12:**
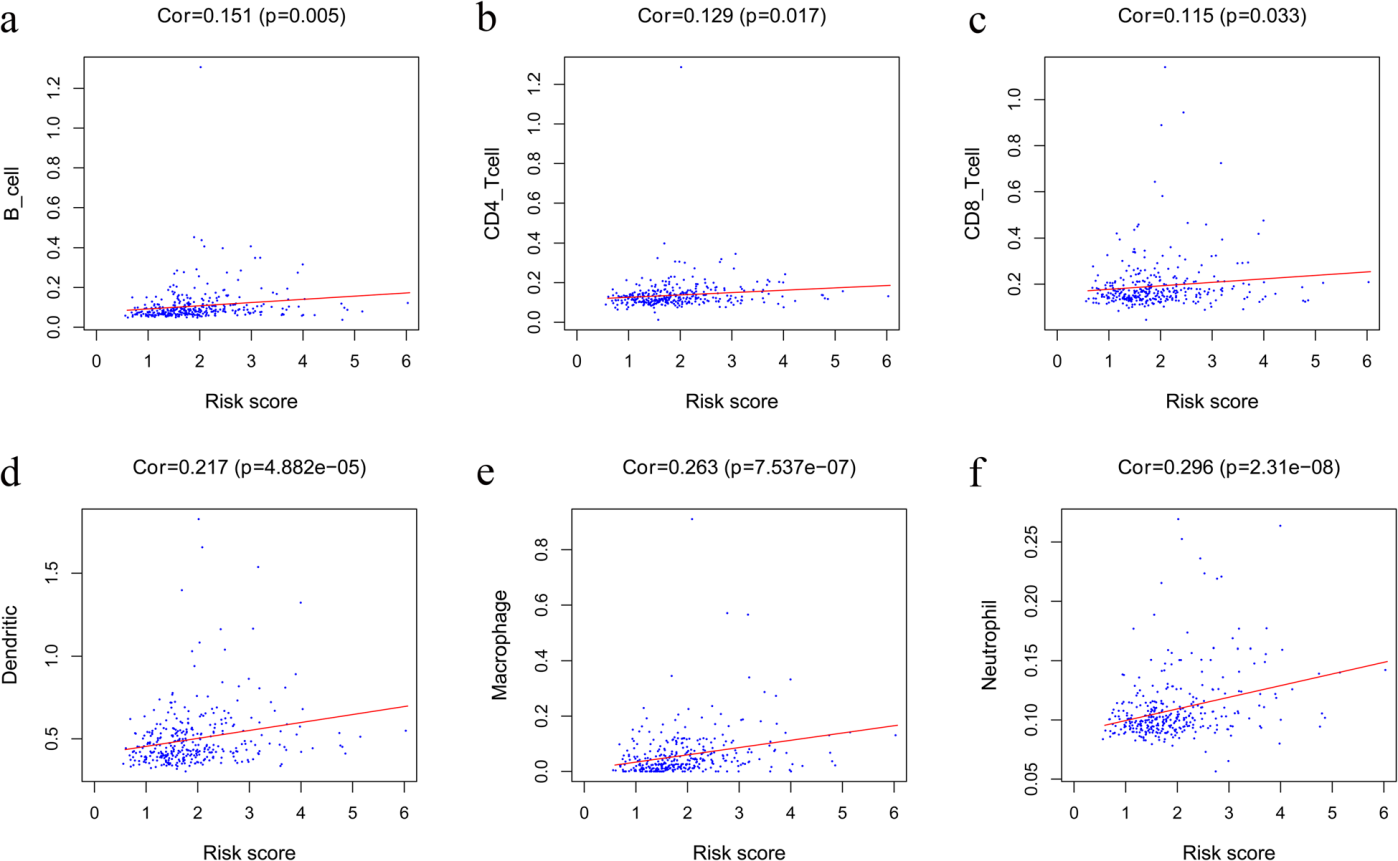
Correlation of the risk score with immune cell infiltration in the entire group. **a** B cells. **b** CD4^+^ T cells. **c** CD8^+^ T cells. **d** Dendritic cells. **e** Macrophages. **f** Neutrophils

After calculation and filtration with *p* < 0.05, the proportions of different immune cells between 3 normal and 69 cancer specimens resulted in a violin plot. We found that the proportion of T cell gamma-delta and macrophages M1 in normal tissues was significantly higher than that in cancer tissues, while the proportion of macrophages M0 in normal tissues is significantly lower (Fig. 11). Besides, we found that the risk score is positively associated with the content of the immune cells, including B cell, CD4^+^ T cells, CD8^+^ T cells, dendritic cells, macrophages, and neutrophils, in HCC samples (*p* < 0.05) (Fig. 12a-f). This indicates that the immune gene model is reliable and can reflect the status of the immune microenvironment in HCC patients.

## Discussion

In this study, we identified an immune gene model that can serve as an independent prognostic factor for HCC. It is closely related to other clinical factors and the tumor immune microenvironment of HCC. Also, GO and KEGG analyses of the DE immune genes and the network between the DE immune genes and TFs were conducted, which may guide future research of HCC.

As described in the results, the five-DE immune gene model consists of HSPA4, ISG20L2, NDRG1, EGF, and IL17D. Due to the difference of these genes between HCC and normal tissues, these genes may also have utility in the early diagnosis of HCC. Additionally, all the five DE immune genes may have the potential to be new molecular targets for immunotherapy. HSPA4, also known as Apg-2, is a member of the HSP110 family. It is expressed in many organs [23] and can be induced by various conditions, including oncogenic stress. Gotoh et al. [24] demonstrated that HSPA4 is overexpressed in HCC. Duzgun et al. [25] revealed that the overexpression of HSPA4 has correlated with worse OS in head and neck squamous cell carcinoma and invasive carcinoma of the breast. NDRG1 has been demonstrated to be a biomarker for metastasis and to indicate poor prognosis in HCC [26, 27], which is in line with our finding. Lu et al. [28] found that NDRG1 is up-regulated in HCC and may be used as a potential therapeutic target for HCC. ISG20L2, as a target of miR-139-3p, has also been found to be related to HCC prognosis [29].

In this study, we also established a risk score that can serve as an independent prognostic variable. The risk score correlates with the histological grade and the pathological stage but seems to provide a better prediction than the pathological stage. Tumor-infiltrating immune cells are an important component of the tumor microenvironment and are regarded as the “seventh marker feature” of the tumor [30]. Previous reports have shown that immune cellular infiltration is a vital factor affecting the treatment efficacy as well as the prognosis of HCC [31, 32]. Ma et al. [33] had reported that PD1 Hi CD8+ T cells correlate with poor clinical outcomes in HCC. Ju et al. [34] had found that overexpression of BRAP is correlated with poor prognosis and has a positive correlation with infiltrating immune cells. In our study, it was found that the risk score is highly associated with the infiltration of immune cells. The result indicates that the risk model may reflect the status of the tumor microenvironment of HCC and further supports the prognostic value of this risk model.

In our analysis, the cellular component terms included “extracellular space”, “extracellular region”, “cell surface”, and “integral component of plasma membrane”. These signify interactions between cancer cells and the tumor microenvironment. The latter is important for tumor proliferation, invasion, and metastasis. Also, biological process terms included “inflammatory response”, “immune response”, and “growth factor activity”. It is well known that HCC often occurs in the context of chronic liver disease with cirrhosis [35, 36]. As for the KEGG terms, cytokine-cytokine receptor interaction is an inflammatory pathway, which is highly associated with the progress of HCC [37]. Antigen processing and presentation are also found to be enriched pathways in HCC [38]. Besides, we identified the DE TFs and detected the DE immune gene-TF pairs with high correlation. The enriched functions and pathways, as well as highly related TFs, may be target for future studies.

The current study has some advantages. First, the model was established using multiple algorithms and verified with two validating groups. Second, the risk score may be used to independently predict the prognosis of HCC. Third, the risk model may also reflect the tumor immune microenvironment of HCC.

The limitations of this study are several. First, the prognostic risk model was built based on the public domain databases, and not confirmed in real-world clinical settings. Secondly, the identified DE immune genes and TFs, as well as the enriched functions and pathways, require further research.

## Conclusion

We identified a five-immune gene model, which can be used as an independent prognostic parameter for HCC.

## Supplementary Information

**Additional file 1: Table S1.** Information of adjacent normal cases and their corresponding tumor cases in HCC

**Additional file 2: Table S2.** DE genes in HCC

**Additional file 3: Table S3.** DE immune genes in HCC

**Additional file 4: Table S4.** Significantly enriched GO terms

**Additional file 5: Table S5.** Significantly enriched KEGG terms

**Additional file 6: Table S6.** DE TFs in HCC

**Additional file 7: Supplementary Figure 1. (a)** The volcano map of the DE genes. **(b)** The heatmap of the DE genes. **(c)** The volcano map of the DE TFs. (**d)** The heatmap of the DE TFs.

**Additional file 8: Supplementary Figure 2.** The dysregulated genes were shown in the top ten pathways with the Pathview package.

**Additional file 9: Supplementary Figure 3.** Identification of the common genes based on WGCNA analysis, Spearman correlation analysis, and protein-protein interaction (PPI) network.

## Data Availability

All analyzed data are included in this published article and its supplementary information file. The original data are available upon reasonable request to the corresponding author.

## Abbreviation

HCC: Hepatocellular carcinoma
OS: Overall survival
ICB: Immune checkpoint blockade
DE: Differentially expressed
TCGA: The Cancer Genome Atlas
TFs: Transcription factors
FDR: False discovery rate
FC: Fold change
*DAVID*: The Database for Annotation, Visualization, and Integrated Discovery
GO: Gene ontology
KEGG: Kyoto Encyclopedia of Genes and Genomes
ROC: Receiver operating characteristic
AUC: Area under the ROC

## References

[1] Cronin KA, Lake AJ, Scott S, Sherman RL, Noone AM, Howlader N, Henley SJ, Anderson RN, Firth AU, Ma J (2018). Annual report to the nation on the status of Cancer, part I: national cancer statistics. Cancer.

[2] Bray F, Ferlay J, Soerjomataram I, Siegel RL, Torre LA, Jemal A (2018). Global cancer statistics 2018: GLOBOCAN estimates of incidence and mortality worldwide for 36 cancers in 185 countries. CA Cancer J Clin.

[3] Zongyi Y, Xiaowu L (2019). Immunotherapy for hepatocellular carcinoma. Cancer Lett.

[4] Zhang X, Li J, Ghoshal K, Fernandez S, Li L (2019). Identification of a subtype of hepatocellular carcinoma with poor prognosis based on expression of genes within the glucose metabolic pathway. Cancers.

[5] Chen Q, Wang M, Wang M, Jin S, Xiao SY, Tian S (2018). Expansile invasive growth pattern is definite evidence for the diagnosis of small hepatocellular carcinomas: a comparative study of 37 cases. Hum Pathol.

[6] Zhu R, Yang X, Guo W, Xu XJ, Zhu L (2019). An eight-mRNA signature predicts the prognosis of patients with bladder urothelial carcinoma. Peer J.

[7] Siu LL, Ivy SP, Dixon EL, Gravell AE, Reeves SA, Rosner GL (2017). Challenges and opportunities in adapting clinical trial Design for Immunotherapies. Clin Cancer Res.

[8] Tai D, Choo SP, Chew V (2019). Rationale of immunotherapy in hepatocellular carcinoma and its potential biomarkers. Cancers.

[9] Joerger M, Guller U, Bastian S, Driessen C, von Moos R (2019). Prolonged tumor response associated with sequential immune checkpoint inhibitor combination treatment and regorafenib in a patient with advanced pretreated hepatocellular carcinoma. J Gastrointest Oncol.

[10] Agdashian D, ElGindi M, Xie C, Sandhu M, Pratt D, Kleiner DE, Figg WD, Rytlewski JA, Sanders C, Yusko EC (2019). The effect of anti-CTLA4 treatment on peripheral and intra-tumoral T cells in patients with hepatocellular carcinoma. Cancer Immunol Immunother.

[11] Liu F, Zeng G, Zhou S, He X, Sun N, Zhu X, Hu A (2018). Blocking Tim-3 or/and PD-1 reverses dysfunction of tumor-infiltrating lymphocytes in HBV-related hepatocellular carcinoma. Bull Cancer.

[12] Zhou G, Sprengers D, Boor PPC, Doukas M, Schutz H, Mancham S, Pedroza-Gonzalez A, Polak WG, de Jonge J, Gaspersz M (2017). Antibodies against immune checkpoint molecules restore functions of tumor-infiltrating t cells in hepatocellular carcinomas. Gastroenterology.

[13] Li W, Wang H, Ma Z, Zhang J, Ou-Yang W, Qi Y, Liu J (2019). Multi-omics analysis of microenvironment characteristics and immune escape mechanisms of hepatocellular carcinoma. Front Oncol.

[14] Dong Z, Chen Y, Yang C, Zhang M, Chen A, Yang J, Huang Y (2019). STAT gene family mRNA expression and prognostic value in hepatocellular carcinoma. Onco Targets Ther.

[15] Mei S, Meyer CA, Zheng R, Qin Q, Wu Q, Jiang P, Li B, Shi X, Wang B, Fan J (2017). Cistrome Cancer: a web resource for integrative gene regulation modeling in Cancer. Cancer Res.

[16] Bhattacharya S, Andorf S, Gomes L, Dunn P, Schaefer H, Pontius J, Berger P, Desborough V, Smith T, Campbell J (2014). ImmPort: disseminating data to the public for the future of immunology. Immunol Res.

[17] Wang Z, Gao L, Guo X, Feng C, Lian W, Deng K, Xing B (2020). Development of a Nomogram with alternative splicing signatures for predicting the prognosis of Glioblastoma: a study based on large-scale sequencing data. Front Oncol.

[18] Chen P, He J, Ye H, Jiang S, Li Y, Li X, Wan J (2020). Comprehensive analysis of prognostic alternative splicing signatures in endometrial Cancer. Front Genet.

[19] Rhodes DR, Yu J, Shanker K, Deshpande N, Varambally R, Ghosh D, Barrette T, Pandey A, Chinnaiyan AM (2004). ONCOMINE: a cancer microarray database and integrated data-mining platform. Neoplasia.

[20] Colwill K, Gräslund S (2011). A roadmap to generate renewable protein binders to the human proteome. Nat Methods.

[21] Newman AM, Liu CL, Green MR, Gentles AJ, Feng W, Xu Y, Hoang CD, Diehn M, Alizadeh AA (2015). Robust enumeration of cell subsets from tissue expression profiles. Nat Methods.

[22] Li B, Severson E, Pignon JC, Zhao H, Li T, Novak J, Jiang P, Shen H, Aster JC, Rodig S (2016). Comprehensive analyses of tumor immunity: implications for cancer immunotherapy. Genome Biol.

[23] Kaneko Y, Kimura T, Kishishita M, Noda Y, Fujita J (1997). Cloning of apg-2 encoding a novel member of heat shock protein 110 family. Gene.

[24] Gotoh K, Nonoguchi K, Higashitsuji H, Kaneko Y, Sakurai T, Sumitomo Y, Itoh K, Subjeck JR, Fujita J (2004). Apg-2 has a chaperone-like activity similar to Hsp110 and is overexpressed in hepatocellular carcinomas. FEBS Lett.

[25] Duzgun MB, Theofilatos K, Georgakilas AG, Pavlopoulou A (2019). A Bioinformatic approach for the identification of molecular determinants of resistance/sensitivity to Cancer thermotherapy. Oxidative Med Cell Longev.

[26] Cheng J, Xie HY, Xu X, Wu J, Wei X, Su R, Zhang W, Lv Z, Zheng S, Zhou L (2011). NDRG1 as a biomarker for metastasis, recurrence and of poor prognosis in hepatocellular carcinoma. Cancer Lett.

[27] Luo Q, Wang CQ, Yang LY, Gao XM, Sun HT, Zhang Y, Zhang KL, Zhu Y, Zheng Y, Sheng YY (2018). FOXQ1/NDRG1 axis exacerbates hepatocellular carcinoma initiation via enhancing crosstalk between fibroblasts and tumor cells. Cancer Lett.

[28] Lu WJ, Chua MS, So SK (2014). Suppressing N-Myc downstream regulated gene 1 reactivates senescence signaling and inhibits tumor growth in hepatocellular carcinoma. Carcinogenesis.

[29] Zhu Y, Zhou C, He Q (2019). High miR-139-3p expression predicts a better prognosis for hepatocellular carcinoma: a pooled analysis. J Int Med Res.

[30] Junttila MR, de Sauvage FJ (2013). Influence of tumour micro-environment heterogeneity on therapeutic response. Nature.

[31] McGranahan N, Furness AJ, Rosenthal R, Ramskov S, Lyngaa R, Saini SK, Jamal-Hanjani M, Wilson GA, Birkbak NJ, Hiley CT (2016). Clonal neoantigens elicit T cell immunoreactivity and sensitivity to immune checkpoint blockade. Science.

[32] Chen QF, Li W, Wu PH, Shen LJ, Huang ZL (2019). Significance of tumor-infiltrating immunocytes for predicting prognosis of hepatitis B virus-related hepatocellular carcinoma. World J Gastroenterol.

[33] Ma J, Zheng B, Goswami S, Meng L, Zhang D, Cao C, Li T, Zhu F, Ma L, Zhang Z (2019). PD1(hi) CD8(+) T cells correlate with exhausted signature and poor clinical outcome in hepatocellular carcinoma. J Immunother Cancer.

[34] Ju Q, Li XM, Zhang H, Zhao YJ (2020). BRCA1-associated protein is a potential prognostic biomarker and is correlated with immune infiltration in liver hepatocellular carcinoma: a pan-Cancer analysis. Front Mol Biosci.

[35] Sia D, Jiao Y, Martinez-Quetglas I, Kuchuk O, Villacorta-Martin C, Castro de Moura M, Putra J, Camprecios G, Bassaganyas L, Akers N (2017). Identification of an immune-specific class of hepatocellular carcinoma. Based Mol Features Gastroenterol.

[36] Xiao SY, Wang HL, Hart J, Fleming D, Beard MR (2001). cDNA arrays and immunohistochemistry identification of CD10/CALLA expression in hepatocellular carcinoma. Am J Pathol.

[37] Souza T, Jennen D, van Delft J, van Herwijnen M, Kyrtoupolos S, Kleinjans J (2016). New insights into BaP-induced toxicity: role of major metabolites in transcriptomics and contribution to hepatocarcinogenesis. Arch Toxicol.

[38] Zhang C, Peng L, Zhang Y, Liu Z, Li W, Chen S, Li G (2017). The identification of key genes and pathways in hepatocellular carcinoma by bioinformatics analysis of high-throughput data. Med Oncol.

