# Supplementary material for: Identification of a five-immune gene model as an independent prognostic factor in hepatocellular carcinoma
Source: BMC Cancer. 2021 Mar 16;21:278. doi: 10.1186/s12885-021-08012-2 (PMC7962305; doi:10.1186/s12885-021-08012-2)
Supplement: Supplementary file 1 — Additional file 1: Table S1. Information of adjacent normal cases and their corresponding tumor cases in HCC [file 12885_2021_8012_MOESM1_ESM.docx]

**Table S1** Information of adjacent normal cases and their corresponding tumor cases in HCC

| Adjacent normal cases | Hepatocellular carcinoma cases |
| --- | --- |
| TCGA-DD-A1EG-11A-11R-A213-07 | TCGA-DD-A1EG-01A-11R-A213-07 |
| TCGA-DD-A3A8-11A-11R-A22L-07 | TCGA-DD-A3A8-01A-11R-A22L-07 |
| TCGA-DD-A11D-11A-12R-A131-07 | TCGA-DD-A11D-01A-12R-A131-07 |
| TCGA-BC-A10Q-11A-11R-A131-07 | TCGA-BC-A10Q-01A-11R-A131-07 |
| TCGA-DD-A1EE-11A-11R-A131-07 | TCGA-DD-A1EE-01A-11R-A131-07 |
| TCGA-DD-A3A6-11A-11R-A22L-07 | TCGA-DD-A3A6-01A-11R-A22L-07 |
| TCGA-BC-A216-11A-11R-A155-07 | TCGA-BC-A216-01A-11R-A155-07 |
| TCGA-DD-A1EC-11A-11R-A131-07 | TCGA-DD-A1EC-01A-11R-A131-07 |
| TCGA-FV-A23B-11A-11R-A16W-07 | TCGA-FV-A23B-01A-11R-A16W-07 |
| TCGA-EP-A12J-11A-11R-A131-07 | TCGA-EP-A12J-01A-11R-A131-07 |
| TCGA-DD-A1EI-11A-11R-A131-07 | TCGA-DD-A1EI-01A-11R-A131-07 |
| TCGA-DD-A3A3-11A-11R-A22L-07 | TCGA-DD-A3A3-01A-11R-A22L-07 |
| TCGA-FV-A3I1-11A-11R-A22L-07 | TCGA-FV-A3I1-01A-11R-A22L-07 |
| TCGA-G3-A3CH-11A-11R-A22L-07 | TCGA-G3-A3CH-01A-11R-A22L-07 |
| TCGA-BC-A10T-11A-11R-A131-07 | TCGA-BC-A10T-01A-11R-A131-07 |
| TCGA-BC-A10X-11A-11R-A131-07 | TCGA-BC-A10X-01A-11R-A131-07 |
| TCGA-BC-A10R-11A-11R-A131-07 | TCGA-BC-A10R-01A-11R-A131-07 |
| TCGA-DD-A1EH-11A-11R-A131-07 | TCGA-DD-A1EH-01A-11R-A131-07 |
| TCGA-DD-A119-11A-11R-A131-07 | TCGA-DD-A119-01A-11R-A131-07 |
| TCGA-DD-A3A4-11A-11R-A22L-07 | TCGA-DD-A3A4-01A-11R-A22L-07 |
| TCGA-DD-A39Z-11A-21R-A213-07 | TCGA-DD-A39Z-01A-21R-A213-07 |
| TCGA-DD-A11C-11A-11R-A131-07 | TCGA-DD-A11C-01A-11R-A131-07 |
| TCGA-DD-A116-11A-12R-A26B-07 | TCGA-DD-A116-01A-12R-A26B-07 |
| TCGA-FV-A3I0-11A-11R-A22L-07 | TCGA-FV-A3I0-01A-11R-A22L-07 |
| TCGA-DD-A113-11A-12R-A131-07 | TCGA-DD-A113-01A-12R-A131-07 |
| TCGA-BC-A10U-11A-11R-A131-07 | TCGA-BC-A10U-01A-11R-A131-07 |
| TCGA-FV-A2QR-11A-11R-A213-07 | TCGA-FV-A2QR-01A-11R-A213-07 |
| TCGA-DD-A3A2-11A-11R-A213-07 | TCGA-DD-A3A2-01A-11R-A213-07 |
| TCGA-FV-A3R2-11A-11R-A22L-07 | TCGA-FV-A3R2-01A-11R-A22L-07 |
| TCGA-DD-A11A-11A-11R-A131-07 | TCGA-DD-A01A-01A-11R-A131-07 |
| TCGA-EP-A26S-11A-12R-A16W-07 | TCGA-EP-A26S-01A-12R-A16W-07 |
| TCGA-BC-A10Z-11A-11R-A131-07 | TCGA-BC-A10Z-01A-11R-A131-07 |
| TCGA-DD-A114-11A-12R-A131-07 | TCGA-DD-A114-01A-12R-A131-07 |
| TCGA-BC-A10W-11A-11R-A131-07 | TCGA-BC-A10W-01A-11R-A131-07 |
| TCGA-BD-A3EP-11A-12R-A22L-07 | TCGA-BD-A3EP-01A-12R-A22L-07 |
| TCGA-BC-A10Y-11A-11R-A131-07 | TCGA-BC-A10Y-01A-11R-A131-07 |
| TCGA-DD-A3A1-11A-11R-A213-07 | TCGA-DD-A3A1-01A-11R-A213-07 |
| TCGA-EP-A3RK-11A-11R-A22L-07 | TCGA-EP-A3RK-01A-11R-A22L-07 |
| TCGA-DD-A1EB-11A-11R-A131-07 | TCGA-DD-A1EB-01A-11R-A131-07 |
| TCGA-DD-A1EJ-11A-11R-A155-07 | TCGA-DD-A1EJ-01A-11R-A155-07 |
| TCGA-DD-A39W-11A-11R-A213-07 | TCGA-DD-A39W-01A-11R-A213-07 |
| TCGA-DD-A3A5-11A-11R-A22L-07 | TCGA-DD-A3A5-01A-11R-A22L-07 |
| TCGA-BC-A110-11A-11R-A131-07 | TCGA-BC-A110-01A-11R-A131-07 |
| TCGA-ES-A2HT-11A-11R-A180-07 | TCGA-ES-A2HT-01A-11R-A180-07 |
| TCGA-DD-A11B-11A-11R-A131-07 | TCGA-DD-A11B-01A-11R-A131-07 |
| TCGA-DD-A39X-11A-11R-A213-07 | TCGA-DD-A39X-01A-11R-A213-07 |
| TCGA-DD-A118-11A-11R-A131-07 | TCGA-DD-A118-01A-11R-A131-07 |
| TCGA-DD-A1EL-11A-11R-A155-07 | TCGA-DD-A1EL-01A-11R-A155-07 |
| TCGA-BD-A2L6-11A-21R-A213-07 | TCGA-BD-A2L6-01A-21R-A213-07 |
| TCGA-DD-A39V-11A-11R-A213-07 | TCGA-DD-A39V-01A-11R-A213-07 |

HCC hepatocellular carcinoma
