# Supplementary material for: Identification of a five-immune gene model as an independent prognostic factor in hepatocellular carcinoma
Source: BMC Cancer. 2021 Mar 16;21:278. doi: 10.1186/s12885-021-08012-2 (PMC7962305; doi:10.1186/s12885-021-08012-2)
Supplement: Supplementary file 2 — Additional file 2: Table S2. DE genes in HCC [file 12885_2021_8012_MOESM2_ESM.docx]

**Table S2** DE genes in HCC

| gene | logFC | pValue | FDR |
| --- | --- | --- | --- |
| SESTD1 | 1.510175 | 4.99E-18 | 1.90E-17 |
| NUP37 | 1.522795 | 1.28E-26 | 4.16E-25 |
| EXOSC2 | 1.000049 | 3.60E-20 | 1.80E-19 |
| CCDC112 | 1.721131 | 6.73E-12 | 1.45E-11 |
| LRIG2 | 1.055296 | 4.93E-17 | 1.68E-16 |
| MYOF | 1.4619 | 0.000112 | 0.000149 |
| CTSC | 1.291931 | 2.92E-07 | 4.62E-07 |
| RHOBTB2 | 1.697115 | 1.14E-16 | 3.75E-16 |
| TCERG1 | 1.399412 | 2.50E-21 | 1.52E-20 |
| COPA | 1.177769 | 4.54E-21 | 2.65E-20 |
| ROMO1 | 1.310601 | 6.44E-20 | 3.11E-19 |
| ZNF766 | 1.104738 | 2.38E-22 | 1.75E-21 |
| CDC42EP5 | 1.040629 | 1.49E-08 | 2.56E-08 |
| TRNAU1AP | 1.196546 | 4.72E-23 | 4.08E-22 |
| MRAP2 | 4.696279 | 2.19E-15 | 6.28E-15 |
| ATG4B | 1.058824 | 4.52E-24 | 5.12E-23 |
| TRIO | 1.230062 | 3.16E-15 | 8.91E-15 |
| RASGRF2 | 2.49854 | 7.64E-26 | 1.65E-24 |
| FAM118B | 1.000543 | 2.09E-21 | 1.30E-20 |
| OSGIN1 | 1.50324 | 1.32E-07 | 2.13E-07 |
| RFC3 | 1.787961 | 4.30E-22 | 3.03E-21 |
| FUBP1 | 1.082917 | 8.63E-15 | 2.34E-14 |
| KCTD2 | 1.148412 | 4.05E-23 | 3.55E-22 |
| SLC9A6 | 1.043758 | 2.17E-17 | 7.72E-17 |
| RPS18 | 1.301158 | 1.80E-18 | 7.16E-18 |
| RMI1 | 1.28039 | 2.60E-16 | 8.21E-16 |
| SMYD2 | 1.642185 | 4.79E-20 | 2.36E-19 |
| FAM111B | 3.243395 | 1.03E-23 | 1.07E-22 |
| PARPBP | 3.473941 | 6.68E-27 | 2.52E-25 |
| PEX2 | 1.011642 | 1.23E-23 | 1.25E-22 |
| GTPBP4 | 1.161702 | 2.13E-20 | 1.11E-19 |
| PCK1 | -1.65532 | 1.33E-19 | 6.12E-19 |
| ZNF385C | 2.621356 | 3.83E-13 | 9.07E-13 |
| FAM83H | 2.078308 | 2.70E-24 | 3.26E-23 |
| C17orf67 | 1.155501 | 0.001768 | 0.002182 |
| AURKA | 3.394565 | 4.06E-28 | 4.50E-26 |
| DDX39B | 1.700148 | 4.11E-18 | 1.58E-17 |
| RASSF4 | 1.363526 | 1.51E-16 | 4.89E-16 |
| HAVCR1 | 6.049339 | 1.75E-08 | 2.99E-08 |
| SHLD1 | 1.445578 | 7.42E-21 | 4.16E-20 |
| CKLF | 1.626423 | 7.16E-23 | 5.92E-22 |
| ZNF737 | 1.554609 | 0.000294 | 0.000381 |
| ZBED4 | 1.298443 | 4.55E-19 | 1.95E-18 |
| CDC20 | 5.073739 | 3.49E-28 | 4.13E-26 |
| BRD9 | 1.371673 | 7.64E-26 | 1.65E-24 |
| ADCK1 | 1.263151 | 4.46E-24 | 5.07E-23 |
| PPIA | 1.300985 | 9.81E-28 | 7.34E-26 |
| RECQL | 1.078552 | 1.25E-08 | 2.16E-08 |
| LMAN2 | 1.06958 | 1.78E-25 | 3.32E-24 |
| UTP23 | 1.106521 | 9.43E-19 | 3.89E-18 |
| MAP3K9 | 2.024884 | 1.18E-24 | 1.62E-23 |
| SACS | 1.227472 | 1.39E-07 | 2.24E-07 |
| DIP2A | 1.054349 | 3.81E-15 | 1.07E-14 |
| TATDN2 | 1.341692 | 1.28E-25 | 2.52E-24 |
| MTA3 | 1.469284 | 3.56E-25 | 5.83E-24 |
| C8orf44 | 1.883851 | 4.59E-26 | 1.11E-24 |
| HIP1 | 1.049547 | 1.97E-12 | 4.42E-12 |
| NCK2 | 1.296592 | 0.003093 | 0.003757 |
| ZNF251 | 1.913264 | 1.93E-27 | 1.15E-25 |
| SETD4 | 1.416993 | 7.34E-26 | 1.62E-24 |
| BRSK1 | 2.448159 | 2.71E-21 | 1.65E-20 |
| RPL18 | 1.079345 | 9.79E-15 | 2.64E-14 |
| FOXK1 | 1.929439 | 7.21E-25 | 1.07E-23 |
| NNMT | -1.73849 | 1.15E-17 | 4.22E-17 |
| LOXL2 | 2.31714 | 1.17E-24 | 1.60E-23 |
| MAP4 | 1.074502 | 2.07E-21 | 1.28E-20 |
| TMEM178B | 3.379415 | 1.52E-06 | 2.29E-06 |
| ARHGEF2 | 1.733705 | 1.18E-15 | 3.48E-15 |
| BAZ2A | 1.168856 | 2.68E-16 | 8.44E-16 |
| C19orf57 | 2.022551 | 7.24E-19 | 3.03E-18 |
| CD163 | -1.33493 | 9.77E-13 | 2.24E-12 |
| DGCR2 | 1.154264 | 1.10E-21 | 7.19E-21 |
| CEP170B | 1.057027 | 1.06E-14 | 2.84E-14 |
| MXD4 | 1.183863 | 1.50E-20 | 8.04E-20 |
| SAFB2 | 1.019433 | 7.03E-25 | 1.04E-23 |
| HIST1H2BO | 2.675472 | 9.43E-14 | 2.35E-13 |
| ZSCAN2 | 1.662467 | 2.45E-27 | 1.35E-25 |
| SLX4 | 1.515826 | 8.30E-22 | 5.55E-21 |
| HSPA12B | 1.360343 | 2.35E-15 | 6.71E-15 |
| C14orf132 | 1.415752 | 0.024572 | 0.027903 |
| PALLD | 1.314775 | 7.88E-09 | 1.38E-08 |
| FAM111A | 1.254751 | 1.25E-17 | 4.58E-17 |
| ARPC1B | 1.413977 | 3.91E-16 | 1.21E-15 |
| ADCK2 | 1.2518 | 1.96E-22 | 1.48E-21 |
| VPS52 | 1.011619 | 2.01E-19 | 9.06E-19 |
| PFKFB4 | 2.321973 | 5.38E-18 | 2.04E-17 |
| SMIM4 | 1.347212 | 1.16E-16 | 3.79E-16 |
| WDR97 | 2.132935 | 1.31E-19 | 6.06E-19 |
| EXD3 | 1.010073 | 3.61E-18 | 1.39E-17 |
| COLGALT1 | 1.230006 | 4.78E-23 | 4.12E-22 |
| FAM13C | 1.542383 | 1.20E-08 | 2.08E-08 |
| ORC1 | 3.681368 | 3.62E-26 | 9.34E-25 |
| EXOC3 | 1.04135 | 6.34E-25 | 9.57E-24 |
| AKR1C3 | 2.237927 | 1.18E-23 | 1.20E-22 |
| PDE7B | -1.02603 | 1.31E-15 | 3.83E-15 |
| UGT2B11 | 3.843339 | 1.32E-12 | 3.01E-12 |
| NCAPH | 3.732156 | 2.71E-26 | 7.37E-25 |
| RFC4 | 2.440069 | 4.41E-28 | 4.65E-26 |
| GINS3 | 2.145934 | 7.94E-24 | 8.47E-23 |
| IDO1 | 1.450023 | 0.000361 | 0.000465 |
| RPL23 | 1.130947 | 5.38E-18 | 2.04E-17 |
| TSEN54 | 1.707803 | 1.30E-26 | 4.19E-25 |
| MARK1 | 1.705822 | 0.007032 | 0.008294 |
| AP4B1 | 1.04159 | 1.42E-21 | 9.06E-21 |
| LAMTOR2 | 1.204087 | 8.19E-23 | 6.69E-22 |
| CDC23 | 1.161207 | 3.74E-23 | 3.32E-22 |
| ATP6V1C2 | 1.711267 | 3.90E-22 | 2.78E-21 |
| HBA2 | -1.1307 | 4.51E-16 | 1.38E-15 |
| C22orf46 | 1.199855 | 1.89E-20 | 9.92E-20 |
| AAAS | 1.049542 | 7.14E-27 | 2.66E-25 |
| THAP8 | 1.422343 | 1.25E-25 | 2.48E-24 |
| FAM217B | 1.302387 | 5.06E-08 | 8.39E-08 |
| PDRG1 | 1.458863 | 3.14E-26 | 8.36E-25 |
| AOC1 | -1.26973 | 0.001028 | 0.001287 |
| TYRO3 | 2.436811 | 9.42E-07 | 1.44E-06 |
| ABAT | -1.01311 | 3.57E-16 | 1.11E-15 |
| WIPF3 | 1.625134 | 0.016486 | 0.01893 |
| DTNBP1 | 1.277421 | 2.19E-16 | 6.95E-16 |
| PLCXD3 | -1.14942 | 3.25E-13 | 7.76E-13 |
| CAPN3 | 1.218126 | 3.22E-05 | 4.45E-05 |
| LIPE | 1.613224 | 0.000249 | 0.000324 |
| MRPS21 | 1.560209 | 2.44E-26 | 6.85E-25 |
| SLC16A11 | 2.028149 | 0.004455 | 0.005339 |
| DTNB | 1.109074 | 6.34E-23 | 5.31E-22 |
| SMC1B | 4.50422 | 3.38E-14 | 8.74E-14 |
| C1RL | -1.0394 | 1.27E-22 | 1.00E-21 |
| ATP8B3 | 1.747603 | 2.24E-10 | 4.36E-10 |
| SAMD1 | 1.598047 | 3.72E-26 | 9.50E-25 |
| RASSF1 | 1.233667 | 1.45E-22 | 1.13E-21 |
| ULK3 | 1.254871 | 3.47E-23 | 3.11E-22 |
| ADAMTS9 | 1.508931 | 1.85E-11 | 3.87E-11 |
| PTGS2 | -1.70841 | 5.31E-17 | 1.80E-16 |
| CCDC154 | 2.533877 | 2.89E-17 | 1.01E-16 |
| FOXC1 | 1.547421 | 0.002096 | 0.002575 |
| ZGLP1 | 1.393614 | 4.76E-13 | 1.12E-12 |
| ITGA9 | -1.21078 | 1.10E-16 | 3.61E-16 |
| MAPK13 | 2.31912 | 0.00835 | 0.009799 |
| ZNF48 | 1.184075 | 2.78E-23 | 2.57E-22 |
| TSGA10 | 1.105198 | 1.99E-14 | 5.26E-14 |
| ZNF233 | 2.18828 | 2.70E-12 | 6.01E-12 |
| CHKB | 1.484327 | 9.12E-19 | 3.77E-18 |
| PSMD10 | 1.361273 | 5.51E-26 | 1.29E-24 |
| VCX | 7.558725 | 7.69E-09 | 1.35E-08 |
| NPAS2 | 1.323001 | 1.34E-10 | 2.64E-10 |
| TMEM165 | 1.318104 | 9.90E-18 | 3.66E-17 |
| TEX29 | 1.288216 | 3.41E-05 | 4.71E-05 |
| GABBR2 | 4.823818 | 2.33E-08 | 3.95E-08 |
| HIST1H2BG | 2.866537 | 3.19E-15 | 8.98E-15 |
| RAB15 | 1.003126 | 2.01E-10 | 3.91E-10 |
| LSM8 | 1.325713 | 6.58E-24 | 7.20E-23 |
| SLC2A1 | 1.897715 | 3.92E-05 | 5.39E-05 |
| TTYH3 | 1.745688 | 1.06E-13 | 2.63E-13 |
| DCDC2 | 2.432896 | 0.00239 | 0.002926 |
| UGGT2 | 1.10431 | 2.18E-20 | 1.13E-19 |
| DDAH2 | 1.391538 | 6.97E-15 | 1.91E-14 |
| P2RY13 | -1.27663 | 5.10E-12 | 1.11E-11 |
| GPR160 | 1.638066 | 3.89E-06 | 5.70E-06 |
| GPR35 | 3.650368 | 2.24E-10 | 4.36E-10 |
| COL15A1 | 4.427621 | 1.69E-28 | 3.39E-26 |
| PPIP5K1 | 1.41851 | 1.36E-23 | 1.36E-22 |
| SFXN3 | 1.233038 | 1.35E-06 | 2.05E-06 |
| C8orf59 | 1.61097 | 2.94E-25 | 5.03E-24 |
| ZFPL1 | 1.134656 | 1.28E-22 | 1.01E-21 |
| RAB6B | 2.252029 | 1.26E-24 | 1.71E-23 |
| PSIP1 | 1.071995 | 2.83E-10 | 5.44E-10 |
| ASAP2 | 1.62557 | 2.39E-05 | 3.34E-05 |
| DCTPP1 | 1.261937 | 2.47E-22 | 1.81E-21 |
| MOGAT2 | -1.70326 | 4.68E-20 | 2.31E-19 |
| ANGPTL6 | -2.41451 | 3.51E-29 | 2.26E-26 |
| FBXL6 | 1.721102 | 2.75E-25 | 4.74E-24 |
| STX1A | 2.305504 | 2.10E-25 | 3.79E-24 |
| AC005041.1 | 1.223202 | 5.75E-07 | 8.93E-07 |
| PKN1 | 1.705371 | 1.92E-20 | 1.00E-19 |
| SKA3 | 4.294851 | 1.75E-27 | 1.08E-25 |
| CEMIP | 3.406533 | 1.04E-13 | 2.58E-13 |
| LRRC1 | 2.972135 | 1.47E-15 | 4.30E-15 |
| BICDL1 | 2.912434 | 4.29E-18 | 1.64E-17 |
| JCAD | 2.489637 | 1.07E-23 | 1.10E-22 |
| RNF20 | 1.009081 | 7.90E-20 | 3.76E-19 |
| HSPA2 | 1.02681 | 5.10E-07 | 7.93E-07 |
| NELFA | 1.198571 | 1.87E-24 | 2.40E-23 |
| NXT2 | 1.125583 | 3.43E-14 | 8.87E-14 |
| NELFB | 1.081426 | 1.03E-23 | 1.07E-22 |
| VSIG10 | 1.417689 | 1.95E-21 | 1.22E-20 |
| CBX8 | 1.871865 | 3.25E-27 | 1.60E-25 |
| SYNGR1 | 1.796226 | 0.002294 | 0.002812 |
| EDNRA | 1.110924 | 0.000584 | 0.000743 |
| UPK3A | 6.448294 | 1.16E-06 | 1.76E-06 |
| MRPL9 | 1.328968 | 9.91E-26 | 2.04E-24 |
| TIGD3 | 2.789713 | 4.35E-19 | 1.87E-18 |
| RAB5IF | 1.010577 | 2.24E-17 | 7.96E-17 |
| RRAS | 1.078834 | 2.61E-12 | 5.81E-12 |
| RAB3GAP2 | 1.081104 | 4.86E-19 | 2.08E-18 |
| C1orf56 | 1.126453 | 4.93E-17 | 1.68E-16 |
| SPACA6 | 1.434964 | 0.039629 | 0.04437 |
| FANCD2 | 2.949769 | 2.65E-25 | 4.61E-24 |
| PRKAA2 | 2.543237 | 2.30E-10 | 4.46E-10 |
| FAM169A | 1.467101 | 0.005475 | 0.006516 |
| ZBTB39 | 1.015695 | 3.05E-12 | 6.76E-12 |
| FLNA | 1.445094 | 1.25E-05 | 1.77E-05 |
| C5AR2 | 1.493777 | 0.00197 | 0.002425 |
| LLGL1 | 1.615669 | 5.08E-19 | 2.16E-18 |
| ZNF611 | 1.161636 | 0.000352 | 0.000453 |
| RAD51B | 1.037436 | 2.19E-13 | 5.30E-13 |
| DENND4B | 1.724638 | 8.59E-26 | 1.81E-24 |
| AC005726.1 | 1.524325 | 1.71E-16 | 5.50E-16 |
| ZNF324 | 1.043013 | 3.65E-23 | 3.25E-22 |
| FNBP1L | 1.172545 | 2.13E-12 | 4.77E-12 |
| FEN1 | 2.347888 | 2.08E-28 | 3.44E-26 |
| RIPOR3 | -1.21241 | 9.14E-23 | 7.39E-22 |
| SAP30BP | 1.052849 | 3.61E-25 | 5.88E-24 |
| RPL17 | 1.301016 | 7.02E-22 | 4.76E-21 |
| HSPB1 | 2.248492 | 7.65E-24 | 8.20E-23 |
| USP22 | 1.309206 | 2.18E-19 | 9.75E-19 |
| CCDC102A | 1.258748 | 8.35E-09 | 1.46E-08 |
| CD3EAP | 1.367369 | 1.38E-17 | 5.01E-17 |
| SNRPN | 1.06725 | 0.001539 | 0.001906 |
| CCT4 | 1.090565 | 1.08E-23 | 1.11E-22 |
| VRK1 | 1.558908 | 5.25E-24 | 5.86E-23 |
| FBLN1 | 2.273495 | 1.30E-05 | 1.84E-05 |
| DDX55 | 1.184619 | 1.77E-21 | 1.11E-20 |
| KPNA7 | 2.812158 | 1.91E-13 | 4.65E-13 |
| RPL10A | 1.037847 | 2.11E-17 | 7.50E-17 |
| ILDR2 | 3.067651 | 2.64E-06 | 3.92E-06 |
| SH3RF3 | 1.915949 | 0.000132 | 0.000174 |
| SLC52A3 | 2.525462 | 0.000156 | 0.000205 |
| RIOK1 | 1.146883 | 3.29E-19 | 1.44E-18 |
| TMEM171 | 1.291318 | 0.000147 | 0.000194 |
| KIAA1328 | 1.021859 | 6.68E-16 | 2.02E-15 |
| PPFIA3 | 1.473764 | 5.87E-19 | 2.48E-18 |
| FOXF2 | 3.446861 | 3.16E-11 | 6.52E-11 |
| JMJD7-PLA2G4B | 1.449593 | 1.59E-10 | 3.12E-10 |
| MFSD3 | 1.365771 | 2.60E-16 | 8.21E-16 |
| ASRGL1 | 1.942226 | 1.13E-09 | 2.10E-09 |
| C19orf71 | 1.333589 | 9.02E-11 | 1.80E-10 |
| ZNF682 | 1.633137 | 2.64E-06 | 3.92E-06 |
| CDC42SE1 | 1.139457 | 4.79E-16 | 1.47E-15 |
| ACTL10 | 1.857743 | 8.59E-11 | 1.72E-10 |
| FGF13 | 2.505838 | 1.98E-09 | 3.60E-09 |
| PSMD2 | 1.147132 | 1.11E-24 | 1.54E-23 |
| PYGB | 2.112338 | 8.70E-26 | 1.83E-24 |
| NFKBIE | 1.133919 | 3.31E-10 | 6.34E-10 |
| PSEN2 | 1.114665 | 2.86E-15 | 8.10E-15 |
| NAGPA | 1.405558 | 2.52E-25 | 4.41E-24 |
| PIGT | 1.34109 | 2.00E-27 | 1.16E-25 |
| SMKR1 | 3.548971 | 3.34E-10 | 6.40E-10 |
| B4GALT6 | 1.151967 | 2.51E-07 | 3.99E-07 |
| KANSL3 | 1.055996 | 3.48E-19 | 1.52E-18 |
| TMED9 | 1.180578 | 1.46E-25 | 2.80E-24 |
| ITGA11 | 1.772251 | 2.55E-07 | 4.04E-07 |
| ASPHD2 | 1.187026 | 2.04E-05 | 2.85E-05 |
| ZNF165 | 1.510451 | 5.04E-11 | 1.02E-10 |
| FBXO27 | 1.600419 | 2.16E-07 | 3.45E-07 |
| TYSND1 | 1.293592 | 2.18E-19 | 9.75E-19 |
| SRCAP | 1.110065 | 8.26E-19 | 3.43E-18 |
| OLFML2B | 3.92054 | 2.35E-27 | 1.32E-25 |
| FBXO9 | 1.098141 | 1.88E-19 | 8.51E-19 |
| ZSCAN31 | 2.049833 | 2.63E-14 | 6.87E-14 |
| AFM | -1.39507 | 1.17E-18 | 4.78E-18 |
| CSE1L | 1.309631 | 9.04E-26 | 1.89E-24 |
| SERPINE1 | -1.52049 | 2.29E-11 | 4.75E-11 |
| BIK | 2.561551 | 4.18E-06 | 6.11E-06 |
| SHANK2 | 1.097127 | 1.26E-07 | 2.04E-07 |
| ARPIN | 1.072346 | 2.19E-14 | 5.77E-14 |
| EDIL3 | 3.540858 | 1.56E-22 | 1.20E-21 |
| PTBP2 | 1.222976 | 4.99E-16 | 1.52E-15 |
| CHMP4C | 1.244689 | 2.37E-14 | 6.21E-14 |
| BAHCC1 | 1.401112 | 2.02E-13 | 4.90E-13 |
| CSNK1G2 | 1.051889 | 1.30E-21 | 8.39E-21 |
| SCARA3 | 2.29082 | 8.34E-10 | 1.56E-09 |
| ZNF8 | 1.581376 | 2.17E-21 | 1.34E-20 |
| COL1A1 | 2.512046 | 6.53E-07 | 1.01E-06 |
| UBE2Q2 | 1.573051 | 6.95E-13 | 1.61E-12 |
| LRP4 | 2.404813 | 0.001344 | 0.00167 |
| PLGLB2 | -1.33755 | 1.93E-19 | 8.68E-19 |
| CEL | 3.974989 | 3.70E-16 | 1.15E-15 |
| E2F1 | 4.35381 | 1.32E-27 | 8.85E-26 |
| STMN1 | 2.829074 | 1.90E-27 | 1.14E-25 |
| C11orf49 | 1.323878 | 3.87E-12 | 8.48E-12 |
| NDC1 | 1.175298 | 4.53E-20 | 2.23E-19 |
| ZNF600 | 1.406438 | 1.00E-10 | 1.99E-10 |
| LRAT | -2.22883 | 9.54E-25 | 1.34E-23 |
| RAB7B | 1.152146 | 1.62E-07 | 2.59E-07 |
| EIF3D | 1.165908 | 1.78E-22 | 1.36E-21 |
| IL1RN | -1.51967 | 2.19E-11 | 4.56E-11 |
| COMP | 3.563985 | 2.43E-05 | 3.40E-05 |
| ZNF200 | 1.287966 | 4.19E-24 | 4.78E-23 |
| DMGDH | -1.03683 | 5.46E-16 | 1.66E-15 |
| CPT1B | 1.717098 | 1.74E-14 | 4.60E-14 |
| METTL3 | 1.352311 | 7.37E-24 | 7.97E-23 |
| KANSL1 | 1.059323 | 3.77E-15 | 1.06E-14 |
| GTSF1 | 5.016995 | 0.003354 | 0.004064 |
| CANT1 | 1.367891 | 6.59E-25 | 9.86E-24 |
| MAP4K2 | 1.373798 | 1.10E-21 | 7.19E-21 |
| EBNA1BP2 | 1.041275 | 3.95E-20 | 1.96E-19 |
| HIST1H4K | 2.530986 | 2.34E-14 | 6.15E-14 |
| HINFP | 1.169688 | 4.43E-25 | 6.99E-24 |
| RMND5B | 1.027214 | 5.23E-26 | 1.24E-24 |
| ANKZF1 | 1.503337 | 2.35E-26 | 6.64E-25 |
| PACSIN1 | 2.860259 | 0.000207 | 0.000271 |
| MGME1 | 1.192981 | 1.73E-21 | 1.09E-20 |
| CCDC138 | 1.685004 | 1.98E-18 | 7.85E-18 |
| MIER2 | 1.441715 | 3.25E-20 | 1.64E-19 |
| MYO5A | 1.296135 | 1.28E-09 | 2.36E-09 |
| SLC9A3 | 4.60506 | 4.29E-10 | 8.15E-10 |
| ZHX1-C8orf76 | 2.0274 | 1.26E-24 | 1.71E-23 |
| PARP2 | 1.316984 | 1.52E-25 | 2.89E-24 |
| HS6ST1 | 1.019626 | 6.11E-10 | 1.15E-09 |
| FNDC10 | 2.342191 | 2.40E-07 | 3.82E-07 |
| PIGU | 1.783746 | 6.36E-28 | 5.57E-26 |
| KCNE3 | 1.419387 | 6.02E-13 | 1.40E-12 |
| IQCD | 3.366684 | 5.48E-21 | 3.15E-20 |
| HIST1H2BC | 1.097409 | 0.017625 | 0.0202 |
| ZNF77 | 1.33446 | 5.07E-20 | 2.48E-19 |
| ANKDD1A | 1.204518 | 4.97E-12 | 1.08E-11 |
| RRP12 | 1.331066 | 1.35E-18 | 5.47E-18 |
| ANGPT1 | 1.547875 | 3.95E-08 | 6.58E-08 |
| PPP6R1 | 1.296676 | 2.56E-22 | 1.87E-21 |
| AZIN2 | 1.157653 | 9.96E-10 | 1.85E-09 |
| FAM129A | 1.319218 | 0.000151 | 0.0002 |
| CA4 | 3.758304 | 5.34E-07 | 8.31E-07 |
| SCN4A | 2.689077 | 4.04E-21 | 2.37E-20 |
| TAP2 | 1.246924 | 8.17E-16 | 2.44E-15 |
| ANKRD39 | 1.259746 | 3.72E-26 | 9.50E-25 |
| SLC29A4 | 2.208179 | 0.015569 | 0.017909 |
| TMEM246 | 1.787194 | 0.001898 | 0.002337 |
| CPSF1 | 1.488821 | 5.96E-26 | 1.36E-24 |
| HAUS4 | 1.167219 | 2.98E-12 | 6.59E-12 |
| MAP3K12 | 1.171387 | 9.40E-12 | 2.00E-11 |
| VPS33B | 1.029589 | 1.18E-24 | 1.62E-23 |
| CCDC66 | 1.256375 | 1.08E-19 | 5.05E-19 |
| ECSCR | 1.2184 | 2.05E-14 | 5.41E-14 |
| AC011448.1 | 1.120771 | 5.09E-17 | 1.73E-16 |
| QPCTL | 1.376359 | 3.55E-21 | 2.12E-20 |
| GSDME | 1.419194 | 0.000548 | 0.000698 |
| ZNF133 | 1.32069 | 4.28E-23 | 3.73E-22 |
| AKIP1 | 1.43672 | 1.78E-27 | 1.09E-25 |
| ZNF675 | 1.67867 | 6.18E-13 | 1.44E-12 |
| SRRM3 | 4.324713 | 1.09E-09 | 2.02E-09 |
| PRPF40B | 1.466173 | 1.37E-19 | 6.33E-19 |
| ZNF300 | 2.823022 | 5.24E-14 | 1.34E-13 |
| ISG20L2 | 1.250603 | 1.85E-24 | 2.37E-23 |
| PELP1 | 1.027739 | 5.19E-20 | 2.54E-19 |
| PADI3 | 8.540083 | 4.41E-07 | 6.89E-07 |
| VPS11 | 1.23633 | 2.80E-24 | 3.37E-23 |
| TIPRL | 1.125283 | 1.54E-25 | 2.91E-24 |
| WSB2 | 1.062413 | 8.24E-21 | 4.58E-20 |
| PITX1 | 6.527362 | 3.23E-15 | 9.12E-15 |
| DDA1 | 1.105688 | 3.61E-25 | 5.88E-24 |
| ADAR | 1.026746 | 4.15E-18 | 1.59E-17 |
| CACNA1D | 1.335015 | 7.31E-05 | 9.86E-05 |
| PLD6 | 1.098993 | 6.28E-12 | 1.35E-11 |
| EIF2D | 1.35414 | 5.05E-28 | 4.88E-26 |
| MYH3 | 2.13798 | 8.23E-05 | 0.000111 |
| CSKMT | 1.734356 | 3.03E-23 | 2.77E-22 |
| THSD1 | 1.003905 | 0.00164 | 0.002028 |
| HIST1H3H | 2.68834 | 4.86E-15 | 1.35E-14 |
| RHPN1 | 2.57295 | 2.00E-21 | 1.24E-20 |
| AVPR1A | -1.81508 | 2.83E-15 | 8.02E-15 |
| BRD3OS | 1.089317 | 5.48E-11 | 1.11E-10 |
| DDX20 | 1.031135 | 3.33E-20 | 1.68E-19 |
| KXD1 | 1.112169 | 9.42E-28 | 7.21E-26 |
| ZNF57 | 1.853113 | 3.72E-19 | 1.61E-18 |
| PALB2 | 1.110715 | 7.91E-18 | 2.95E-17 |
| ZNF189 | 1.246761 | 2.65E-14 | 6.93E-14 |
| TSPO2 | 2.808003 | 1.83E-15 | 5.31E-15 |
| ZNF865 | 1.380571 | 3.52E-25 | 5.77E-24 |
| SNRPE | 1.824194 | 6.62E-28 | 5.68E-26 |
| TPM4 | 1.29412 | 1.79E-09 | 3.27E-09 |
| KISS1 | 2.344371 | 1.14E-13 | 2.82E-13 |
| ITPR3 | 2.214343 | 0.036548 | 0.041046 |
| COL7A1 | 3.151283 | 7.40E-19 | 3.09E-18 |
| SLC22A4 | 2.520216 | 1.47E-16 | 4.74E-16 |
| CYP1B1 | 2.152829 | 0.006929 | 0.008179 |
| CCSAP | 1.741703 | 3.85E-19 | 1.67E-18 |
| GDI1 | 1.18684 | 2.72E-25 | 4.71E-24 |
| GSN | 1.021652 | 8.66E-10 | 1.62E-09 |
| HEATR1 | 1.603913 | 9.64E-19 | 3.97E-18 |
| AC012651.1 | 1.313049 | 1.40E-06 | 2.12E-06 |
| ALDH3B1 | 1.273663 | 1.40E-06 | 2.12E-06 |
| BMP5 | -1.72627 | 2.06E-21 | 1.28E-20 |
| NAT2 | -2.40044 | 1.20E-25 | 2.39E-24 |
| REEP4 | 1.509316 | 1.50E-20 | 8.04E-20 |
| DZIP3 | 1.298579 | 6.85E-19 | 2.87E-18 |
| SMAD3 | 1.093508 | 8.62E-13 | 1.99E-12 |
| KCND3 | -1.33663 | 1.78E-18 | 7.08E-18 |
| FXYD1 | -1.30864 | 2.02E-16 | 6.43E-16 |
| SLC45A2 | 2.466657 | 8.10E-05 | 0.000109 |
| FRMPD1 | 1.951042 | 0.028059 | 0.031751 |
| METTL23 | 1.101345 | 1.32E-23 | 1.33E-22 |
| CDH19 | -1.26539 | 1.65E-17 | 5.95E-17 |
| LTA | 1.275401 | 1.78E-05 | 2.51E-05 |
| BRAT1 | 1.15893 | 1.42E-25 | 2.74E-24 |
| ZNF404 | 1.622683 | 7.61E-07 | 1.17E-06 |
| ALKBH4 | 1.049126 | 7.30E-25 | 1.07E-23 |
| ZBTB7B | 1.40972 | 7.16E-21 | 4.04E-20 |
| KCNF1 | 2.85802 | 8.27E-08 | 1.35E-07 |
| JRK | 2.430051 | 2.62E-27 | 1.39E-25 |
| RTL8A | 1.268357 | 1.37E-11 | 2.88E-11 |
| RDM1 | 4.164067 | 1.70E-23 | 1.67E-22 |
| WRAP53 | 1.058402 | 1.00E-17 | 3.69E-17 |
| ZNF70 | 1.271815 | 2.15E-09 | 3.90E-09 |
| ZNF668 | 1.440186 | 1.86E-19 | 8.42E-19 |
| OAZ3 | 1.533371 | 2.78E-21 | 1.68E-20 |
| PRR14L | 1.001786 | 2.13E-15 | 6.11E-15 |
| TMC6 | 1.455187 | 0.005794 | 0.006881 |
| AC023509.3 | 1.658966 | 3.53E-18 | 1.36E-17 |
| MOB3A | 1.054091 | 9.11E-17 | 3.02E-16 |
| TOMM34 | 1.055296 | 2.09E-21 | 1.30E-20 |
| SMC2 | 1.648753 | 1.48E-18 | 5.93E-18 |
| SKAP1 | -1.00392 | 2.21E-15 | 6.34E-15 |
| SFRP2 | 2.64587 | 0.030322 | 0.034242 |
| CBSL | -1.10308 | 0.000395 | 0.000507 |
| TTC1 | 1.004515 | 1.39E-25 | 2.68E-24 |
| NEK11 | 1.012147 | 0.005192 | 0.006189 |
| NELFCD | 1.082635 | 8.09E-25 | 1.16E-23 |
| PLEKHG6 | 1.298507 | 0.000416 | 0.000534 |
| CCZ1 | 1.049613 | 1.00E-13 | 2.49E-13 |
| TRPS1 | 1.28278 | 5.13E-05 | 7.00E-05 |
| PBX2 | 1.304329 | 3.68E-21 | 2.18E-20 |
| DNAJC18 | 1.107191 | 5.56E-10 | 1.05E-09 |
| EHMT2 | 2.01215 | 4.23E-28 | 4.56E-26 |
| NEMP1 | 1.271188 | 3.93E-12 | 8.62E-12 |
| MCF2L | 1.417648 | 4.46E-16 | 1.37E-15 |
| LRRC59 | 1.093251 | 1.95E-21 | 1.22E-20 |
| CYSTM1 | 1.38886 | 5.33E-22 | 3.69E-21 |
| ZNF577 | 1.219236 | 0.000432 | 0.000553 |
| SEC31B | 1.190232 | 7.30E-11 | 1.47E-10 |
| CNPY4 | 1.147844 | 2.10E-16 | 6.68E-16 |
| CBR3 | 2.587101 | 8.94E-16 | 2.66E-15 |
| SIGLEC7 | -1.36301 | 2.89E-17 | 1.01E-16 |
| ADNP | 1.111343 | 1.74E-19 | 7.91E-19 |
| SSR1 | 1.12684 | 7.75E-24 | 8.28E-23 |
| RHOF | 2.084305 | 1.43E-11 | 3.00E-11 |
| XRCC6 | 1.001809 | 9.42E-25 | 1.33E-23 |
| PFKFB2 | 1.894455 | 6.30E-20 | 3.04E-19 |
| ZBTB40 | 1.609197 | 5.54E-23 | 4.72E-22 |
| MFSD10 | 1.829438 | 4.83E-18 | 1.84E-17 |
| UGT1A6 | 1.292981 | 0.00698 | 0.008235 |
| ULK4 | 1.702535 | 5.48E-11 | 1.11E-10 |
| PPP2R1A | 1.127281 | 1.92E-24 | 2.45E-23 |
| CD70 | 2.420479 | 1.61E-05 | 2.27E-05 |
| SMARCA4 | 1.640492 | 4.92E-27 | 2.12E-25 |
| PAG1 | 1.012263 | 0.001037 | 0.001298 |
| VPS54 | 1.094647 | 1.03E-19 | 4.80E-19 |
| GPX2 | 1.323232 | 0.010098 | 0.011768 |
| SYNM | 1.496376 | 6.50E-10 | 1.22E-09 |
| KHDC4 | 2.0192 | 6.68E-27 | 2.52E-25 |
| ADGRL1 | 2.44061 | 1.22E-10 | 2.41E-10 |
| ATP6V1F | 1.372962 | 4.43E-25 | 6.99E-24 |
| CD248 | 1.791458 | 1.36E-15 | 3.98E-15 |
| ADAMTS13 | -2.18903 | 5.33E-28 | 5.01E-26 |
| PAMR1 | -1.14309 | 7.82E-22 | 5.27E-21 |
| KNTC1 | 2.687834 | 1.34E-23 | 1.35E-22 |
| ZNF581 | 1.84771 | 4.41E-26 | 1.08E-24 |
| WDR76 | 2.959637 | 1.89E-24 | 2.43E-23 |
| SERPINE2 | 1.964509 | 0.004075 | 0.004901 |
| CAMK2N2 | 3.062848 | 1.80E-12 | 4.06E-12 |
| SPAG5 | 2.091993 | 1.28E-19 | 5.93E-19 |
| TET3 | 1.348231 | 2.65E-14 | 6.93E-14 |
| MRPS23 | 1.452648 | 1.14E-27 | 7.96E-26 |
| FGD6 | 1.320977 | 1.05E-07 | 1.71E-07 |
| CERK | 1.020487 | 1.28E-14 | 3.43E-14 |
| GTF2H4 | 1.354983 | 3.52E-20 | 1.77E-19 |
| BLM | 3.167263 | 2.11E-22 | 1.57E-21 |
| CKAP4 | 1.701548 | 3.27E-21 | 1.96E-20 |
| ZNF287 | 1.210997 | 0.012638 | 0.014614 |
| SLC41A1 | 1.713818 | 1.26E-10 | 2.49E-10 |
| NFATC4 | 2.011999 | 1.21E-12 | 2.76E-12 |
| CCNQ | 1.144918 | 6.26E-23 | 5.25E-22 |
| MED27 | 1.180148 | 3.88E-23 | 3.41E-22 |
| RBCK1 | 1.440661 | 2.63E-24 | 3.19E-23 |
| IHH | 1.955993 | 1.24E-06 | 1.88E-06 |
| PDGFB | 1.568964 | 1.84E-18 | 7.31E-18 |
| VPS33A | 1.396316 | 2.39E-27 | 1.33E-25 |
| CARMIL1 | 1.113398 | 0.000362 | 0.000466 |
| METTL1 | 1.178247 | 4.50E-19 | 1.93E-18 |
| RPLP0 | 1.203841 | 7.81E-20 | 3.72E-19 |
| AIFM3 | 1.950905 | 2.82E-12 | 6.27E-12 |
| MAP2K6 | 1.206142 | 0.00015 | 0.000198 |
| SP6 | 3.816407 | 2.44E-24 | 3.00E-23 |
| ARPC1A | 1.216961 | 2.48E-26 | 6.93E-25 |
| RAD54L | 4.173081 | 1.82E-25 | 3.38E-24 |
| ARG2 | 2.019573 | 7.62E-05 | 0.000103 |
| RPL7 | 1.362595 | 1.47E-16 | 4.74E-16 |
| RAD51D | 1.265745 | 1.89E-20 | 9.92E-20 |
| MAF1 | 1.354775 | 7.94E-24 | 8.47E-23 |
| TPGS2 | 1.471635 | 4.30E-19 | 1.85E-18 |
| SLC7A5 | 1.032477 | 0.026349 | 0.029868 |
| PCSK9 | 1.522558 | 6.16E-10 | 1.16E-09 |
| PPP1R14C | 5.25238 | 6.58E-05 | 8.90E-05 |
| ITGA3 | 2.096898 | 0.000448 | 0.000573 |
| LYZ | 2.742507 | 2.09E-07 | 3.34E-07 |
| BBS4 | 1.223625 | 7.19E-22 | 4.87E-21 |
| CNFN | 2.609345 | 2.78E-09 | 5.00E-09 |
| TFPT | 1.152119 | 7.30E-20 | 3.49E-19 |
| POLR3G | 1.184477 | 3.97E-13 | 9.40E-13 |
| C1orf112 | 1.620135 | 7.68E-15 | 2.09E-14 |
| DENR | 1.017454 | 3.43E-23 | 3.08E-22 |
| PCOLCE2 | 1.126896 | 0.04318 | 0.048183 |
| DNM1L | 1.133129 | 3.29E-20 | 1.66E-19 |
| LSMEM1 | 1.331675 | 2.77E-12 | 6.16E-12 |
| ZFAT | 1.367977 | 7.19E-24 | 7.80E-23 |
| GBA2 | 1.160355 | 9.46E-24 | 9.91E-23 |
| CYP26A1 | -3.31785 | 1.48E-21 | 9.42E-21 |
| MMP14 | 2.12724 | 7.85E-16 | 2.35E-15 |
| ADRA1D | 2.847325 | 2.16E-05 | 3.02E-05 |
| ELOVL3 | 3.198615 | 1.64E-06 | 2.46E-06 |
| HIST1H4J | 1.083409 | 8.69E-05 | 0.000117 |
| EIF3G | 1.026533 | 2.56E-21 | 1.56E-20 |
| SLX4IP | 1.040395 | 1.60E-18 | 6.38E-18 |
| MYH4 | 2.148133 | 0.000814 | 0.001028 |
| PDLIM4 | 1.073095 | 0.029405 | 0.03322 |
| ADAM15 | 1.984843 | 1.42E-26 | 4.50E-25 |
| EPS15L1 | 1.365717 | 1.32E-25 | 2.58E-24 |
| IRAK1 | 1.946477 | 6.88E-26 | 1.52E-24 |
| CAV1 | 1.046894 | 3.25E-09 | 5.81E-09 |
| CD24 | 2.74087 | 1.14E-06 | 1.74E-06 |
| ADAMTS16 | 4.457925 | 1.68E-05 | 2.36E-05 |
| NOX4 | 3.487096 | 1.94E-28 | 3.44E-26 |
| TBC1D20 | 1.046554 | 7.21E-25 | 1.07E-23 |
| ABCA7 | 1.589683 | 8.68E-16 | 2.59E-15 |
| NECAB3 | 2.087398 | 9.77E-29 | 2.81E-26 |
| PLK1 | 4.342699 | 1.55E-27 | 9.94E-26 |
| KPNA2 | 2.142876 | 3.99E-24 | 4.58E-23 |
| KIFAP3 | 1.099045 | 3.73E-20 | 1.86E-19 |
| JADE2 | 1.364637 | 6.45E-18 | 2.44E-17 |
| UROC1 | -1.77166 | 3.22E-19 | 1.41E-18 |
| INTS3 | 1.389898 | 4.17E-17 | 1.43E-16 |
| ANKHD1-EIF4EBP3 | 1.074384 | 8.17E-18 | 3.04E-17 |
| HNRNPA1 | 1.270128 | 1.94E-23 | 1.88E-22 |
| RPUSD1 | 1.347394 | 1.28E-24 | 1.73E-23 |
| RPL37A | 1.100312 | 6.15E-14 | 1.56E-13 |
| GPR137C | 1.944809 | 2.74E-16 | 8.61E-16 |
| ZIC4 | 4.09535 | 3.82E-05 | 5.25E-05 |
| SH3GLB2 | 1.275387 | 1.19E-22 | 9.47E-22 |
| PPIC | 1.004786 | 9.03E-16 | 2.68E-15 |
| CKMT1B | 5.366102 | 0.004189 | 0.005031 |
| TCF7 | 1.538831 | 1.20E-06 | 1.83E-06 |
| APLP1 | 3.787078 | 3.46E-07 | 5.44E-07 |
| YEATS2 | 1.767461 | 1.50E-23 | 1.49E-22 |
| WASF1 | 1.407866 | 3.00E-12 | 6.65E-12 |
| BOLA3 | 1.431738 | 5.96E-26 | 1.36E-24 |
| GON4L | 1.410378 | 1.02E-21 | 6.66E-21 |
| MLEC | 1.323551 | 1.03E-22 | 8.27E-22 |
| RIBC2 | 3.702249 | 3.00E-17 | 1.05E-16 |
| NUDT17 | 2.529904 | 1.80E-25 | 3.35E-24 |
| CHCHD6 | 1.040419 | 5.99E-18 | 2.26E-17 |
| MGAT3 | 2.281821 | 4.56E-12 | 9.93E-12 |
| TRIM6 | 1.979409 | 8.94E-08 | 1.46E-07 |
| PRSS35 | 1.200443 | 0.022605 | 0.02574 |
| AMH | 3.293923 | 6.50E-08 | 1.07E-07 |
| STRIP2 | 3.427093 | 4.87E-21 | 2.83E-20 |
| SLC35F2 | 1.849814 | 0.006083 | 0.007212 |
| PAX8 | 1.981161 | 2.00E-13 | 4.86E-13 |
| KLHL22 | 1.052271 | 1.14E-20 | 6.21E-20 |
| LARP1 | 1.326871 | 1.82E-24 | 2.35E-23 |
| FBXL8 | 1.164005 | 5.26E-15 | 1.45E-14 |
| OSGEPL1 | 1.117562 | 4.05E-22 | 2.87E-21 |
| UQCRH | 1.245501 | 2.01E-22 | 1.51E-21 |
| MEOX1 | 2.193116 | 2.07E-06 | 3.09E-06 |
| GSDMC | 4.817161 | 8.32E-19 | 3.46E-18 |
| LRP5L | 1.219648 | 1.59E-13 | 3.90E-13 |
| SLC27A3 | 1.063648 | 2.00E-06 | 2.99E-06 |
| C9orf116 | 1.272512 | 3.06E-07 | 4.83E-07 |
| BLMH | 1.264362 | 5.60E-06 | 8.12E-06 |
| DCST2 | 2.765325 | 4.30E-22 | 3.03E-21 |
| CPSF7 | 1.008305 | 1.70E-19 | 7.75E-19 |
| ADH1A | -1.23568 | 5.65E-17 | 1.91E-16 |
| PPP1R13L | 1.581416 | 7.64E-17 | 2.55E-16 |
| CAPN10 | 1.499404 | 4.96E-26 | 1.19E-24 |
| FBLN7 | 1.562589 | 1.74E-12 | 3.92E-12 |
| TIAM2 | 1.475282 | 1.24E-10 | 2.45E-10 |
| RBMX2 | 1.097505 | 9.03E-23 | 7.32E-22 |
| SPEF2 | 1.696139 | 1.95E-15 | 5.62E-15 |
| CROCC | 1.562622 | 4.25E-22 | 3.00E-21 |
| KRT10 | 1.099961 | 2.63E-16 | 8.28E-16 |
| PSKH1 | 1.404673 | 3.77E-22 | 2.68E-21 |
| PDGFRL | 2.365731 | 8.68E-16 | 2.59E-15 |
| PON2 | 1.15829 | 2.85E-16 | 8.94E-16 |
| ZNF845 | 1.072842 | 2.27E-08 | 3.85E-08 |
| RAPGEFL1 | 1.536149 | 1.91E-09 | 3.47E-09 |
| L3MBTL1 | 2.114336 | 3.56E-20 | 1.78E-19 |
| CENPN | 1.362548 | 5.93E-19 | 2.50E-18 |
| RPL8 | 1.735396 | 4.54E-21 | 2.65E-20 |
| RHBDF1 | 1.450761 | 1.31E-15 | 3.83E-15 |
| TERT | 9.483907 | 5.48E-26 | 1.29E-24 |
| ETS2 | -1.05782 | 5.52E-16 | 1.68E-15 |
| LRRC46 | 1.439118 | 8.93E-17 | 2.96E-16 |
| TALDO1 | 1.137989 | 2.81E-18 | 1.09E-17 |
| SHCBP1 | 4.083711 | 1.24E-25 | 2.45E-24 |
| ZNF346 | 1.143406 | 7.39E-25 | 1.08E-23 |
| UNK | 1.391064 | 3.26E-24 | 3.84E-23 |
| ZNF710 | 1.325099 | 1.19E-10 | 2.36E-10 |
| MCM3 | 2.265828 | 4.41E-26 | 1.08E-24 |
| ASF1B | 3.712236 | 2.03E-26 | 5.96E-25 |
| TOM1L2 | 1.05483 | 5.52E-16 | 1.68E-15 |
| POLR2K | 1.54035 | 2.97E-28 | 3.94E-26 |
| COMMD3 | 1.055923 | 2.35E-24 | 2.91E-23 |
| MADCAM1 | 1.092141 | 5.93E-08 | 9.79E-08 |
| TATDN3 | 1.131034 | 3.26E-23 | 2.95E-22 |
| EXOSC1 | 1.062839 | 3.99E-24 | 4.58E-23 |
| TMC5 | 3.73823 | 0.001146 | 0.00143 |
| ATAD5 | 2.400079 | 2.75E-23 | 2.54E-22 |
| PYCR2 | 1.504727 | 5.48E-28 | 5.11E-26 |
| RNPS1 | 1.00342 | 3.16E-21 | 1.90E-20 |
| SHROOM1 | 1.011217 | 6.35E-13 | 1.48E-12 |
| RNF32 | 1.11397 | 4.04E-15 | 1.13E-14 |
| CNOT11 | 1.250275 | 8.09E-25 | 1.16E-23 |
| ZNF354A | 1.493853 | 1.57E-13 | 3.86E-13 |
| HIST1H2BD | 1.068049 | 1.88E-05 | 2.63E-05 |
| TRIM37 | 1.23004 | 5.37E-19 | 2.28E-18 |
| RNF34 | 1.098682 | 1.78E-19 | 8.07E-19 |
| TMEM145 | 4.702609 | 1.00E-19 | 4.69E-19 |
| MREG | 1.027033 | 4.49E-11 | 9.16E-11 |
| RGS12 | 1.192718 | 1.10E-20 | 6.01E-20 |
| ZNF786 | 1.043328 | 1.00E-19 | 4.69E-19 |
| GIPC1 | 1.391559 | 5.59E-22 | 3.86E-21 |
| NUCB2 | 1.138133 | 5.06E-15 | 1.40E-14 |
| CASTOR2 | 2.291739 | 9.85E-20 | 4.62E-19 |
| RMI2 | 2.624478 | 7.30E-20 | 3.49E-19 |
| IDH3G | 1.055229 | 3.11E-23 | 2.83E-22 |
| LYVE1 | -2.26605 | 2.23E-23 | 2.11E-22 |
| RHBDL1 | 2.314805 | 9.80E-20 | 4.60E-19 |
| CORO1B | 1.296647 | 1.06E-24 | 1.47E-23 |
| CCDC170 | 3.058822 | 4.31E-13 | 1.02E-12 |
| ACVRL1 | 1.321845 | 2.58E-18 | 1.01E-17 |
| TPD52 | 1.087526 | 4.15E-13 | 9.82E-13 |
| TCF20 | 1.123481 | 3.42E-17 | 1.19E-16 |
| DUT | 1.415465 | 3.56E-24 | 4.16E-23 |
| ZNF503 | 1.372324 | 8.54E-06 | 1.22E-05 |
| TRIM32 | 1.120785 | 2.20E-17 | 7.80E-17 |
| LRP11 | 1.333189 | 2.24E-17 | 7.96E-17 |
| CIP2A | 2.970179 | 5.21E-22 | 3.61E-21 |
| AP1S1 | 1.222209 | 4.10E-25 | 6.53E-24 |
| GPD2 | 1.214134 | 1.94E-12 | 4.34E-12 |
| TAF1A | 1.649994 | 6.00E-19 | 2.53E-18 |
| ZDHHC24 | 1.152046 | 7.43E-23 | 6.10E-22 |
| PHPT1 | 1.836608 | 3.87E-26 | 9.76E-25 |
| ZPR1 | 1.204453 | 3.79E-24 | 4.40E-23 |
| VIM | 1.039764 | 1.08E-08 | 1.88E-08 |
| CFAP44 | 1.671827 | 1.17E-15 | 3.45E-15 |
| RPRD2 | 1.087206 | 9.50E-17 | 3.14E-16 |
| MARS | 1.314327 | 1.39E-25 | 2.68E-24 |
| NUP133 | 1.245518 | 1.63E-24 | 2.13E-23 |
| CLCNKA | 3.413261 | 3.60E-11 | 7.38E-11 |
| OXCT1 | 2.440371 | 0.000229 | 0.000299 |
| FAM174B | 1.050441 | 0.004012 | 0.004828 |
| GUK1 | 1.116927 | 8.60E-22 | 5.73E-21 |
| SARM1 | 1.422752 | 2.23E-11 | 4.63E-11 |
| NOP58 | 1.090402 | 4.13E-20 | 2.04E-19 |
| SETDB1 | 1.469838 | 6.34E-25 | 9.57E-24 |
| HOXD9 | 5.23589 | 2.81E-21 | 1.70E-20 |
| ZNF467 | 1.792991 | 2.16E-10 | 4.19E-10 |
| GAL3ST4 | 1.954168 | 7.96E-08 | 1.30E-07 |
| EIF2B1 | 1.04962 | 2.69E-27 | 1.42E-25 |
| PSMB5 | 1.010406 | 5.62E-27 | 2.30E-25 |
| ENOX1 | 2.468824 | 5.46E-08 | 9.04E-08 |
| RDH13 | 1.242838 | 1.11E-16 | 3.64E-16 |
| DISC1 | 1.075189 | 0.005393 | 0.006422 |
| KCNIP3 | 2.27893 | 0.004577 | 0.005479 |
| ANKRD52 | 2.145014 | 2.90E-26 | 7.77E-25 |
| DDX49 | 1.46691 | 1.92E-28 | 3.44E-26 |
| FAM163B | -1.7478 | 3.94E-18 | 1.51E-17 |
| BCL7C | 1.138399 | 3.77E-18 | 1.45E-17 |
| CAMK2G | 1.219395 | 1.02E-22 | 8.18E-22 |
| CD36 | 1.274281 | 0.043948 | 0.049009 |
| KCNN2 | -2.83615 | 2.96E-23 | 2.71E-22 |
| TRMT2A | 1.142098 | 1.61E-24 | 2.11E-23 |
| PI15 | 4.830642 | 1.88E-12 | 4.22E-12 |
| UTP18 | 1.106024 | 4.39E-21 | 2.57E-20 |
| RPL27 | 1.22801 | 1.00E-20 | 5.51E-20 |
| APOO | 1.013683 | 6.34E-19 | 2.67E-18 |
| CYP39A1 | -1.88792 | 9.95E-23 | 8.00E-22 |
| KIF2A | 1.295587 | 2.58E-14 | 6.74E-14 |
| UCHL1 | 5.517809 | 8.24E-08 | 1.35E-07 |
| SNCG | 4.14345 | 1.76E-19 | 7.99E-19 |
| MISP3 | 1.08415 | 7.56E-07 | 1.16E-06 |
| E2F3 | 1.418712 | 2.54E-13 | 6.10E-13 |
| NBL1 | 1.890368 | 6.36E-06 | 9.19E-06 |
| FAM216A | 1.067389 | 1.20E-12 | 2.73E-12 |
| IL22RA1 | 1.132872 | 0.001141 | 0.001424 |
| MDC1 | 1.385963 | 5.56E-20 | 2.71E-19 |
| RECQL4 | 3.369344 | 6.62E-28 | 5.68E-26 |
| CDK6 | 1.042591 | 0.00698 | 0.008235 |
| CYSRT1 | 1.331811 | 2.12E-11 | 4.41E-11 |
| KLHL21 | 1.375255 | 1.55E-15 | 4.51E-15 |
| TBKBP1 | 1.709476 | 3.60E-19 | 1.57E-18 |
| TSACC | 2.211372 | 3.95E-22 | 2.81E-21 |
| BLOC1S4 | 1.068974 | 2.38E-22 | 1.75E-21 |
| RAPGEF3 | 1.392 | 1.90E-18 | 7.53E-18 |
| REXO5 | 2.083278 | 2.27E-22 | 1.67E-21 |
| GGPS1 | 1.188946 | 4.94E-24 | 5.52E-23 |
| SCUBE2 | 2.805637 | 0.010425 | 0.012138 |
| ZNF543 | 1.308467 | 6.62E-16 | 2.00E-15 |
| PRKAB2 | 1.301856 | 9.79E-16 | 2.90E-15 |
| DUSP9 | 5.799062 | 3.22E-23 | 2.92E-22 |
| CRYBA2 | 6.540216 | 1.85E-06 | 2.77E-06 |
| MFAP3L | -1.27333 | 2.06E-16 | 6.55E-16 |
| ZNF691 | 1.101507 | 9.60E-23 | 7.73E-22 |
| PKDCC | 1.999502 | 2.84E-11 | 5.86E-11 |
| CD34 | 2.697881 | 9.90E-29 | 2.81E-26 |
| LYPD3 | 1.625615 | 4.75E-09 | 8.41E-09 |
| OXTR | 2.22937 | 1.42E-07 | 2.29E-07 |
| FAM98C | 1.049662 | 9.79E-25 | 1.38E-23 |
| RUFY2 | 1.056752 | 1.54E-16 | 4.98E-16 |
| INO80E | 1.346689 | 1.85E-26 | 5.52E-25 |
| LAPTM4B | 2.293502 | 1.66E-16 | 5.34E-16 |
| TRIM17 | 4.293672 | 4.65E-11 | 9.47E-11 |
| DALRD3 | 1.092008 | 2.44E-20 | 1.26E-19 |
| PRPF39 | 1.265383 | 3.64E-21 | 2.16E-20 |
| LZTS2 | 1.617683 | 2.96E-22 | 2.14E-21 |
| SIRT4 | 1.036035 | 1.18E-15 | 3.48E-15 |
| ZNF681 | 2.88198 | 1.73E-11 | 3.63E-11 |
| WTIP | 1.27153 | 0.010315 | 0.012013 |
| EGLN2 | 1.24673 | 1.96E-20 | 1.03E-19 |
| SH2D3C | 1.077526 | 1.92E-14 | 5.08E-14 |
| FOSB | -2.6829 | 2.27E-22 | 1.67E-21 |
| PAQR8 | 2.002137 | 2.94E-08 | 4.95E-08 |
| GTPBP2 | 1.652066 | 1.94E-22 | 1.46E-21 |
| GTF3C6 | 1.066869 | 5.74E-15 | 1.58E-14 |
| RIT1 | 1.334312 | 1.09E-21 | 7.12E-21 |
| ACSL1 | -1.21723 | 8.80E-18 | 3.27E-17 |
| CNIH2 | 2.374526 | 3.68E-17 | 1.27E-16 |
| LRRC37A3 | 2.056886 | 1.18E-20 | 6.42E-20 |
| FSTL4 | 5.185102 | 1.96E-19 | 8.81E-19 |
| PAK1 | 1.264339 | 2.19E-15 | 6.28E-15 |
| INHA | 5.42 | 4.72E-07 | 7.36E-07 |
| HIST1H4I | 1.646756 | 1.78E-17 | 6.38E-17 |
| ZC3H10 | 1.100708 | 5.10E-25 | 7.89E-24 |
| NTF3 | -2.86819 | 1.89E-27 | 1.14E-25 |
| BMF | 1.62736 | 5.80E-06 | 8.40E-06 |
| LRRC56 | 1.193093 | 2.74E-07 | 4.34E-07 |
| NSMF | 1.172221 | 9.47E-13 | 2.18E-12 |
| ANKRD9 | 1.43486 | 2.11E-17 | 7.50E-17 |
| FPR1 | -1.17497 | 2.27E-12 | 5.06E-12 |
| FAM20B | 1.300468 | 1.20E-25 | 2.39E-24 |
| ANXA5 | 1.065779 | 6.91E-09 | 1.21E-08 |
| SLC38A1 | 1.758287 | 3.00E-05 | 4.16E-05 |
| PDCD5 | 1.465467 | 8.05E-27 | 2.92E-25 |
| TBX10 | 1.554831 | 5.63E-09 | 9.93E-09 |
| UQCRB | 1.280688 | 2.62E-21 | 1.59E-20 |
| SLC52A2 | 2.085582 | 1.60E-25 | 3.03E-24 |
| NAP1L1 | 1.298062 | 1.69E-18 | 6.72E-18 |
| AL136454.1 | 1.522297 | 2.57E-15 | 7.30E-15 |
| TRMT61A | 1.265806 | 2.85E-23 | 2.63E-22 |
| ZBTB17 | 1.051057 | 7.65E-24 | 8.20E-23 |
| SVBP | 1.028302 | 2.52E-12 | 5.61E-12 |
| ANGEL2 | 1.110303 | 9.92E-21 | 5.45E-20 |
| OR2B6 | 3.014873 | 2.38E-15 | 6.79E-15 |
| ZNF260 | 1.06491 | 1.91E-16 | 6.13E-16 |
| PVALB | -2.89979 | 8.21E-27 | 2.96E-25 |
| VSIG1 | 4.757723 | 1.94E-08 | 3.31E-08 |
| ATXN7L2 | 1.641193 | 5.59E-24 | 6.22E-23 |
| CDK7 | 1.195768 | 4.38E-25 | 6.93E-24 |
| CLDND2 | 1.600902 | 2.65E-10 | 5.12E-10 |
| SLC39A10 | 1.969385 | 2.81E-18 | 1.09E-17 |
| KLHL29 | 1.537895 | 7.46E-07 | 1.15E-06 |
| MANEAL | 1.54649 | 6.21E-15 | 1.70E-14 |
| ZNF696 | 1.861364 | 8.01E-28 | 6.40E-26 |
| MCRS1 | 1.44843 | 4.29E-28 | 4.56E-26 |
| RBM19 | 1.188198 | 1.69E-25 | 3.17E-24 |
| SUSD2 | 1.24536 | 0.0238 | 0.027057 |
| PCDHB14 | 1.461632 | 0.000494 | 0.00063 |
| PWWP2B | 1.682015 | 1.60E-14 | 4.25E-14 |
| ALMS1 | 1.204334 | 1.32E-18 | 5.36E-18 |
| SGO2 | 3.171216 | 3.77E-26 | 9.59E-25 |
| FAT1 | 1.467379 | 1.02E-10 | 2.03E-10 |
| PLXNC1 | 2.441815 | 1.58E-15 | 4.60E-15 |
| EDA2R | 1.708993 | 0.000211 | 0.000275 |
| STOX1 | 2.576413 | 6.27E-07 | 9.71E-07 |
| WDR45B | 1.03611 | 4.09E-21 | 2.40E-20 |
| DYNLL1 | 1.284686 | 7.78E-25 | 1.12E-23 |
| TAOK2 | 1.367983 | 2.35E-26 | 6.64E-25 |
| SENP1 | 1.142601 | 7.50E-18 | 2.81E-17 |
| THBS3 | 1.508656 | 5.02E-22 | 3.49E-21 |
| RPL27A | 1.056642 | 2.65E-14 | 6.93E-14 |
| PRELID2 | 1.383032 | 3.85E-10 | 7.34E-10 |
| NTM | 3.681491 | 2.28E-07 | 3.63E-07 |
| ZNF701 | 1.443726 | 5.82E-05 | 7.90E-05 |
| PIAS3 | 1.74262 | 9.92E-22 | 6.52E-21 |
| BPIFA2 | 8.006012 | 8.24E-09 | 1.44E-08 |
| ANXA2R | 2.117059 | 2.41E-21 | 1.48E-20 |
| SLC35E4 | 2.125595 | 3.36E-20 | 1.69E-19 |
| CS | 1.361233 | 1.45E-22 | 1.13E-21 |
| AURKB | 4.298318 | 8.80E-28 | 6.91E-26 |
| ZNF34 | 1.280895 | 6.18E-24 | 6.80E-23 |
| MOK | 1.115399 | 1.46E-12 | 3.31E-12 |
| PABPC1L | 2.360838 | 2.17E-21 | 1.34E-20 |
| ATXN2L | 1.290798 | 1.36E-20 | 7.30E-20 |
| ZFP14 | 1.317926 | 1.89E-16 | 6.07E-16 |
| PJA1 | 1.079687 | 2.86E-14 | 7.46E-14 |
| SRXN1 | 2.040729 | 5.06E-15 | 1.40E-14 |
| VPS28 | 1.535494 | 8.95E-25 | 1.27E-23 |
| EPHA2 | -1.41554 | 2.54E-13 | 6.10E-13 |
| TNFRSF4 | 2.877453 | 6.79E-26 | 1.51E-24 |
| LCAT | -2.01752 | 2.17E-26 | 6.31E-25 |
| RAB34 | 2.111647 | 0.000739 | 0.000935 |
| ARHGEF9 | 1.01299 | 1.33E-17 | 4.86E-17 |
| NUSAP1 | 3.018903 | 6.03E-25 | 9.16E-24 |
| SKA2 | 1.144153 | 1.67E-18 | 6.65E-18 |
| FRZB | 1.799797 | 2.47E-10 | 4.78E-10 |
| CUZD1 | 4.678604 | 7.37E-16 | 2.22E-15 |
| SMARCD3 | 2.047094 | 2.97E-10 | 5.70E-10 |
| DNAJB6 | 1.168527 | 2.68E-26 | 7.29E-25 |
| MLANA | 3.905126 | 0.002592 | 0.003165 |
| FABP4 | 3.262002 | 4.89E-09 | 8.66E-09 |
| SPRN | 1.392243 | 4.24E-18 | 1.63E-17 |
| TOP2A | 4.707048 | 1.85E-27 | 1.13E-25 |
| 9-Mar | 1.106446 | 3.48E-08 | 5.82E-08 |
| TMEM104 | 1.552886 | 9.56E-27 | 3.31E-25 |
| IGSF3 | 3.298042 | 8.68E-16 | 2.59E-15 |
| HIST1H4D | 2.528714 | 7.79E-12 | 1.67E-11 |
| HNRNPH1 | 1.169444 | 2.13E-19 | 9.55E-19 |
| FAM198B | 1.005036 | 1.68E-07 | 2.70E-07 |
| TMEM67 | 1.289457 | 1.73E-09 | 3.15E-09 |
| NCAPG2 | 1.997485 | 9.35E-22 | 6.17E-21 |
| TSC22D4 | 1.114854 | 6.74E-17 | 2.26E-16 |
| ATP7A | 1.013774 | 2.02E-09 | 3.68E-09 |
| RPL10 | 1.036397 | 3.76E-19 | 1.63E-18 |
| PSMC4 | 1.293546 | 1.27E-28 | 3.14E-26 |
| TRIM55 | 1.136368 | 0.00641 | 0.007586 |
| TNRC18 | 1.328112 | 1.75E-20 | 9.22E-20 |
| SLC35G2 | 1.325753 | 4.54E-06 | 6.62E-06 |
| NPLOC4 | 1.52489 | 2.42E-28 | 3.60E-26 |
| LAMA5 | 1.765113 | 7.85E-14 | 1.97E-13 |
| EME1 | 4.019325 | 1.04E-27 | 7.57E-26 |
| P2RY8 | 1.411982 | 9.25E-11 | 1.84E-10 |
| GDPD3 | 2.234847 | 1.36E-17 | 4.96E-17 |
| HSP90AA1 | 1.057758 | 6.55E-19 | 2.75E-18 |
| GALR3 | 1.958779 | 1.44E-05 | 2.04E-05 |
| ECEL1 | 6.787726 | 0.000109 | 0.000145 |
| NIPAL1 | -1.00981 | 9.60E-13 | 2.20E-12 |
| RAP1GAP | 1.485443 | 0.000357 | 0.00046 |
| TOMM20 | 1.308038 | 2.55E-25 | 4.44E-24 |
| CDC37 | 1.152096 | 9.68E-28 | 7.28E-26 |
| SRC | 1.862442 | 2.16E-10 | 4.19E-10 |
| CACNA2D3 | 2.704262 | 0.000145 | 0.000191 |
| GIPR | 2.752406 | 0.00343 | 0.004151 |
| RPS12 | 1.097963 | 4.84E-12 | 1.05E-11 |
| ZNF205 | 1.335457 | 5.65E-25 | 8.66E-24 |
| ATP6V1FNB | 3.603419 | 4.30E-22 | 3.03E-21 |
| BAK1 | 1.615915 | 1.55E-17 | 5.59E-17 |
| STK39 | 2.708237 | 6.27E-14 | 1.59E-13 |
| FKBP11 | 1.509843 | 2.57E-17 | 9.07E-17 |
| CENPJ | 1.965902 | 1.03E-23 | 1.07E-22 |
| SOX9 | 1.970541 | 2.70E-06 | 4.01E-06 |
| NUP205 | 1.335224 | 5.02E-19 | 2.14E-18 |
| NELFE | 2.010097 | 5.54E-27 | 2.28E-25 |
| TRMT13 | 1.026096 | 2.21E-15 | 6.34E-15 |
| CDPF1 | 1.478744 | 4.09E-24 | 4.68E-23 |
| RABIF | 1.531789 | 3.31E-28 | 4.02E-26 |
| PPARG | 1.208227 | 6.56E-11 | 1.32E-10 |
| NR4A3 | -1.65012 | 2.23E-13 | 5.40E-13 |
| FIGNL1 | 1.826981 | 4.04E-20 | 2.00E-19 |
| NTPCR | 1.371365 | 1.38E-24 | 1.84E-23 |
| ZNF706 | 1.459234 | 2.09E-26 | 6.10E-25 |
| LY6G5B | 2.081078 | 1.18E-15 | 3.48E-15 |
| GJA5 | 2.173413 | 5.86E-16 | 1.78E-15 |
| UPK1A | 3.655263 | 4.11E-08 | 6.85E-08 |
| CCNE2 | 2.874546 | 1.61E-21 | 1.02E-20 |
| QPCT | 2.033988 | 3.43E-05 | 4.74E-05 |
| CA12 | 3.518653 | 1.48E-09 | 2.72E-09 |
| RCC1 | 1.301267 | 2.63E-19 | 1.17E-18 |
| KIF20A | 4.524544 | 2.80E-27 | 1.45E-25 |
| CKAP2 | 1.951206 | 1.22E-20 | 6.62E-20 |
| RPS11 | 1.057242 | 2.20E-17 | 7.80E-17 |
| PDZK1 | 1.403763 | 2.74E-16 | 8.61E-16 |
| KRT20 | 7.687861 | 1.81E-09 | 3.31E-09 |
| SUPT5H | 1.074232 | 6.42E-27 | 2.47E-25 |
| UQCC2 | 1.613284 | 2.41E-26 | 6.79E-25 |
| ZBTB18 | 1.178474 | 1.65E-13 | 4.04E-13 |
| CDKN2C | 3.018498 | 3.01E-28 | 3.95E-26 |
| PTGFRN | 1.434433 | 7.15E-14 | 1.80E-13 |
| PDCL | 1.053679 | 8.92E-19 | 3.69E-18 |
| AGER | 1.25595 | 1.99E-15 | 5.72E-15 |
| RPL19 | 1.026825 | 5.37E-20 | 2.62E-19 |
| LZTR1 | 1.154099 | 1.48E-25 | 2.83E-24 |
| CNIH3 | 1.523451 | 2.40E-13 | 5.79E-13 |
| CHTOP | 1.253914 | 2.48E-27 | 1.35E-25 |
| PBK | 4.174713 | 1.71E-26 | 5.20E-25 |
| VGF | 2.784247 | 5.53E-08 | 9.14E-08 |
| KIF24 | 2.650458 | 9.70E-24 | 1.01E-22 |
| GAS2L1 | 1.136582 | 3.13E-18 | 1.21E-17 |
| FDCSP | 6.205037 | 1.35E-07 | 2.18E-07 |
| AGPAT4 | 2.069272 | 9.51E-10 | 1.77E-09 |
| PPAT | 1.0314 | 3.59E-13 | 8.53E-13 |
| MAGEA3 | 7.968757 | 7.71E-05 | 0.000104 |
| CDHR2 | -1.31647 | 1.26E-13 | 3.11E-13 |
| SPATA24 | 1.31905 | 2.47E-18 | 9.67E-18 |
| EIF4ENIF1 | 1.053802 | 4.54E-21 | 2.65E-20 |
| AL157935.2 | 1.265505 | 1.40E-07 | 2.26E-07 |
| ERCC2 | 1.232972 | 6.75E-24 | 7.37E-23 |
| AGO2 | 1.711959 | 3.15E-23 | 2.87E-22 |
| LCN2 | 5.190369 | 1.91E-10 | 3.73E-10 |
| SYT9 | -1.80542 | 1.07E-20 | 5.86E-20 |
| NCSTN | 1.12762 | 3.18E-20 | 1.61E-19 |
| TTC39A | 3.963237 | 1.76E-17 | 6.32E-17 |
| XK | 1.708564 | 0.001674 | 0.002069 |
| SLC25A33 | 1.070467 | 1.63E-10 | 3.19E-10 |
| DEDD | 1.260469 | 1.55E-27 | 9.94E-26 |
| UFSP1 | 1.216125 | 2.95E-15 | 8.34E-15 |
| ATP2A2 | 1.041329 | 2.33E-16 | 7.37E-16 |
| C19orf44 | 1.177753 | 7.59E-21 | 4.25E-20 |
| PA2G4 | 1.133702 | 1.85E-25 | 3.42E-24 |
| TIGD5 | 2.236842 | 4.59E-28 | 4.65E-26 |
| TRPM4 | 1.053285 | 2.85E-10 | 5.48E-10 |
| MPV17 | 1.601078 | 4.79E-25 | 7.47E-24 |
| C5orf63 | 1.110814 | 2.48E-08 | 4.20E-08 |
| SLC25A14 | 1.189282 | 3.68E-21 | 2.18E-20 |
| FOS | -2.41925 | 6.68E-21 | 3.78E-20 |
| POLD1 | 1.997186 | 1.56E-26 | 4.82E-25 |
| CUEDC1 | 1.748379 | 3.81E-21 | 2.25E-20 |
| EVA1B | 1.174327 | 2.53E-11 | 5.24E-11 |
| SERPINH1 | 1.835011 | 1.75E-20 | 9.22E-20 |
| MLH3 | 1.163717 | 5.48E-21 | 3.15E-20 |
| ACADS | -1.1693 | 9.35E-22 | 6.17E-21 |
| ATP6AP1 | 1.420662 | 4.59E-28 | 4.65E-26 |
| SH3PXD2B | 2.04149 | 1.80E-24 | 2.33E-23 |
| KAT7 | 1.05674 | 9.80E-20 | 4.60E-19 |
| CEP72 | 2.065387 | 1.83E-23 | 1.78E-22 |
| ZNF787 | 1.042439 | 6.44E-20 | 3.11E-19 |
| DVL3 | 1.368568 | 2.63E-24 | 3.19E-23 |
| COA6 | 1.893904 | 8.51E-29 | 2.81E-26 |
| ANKRD54 | 1.174415 | 1.06E-26 | 3.59E-25 |
| SPOCK1 | 5.167744 | 7.46E-15 | 2.03E-14 |
| NOP56 | 1.559976 | 2.07E-23 | 1.98E-22 |
| ZFP36 | -1.52034 | 8.00E-18 | 2.98E-17 |
| ASS1 | -1.20928 | 1.11E-18 | 4.54E-18 |
| FANCG | 2.184508 | 8.95E-27 | 3.15E-25 |
| LAIR1 | 1.096301 | 5.79E-05 | 7.86E-05 |
| SYNE4 | 1.412715 | 4.61E-09 | 8.18E-09 |
| DBN1 | 2.497636 | 1.73E-15 | 5.01E-15 |
| WNT6 | 3.748051 | 4.69E-11 | 9.55E-11 |
| MROH6 | 1.945622 | 7.67E-11 | 1.54E-10 |
| ARHGEF25 | 1.050066 | 0.02771 | 0.031369 |
| DCUN1D5 | 1.030516 | 1.93E-17 | 6.91E-17 |
| MAEL | 4.828399 | 1.26E-05 | 1.78E-05 |
| TBC1D13 | 1.515419 | 1.11E-23 | 1.14E-22 |
| NLRC5 | 1.191499 | 2.85E-10 | 5.48E-10 |
| CACYBP | 1.748309 | 6.00E-27 | 2.39E-25 |
| AGTRAP | 1.194661 | 3.06E-16 | 9.57E-16 |
| HAUS1 | 1.38924 | 1.79E-21 | 1.13E-20 |
| TMEM168 | 1.137207 | 3.84E-17 | 1.32E-16 |
| CENPM | 4.24869 | 1.90E-26 | 5.63E-25 |
| MEP1A | 7.264253 | 1.20E-09 | 2.22E-09 |
| LCMT1 | 1.323937 | 5.58E-25 | 8.57E-24 |
| FAM184A | 1.132421 | 4.41E-05 | 6.04E-05 |
| TXNRD1 | 2.055145 | 1.93E-17 | 6.91E-17 |
| DUSP18 | 1.271893 | 5.21E-15 | 1.44E-14 |
| WDR62 | 3.312991 | 1.21E-26 | 4.03E-25 |
| CDK5RAP1 | 1.115711 | 1.72E-28 | 3.39E-26 |
| FHL5 | 1.993176 | 5.34E-06 | 7.75E-06 |
| ZNF579 | 1.528973 | 2.95E-19 | 1.30E-18 |
| TYMS | 2.51029 | 1.44E-20 | 7.71E-20 |
| EHD2 | 1.424772 | 2.77E-12 | 6.16E-12 |
| FAM78B | 1.894567 | 2.80E-17 | 9.83E-17 |
| ETV4 | 3.602327 | 2.20E-09 | 3.98E-09 |
| ING5 | 1.270812 | 2.46E-23 | 2.30E-22 |
| TNFRSF25 | 2.149708 | 3.26E-22 | 2.34E-21 |
| OXLD1 | 1.635159 | 1.04E-26 | 3.52E-25 |
| IPO4 | 1.248045 | 1.45E-19 | 6.68E-19 |
| STC2 | 2.8251 | 1.14E-18 | 4.63E-18 |
| ACHE | 1.437664 | 0.032861 | 0.037021 |
| ARIH2OS | 1.674513 | 1.82E-24 | 2.35E-23 |
| INPP5F | 1.07383 | 3.66E-13 | 8.68E-13 |
| RSPH9 | 1.106255 | 9.80E-20 | 4.60E-19 |
| SCNN1D | 1.301566 | 5.83E-10 | 1.10E-09 |
| PCLAF | 3.220231 | 7.06E-26 | 1.56E-24 |
| TUBA1C | 1.256844 | 1.64E-12 | 3.70E-12 |
| SOWAHD | 1.235638 | 3.78E-05 | 5.20E-05 |
| C9orf40 | 1.408566 | 2.55E-19 | 1.13E-18 |
| HSD17B13 | -1.66059 | 8.46E-20 | 4.01E-19 |
| TSPO | 1.123652 | 7.10E-06 | 1.02E-05 |
| RFX3 | 1.075914 | 4.88E-11 | 9.91E-11 |
| ASPSCR1 | 1.849182 | 9.13E-22 | 6.04E-21 |
| CSF2RA | 1.002201 | 0.010499 | 0.012217 |
| VXN | 1.014633 | 0.005859 | 0.006955 |
| ZNF35 | 1.093839 | 1.41E-10 | 2.77E-10 |
| DHX16 | 1.099086 | 2.60E-24 | 3.17E-23 |
| CAD | 1.9938 | 5.58E-26 | 1.30E-24 |
| HP | -1.55074 | 6.60E-21 | 3.74E-20 |
| ASPDH | -1.07267 | 7.62E-16 | 2.28E-15 |
| VPS13B | 1.196807 | 3.19E-16 | 9.95E-16 |
| LRRC75A | 1.362773 | 2.92E-07 | 4.62E-07 |
| TRIM3 | 1.223756 | 1.24E-22 | 9.79E-22 |
| EFNB3 | -1.13618 | 6.51E-20 | 3.14E-19 |
| ZNF718 | 1.178844 | 3.89E-09 | 6.92E-09 |
| PLA2G7 | 1.214959 | 0.000584 | 0.000743 |
| LIMK2 | 1.658448 | 8.59E-16 | 2.56E-15 |
| OCRL | 1.529868 | 1.85E-24 | 2.37E-23 |
| SWT1 | 1.100351 | 3.37E-19 | 1.47E-18 |
| CBX1 | 1.585812 | 1.47E-24 | 1.95E-23 |
| VWA7 | 1.611893 | 2.59E-10 | 5.00E-10 |
| ATOH8 | -1.09184 | 1.26E-13 | 3.11E-13 |
| C1QL4 | 5.11791 | 6.76E-13 | 1.57E-12 |
| PRMT3 | 1.475738 | 1.80E-22 | 1.37E-21 |
| SPECC1 | 2.262943 | 3.80E-12 | 8.34E-12 |
| ARFIP2 | 1.057015 | 3.19E-23 | 2.89E-22 |
| C7orf31 | 1.333517 | 2.92E-05 | 4.05E-05 |
| RRP9 | 1.408405 | 2.60E-24 | 3.17E-23 |
| HMGA2 | 6.883956 | 0.000626 | 0.000794 |
| CNOT9 | 1.071382 | 7.73E-20 | 3.68E-19 |
| CYCS | 1.005218 | 1.71E-15 | 4.96E-15 |
| POLA2 | 1.961333 | 8.15E-26 | 1.73E-24 |
| NKD1 | 4.530712 | 0.001647 | 0.002036 |
| RAD52 | 1.118449 | 2.04E-16 | 6.49E-16 |
| LRRC37A2 | 1.269956 | 2.03E-11 | 4.24E-11 |
| APOD | 1.826481 | 3.76E-05 | 5.17E-05 |
| GTSE1 | 4.340927 | 9.95E-27 | 3.41E-25 |
| COMMD5 | 1.54882 | 3.08E-27 | 1.54E-25 |
| CDC42EP4 | 1.016433 | 1.62E-16 | 5.24E-16 |
| PARP15 | 1.000011 | 0.004303 | 0.005164 |
| UBE2M | 1.273238 | 2.35E-26 | 6.64E-25 |
| ZNF544 | 1.53791 | 5.05E-21 | 2.92E-20 |
| OTUB2 | 2.191475 | 2.23E-23 | 2.11E-22 |
| SRM | 1.414872 | 2.34E-23 | 2.20E-22 |
| RIBC1 | 1.10733 | 4.22E-12 | 9.22E-12 |
| CCNF | 2.930903 | 1.57E-27 | 1.00E-25 |
| ARPC3 | 1.092887 | 1.58E-22 | 1.22E-21 |
| ARNTL2 | 1.185235 | 9.94E-05 | 0.000133 |
| QRICH2 | 2.145908 | 2.71E-21 | 1.65E-20 |
| ZNF81 | 1.009033 | 3.06E-10 | 5.87E-10 |
| C9 | -2.14152 | 2.02E-23 | 1.94E-22 |
| CPSF3 | 1.175408 | 5.70E-29 | 2.53E-26 |
| ZNF497 | 1.139723 | 8.08E-19 | 3.36E-18 |
| TNNC1 | 3.714481 | 1.47E-08 | 2.52E-08 |
| PCNX3 | 1.611074 | 5.54E-27 | 2.28E-25 |
| ADCK5 | 1.585731 | 2.98E-25 | 5.08E-24 |
| LTB4R | 1.197559 | 4.00E-10 | 7.63E-10 |
| CLIP2 | 1.970965 | 5.24E-08 | 8.68E-08 |
| KNOP1 | 1.446191 | 6.28E-26 | 1.41E-24 |
| NFYA | 1.330314 | 5.49E-19 | 2.32E-18 |
| NANOS1 | 3.386158 | 2.30E-16 | 7.30E-16 |
| PALD1 | 1.435285 | 2.28E-13 | 5.49E-13 |
| EGR1 | -1.94885 | 4.40E-19 | 1.89E-18 |
| PLA2G6 | 1.98384 | 5.46E-24 | 6.07E-23 |
| CERS5 | 1.288529 | 3.19E-23 | 2.89E-22 |
| RRAGB | 1.108853 | 2.49E-17 | 8.81E-17 |
| DBH | -2.57965 | 1.78E-24 | 2.30E-23 |
| DUSP28 | 1.355204 | 3.90E-28 | 4.48E-26 |
| RAB24 | 1.700564 | 8.37E-27 | 2.98E-25 |
| FBRSL1 | 1.07787 | 2.26E-24 | 2.82E-23 |
| ACYP1 | 1.822796 | 4.19E-26 | 1.04E-24 |
| GBGT1 | 1.16174 | 4.78E-06 | 6.97E-06 |
| KIF3C | 1.475215 | 0.006015 | 0.007135 |
| NCOA5 | 1.053759 | 6.41E-19 | 2.69E-18 |
| CPSF4 | 1.500701 | 1.49E-27 | 9.64E-26 |
| RPL35 | 1.011796 | 1.26E-10 | 2.49E-10 |
| LHX3 | 4.298842 | 0.005066 | 0.006044 |
| LSM11 | 1.48544 | 4.67E-22 | 3.27E-21 |
| GPAA1 | 2.021866 | 1.40E-28 | 3.25E-26 |
| HOPX | 1.835516 | 9.18E-09 | 1.60E-08 |
| MRPL24 | 1.401484 | 5.67E-24 | 6.28E-23 |
| PLSCR4 | -1.22122 | 3.48E-20 | 1.75E-19 |
| ZMYND8 | 1.083686 | 4.33E-16 | 1.33E-15 |
| WNT5A | 1.404518 | 0.022607 | 0.02574 |
| ZNF280C | 1.34313 | 5.98E-16 | 1.81E-15 |
| SLC16A3 | 2.397205 | 1.33E-09 | 2.45E-09 |
| HYOU1 | 1.220661 | 4.77E-15 | 1.32E-14 |
| RALY | 1.418719 | 6.59E-27 | 2.52E-25 |
| ADORA2B | 1.414773 | 0.040703 | 0.045537 |
| PLCD3 | 2.202052 | 8.51E-16 | 2.54E-15 |
| NRF1 | 1.019899 | 3.77E-22 | 2.68E-21 |
| FOXM1 | 4.385308 | 4.85E-27 | 2.09E-25 |
| XPO5 | 1.636147 | 6.09E-27 | 2.40E-25 |
| GORAB | 1.397668 | 2.21E-14 | 5.82E-14 |
| GJA1 | 1.494318 | 1.75E-10 | 3.42E-10 |
| STK19 | 1.356637 | 2.98E-24 | 3.58E-23 |
| NISCH | 1.152872 | 1.27E-25 | 2.51E-24 |
| JAM3 | 1.137914 | 1.73E-12 | 3.89E-12 |
| TNFRSF11A | 1.526322 | 0.000297 | 0.000384 |
| INMT | -1.94474 | 7.33E-21 | 4.12E-20 |
| TMEM150B | 2.90462 | 1.71E-16 | 5.50E-16 |
| MYCN | 4.375771 | 4.27E-15 | 1.19E-14 |
| INTS7 | 1.278098 | 2.50E-22 | 1.83E-21 |
| RETREG1 | -1.681 | 1.21E-18 | 4.93E-18 |
| CCDC84 | 1.384043 | 2.36E-19 | 1.05E-18 |
| MYO19 | 1.904055 | 4.84E-26 | 1.16E-24 |
| TBC1D12 | 1.015427 | 2.01E-14 | 5.31E-14 |
| PPP1R3E | 1.188713 | 1.04E-18 | 4.27E-18 |
| RANBP3L | -1.6364 | 1.82E-17 | 6.51E-17 |
| ROBO3 | 1.503188 | 2.58E-09 | 4.64E-09 |
| RBM3 | 1.592823 | 6.73E-23 | 5.60E-22 |
| TBX19 | 1.658986 | 3.86E-20 | 1.92E-19 |
| CENPP | 1.7902 | 7.39E-25 | 1.08E-23 |
| CCNE1 | 4.716404 | 1.67E-26 | 5.10E-25 |
| EHBP1L1 | 1.063707 | 8.78E-14 | 2.19E-13 |
| TNFRSF21 | 1.332744 | 0.000119 | 0.000158 |
| ZBTB12 | 2.688932 | 1.35E-22 | 1.06E-21 |
| ZFP82 | 1.647241 | 1.17E-05 | 1.67E-05 |
| RTL10 | 1.807858 | 2.52E-27 | 1.37E-25 |
| OTX1 | 5.214417 | 8.33E-22 | 5.58E-21 |
| DEK | 1.093795 | 3.40E-13 | 8.10E-13 |
| SCAMP5 | 2.022577 | 4.22E-12 | 9.22E-12 |
| FAM171B | 1.977879 | 1.37E-11 | 2.88E-11 |
| PCGF2 | 1.630645 | 2.94E-21 | 1.78E-20 |
| ZNF440 | 1.014277 | 2.57E-15 | 7.30E-15 |
| ARFRP1 | 1.249427 | 7.78E-25 | 1.12E-23 |
| DDIT4L | 3.108145 | 3.54E-05 | 4.89E-05 |
| ZFP64 | 1.186611 | 8.81E-22 | 5.85E-21 |
| ASAP1 | 1.438208 | 1.38E-13 | 3.40E-13 |
| SAP30L | 1.063551 | 2.41E-22 | 1.77E-21 |
| PHF14 | 1.094578 | 7.33E-27 | 2.72E-25 |
| ALS2CL | 2.094646 | 9.11E-17 | 3.02E-16 |
| MSH6 | 1.090227 | 5.56E-20 | 2.71E-19 |
| DUOXA2 | 4.870331 | 0.00079 | 0.000998 |
| LRRC10B | 2.35983 | 2.77E-17 | 9.73E-17 |
| IARS | 1.31296 | 1.26E-19 | 5.81E-19 |
| NCOA6 | 1.184071 | 1.20E-18 | 4.88E-18 |
| AADAT | -2.18237 | 7.44E-26 | 1.63E-24 |
| WDR46 | 1.361813 | 4.65E-26 | 1.12E-24 |
| RNF152 | -1.17342 | 6.64E-15 | 1.82E-14 |
| MAN1C1 | -1.02589 | 8.84E-17 | 2.93E-16 |
| GSK3A | 1.046974 | 3.48E-26 | 9.04E-25 |
| CEP170 | 1.046117 | 2.35E-09 | 4.26E-09 |
| PSPH | 2.185944 | 3.94E-24 | 4.54E-23 |
| MOB3B | 1.506098 | 2.79E-06 | 4.12E-06 |
| GBA | 2.028235 | 3.64E-28 | 4.26E-26 |
| ROBO1 | 3.534811 | 7.16E-19 | 2.99E-18 |
| GNPDA1 | 1.415973 | 3.86E-20 | 1.92E-19 |
| VAC14 | 1.068784 | 2.58E-23 | 2.40E-22 |
| PCNX2 | 2.19328 | 1.90E-08 | 3.24E-08 |
| ACSL6 | 1.96238 | 0.03546 | 0.039841 |
| PPARD | 1.042313 | 1.51E-11 | 3.18E-11 |
| ZNF224 | 1.139529 | 5.65E-17 | 1.91E-16 |
| NFATC2IP | 1.070339 | 6.44E-20 | 3.11E-19 |
| CLDN19 | 3.540851 | 0.004453 | 0.005339 |
| RPL36A | 1.593735 | 2.01E-18 | 7.93E-18 |
| AARS2 | 1.473713 | 1.71E-26 | 5.20E-25 |
| CGAS | 1.212702 | 0.00179 | 0.002209 |
| ZNF256 | 1.32302 | 9.24E-07 | 1.41E-06 |
| PGP | 1.76056 | 1.37E-25 | 2.66E-24 |
| MID1 | 1.262237 | 1.63E-10 | 3.19E-10 |
| PMF1-BGLAP | 1.252232 | 2.02E-12 | 4.53E-12 |
| EFCAB2 | 1.315909 | 3.08E-19 | 1.35E-18 |
| DAPK3 | 1.058939 | 1.12E-18 | 4.59E-18 |
| MSMB | 3.204065 | 9.23E-05 | 0.000124 |
| CD302 | -1.09416 | 9.39E-18 | 3.48E-17 |
| FABP6 | 5.416595 | 4.64E-06 | 6.77E-06 |
| ZNF525 | 1.644772 | 9.79E-06 | 1.40E-05 |
| HLA-F | 1.175781 | 1.95E-08 | 3.33E-08 |
| CCDC77 | 1.582107 | 1.94E-25 | 3.56E-24 |
| KCTD6 | 1.198252 | 3.27E-14 | 8.48E-14 |
| ZNF816 | 1.243728 | 0.001097 | 0.001371 |
| CHRM3 | 2.03945 | 8.79E-07 | 1.35E-06 |
| KLK4 | 7.685089 | 0.000193 | 0.000253 |
| ZNF226 | 1.032717 | 2.81E-15 | 7.95E-15 |
| KMT5C | 2.180339 | 1.06E-27 | 7.60E-26 |
| RRNAD1 | 1.01554 | 1.12E-22 | 8.95E-22 |
| TNNT1 | 4.387871 | 0.001024 | 0.001282 |
| COL9A2 | 2.592363 | 2.31E-05 | 3.23E-05 |
| MND1 | 3.528328 | 7.63E-27 | 2.80E-25 |
| POLR2H | 1.116899 | 1.04E-22 | 8.36E-22 |
| SSH3 | 1.047948 | 4.18E-14 | 1.07E-13 |
| ATAD2 | 2.388775 | 1.92E-23 | 1.86E-22 |
| DBNL | 1.076653 | 6.42E-25 | 9.67E-24 |
| SMARCB1 | 1.142845 | 8.60E-22 | 5.73E-21 |
| TCTEX1D1 | -1.57269 | 7.77E-16 | 2.33E-15 |
| TAF4 | 1.096039 | 4.57E-18 | 1.75E-17 |
| SSRP1 | 1.014534 | 3.35E-21 | 2.00E-20 |
| TRMT6 | 1.316544 | 6.90E-23 | 5.73E-22 |
| CCDC40 | 1.666729 | 8.62E-18 | 3.20E-17 |
| AP4M1 | 1.528805 | 9.56E-27 | 3.31E-25 |
| AFF3 | 1.174809 | 0.000376 | 0.000483 |
| IK | 1.05447 | 6.09E-27 | 2.40E-25 |
| ZSWIM9 | 1.348958 | 9.28E-26 | 1.93E-24 |
| CUTA | 1.31146 | 6.58E-24 | 7.20E-23 |
| TUSC2 | 1.041257 | 1.17E-24 | 1.60E-23 |
| ZNF83 | 2.120462 | 5.22E-06 | 7.59E-06 |
| PHLDA3 | 1.913295 | 2.64E-06 | 3.92E-06 |
| GJC1 | 3.073442 | 4.53E-26 | 1.10E-24 |
| ORM1 | -1.30454 | 3.46E-14 | 8.95E-14 |
| NUP188 | 1.121341 | 3.60E-17 | 1.25E-16 |
| SNX11 | 1.021325 | 7.34E-23 | 6.04E-22 |
| FAM161A | 1.007993 | 1.50E-06 | 2.26E-06 |
| GARS | 1.252967 | 4.58E-24 | 5.18E-23 |
| GM2A | 1.161592 | 2.41E-21 | 1.48E-20 |
| KLHDC7B | 2.087341 | 7.80E-08 | 1.28E-07 |
| NEIL3 | 4.923093 | 5.92E-27 | 2.38E-25 |
| SLC51B | 2.153518 | 7.99E-15 | 2.17E-14 |
| FICD | 1.090479 | 2.13E-15 | 6.11E-15 |
| ZNF607 | 2.27984 | 9.77E-13 | 2.24E-12 |
| FAM229A | 1.686188 | 3.15E-19 | 1.38E-18 |
| TIMM13 | 1.160519 | 5.82E-20 | 2.82E-19 |
| GABRE | 3.491859 | 9.40E-17 | 3.11E-16 |
| BRCA1 | 1.502983 | 5.86E-12 | 1.27E-11 |
| RUSC1 | 2.098527 | 1.28E-26 | 4.16E-25 |
| DPM2 | 1.330919 | 2.09E-22 | 1.55E-21 |
| RRM2 | 3.764286 | 3.18E-26 | 8.43E-25 |
| EGR3 | -1.48105 | 3.08E-13 | 7.35E-13 |
| LGALS4 | 1.594596 | 0.000205 | 0.000268 |
| CCNO | 3.829686 | 2.40E-08 | 4.07E-08 |
| NBPF11 | 1.308677 | 6.30E-13 | 1.47E-12 |
| SMOC2 | 1.485986 | 4.92E-06 | 7.17E-06 |
| ZNF623 | 1.535716 | 5.14E-23 | 4.40E-22 |
| PPP1R37 | 1.454703 | 3.05E-25 | 5.18E-24 |
| PPM1G | 1.254198 | 3.04E-27 | 1.53E-25 |
| ABCA3 | 2.277927 | 1.67E-11 | 3.51E-11 |
| E2F8 | 4.399368 | 3.28E-25 | 5.47E-24 |
| BUB1B | 4.342403 | 2.75E-26 | 7.42E-25 |
| FAM50A | 1.696804 | 9.82E-24 | 1.02E-22 |
| CPD | 1.454435 | 8.39E-17 | 2.79E-16 |
| SLC5A11 | 2.988666 | 6.36E-06 | 9.19E-06 |
| DCAF15 | 1.146149 | 4.96E-22 | 3.46E-21 |
| ACTG2 | 2.539275 | 1.82E-17 | 6.51E-17 |
| NACC1 | 1.231515 | 3.74E-23 | 3.32E-22 |
| ZNF75A | 1.187771 | 6.30E-20 | 3.04E-19 |
| COL2A1 | 10.00783 | 1.66E-07 | 2.67E-07 |
| ZC3H3 | 1.78145 | 2.51E-26 | 6.93E-25 |
| BFSP1 | 2.155797 | 2.87E-20 | 1.46E-19 |
| ANKRD55 | -1.72994 | 6.27E-17 | 2.11E-16 |
| GNAO1 | -1.62539 | 4.13E-17 | 1.42E-16 |
| NPEPL1 | 1.82664 | 5.88E-26 | 1.35E-24 |
| PIK3C2B | 1.622683 | 2.26E-18 | 8.89E-18 |
| PAXIP1 | 1.175672 | 1.48E-18 | 5.93E-18 |
| STRADA | 1.263015 | 2.61E-19 | 1.16E-18 |
| PRPF3 | 1.680846 | 8.37E-27 | 2.98E-25 |
| CCDC85C | 1.213427 | 1.06E-14 | 2.84E-14 |
| RRAGD | 1.661052 | 2.74E-16 | 8.61E-16 |
| IQGAP3 | 4.043825 | 3.67E-26 | 9.43E-25 |
| CLEC12A | -1.02806 | 9.40E-12 | 2.00E-11 |
| CEACAM19 | 1.408575 | 1.20E-10 | 2.37E-10 |
| BIRC7 | 3.103673 | 0.000356 | 0.000459 |
| MRPL53 | 1.689069 | 4.29E-28 | 4.56E-26 |
| DDX27 | 1.088437 | 6.49E-23 | 5.43E-22 |
| PSMB3 | 1.052964 | 6.30E-20 | 3.04E-19 |
| UVSSA | 1.406308 | 1.30E-16 | 4.22E-16 |
| ALDH18A1 | 1.085077 | 4.28E-15 | 1.19E-14 |
| ITLN1 | -1.8395 | 1.90E-22 | 1.43E-21 |
| MKS1 | 1.094651 | 4.25E-22 | 3.00E-21 |
| SLC39A6 | 1.086104 | 3.47E-11 | 7.13E-11 |
| TLDC2 | 3.064984 | 4.65E-19 | 1.99E-18 |
| FBXL19 | 1.794927 | 1.00E-25 | 2.06E-24 |
| HIST1H2BH | 3.210985 | 6.29E-07 | 9.74E-07 |
| RAD51C | 1.346931 | 1.80E-25 | 3.35E-24 |
| RASGRF1 | 4.638908 | 1.36E-09 | 2.50E-09 |
| PSMB4 | 1.390161 | 2.52E-28 | 3.60E-26 |
| EXTL3 | 1.27218 | 4.11E-16 | 1.27E-15 |
| FGD1 | 2.086401 | 8.33E-21 | 4.63E-20 |
| ZWILCH | 1.634456 | 2.49E-23 | 2.32E-22 |
| TRIM28 | 1.405146 | 3.70E-25 | 6.00E-24 |
| CRTC2 | 1.250498 | 9.65E-26 | 2.00E-24 |
| POPDC3 | 7.217556 | 8.17E-07 | 1.25E-06 |
| SGSH | 1.128226 | 4.76E-21 | 2.77E-20 |
| MTBP | 2.59798 | 6.85E-25 | 1.02E-23 |
| HIST1H3B | 3.881509 | 5.27E-18 | 2.00E-17 |
| NUTF2 | 1.138646 | 2.71E-23 | 2.52E-22 |
| NONO | 1.142886 | 2.66E-22 | 1.93E-21 |
| TINAG | 7.589287 | 6.11E-10 | 1.15E-09 |
| SCN8A | 1.162958 | 4.35E-08 | 7.24E-08 |
| VDR | 1.55964 | 1.87E-05 | 2.62E-05 |
| ASIC3 | 2.649976 | 8.40E-22 | 5.61E-21 |
| RPS23 | 1.037921 | 2.92E-17 | 1.02E-16 |
| MYEF2 | 3.122069 | 0.001181 | 0.001473 |
| ZNF248 | 1.29256 | 1.75E-20 | 9.22E-20 |
| ZNF414 | 1.128068 | 5.40E-23 | 4.62E-22 |
| WDR54 | 1.483387 | 4.94E-11 | 1.00E-10 |
| IKBKG | 1.508548 | 9.70E-15 | 2.62E-14 |
| ZNF143 | 1.092226 | 3.69E-20 | 1.84E-19 |
| CCDC191 | 1.383141 | 1.93E-15 | 5.57E-15 |
| PGGHG | 2.26468 | 2.48E-12 | 5.52E-12 |
| ACSM5 | -1.31929 | 2.44E-17 | 8.63E-17 |
| SIN3B | 1.206171 | 3.46E-22 | 2.48E-21 |
| NAXE | 1.41149 | 7.44E-26 | 1.63E-24 |
| CHRAC1 | 1.049418 | 2.79E-19 | 1.23E-18 |
| IL3RA | 1.825694 | 3.14E-24 | 3.73E-23 |
| SSSCA1 | 1.003485 | 3.77E-20 | 1.88E-19 |
| CERS1 | 5.12231 | 2.21E-10 | 4.29E-10 |
| HOXC9 | 6.726338 | 3.78E-11 | 7.74E-11 |
| CEP128 | 1.504363 | 1.24E-13 | 3.06E-13 |
| POLR2L | 1.037674 | 3.18E-19 | 1.39E-18 |
| DAPK2 | 2.158688 | 1.19E-20 | 6.48E-20 |
| HTR1D | 4.993535 | 1.69E-07 | 2.71E-07 |
| MED10 | 1.034669 | 4.81E-19 | 2.05E-18 |
| SOX13 | 1.448185 | 5.61E-23 | 4.77E-22 |
| SAPCD2 | 3.644978 | 5.40E-22 | 3.73E-21 |
| CDC25B | 1.707773 | 1.29E-17 | 4.71E-17 |
| C1QL1 | 4.549574 | 7.00E-10 | 1.31E-09 |
| FAM110D | 1.126702 | 3.37E-10 | 6.44E-10 |
| PET100 | 1.048999 | 4.59E-15 | 1.27E-14 |
| BLZF1 | 1.204272 | 3.07E-20 | 1.56E-19 |
| TCF21 | -1.07805 | 1.62E-16 | 5.24E-16 |
| SVIL | 1.181901 | 5.85E-11 | 1.18E-10 |
| GPR158 | 4.139808 | 7.38E-11 | 1.48E-10 |
| PHLDA2 | 1.290176 | 2.47E-05 | 3.44E-05 |
| EIF4A3 | 1.064468 | 5.29E-21 | 3.06E-20 |
| EFNA4 | 2.237316 | 1.78E-26 | 5.37E-25 |
| SPP1 | 5.006114 | 2.72E-07 | 4.31E-07 |
| TFPI2 | -1.17965 | 4.26E-17 | 1.46E-16 |
| NCAPG | 4.54893 | 7.79E-28 | 6.35E-26 |
| ANKRD16 | 1.093009 | 4.71E-21 | 2.74E-20 |
| PKMYT1 | 3.88234 | 4.72E-28 | 4.74E-26 |
| SCART1 | 1.660638 | 7.70E-05 | 0.000104 |
| ANXA2 | 1.833935 | 6.77E-19 | 2.84E-18 |
| INTS4 | 1.194731 | 4.35E-26 | 1.07E-24 |
| CYP19A1 | 7.522496 | 2.10E-10 | 4.08E-10 |
| TAGLN2 | 1.52033 | 1.87E-20 | 9.82E-20 |
| TMEM101 | 1.525045 | 1.93E-26 | 5.69E-25 |
| CNPY2 | 1.137917 | 6.18E-23 | 5.20E-22 |
| S100PBP | 1.238068 | 1.68E-19 | 7.67E-19 |
| CTTN | 1.292172 | 2.55E-27 | 1.37E-25 |
| MOSPD1 | 1.318432 | 8.39E-15 | 2.28E-14 |
| FAM220A | 1.439568 | 9.31E-27 | 3.25E-25 |
| FAM222A | 2.41595 | 5.08E-22 | 3.53E-21 |
| MMP11 | 4.555641 | 2.95E-24 | 3.53E-23 |
| RADX | 1.572361 | 6.31E-08 | 1.04E-07 |
| ZNF480 | 1.218456 | 2.53E-20 | 1.30E-19 |
| SULT1C2 | 4.276251 | 5.02E-13 | 1.18E-12 |
| MCM8 | 2.122818 | 5.53E-22 | 3.82E-21 |
| TRAPPC2B | 1.036534 | 4.29E-12 | 9.37E-12 |
| ZNF610 | 2.132052 | 0.001696 | 0.002095 |
| MCM10 | 3.815023 | 7.78E-25 | 1.12E-23 |
| NARF | 1.598469 | 3.76E-27 | 1.79E-25 |
| DLX5 | 6.479687 | 3.86E-14 | 9.96E-14 |
| KMO | -1.28096 | 8.62E-18 | 3.20E-17 |
| UHRF1BP1 | 1.25261 | 7.24E-16 | 2.18E-15 |
| MT1E | -2.09429 | 1.27E-22 | 1.00E-21 |
| HSPA1L | 1.0444 | 1.89E-13 | 4.61E-13 |
| CCDC9B | 1.692891 | 2.06E-06 | 3.08E-06 |
| HOXA5 | 1.580681 | 0.029958 | 0.033833 |
| USP35 | 1.387145 | 1.48E-17 | 5.36E-17 |
| DENND5A | 1.094798 | 3.68E-16 | 1.14E-15 |
| RNF187 | 1.414451 | 2.75E-25 | 4.74E-24 |
| CNKSR2 | 1.345933 | 0.010876 | 0.012635 |
| STPG1 | 1.250258 | 3.95E-16 | 1.22E-15 |
| C11orf80 | 1.931448 | 9.12E-19 | 3.77E-18 |
| MMS19 | 1.233996 | 8.09E-25 | 1.16E-23 |
| LAMB3 | 1.812654 | 0.000113 | 0.000151 |
| CCL8 | 1.007602 | 0.00037 | 0.000477 |
| GATAD1 | 1.13109 | 3.23E-21 | 1.94E-20 |
| GLDN | 4.516438 | 7.31E-10 | 1.37E-09 |
| FBXO31 | 1.000479 | 6.04E-05 | 8.18E-05 |
| SLC39A14 | -1.00625 | 4.26E-17 | 1.46E-16 |
| FITM1 | -1.5117 | 1.39E-20 | 7.46E-20 |
| MAP1B | 1.770843 | 1.65E-13 | 4.04E-13 |
| GPATCH2 | 1.16092 | 2.58E-18 | 1.01E-17 |
| BTN2A2 | 1.367307 | 6.08E-17 | 2.05E-16 |
| RHBDL3 | 4.977021 | 3.97E-09 | 7.07E-09 |
| EEF1AKNMT | 1.194477 | 2.49E-23 | 2.32E-22 |
| PPIL1 | 1.278045 | 8.91E-22 | 5.91E-21 |
| SLC36A1 | 1.721937 | 3.69E-20 | 1.84E-19 |
| NDOR1 | 1.718777 | 1.73E-24 | 2.25E-23 |
| PLAC8 | -1.52456 | 4.54E-21 | 2.65E-20 |
| CENPH | 2.574034 | 1.30E-26 | 4.19E-25 |
| DTX1 | -1.16413 | 3.16E-13 | 7.55E-13 |
| EXOC7 | 1.062995 | 9.26E-20 | 4.37E-19 |
| ZNF253 | 1.050952 | 3.85E-10 | 7.34E-10 |
| CANX | 1.102288 | 2.00E-21 | 1.24E-20 |
| ZNF385D | 4.423497 | 2.47E-08 | 4.18E-08 |
| SMURF2 | 1.052863 | 1.76E-11 | 3.69E-11 |
| REG4 | 2.952185 | 0.000106 | 0.000142 |
| SSUH2 | 4.479283 | 2.30E-13 | 5.54E-13 |
| LYSMD1 | 1.480065 | 1.28E-25 | 2.52E-24 |
| PSMC3IP | 2.380437 | 2.17E-23 | 2.07E-22 |
| JARID2 | 1.102662 | 6.82E-16 | 2.06E-15 |
| RTN1 | 1.183462 | 0.003219 | 0.003905 |
| SUZ12 | 1.02331 | 5.91E-15 | 1.63E-14 |
| DRAM1 | 1.200034 | 1.11E-11 | 2.36E-11 |
| UNC5B | 1.460875 | 7.51E-07 | 1.16E-06 |
| TTC13 | 2.076169 | 8.39E-29 | 2.81E-26 |
| HNRNPU | 1.003868 | 6.99E-23 | 5.79E-22 |
| TFRC | 1.578697 | 1.93E-16 | 6.18E-16 |
| APBB1 | 1.226366 | 5.76E-14 | 1.46E-13 |
| CPQ | 1.205184 | 5.05E-21 | 2.92E-20 |
| AGAP2 | 1.282612 | 7.93E-16 | 2.37E-15 |
| TDO2 | -1.25916 | 2.05E-15 | 5.89E-15 |
| CYP2B6 | -2.11705 | 1.14E-20 | 6.21E-20 |
| ZKSCAN3 | 2.104112 | 1.99E-24 | 2.53E-23 |
| MAP2 | 2.133864 | 6.30E-10 | 1.18E-09 |
| FOXK2 | 1.172618 | 5.02E-19 | 2.14E-18 |
| BARD1 | 2.039055 | 2.17E-17 | 7.72E-17 |
| ARHGEF11 | 1.833675 | 5.61E-23 | 4.77E-22 |
| TMEM183A | 1.060655 | 2.20E-24 | 2.76E-23 |
| LINC00672 | 2.024933 | 4.22E-17 | 1.45E-16 |
| MSRB3 | 1.036538 | 0.005393 | 0.006422 |
| KIAA1586 | 1.163643 | 9.64E-19 | 3.97E-18 |
| POP5 | 1.118446 | 3.47E-23 | 3.11E-22 |
| NUDCD1 | 1.531028 | 7.80E-23 | 6.39E-22 |
| ATP1B1 | 1.411997 | 1.00E-12 | 2.30E-12 |
| DESI2 | 1.420237 | 3.56E-23 | 3.18E-22 |
| CNOT10 | 1.060643 | 1.76E-23 | 1.72E-22 |
| KATNB1 | 1.271165 | 2.12E-23 | 2.02E-22 |
| PDCD6 | 1.146659 | 8.01E-28 | 6.40E-26 |
| ATOX1 | 1.223424 | 2.85E-16 | 8.94E-16 |
| THOC1 | 1.113881 | 1.09E-20 | 5.95E-20 |
| GOSR2 | 1.120369 | 3.01E-25 | 5.13E-24 |
| TBC1D22B | 1.426665 | 2.06E-22 | 1.54E-21 |
| HCFC1 | 1.374623 | 4.46E-24 | 5.07E-23 |
| SPRYD3 | 1.099699 | 1.52E-23 | 1.51E-22 |
| ECT2 | 3.212066 | 1.23E-24 | 1.67E-23 |
| CLK2 | 1.736391 | 2.88E-27 | 1.48E-25 |
| PLXDC1 | 3.738734 | 1.95E-27 | 1.15E-25 |
| GPR107 | 1.33508 | 1.02E-22 | 8.18E-22 |
| RPP30 | 1.019436 | 1.76E-23 | 1.72E-22 |
| PRR15 | 2.301555 | 0.014749 | 0.016984 |
| CCDC24 | 1.67232 | 9.96E-19 | 4.10E-18 |
| SPDL1 | 2.552661 | 5.78E-28 | 5.17E-26 |
| MZF1 | 1.178833 | 3.68E-19 | 1.60E-18 |
| SLC4A2 | 1.331784 | 4.21E-19 | 1.81E-18 |
| PIR | 1.564094 | 2.09E-15 | 6.00E-15 |
| ADRA2B | -1.64105 | 3.00E-20 | 1.53E-19 |
| KIF26B | 2.541032 | 0.039511 | 0.044246 |
| TCAP | 2.521882 | 2.37E-15 | 6.77E-15 |
| TXNL4A | 1.161574 | 1.02E-24 | 1.42E-23 |
| SLC37A1 | 1.028209 | 1.08E-07 | 1.75E-07 |
| ZNF671 | 1.044381 | 3.43E-06 | 5.05E-06 |
| RBM42 | 1.225917 | 1.02E-24 | 1.42E-23 |
| TRIM59 | 2.707877 | 4.00E-22 | 2.84E-21 |
| TRIOBP | 1.221459 | 8.14E-21 | 4.53E-20 |
| TMEM81 | 1.701281 | 7.84E-27 | 2.85E-25 |
| MYO9B | 1.165831 | 9.36E-23 | 7.56E-22 |
| PODXL | 2.245678 | 1.93E-21 | 1.20E-20 |
| HPX | -1.22743 | 1.21E-21 | 7.86E-21 |
| RAD51 | 3.083487 | 3.17E-25 | 5.33E-24 |
| RARS | 1.019065 | 8.24E-24 | 8.76E-23 |
| AFAP1 | 1.425072 | 6.62E-07 | 1.02E-06 |
| TM4SF18 | 1.00733 | 2.12E-09 | 3.84E-09 |
| OR2I1P | 2.61794 | 1.36E-13 | 3.34E-13 |
| INS-IGF2 | -3.65141 | 5.02E-30 | 1.08E-26 |
| NPM3 | 1.66092 | 1.78E-15 | 5.16E-15 |
| RAD51AP1 | 3.007256 | 3.78E-23 | 3.34E-22 |
| NAE1 | 1.010159 | 5.67E-23 | 4.83E-22 |
| PDCD1 | 2.1504 | 0.01699 | 0.019493 |
| MMP28 | 1.638734 | 0.00302 | 0.00367 |
| PIP4K2B | 1.24235 | 1.56E-23 | 1.54E-22 |
| RB1CC1 | 1.014202 | 4.73E-14 | 1.21E-13 |
| ZG16 | -1.20343 | 7.61E-15 | 2.07E-14 |
| ZNF621 | 1.114132 | 5.30E-16 | 1.62E-15 |
| KMT2B | 1.38468 | 5.94E-22 | 4.08E-21 |
| ARHGEF39 | 3.665193 | 1.96E-29 | 1.44E-26 |
| TMEM54 | 1.773691 | 3.35E-06 | 4.94E-06 |
| PAK4 | 1.139853 | 1.88E-18 | 7.45E-18 |
| FREM2 | -1.55813 | 5.48E-18 | 2.08E-17 |
| MMP1 | 3.564235 | 2.20E-09 | 3.99E-09 |
| CDYL | 1.134476 | 4.35E-19 | 1.87E-18 |
| HEXB | 1.03684 | 3.86E-21 | 2.27E-20 |
| MAGEE1 | 1.701997 | 3.11E-09 | 5.57E-09 |
| PRRC2B | 1.063295 | 2.99E-10 | 5.74E-10 |
| IL34 | 1.415326 | 5.27E-05 | 7.18E-05 |
| KHNYN | 1.215418 | 1.02E-14 | 2.74E-14 |
| RPN2 | 1.132893 | 3.35E-26 | 8.78E-25 |
| MYMX | 3.938952 | 2.94E-13 | 7.03E-13 |
| IQANK1 | 2.367794 | 7.31E-10 | 1.37E-09 |
| JMJD4 | 1.569515 | 5.33E-27 | 2.23E-25 |
| IGHMBP2 | 1.353488 | 7.91E-22 | 5.33E-21 |
| FAM47E | 1.149577 | 2.04E-10 | 3.97E-10 |
| HMMR | 3.997017 | 4.54E-27 | 1.99E-25 |
| ELK4 | 1.015572 | 5.00E-14 | 1.28E-13 |
| LRP12 | 1.837933 | 6.96E-07 | 1.07E-06 |
| ADGRE1 | -1.28138 | 1.39E-15 | 4.06E-15 |
| ULBP2 | 2.041193 | 0.001139 | 0.001421 |
| LFNG | 1.26488 | 9.84E-07 | 1.50E-06 |
| GPATCH4 | 1.486541 | 4.04E-24 | 4.63E-23 |
| C15orf40 | 1.109425 | 2.41E-24 | 2.97E-23 |
| INTS8 | 1.669618 | 4.13E-27 | 1.88E-25 |
| AIFM2 | 1.433725 | 4.82E-21 | 2.80E-20 |
| ZNF763 | 1.020041 | 6.49E-07 | 1.00E-06 |
| ZNF30 | 1.300598 | 1.97E-21 | 1.23E-20 |
| ILF2 | 1.640823 | 1.08E-27 | 7.67E-26 |
| ARHGEF15 | 1.17402 | 1.13E-14 | 3.03E-14 |
| ZNF84 | 1.151031 | 7.91E-18 | 2.95E-17 |
| MSI1 | 4.105341 | 6.66E-07 | 1.03E-06 |
| FLNC | 4.011974 | 0.00021 | 0.000275 |
| RUNDC1 | 1.013165 | 3.40E-19 | 1.48E-18 |
| FAM45A | 1.051798 | 1.08E-16 | 3.54E-16 |
| AK8 | 2.543035 | 4.68E-05 | 6.40E-05 |
| TMEM200B | 1.246664 | 0.000922 | 0.001159 |
| ASNS | 2.360284 | 2.43E-09 | 4.38E-09 |
| THEM6 | 1.316627 | 5.49E-19 | 2.32E-18 |
| GHRHR | 4.203929 | 6.61E-06 | 9.54E-06 |
| HAPLN3 | 2.035022 | 4.64E-11 | 9.46E-11 |
| COPS9 | 1.263663 | 1.73E-20 | 9.13E-20 |
| SUSD4 | 2.133533 | 0.019785 | 0.022601 |
| ZNF322 | 1.036147 | 3.04E-10 | 5.83E-10 |
| SBF1 | 1.706838 | 7.54E-22 | 5.10E-21 |
| IL17D | 3.841742 | 9.47E-13 | 2.18E-12 |
| SNCAIP | 2.271849 | 5.41E-06 | 7.85E-06 |
| MCM4 | 2.356149 | 7.59E-25 | 1.11E-23 |
| HOXB9 | 4.728023 | 1.29E-10 | 2.54E-10 |
| NEK2 | 4.710136 | 2.77E-27 | 1.45E-25 |
| TSC1 | 1.567288 | 2.41E-24 | 2.97E-23 |
| RPL18A | 1.202517 | 2.28E-15 | 6.52E-15 |
| RCAN3 | 1.28708 | 2.70E-08 | 4.56E-08 |
| CDT1 | 4.113853 | 3.87E-27 | 1.82E-25 |
| LILRA2 | -1.32677 | 4.37E-15 | 1.22E-14 |
| GRINA | 1.096543 | 3.45E-18 | 1.33E-17 |
| NEB | 3.638108 | 2.43E-12 | 5.43E-12 |
| TAZ | 1.679503 | 7.52E-29 | 2.81E-26 |
| SCGN | 2.877996 | 0.004187 | 0.00503 |
| TMIE | 2.437506 | 2.77E-07 | 4.39E-07 |
| NIPA1 | 1.106876 | 5.48E-11 | 1.11E-10 |
| SCML2 | 2.073651 | 1.51E-18 | 6.06E-18 |
| CDK5RAP2 | 1.266637 | 2.02E-23 | 1.94E-22 |
| HLA-DMA | 1.330973 | 4.11E-05 | 5.64E-05 |
| FKBP10 | 2.050628 | 0.002776 | 0.003383 |
| ITGAV | 1.428586 | 7.54E-09 | 1.32E-08 |
| IGFALS | -2.46963 | 3.38E-22 | 2.43E-21 |
| PSMB9 | 1.144777 | 1.87E-07 | 2.99E-07 |
| C6orf62 | 1.090869 | 6.74E-20 | 3.24E-19 |
| SLC44A3 | 1.695242 | 1.72E-10 | 3.37E-10 |
| C12orf73 | 1.477332 | 5.63E-28 | 5.13E-26 |
| TUBD1 | 1.083619 | 1.11E-18 | 4.54E-18 |
| GMFB | 1.023249 | 1.61E-16 | 5.19E-16 |
| CAPRIN1 | 1.009895 | 1.14E-18 | 4.63E-18 |
| VPREB3 | 1.112812 | 0.006245 | 0.007398 |
| OTUD6B | 1.434519 | 3.81E-21 | 2.25E-20 |
| POGZ | 1.516732 | 5.67E-23 | 4.83E-22 |
| YBX2 | 2.700391 | 0.00817 | 0.009592 |
| SWAP70 | 1.244149 | 1.36E-16 | 4.43E-16 |
| UFC1 | 1.422084 | 7.38E-28 | 6.13E-26 |
| DAP3 | 1.359548 | 3.79E-28 | 4.40E-26 |
| AP002990.1 | 1.276462 | 8.91E-22 | 5.91E-21 |
| SMURF1 | 1.096027 | 1.74E-17 | 6.26E-17 |
| BUB3 | 1.153722 | 8.04E-26 | 1.72E-24 |
| S100A13 | 1.060436 | 4.62E-05 | 6.32E-05 |
| GRPEL2 | 1.320823 | 1.71E-26 | 5.20E-25 |
| ACP4 | 4.832883 | 1.58E-12 | 3.58E-12 |
| CEP85 | 1.549214 | 3.24E-17 | 1.13E-16 |
| TMOD2 | 1.151338 | 9.25E-09 | 1.61E-08 |
| CCBE1 | -2.85642 | 2.54E-26 | 6.97E-25 |
| KCTD13 | 1.12767 | 4.82E-21 | 2.80E-20 |
| MAVS | 1.548938 | 1.23E-24 | 1.67E-23 |
| ZC2HC1A | 1.461362 | 5.28E-06 | 7.67E-06 |
| C8orf76 | 1.608825 | 2.20E-26 | 6.36E-25 |
| TMEM82 | -1.1668 | 5.41E-16 | 1.65E-15 |
| QARS | 1.271358 | 9.55E-28 | 7.23E-26 |
| HSPA1A | 1.410242 | 5.02E-12 | 1.09E-11 |
| TUBB2A | 1.006757 | 8.41E-07 | 1.29E-06 |
| DLK2 | 2.638864 | 2.04E-22 | 1.52E-21 |
| DNER | 8.025272 | 0.000118 | 0.000157 |
| PHOSPHO2 | 1.189905 | 1.16E-16 | 3.79E-16 |
| PUDP | 1.001769 | 3.71E-11 | 7.61E-11 |
| CYP4A11 | -1.68272 | 1.82E-24 | 2.35E-23 |
| WWC3 | 1.190672 | 1.13E-14 | 3.03E-14 |
| SLC1A3 | 1.798083 | 1.13E-11 | 2.40E-11 |
| NT5DC2 | 3.401841 | 3.56E-23 | 3.18E-22 |
| STEAP3 | -1.12636 | 2.11E-19 | 9.46E-19 |
| LLGL2 | 1.219503 | 9.64E-19 | 3.97E-18 |
| CCDC34 | 2.400841 | 3.13E-25 | 5.28E-24 |
| KLHL17 | 1.647585 | 1.52E-23 | 1.51E-22 |
| ZNF284 | 1.285438 | 8.10E-13 | 1.87E-12 |
| HIST2H2AC | 1.662066 | 8.39E-15 | 2.28E-14 |
| CNNM1 | 3.290813 | 0.022098 | 0.025177 |
| SPARCL1 | 3.347262 | 5.59E-17 | 1.89E-16 |
| COL11A1 | 5.106898 | 0.000211 | 0.000276 |
| SLC50A1 | 1.89003 | 7.74E-26 | 1.67E-24 |
| ZFP69B | 1.592859 | 8.39E-17 | 2.79E-16 |
| TECTB | 1.40249 | 0.019708 | 0.022517 |
| GPRC5D | 1.465281 | 7.00E-06 | 1.01E-05 |
| ZSCAN25 | 1.057001 | 1.05E-19 | 4.89E-19 |
| WDR90 | 1.41117 | 5.14E-19 | 2.18E-18 |
| RPL36 | 1.14829 | 5.97E-15 | 1.64E-14 |
| TMEM88 | 1.096135 | 7.06E-11 | 1.42E-10 |
| PIP5K1A | 1.060206 | 1.21E-14 | 3.24E-14 |
| SMIM29 | 1.530154 | 1.99E-23 | 1.92E-22 |
| ARID3A | 3.256725 | 2.49E-18 | 9.77E-18 |
| KIFC1 | 4.537156 | 3.95E-28 | 4.50E-26 |
| EEF2KMT | 1.273236 | 5.00E-24 | 5.59E-23 |
| RPL39L | 2.311598 | 1.21E-05 | 1.72E-05 |
| KIF14 | 3.915017 | 5.88E-26 | 1.35E-24 |
| SAP130 | 1.283953 | 1.37E-23 | 1.38E-22 |
| MAP10 | 1.506152 | 6.24E-13 | 1.45E-12 |
| FUT1 | 1.681774 | 5.48E-10 | 1.03E-09 |
| POLM | 1.399762 | 1.39E-26 | 4.39E-25 |
| ATAT1 | 1.789559 | 8.39E-23 | 6.84E-22 |
| ARHGEF17 | 1.041897 | 5.08E-05 | 6.93E-05 |
| MBL2 | -1.27189 | 7.42E-14 | 1.87E-13 |
| KLF13 | 1.713495 | 4.91E-19 | 2.10E-18 |
| CHMP3 | 1.031297 | 4.04E-17 | 1.39E-16 |
| CAVIN1 | 1.007271 | 1.37E-08 | 2.36E-08 |
| LYPD2 | -1.29914 | 1.23E-19 | 5.71E-19 |
| NFXL1 | 1.210078 | 9.98E-15 | 2.69E-14 |
| NSD2 | 1.870725 | 8.67E-24 | 9.16E-23 |
| LAYN | 1.507505 | 5.43E-11 | 1.10E-10 |
| NOVA2 | 1.095411 | 3.66E-14 | 9.46E-14 |
| ZNF888 | 1.961426 | 0.001618 | 0.002002 |
| PHF12 | 1.119597 | 7.33E-21 | 4.12E-20 |
| MAST1 | 2.073487 | 1.14E-08 | 1.98E-08 |
| GPX8 | 1.789566 | 1.64E-09 | 3.00E-09 |
| POLA1 | 1.541318 | 4.30E-19 | 1.85E-18 |
| ZNF439 | 1.137043 | 0.042927 | 0.047904 |
| CHKA | 1.783966 | 5.27E-22 | 3.65E-21 |
| NEURL3 | 2.234479 | 3.96E-05 | 5.44E-05 |
| HSD17B1 | 1.582834 | 3.60E-23 | 3.21E-22 |
| ZNF92 | 1.331159 | 5.19E-16 | 1.59E-15 |
| HOXA3 | 3.452335 | 3.81E-11 | 7.79E-11 |
| PUS7 | 1.364454 | 4.45E-19 | 1.91E-18 |
| ZNF821 | 1.195075 | 8.77E-16 | 2.61E-15 |
| DLG5 | 2.353447 | 8.84E-17 | 2.93E-16 |
| TSPAN8 | 2.416796 | 5.31E-08 | 8.80E-08 |
| CAMKK1 | 1.053043 | 5.76E-05 | 7.82E-05 |
| FABP5 | 2.386572 | 2.91E-19 | 1.28E-18 |
| HIST1H3E | 2.370936 | 6.92E-16 | 2.08E-15 |
| HIC2 | 1.990784 | 4.06E-18 | 1.56E-17 |
| IFT27 | 1.17475 | 1.06E-23 | 1.09E-22 |
| PTPN23 | 1.198039 | 2.43E-23 | 2.28E-22 |
| SPATA25 | 1.866091 | 5.46E-16 | 1.66E-15 |
| KCNK9 | 7.28133 | 1.11E-12 | 2.54E-12 |
| ARID3B | 1.222057 | 8.10E-13 | 1.87E-12 |
| DOK5 | 1.321045 | 0.000155 | 0.000204 |
| PLEKHN1 | 2.412149 | 6.14E-11 | 1.24E-10 |
| TMEM250 | 1.305714 | 6.51E-25 | 9.77E-24 |
| PIP4P1 | 1.166128 | 5.73E-26 | 1.33E-24 |
| TTC26 | 1.22655 | 5.99E-11 | 1.21E-10 |
| MYO5C | 1.509961 | 1.91E-15 | 5.51E-15 |
| ADRA1A | -2.56033 | 1.94E-24 | 2.48E-23 |
| FADS1 | 1.398518 | 4.01E-06 | 5.87E-06 |
| TSPAN17 | 1.482213 | 4.30E-26 | 1.06E-24 |
| RCAN1 | -1.79736 | 5.46E-24 | 6.07E-23 |
| PHETA2 | 1.449281 | 0.006268 | 0.007424 |
| CA5B | 1.945872 | 6.48E-16 | 1.96E-15 |
| AC010616.1 | 3.257895 | 0.004065 | 0.004889 |
| VPS72 | 1.745688 | 2.11E-28 | 3.44E-26 |
| MUC15 | 5.91502 | 0.006052 | 0.007177 |
| MLLT6 | 1.319781 | 9.00E-18 | 3.34E-17 |
| ZNF101 | 1.241927 | 2.88E-21 | 1.74E-20 |
| C1orf226 | 1.247978 | 3.64E-12 | 8.00E-12 |
| EXO1 | 4.34866 | 2.45E-27 | 1.35E-25 |
| RIC8A | 1.188313 | 4.02E-27 | 1.86E-25 |
| RUVBL2 | 1.159347 | 4.49E-25 | 7.06E-24 |
| CAPN12 | 2.174355 | 3.25E-11 | 6.68E-11 |
| BDKRB1 | 3.469197 | 5.68E-15 | 1.57E-14 |
| FHOD1 | 1.243432 | 1.85E-11 | 3.87E-11 |
| C7orf50 | 1.347325 | 2.12E-21 | 1.31E-20 |
| CRAMP1 | 1.628717 | 2.15E-23 | 2.04E-22 |
| RAVER2 | 1.851619 | 4.28E-06 | 6.25E-06 |
| MS4A6A | -1.15767 | 1.10E-14 | 2.95E-14 |
| HASPIN | 3.501181 | 9.82E-24 | 1.02E-22 |
| HEXA | 1.07083 | 1.23E-18 | 4.98E-18 |
| PREB | 1.207633 | 3.15E-23 | 2.87E-22 |
| HTATSF1 | 1.017496 | 3.01E-21 | 1.82E-20 |
| CCR10 | 1.953652 | 7.20E-10 | 1.35E-09 |
| NR4A1 | -1.51633 | 3.37E-14 | 8.71E-14 |
| DYRK1B | 1.229559 | 1.23E-15 | 3.62E-15 |
| MFSD6 | 1.933064 | 5.11E-15 | 1.41E-14 |
| CBX3 | 1.221811 | 2.24E-25 | 4.02E-24 |
| SLC39A7 | 1.465269 | 1.07E-25 | 2.16E-24 |
| ENSA | 1.23965 | 3.04E-27 | 1.53E-25 |
| BRIX1 | 1.118035 | 2.13E-19 | 9.55E-19 |
| TRIM27 | 1.178554 | 2.30E-25 | 4.08E-24 |
| DUSP26 | 2.108519 | 0.004644 | 0.005557 |
| MCAM | 2.075291 | 8.30E-22 | 5.55E-21 |
| WDR53 | 1.09709 | 6.26E-25 | 9.48E-24 |
| MKI67 | 4.142196 | 1.44E-26 | 4.55E-25 |
| GSTZ1 | -1.46296 | 2.84E-21 | 1.72E-20 |
| SMCR8 | 1.201235 | 5.80E-15 | 1.59E-14 |
| ANAPC1 | 1.056946 | 9.60E-17 | 3.17E-16 |
| ZNF829 | 1.080023 | 0.002677 | 0.003265 |
| PRR3 | 1.437317 | 1.64E-25 | 3.10E-24 |
| MYOM2 | -1.40264 | 3.89E-19 | 1.68E-18 |
| CSTB | 1.392248 | 1.45E-21 | 9.27E-21 |
| MAGED1 | 1.062222 | 2.36E-08 | 4.01E-08 |
| PPP1R16A | 1.706225 | 4.07E-23 | 3.56E-22 |
| PRRT3 | 1.716457 | 5.81E-23 | 4.92E-22 |
| PLAGL2 | 1.240568 | 4.07E-12 | 8.91E-12 |
| MTFR2 | 3.763397 | 3.44E-26 | 8.96E-25 |
| TRAM1L1 | 3.744895 | 2.68E-08 | 4.51E-08 |
| MIS18BP1 | 1.253715 | 4.68E-12 | 1.02E-11 |
| SECTM1 | 1.31209 | 9.90E-06 | 1.42E-05 |
| KLHDC8B | 1.349215 | 6.21E-17 | 2.09E-16 |
| PCDH12 | 1.181643 | 1.39E-15 | 4.06E-15 |
| IDO2 | -1.3119 | 9.61E-15 | 2.60E-14 |
| UNC119B | 1.8912 | 1.39E-20 | 7.46E-20 |
| TSPAN10 | 2.423116 | 3.66E-13 | 8.68E-13 |
| AQP10 | 5.425471 | 0.005191 | 0.006189 |
| ZNF142 | 1.537694 | 8.62E-25 | 1.23E-23 |
| HAGHL | 3.536518 | 1.02E-21 | 6.66E-21 |
| P4HA2 | 2.093794 | 1.64E-22 | 1.26E-21 |
| POLD3 | 1.292695 | 2.33E-19 | 1.04E-18 |
| KCNE5 | 3.671186 | 2.39E-13 | 5.75E-13 |
| NEDD4L | 1.45802 | 1.30E-19 | 5.99E-19 |
| ZNF620 | 1.091028 | 1.39E-12 | 3.17E-12 |
| FAM24B | 2.84878 | 4.00E-15 | 1.12E-14 |
| WDR73 | 1.026911 | 1.43E-14 | 3.80E-14 |
| AKAP17A | 1.10056 | 1.41E-19 | 6.46E-19 |
| IBSP | 8.174658 | 4.82E-11 | 9.81E-11 |
| DIPK2B | 2.807533 | 1.93E-29 | 1.44E-26 |
| LNPEP | 1.012701 | 8.78E-12 | 1.87E-11 |
| SLC26A2 | 1.935393 | 1.87E-22 | 1.42E-21 |
| RAE1 | 1.214195 | 9.65E-26 | 2.00E-24 |
| TTL | 1.490274 | 6.25E-27 | 2.44E-25 |
| RTL8C | 1.371445 | 1.97E-14 | 5.21E-14 |
| TPM3 | 1.263442 | 1.50E-23 | 1.49E-22 |
| COP1 | 1.328267 | 8.26E-27 | 2.97E-25 |
| ZNF333 | 1.059693 | 5.69E-16 | 1.73E-15 |
| SHKBP1 | 1.337553 | 6.38E-22 | 4.36E-21 |
| LGALS3 | 1.272749 | 4.98E-06 | 7.25E-06 |
| GTF3C5 | 1.120147 | 2.35E-24 | 2.91E-23 |
| SLC17A5 | 1.115198 | 1.05E-13 | 2.61E-13 |
| TPBGL | 3.647225 | 7.95E-15 | 2.16E-14 |
| GK5 | 1.371471 | 2.55E-17 | 8.98E-17 |
| CD58 | 1.417816 | 8.95E-20 | 4.23E-19 |
| POLR3A | 1.04707 | 7.34E-23 | 6.04E-22 |
| SPTAN1 | 1.31898 | 3.66E-25 | 5.93E-24 |
| AC007906.2 | -1.74307 | 3.85E-21 | 2.27E-20 |
| FADS2 | 1.056728 | 0.011186 | 0.012988 |
| HIST1H4E | 3.266091 | 2.46E-17 | 8.70E-17 |
| F9 | -1.36231 | 7.14E-20 | 3.42E-19 |
| NF2 | 1.346259 | 2.04E-24 | 2.58E-23 |
| CBFA2T3 | -1.29444 | 7.91E-18 | 2.95E-17 |
| EP400 | 1.153527 | 1.31E-18 | 5.30E-18 |
| INCENP | 2.033421 | 3.21E-20 | 1.63E-19 |
| CLDN6 | 2.731347 | 0.010854 | 0.012611 |
| TAF11 | 1.200971 | 2.44E-26 | 6.85E-25 |
| PRPF6 | 1.149428 | 3.56E-25 | 5.83E-24 |
| 3-Sep | 3.899421 | 3.86E-17 | 1.33E-16 |
| STX16 | 1.230394 | 2.15E-24 | 2.70E-23 |
| SP140 | 1.323724 | 0.003297 | 0.003996 |
| CALU | 1.110789 | 2.51E-14 | 6.56E-14 |
| FHDC1 | 2.809309 | 1.05E-12 | 2.40E-12 |
| C8orf33 | 1.91515 | 9.63E-29 | 2.81E-26 |
| OBSCN | 2.684748 | 0.026768 | 0.030322 |
| FAM189B | 2.363781 | 5.25E-29 | 2.50E-26 |
| VAX2 | 4.098194 | 4.22E-17 | 1.45E-16 |
| S100A11 | 1.707509 | 2.44E-05 | 3.41E-05 |
| P2RY6 | 1.453134 | 0.005837 | 0.00693 |
| ACTA2 | 1.250331 | 4.10E-10 | 7.80E-10 |
| TCOF1 | 1.738158 | 3.21E-27 | 1.59E-25 |
| NKIRAS2 | 1.172917 | 3.26E-26 | 8.62E-25 |
| SNRPA1 | 1.310949 | 1.65E-23 | 1.63E-22 |
| PRR16 | 2.038443 | 4.12E-13 | 9.73E-13 |
| CBX4 | 1.358808 | 4.81E-19 | 2.05E-18 |
| ZNF773 | 1.020007 | 4.69E-05 | 6.42E-05 |
| RRP1 | 1.246153 | 1.99E-22 | 1.49E-21 |
| PCDHGB2 | 1.586721 | 0.043688 | 0.048735 |
| DNA2 | 2.110923 | 5.67E-21 | 3.25E-20 |
| PAXX | 1.156485 | 1.73E-16 | 5.56E-16 |
| U2AF1L4 | 1.173016 | 6.88E-18 | 2.59E-17 |
| ARHGAP39 | 2.281404 | 1.46E-23 | 1.46E-22 |
| HMGA1 | 2.412214 | 5.29E-21 | 3.06E-20 |
| SMPD3 | -1.08888 | 4.31E-17 | 1.48E-16 |
| ZGRF1 | 2.09168 | 7.16E-23 | 5.92E-22 |
| TUBG2 | 1.62663 | 5.12E-24 | 5.72E-23 |
| PIGS | 1.169293 | 1.13E-14 | 3.03E-14 |
| MMRN1 | -1.34619 | 3.57E-12 | 7.87E-12 |
| TAF1D | 1.345585 | 4.71E-21 | 2.74E-20 |
| DCAF4L2 | 10.37221 | 1.04E-07 | 1.69E-07 |
| SPATS2 | 2.294772 | 7.09E-28 | 5.92E-26 |
| PLCXD1 | 1.567535 | 2.95E-09 | 5.29E-09 |
| CLEC14A | 1.083079 | 2.75E-11 | 5.68E-11 |
| COPE | 1.210815 | 2.45E-25 | 4.32E-24 |
| SGK494 | 1.647655 | 1.09E-11 | 2.31E-11 |
| MSX1 | 2.763016 | 8.45E-24 | 8.95E-23 |
| MVB12B | 1.036289 | 1.92E-12 | 4.31E-12 |
| SLC25A40 | 1.002931 | 2.47E-15 | 7.04E-15 |
| CDC25A | 2.887125 | 3.35E-21 | 2.00E-20 |
| MYL6B | 1.614971 | 1.27E-20 | 6.84E-20 |
| RASL12 | 2.909686 | 9.56E-27 | 3.31E-25 |
| CDK5 | 1.547452 | 1.87E-27 | 1.13E-25 |
| TGFB1 | 1.303598 | 0.00252 | 0.003079 |
| SPRTN | 1.099738 | 2.09E-21 | 1.30E-20 |
| ABCC10 | 2.04364 | 1.46E-26 | 4.59E-25 |
| RBM33 | 1.072989 | 1.30E-21 | 8.39E-21 |
| SPINK5 | 3.864661 | 7.88E-17 | 2.63E-16 |
| MAB21L4 | 1.596256 | 0.007058 | 0.008324 |
| SRGAP1 | 1.663099 | 1.02E-08 | 1.76E-08 |
| RPL7A | 1.043064 | 6.10E-16 | 1.85E-15 |
| HIST1H2AM | 3.164142 | 2.63E-20 | 1.35E-19 |
| CLIP4 | 1.20828 | 0.013447 | 0.015526 |
| GDF2 | -4.23731 | 1.12E-32 | 1.44E-28 |
| APC2 | 1.727784 | 1.02E-18 | 4.18E-18 |
| RPS15 | 1.07382 | 8.80E-15 | 2.38E-14 |
| FAM110A | 1.620284 | 6.59E-18 | 2.49E-17 |
| KIAA1549 | 2.138791 | 2.13E-05 | 2.98E-05 |
| DNAH14 | 1.966346 | 3.77E-26 | 9.59E-25 |
| RNF227 | 1.72096 | 6.84E-12 | 1.47E-11 |
| NFE2L3 | 1.794543 | 2.94E-10 | 5.65E-10 |
| CLDN7 | 1.555662 | 1.15E-06 | 1.75E-06 |
| TBX15 | -1.18225 | 6.78E-12 | 1.46E-11 |
| FAM53A | 1.654531 | 2.13E-14 | 5.62E-14 |
| ARHGAP6 | 1.103142 | 9.18E-07 | 1.40E-06 |
| MCCD1 | 6.982707 | 1.02E-05 | 1.45E-05 |
| TTK | 4.567796 | 3.81E-27 | 1.80E-25 |
| CRELD2 | 1.397601 | 4.85E-22 | 3.38E-21 |
| COPS5 | 1.186956 | 1.22E-27 | 8.33E-26 |
| CENPA | 4.889403 | 1.73E-27 | 1.07E-25 |
| C7orf77 | 2.762503 | 0.019876 | 0.0227 |
| PLPP1 | 1.042439 | 9.08E-10 | 1.69E-09 |
| ZDHHC11B | 1.299027 | 0.000836 | 0.001054 |
| ANKRD27 | 1.652426 | 1.12E-22 | 8.95E-22 |
| LRFN4 | 1.681074 | 0.000331 | 0.000427 |
| RNF214 | 1.031818 | 3.22E-18 | 1.25E-17 |
| CDH6 | 1.551108 | 0.003206 | 0.00389 |
| GTPBP3 | 1.289097 | 1.34E-25 | 2.61E-24 |
| DHX9 | 1.01308 | 1.08E-17 | 3.97E-17 |
| PLCE1 | 1.515233 | 9.85E-19 | 4.06E-18 |
| IQSEC1 | 1.097987 | 1.38E-18 | 5.59E-18 |
| MAP4K4 | 1.278231 | 3.00E-20 | 1.53E-19 |
| SLC44A4 | 1.685032 | 0.009303 | 0.010872 |
| RPAP1 | 1.193591 | 3.14E-24 | 3.73E-23 |
| PPBP | -1.42162 | 1.80E-17 | 6.47E-17 |
| ALG1L | 3.997041 | 7.72E-17 | 2.58E-16 |
| TIMD4 | -2.80671 | 1.26E-20 | 6.83E-20 |
| MAGEC2 | 9.603353 | 8.78E-09 | 1.53E-08 |
| EFHC1 | 1.469888 | 8.35E-18 | 3.10E-17 |
| GLYATL1 | -1.53655 | 3.26E-24 | 3.84E-23 |
| TRPM2 | 1.393243 | 5.54E-07 | 8.60E-07 |
| CLIP3 | 1.150076 | 0.008783 | 0.010292 |
| ID1 | -1.20676 | 1.65E-14 | 4.37E-14 |
| NEDD1 | 1.093161 | 5.12E-14 | 1.31E-13 |
| EZH1 | 1.313501 | 7.55E-24 | 8.13E-23 |
| NOSIP | 1.025013 | 4.07E-19 | 1.75E-18 |
| ZFP1 | -1.07951 | 3.84E-17 | 1.32E-16 |
| SNPH | 1.947612 | 3.80E-09 | 6.78E-09 |
| ACTL8 | 8.494578 | 6.85E-07 | 1.06E-06 |
| BCHE | -1.22071 | 5.74E-16 | 1.74E-15 |
| KNL1 | 1.402316 | 5.39E-14 | 1.37E-13 |
| CRTC1 | 1.099376 | 3.95E-20 | 1.96E-19 |
| PTGES3 | 1.063008 | 5.04E-25 | 7.82E-24 |
| CBLN1 | 2.108713 | 2.02E-09 | 3.66E-09 |
| NXF1 | 1.004601 | 1.62E-22 | 1.24E-21 |
| CRACR2B | 2.004683 | 4.54E-06 | 6.62E-06 |
| RAB41 | 1.648144 | 5.09E-18 | 1.94E-17 |
| PDZD11 | 1.331455 | 5.12E-28 | 4.91E-26 |
| CLEC1B | -4.77531 | 1.77E-29 | 1.44E-26 |
| KLHDC9 | 1.403364 | 1.92E-09 | 3.50E-09 |
| DOT1L | 1.182412 | 7.39E-16 | 2.22E-15 |
| DNASE1L3 | -2.42828 | 4.85E-25 | 7.56E-24 |
| CDHR3 | 1.209554 | 3.73E-14 | 9.63E-14 |
| GTF3C2 | 1.160885 | 7.28E-24 | 7.88E-23 |
| PSMD4 | 1.708901 | 2.02E-28 | 3.44E-26 |
| NDRG3 | 1.698344 | 2.64E-26 | 7.22E-25 |
| ZNF107 | 1.650122 | 1.03E-11 | 2.19E-11 |
| SLC7A6 | 2.482402 | 1.24E-22 | 9.79E-22 |
| ANKFN1 | 8.992326 | 2.29E-06 | 3.41E-06 |
| CEP131 | 2.491325 | 1.56E-29 | 1.44E-26 |
| GOLT1B | 1.161149 | 1.13E-23 | 1.15E-22 |
| TRPC1 | 1.562441 | 9.57E-06 | 1.37E-05 |
| PGK1 | 1.025514 | 2.21E-14 | 5.82E-14 |
| TMEM94 | 1.300593 | 1.67E-24 | 2.17E-23 |
| MSI2 | 1.3531 | 5.09E-16 | 1.55E-15 |
| P2RX5 | 1.540249 | 0.035459 | 0.039841 |
| HSPA1B | 1.331347 | 4.69E-08 | 7.79E-08 |
| RBM28 | 1.271062 | 4.08E-26 | 1.02E-24 |
| MN1 | 1.692003 | 0.001475 | 0.001829 |
| TRAF2 | 1.826032 | 3.25E-27 | 1.60E-25 |
| MMP24 | 2.262773 | 1.66E-06 | 2.50E-06 |
| ZNF624 | 1.153024 | 2.99E-11 | 6.16E-11 |
| EGR2 | -1.81224 | 1.71E-15 | 4.96E-15 |
| ZNF324B | 1.190864 | 3.88E-23 | 3.41E-22 |
| SPINK1 | 3.358956 | 5.20E-07 | 8.08E-07 |
| CEP68 | 1.301939 | 3.79E-24 | 4.40E-23 |
| FRS3 | 1.478793 | 1.29E-24 | 1.74E-23 |
| PLP2 | 2.221575 | 1.16E-13 | 2.87E-13 |
| KIF21B | 2.099518 | 1.10E-12 | 2.51E-12 |
| PML | 1.061959 | 9.29E-18 | 3.44E-17 |
| KNSTRN | 1.654386 | 1.59E-23 | 1.57E-22 |
| TUBA4A | 1.175528 | 2.96E-08 | 4.98E-08 |
| RFC5 | 1.252115 | 1.68E-19 | 7.67E-19 |
| COL27A1 | 1.115346 | 9.06E-08 | 1.48E-07 |
| VWF | 2.050686 | 2.34E-17 | 8.29E-17 |
| MARCO | -4.2941 | 9.04E-28 | 7.01E-26 |
| B3GNT3 | 2.628813 | 0.000302 | 0.000391 |
| SRD5A2 | -1.43373 | 6.08E-17 | 2.05E-16 |
| TDRKH | 1.910796 | 1.52E-20 | 8.11E-20 |
| LYSMD4 | 1.562147 | 3.60E-23 | 3.21E-22 |
| GPANK1 | 1.387494 | 1.03E-24 | 1.44E-23 |
| SCX | 3.94524 | 4.95E-27 | 2.12E-25 |
| PGS1 | 1.384022 | 3.10E-24 | 3.69E-23 |
| USP39 | 1.141449 | 1.95E-26 | 5.75E-25 |
| ITGB1BP1 | 1.544626 | 1.28E-26 | 4.16E-25 |
| FOXL1 | 2.450122 | 2.23E-08 | 3.79E-08 |
| SLC41A3 | 1.499637 | 1.20E-27 | 8.27E-26 |
| NETO2 | 2.936938 | 7.75E-24 | 8.28E-23 |
| JMJD6 | 1.104572 | 4.13E-17 | 1.42E-16 |
| VPS25 | 1.025527 | 2.63E-24 | 3.19E-23 |
| URB1 | 1.004634 | 1.84E-13 | 4.49E-13 |
| C4orf46 | 1.89974 | 2.78E-23 | 2.57E-22 |
| HIST2H2BE | 1.259648 | 5.03E-07 | 7.83E-07 |
| DCK | 1.344975 | 1.32E-15 | 3.87E-15 |
| CCDC65 | 1.479834 | 3.14E-17 | 1.10E-16 |
| DVL2 | 1.677617 | 6.59E-25 | 9.86E-24 |
| CABLES2 | 1.637694 | 4.34E-21 | 2.54E-20 |
| TSC2 | 1.167343 | 4.51E-22 | 3.16E-21 |
| TNFAIP8L3 | 1.280028 | 0.0007 | 0.000886 |
| ZNF219 | 1.947416 | 5.85E-27 | 2.37E-25 |
| VASH2 | 2.964148 | 1.02E-17 | 3.77E-17 |
| ASAP3 | 1.414713 | 9.28E-14 | 2.32E-13 |
| UNC5A | 3.739363 | 2.46E-12 | 5.49E-12 |
| CDCA5 | 4.162523 | 1.60E-28 | 3.39E-26 |
| CYP7A1 | 2.483355 | 0.000352 | 0.000453 |
| PCYOX1L | 1.313821 | 6.45E-11 | 1.30E-10 |
| C15orf39 | 1.645324 | 1.40E-18 | 5.64E-18 |
| TNFAIP6 | 2.587941 | 0.007496 | 0.008827 |
| PSMG4 | 1.103339 | 3.60E-20 | 1.80E-19 |
| DAGLA | 2.078738 | 8.66E-09 | 1.51E-08 |
| CCDC183 | 2.172891 | 1.06E-18 | 4.36E-18 |
| CCDC86 | 1.270392 | 3.95E-20 | 1.96E-19 |
| LRG1 | -1.10698 | 1.51E-11 | 3.18E-11 |
| MIS18A | 1.652804 | 2.96E-23 | 2.71E-22 |
| SFSWAP | 1.364695 | 5.26E-27 | 2.22E-25 |
| DMTF1 | 1.352474 | 2.14E-18 | 8.44E-18 |
| SHLD3 | 1.234007 | 1.72E-19 | 7.83E-19 |
| OGT | 1.372058 | 5.94E-21 | 3.40E-20 |
| MTX1 | 1.316184 | 3.72E-26 | 9.50E-25 |
| ZNF736 | 1.725004 | 4.59E-15 | 1.27E-14 |
| BTBD3 | 1.162856 | 4.22E-12 | 9.22E-12 |
| U2AF2 | 1.104236 | 8.95E-25 | 1.27E-23 |
| CCER2 | 2.363329 | 3.56E-17 | 1.24E-16 |
| PDPR | 1.191988 | 2.33E-15 | 6.65E-15 |
| PRSS2 | 10.46695 | 3.11E-06 | 4.59E-06 |
| SCN4B | 1.776694 | 1.66E-14 | 4.41E-14 |
| MAGEA12 | 8.511931 | 2.14E-06 | 3.20E-06 |
| ZNF398 | 1.111777 | 2.58E-23 | 2.40E-22 |
| MAPK3 | 1.378505 | 5.73E-26 | 1.33E-24 |
| ZNF646 | 1.534293 | 1.45E-24 | 1.93E-23 |
| LAMTOR4 | 1.324561 | 3.31E-21 | 1.99E-20 |
| POLR3C | 1.284888 | 1.45E-22 | 1.13E-21 |
| STK26 | 1.015393 | 0.024888 | 0.028246 |
| PAQR4 | 2.727883 | 7.33E-21 | 4.12E-20 |
| DCLRE1B | 1.47615 | 7.08E-21 | 3.99E-20 |
| PLXND1 | 1.447657 | 5.61E-18 | 2.13E-17 |
| MVP | 1.238846 | 1.89E-13 | 4.61E-13 |
| KDELC1 | 1.25929 | 8.66E-11 | 1.73E-10 |
| TCEAL9 | 1.391239 | 4.81E-06 | 7.01E-06 |
| THBS4 | 6.373337 | 1.45E-27 | 9.53E-26 |
| UCN | 1.905396 | 8.59E-16 | 2.56E-15 |
| SLC48A1 | 1.033825 | 7.90E-19 | 3.29E-18 |
| PLEC | 1.368927 | 1.95E-19 | 8.77E-19 |
| TDGF1 | 3.023943 | 3.96E-05 | 5.44E-05 |
| ZNF517 | 2.340418 | 1.05E-28 | 2.87E-26 |
| C10orf88 | 1.093653 | 8.70E-22 | 5.79E-21 |
| STAMBPL1 | 1.785053 | 3.27E-14 | 8.48E-14 |
| LEF1 | 3.567111 | 3.47E-17 | 1.21E-16 |
| KIRREL2 | 8.794653 | 1.59E-06 | 2.39E-06 |
| TCIM | -1.57501 | 9.47E-21 | 5.21E-20 |
| TXN | 1.35658 | 3.68E-17 | 1.27E-16 |
| RNF207 | 1.507168 | 3.45E-18 | 1.33E-17 |
| UTS2 | 2.58767 | 0.001018 | 0.001275 |
| CEP192 | 1.060449 | 5.17E-11 | 1.05E-10 |
| POPDC2 | 1.65294 | 1.93E-19 | 8.68E-19 |
| RNASEH1 | 1.144402 | 2.80E-24 | 3.37E-23 |
| SOSTDC1 | 3.757522 | 6.40E-07 | 9.91E-07 |
| KDM8 | -1.82292 | 1.11E-20 | 6.08E-20 |
| SLC43A2 | 1.256359 | 1.63E-08 | 2.80E-08 |
| PASK | 1.484983 | 1.22E-15 | 3.59E-15 |
| PFAS | 1.240158 | 5.61E-19 | 2.38E-18 |
| FATE1 | 5.20155 | 8.16E-26 | 1.73E-24 |
| MT3 | 4.881318 | 0.000988 | 0.00124 |
| LY6K | 4.876416 | 0.000102 | 0.000136 |
| MT1G | -1.93225 | 3.78E-23 | 3.34E-22 |
| ACTA1 | 2.073989 | 7.24E-11 | 1.45E-10 |
| MRTFA | 1.193897 | 3.47E-23 | 3.11E-22 |
| TP53I3 | 2.470917 | 1.47E-22 | 1.14E-21 |
| IGSF8 | 1.186134 | 7.42E-14 | 1.87E-13 |
| INPP5E | 1.25277 | 1.93E-19 | 8.68E-19 |
| MAGEC1 | 9.51278 | 1.09E-07 | 1.78E-07 |
| MKRN2OS | 2.187863 | 6.07E-06 | 8.78E-06 |
| CCDC43 | 1.052803 | 7.55E-20 | 3.60E-19 |
| CYB561 | 1.379172 | 4.43E-07 | 6.92E-07 |
| RAVER1 | 1.174582 | 2.31E-08 | 3.93E-08 |
| POLDIP3 | 1.014482 | 6.82E-20 | 3.27E-19 |
| NRM | 2.212588 | 4.54E-21 | 2.65E-20 |
| PRKCA | 1.475494 | 2.33E-19 | 1.04E-18 |
| MAPK12 | 2.784453 | 9.53E-19 | 3.93E-18 |
| CASTOR3 | 2.188302 | 3.26E-23 | 2.95E-22 |
| CLCN7 | 1.496308 | 2.92E-23 | 2.68E-22 |
| FBXL16 | 2.966658 | 0.00014 | 0.000186 |
| NR6A1 | 1.97935 | 1.80E-22 | 1.37E-21 |
| WDR83 | 1.342439 | 2.30E-25 | 4.08E-24 |
| GOLGA6L9 | 1.881048 | 9.05E-20 | 4.28E-19 |
| GLUL | 2.656971 | 0.000191 | 0.00025 |
| NINJ2 | 1.305804 | 3.20E-06 | 4.71E-06 |
| AP1G2 | 1.329619 | 1.02E-07 | 1.66E-07 |
| CDC45 | 3.79979 | 2.20E-26 | 6.36E-25 |
| PALM2 | -1.20329 | 2.84E-14 | 7.39E-14 |
| RPS14 | 1.001492 | 1.31E-15 | 3.83E-15 |
| CDCA8 | 3.957511 | 5.26E-28 | 5.01E-26 |
| VPS50 | 1.04328 | 2.69E-19 | 1.19E-18 |
| KCNJ5 | 2.64017 | 1.42E-17 | 5.16E-17 |
| MEX3A | 2.694376 | 7.86E-21 | 4.39E-20 |
| LAMA3 | 2.946004 | 2.00E-12 | 4.50E-12 |
| DHODH | -1.21715 | 4.99E-16 | 1.52E-15 |
| MEOX2 | 2.247903 | 1.87E-11 | 3.90E-11 |
| DXO | 1.569138 | 1.25E-26 | 4.10E-25 |
| ZNF789 | 1.725401 | 3.35E-26 | 8.78E-25 |
| SIPA1L2 | 1.808824 | 1.39E-09 | 2.57E-09 |
| LCN12 | 1.060613 | 1.64E-10 | 3.22E-10 |
| SLC25A19 | 1.548801 | 3.25E-20 | 1.64E-19 |
| FBLIM1 | 2.046443 | 2.28E-14 | 5.99E-14 |
| PFKP | 2.32215 | 1.35E-06 | 2.05E-06 |
| MAPK11 | 1.999835 | 1.08E-19 | 5.05E-19 |
| ACAP3 | 1.216782 | 1.06E-19 | 4.94E-19 |
| HRAS | 1.585403 | 7.59E-25 | 1.11E-23 |
| IGBP1 | 1.146218 | 1.19E-22 | 9.47E-22 |
| ZBTB41 | 1.556427 | 2.86E-17 | 1.00E-16 |
| NT5M | 1.954774 | 3.24E-17 | 1.13E-16 |
| S100A10 | 1.914281 | 1.88E-18 | 7.45E-18 |
| DRAP1 | 1.162921 | 4.14E-24 | 4.73E-23 |
| SPA17 | 1.468487 | 7.35E-14 | 1.85E-13 |
| SERHL2 | 1.803605 | 1.05E-05 | 1.50E-05 |
| ESAM | 1.010927 | 2.47E-13 | 5.94E-13 |
| METTL21A | 1.040933 | 1.92E-22 | 1.45E-21 |
| PRND | 4.646784 | 3.56E-15 | 1.00E-14 |
| CHRDL2 | 1.243593 | 0.002821 | 0.003436 |
| TMEM206 | 2.010015 | 9.31E-27 | 3.25E-25 |
| SCUBE1 | 2.709124 | 0.023346 | 0.026558 |
| PHIP | 1.04899 | 9.24E-12 | 1.97E-11 |
| TAX1BP3 | 1.615056 | 8.46E-14 | 2.12E-13 |
| SLC25A12 | 1.407011 | 1.99E-08 | 3.40E-08 |
| GCNT3 | 3.417831 | 3.53E-09 | 6.30E-09 |
| LTO1 | 2.751215 | 1.06E-25 | 2.14E-24 |
| CPXM1 | 2.984843 | 2.58E-16 | 8.14E-16 |
| HOXA13 | 7.556635 | 5.68E-21 | 3.26E-20 |
| PDE7A | 1.340756 | 3.24E-14 | 8.40E-14 |
| EIPR1 | 1.090102 | 8.60E-22 | 5.73E-21 |
| MYDGF | 1.049282 | 4.46E-22 | 3.13E-21 |
| IRGQ | 1.562057 | 1.05E-27 | 7.59E-26 |
| ZNF775 | 2.262425 | 1.50E-25 | 2.87E-24 |
| RBFOX2 | 1.026099 | 1.80E-15 | 5.21E-15 |
| BCO2 | -2.48965 | 6.26E-25 | 9.48E-24 |
| ZNF473 | 1.364561 | 7.74E-26 | 1.67E-24 |
| FOXQ1 | 2.201999 | 0.022965 | 0.02614 |
| C15orf65 | 1.175972 | 2.49E-11 | 5.15E-11 |
| HNRNPUL1 | 1.049631 | 8.10E-22 | 5.44E-21 |
| SH3RF2 | 1.324048 | 2.04E-09 | 3.71E-09 |
| MYH7B | 2.90024 | 1.39E-06 | 2.11E-06 |
| SUGP2 | 1.480758 | 2.68E-21 | 1.63E-20 |
| UBE2I | 1.065272 | 1.87E-22 | 1.42E-21 |
| TUBA4B | 2.079961 | 0.000386 | 0.000496 |
| PHYHIPL | 2.739674 | 5.85E-08 | 9.66E-08 |
| AGBL2 | 1.415836 | 7.70E-05 | 0.000104 |
| TMEM241 | 1.095155 | 5.16E-15 | 1.42E-14 |
| THOC2 | 1.16835 | 1.48E-18 | 5.93E-18 |
| ZKSCAN4 | 1.418412 | 9.47E-20 | 4.46E-19 |
| C9orf16 | 1.365243 | 1.58E-18 | 6.32E-18 |
| UBL4A | 1.410476 | 6.17E-27 | 2.42E-25 |
| FOXC2 | 2.450847 | 3.87E-11 | 7.91E-11 |
| FANCI | 3.018736 | 2.32E-26 | 6.61E-25 |
| TSLP | -1.77599 | 1.59E-20 | 8.46E-20 |
| TPPP2 | -1.56472 | 2.39E-20 | 1.24E-19 |
| F13A1 | 2.456453 | 2.11E-07 | 3.36E-07 |
| P2RX4 | 1.262878 | 5.20E-14 | 1.33E-13 |
| OTUD3 | 1.516272 | 1.11E-15 | 3.29E-15 |
| CHODL | 4.746851 | 0.039508 | 0.044246 |
| TCEAL8 | 1.075328 | 0.000127 | 0.000169 |
| OPRPN | 4.604886 | 0.015775 | 0.018142 |
| PRUNE1 | 1.472782 | 5.02E-23 | 4.31E-22 |
| FAM155B | 3.384589 | 1.68E-06 | 2.52E-06 |
| DPM3 | 1.121956 | 9.60E-13 | 2.20E-12 |
| PTPN14 | 1.954726 | 1.10E-13 | 2.73E-13 |
| CCDC71L | -1.12 | 9.53E-07 | 1.46E-06 |
| MMP10 | 4.564037 | 1.31E-05 | 1.86E-05 |
| FTH1 | 1.042351 | 8.93E-17 | 2.96E-16 |
| ACD | 1.27662 | 3.39E-23 | 3.05E-22 |
| IP6K1 | 1.541471 | 6.19E-28 | 5.45E-26 |
| NECTIN4 | 2.620887 | 0.011125 | 0.012918 |
| CCDC124 | 1.225276 | 2.52E-25 | 4.41E-24 |
| ZNF14 | 1.581939 | 8.57E-05 | 0.000115 |
| PDSS1 | 1.521897 | 1.29E-21 | 8.31E-21 |
| STXBP6 | 2.335904 | 1.06E-20 | 5.82E-20 |
| NR2C2 | 1.219943 | 2.68E-12 | 5.96E-12 |
| MAML1 | 1.16566 | 4.24E-18 | 1.63E-17 |
| PPIH | 1.295659 | 2.14E-22 | 1.58E-21 |
| COL4A2 | 2.301644 | 1.52E-21 | 9.68E-21 |
| HDAC1 | 1.026001 | 6.77E-19 | 2.84E-18 |
| CARD10 | 1.045864 | 5.96E-12 | 1.29E-11 |
| BAP1 | 1.061179 | 1.68E-23 | 1.65E-22 |
| TRIM31 | 2.831397 | 3.18E-11 | 6.55E-11 |
| PITPNM1 | 1.063731 | 4.40E-10 | 8.36E-10 |
| DIO2 | 4.965537 | 2.15E-08 | 3.66E-08 |
| MSANTD2 | 1.206502 | 2.55E-16 | 8.06E-16 |
| TSEN15 | 1.281388 | 1.71E-25 | 3.20E-24 |
| PFDN4 | 1.320288 | 1.75E-20 | 9.22E-20 |
| GPS2 | 1.227857 | 7.10E-17 | 2.38E-16 |
| UQCC3 | 1.043901 | 1.27E-16 | 4.14E-16 |
| DNAJC10 | 1.286408 | 8.08E-19 | 3.36E-18 |
| RAD18 | 1.50612 | 6.20E-26 | 1.40E-24 |
| RPP21 | 1.575584 | 1.85E-25 | 3.42E-24 |
| PLCG1 | 1.664063 | 1.77E-21 | 1.11E-20 |
| SLC7A2 | -1.1158 | 2.68E-17 | 9.44E-17 |
| ANKRD33 | 6.080949 | 0.00042 | 0.000539 |
| VPS45 | 1.785271 | 1.03E-29 | 1.32E-26 |
| PLAG1 | 2.434622 | 7.71E-09 | 1.35E-08 |
| DSCC1 | 2.787418 | 2.52E-28 | 3.60E-26 |
| ZNF580 | 1.776343 | 1.39E-25 | 2.68E-24 |
| TAF1B | 1.038643 | 1.10E-15 | 3.26E-15 |
| NEURL2 | 1.214286 | 4.13E-17 | 1.42E-16 |
| GAPDH | 1.248415 | 5.02E-20 | 2.46E-19 |
| 9-Sep | 1.159944 | 8.10E-22 | 5.44E-21 |
| VIRMA | 1.232872 | 5.59E-24 | 6.22E-23 |
| R3HDM1 | 1.321247 | 2.50E-24 | 3.07E-23 |
| MAP7D1 | 1.194612 | 9.39E-18 | 3.48E-17 |
| PAEP | 10.25909 | 2.23E-11 | 4.63E-11 |
| FBXL7 | 1.468529 | 4.35E-08 | 7.24E-08 |
| DYDC2 | 4.039943 | 4.21E-11 | 8.58E-11 |
| RRS1 | 1.511821 | 2.91E-16 | 9.11E-16 |
| YKT6 | 1.485135 | 4.08E-27 | 1.87E-25 |
| GPR89A | 1.130329 | 4.07E-23 | 3.56E-22 |
| DKC1 | 1.257654 | 3.72E-21 | 2.20E-20 |
| PKM | 2.651136 | 1.86E-14 | 4.94E-14 |
| ATAD3B | 1.521558 | 1.83E-20 | 9.61E-20 |
| NAA10 | 1.555228 | 2.23E-26 | 6.43E-25 |
| SLC4A3 | 3.37348 | 0.00058 | 0.000738 |
| COX20 | 1.068797 | 1.04E-15 | 3.07E-15 |
| BCAN | 5.182237 | 2.02E-21 | 1.25E-20 |
| ADAMTSL5 | 1.405885 | 1.82E-05 | 2.56E-05 |
| POLR2J | 1.0276 | 2.75E-19 | 1.22E-18 |
| SMC6 | 1.033352 | 4.16E-15 | 1.16E-14 |
| SH3BP5L | 1.550069 | 4.98E-27 | 2.13E-25 |
| CPA6 | 6.792112 | 9.17E-16 | 2.72E-15 |
| FUNDC1 | 1.041631 | 6.52E-18 | 2.46E-17 |
| MARK4 | 1.586297 | 1.30E-27 | 8.78E-26 |
| ZNF250 | 1.32291 | 2.10E-24 | 2.64E-23 |
| PDK1 | 1.10265 | 3.65E-08 | 6.11E-08 |
| ADAMTS14 | 2.193561 | 1.68E-10 | 3.28E-10 |
| PIGC | 1.734365 | 3.05E-28 | 3.96E-26 |
| AP3D1 | 1.116263 | 2.59E-22 | 1.89E-21 |
| SLAMF8 | 1.253046 | 3.44E-07 | 5.41E-07 |
| HSPBP1 | 1.047794 | 1.50E-20 | 8.04E-20 |
| KRT17 | 1.474689 | 5.11E-06 | 7.44E-06 |
| NME6 | 1.25153 | 3.81E-27 | 1.80E-25 |
| SPON2 | 1.691528 | 1.12E-16 | 3.68E-16 |
| EZH2 | 3.104109 | 4.59E-28 | 4.65E-26 |
| ZFYVE19 | 1.034227 | 1.79E-20 | 9.41E-20 |
| PLXNA1 | 2.023359 | 1.53E-17 | 5.53E-17 |
| TPCN1 | 1.372125 | 2.15E-20 | 1.12E-19 |
| TMEM107 | 1.048933 | 7.20E-12 | 1.55E-11 |
| ZNF335 | 1.432722 | 5.19E-27 | 2.20E-25 |
| DNAH1 | 1.515487 | 9.68E-13 | 2.22E-12 |
| MRPL52 | 1.117246 | 1.58E-21 | 1.00E-20 |
| LIN9 | 2.301167 | 1.07E-23 | 1.10E-22 |
| RHBDL2 | 1.750876 | 3.58E-08 | 5.99E-08 |
| LRRC73 | 1.493689 | 0.003498 | 0.004231 |
| ANAPC4 | 1.209941 | 1.17E-24 | 1.60E-23 |
| SUPT3H | 1.467262 | 8.93E-17 | 2.96E-16 |
| CEP295 | 1.560061 | 3.38E-18 | 1.31E-17 |
| RHEBL1 | 1.827218 | 8.75E-20 | 4.14E-19 |
| UHRF1 | 4.006189 | 6.42E-25 | 9.67E-24 |
| ZNF841 | 1.608945 | 3.99E-16 | 1.23E-15 |
| POP4 | 1.096415 | 2.32E-27 | 1.32E-25 |
| CCT6A | 1.509652 | 2.96E-27 | 1.51E-25 |
| TOPBP1 | 1.500112 | 5.37E-20 | 2.62E-19 |
| ARHGAP33 | 2.250556 | 2.84E-24 | 3.41E-23 |
| TCF3 | 1.760247 | 8.37E-26 | 1.77E-24 |
| FZD6 | 1.671738 | 7.54E-10 | 1.41E-09 |
| CHEK2 | 1.604562 | 2.80E-16 | 8.77E-16 |
| MAU2 | 1.122238 | 5.69E-20 | 2.76E-19 |
| ABCG1 | 1.219197 | 6.94E-05 | 9.38E-05 |
| IL1B | -1.06356 | 2.07E-10 | 4.03E-10 |
| CST2 | 3.857661 | 3.34E-07 | 5.26E-07 |
| MT2A | -1.98211 | 7.45E-22 | 5.04E-21 |
| FHIT | 1.516127 | 6.30E-22 | 4.32E-21 |
| PCDHB11 | 2.24039 | 1.54E-05 | 2.18E-05 |
| HSD11B2 | 1.407703 | 2.55E-10 | 4.93E-10 |
| ZCCHC17 | 1.089002 | 1.85E-23 | 1.80E-22 |
| DERL3 | 1.820421 | 1.25E-06 | 1.90E-06 |
| SH2D3A | 1.78577 | 8.78E-06 | 1.26E-05 |
| AKR1C1 | 1.221446 | 0.000384 | 0.000494 |
| FAM198A | -1.14605 | 2.11E-14 | 5.56E-14 |
| CNTD2 | 2.1278 | 0.002949 | 0.003585 |
| ATP13A2 | 1.57232 | 2.33E-19 | 1.04E-18 |
| VCAN | 2.702291 | 0.001654 | 0.002044 |
| RPL32 | 1.027831 | 1.46E-13 | 3.59E-13 |
| LILRB5 | -1.34969 | 1.27E-16 | 4.14E-16 |
| HIST1H2AC | 1.234019 | 1.59E-07 | 2.56E-07 |
| HIST1H2AE | 2.1555 | 3.01E-10 | 5.79E-10 |
| ALPI | 9.017572 | 4.57E-07 | 7.13E-07 |
| NME3 | 1.286787 | 6.38E-22 | 4.36E-21 |
| ALPK3 | 2.710736 | 3.80E-12 | 8.34E-12 |
| MYBL2 | 5.305148 | 3.92E-27 | 1.83E-25 |
| CABIN1 | 1.391071 | 3.22E-23 | 2.92E-22 |
| ANP32E | 1.029017 | 2.06E-16 | 6.55E-16 |
| RUBCN | 1.057994 | 1.65E-18 | 6.59E-18 |
| CMTM3 | 1.276785 | 1.35E-07 | 2.18E-07 |
| KLHL13 | 1.011177 | 0.019336 | 0.022105 |
| ACLY | 1.729162 | 4.16E-25 | 6.60E-24 |
| PLPPR2 | 1.75935 | 1.97E-25 | 3.58E-24 |
| HEATR6 | 1.320116 | 4.74E-20 | 2.33E-19 |
| ORM2 | -1.0585 | 2.57E-15 | 7.30E-15 |
| TEX11 | 4.780148 | 1.36E-05 | 1.93E-05 |
| ZNF213 | 1.735911 | 4.00E-22 | 2.84E-21 |
| CRMP1 | 1.717773 | 0.000106 | 0.000142 |
| GDF11 | 1.18641 | 0.025046 | 0.028421 |
| NEU3 | 1.177096 | 1.28E-17 | 4.67E-17 |
| SLC17A9 | 1.46636 | 3.90E-12 | 8.55E-12 |
| SIPA1L3 | 1.905525 | 1.39E-20 | 7.46E-20 |
| JOSD2 | 1.229313 | 4.77E-18 | 1.82E-17 |
| GEMIN2 | 1.042904 | 8.71E-18 | 3.23E-17 |
| ME1 | 1.66913 | 0.000434 | 0.000556 |
| CENPI | 3.8844 | 2.01E-26 | 5.89E-25 |
| HMGB3 | 1.196058 | 2.17E-09 | 3.93E-09 |
| SERPINI1 | 2.695321 | 1.52E-19 | 6.97E-19 |
| HIGD1B | 3.719754 | 8.34E-29 | 2.81E-26 |
| CEP19 | 1.333439 | 2.90E-12 | 6.43E-12 |
| LTBP2 | 1.908051 | 6.44E-08 | 1.06E-07 |
| S100A12 | -2.35595 | 4.41E-17 | 1.51E-16 |
| FAM193B | 1.45177 | 7.46E-24 | 8.04E-23 |
| ZNF341 | 1.827217 | 5.63E-28 | 5.13E-26 |
| RPS7 | 1.230343 | 3.01E-21 | 1.82E-20 |
| YY1AP1 | 1.244393 | 1.04E-23 | 1.08E-22 |
| GNG4 | 6.145102 | 6.19E-11 | 1.25E-10 |
| GSTM2 | 1.01262 | 0.000818 | 0.001032 |
| DNMT3B | 2.48024 | 7.59E-21 | 4.25E-20 |
| C2orf76 | 1.135864 | 2.20E-24 | 2.76E-23 |
| MIF | 1.301775 | 2.78E-13 | 6.67E-13 |
| GSTA4 | 1.731844 | 2.91E-16 | 9.11E-16 |
| NES | 1.989821 | 6.16E-22 | 4.22E-21 |
| DNHD1 | 1.36742 | 1.14E-13 | 2.82E-13 |
| ENTPD1 | 1.223624 | 2.79E-19 | 1.23E-18 |
| MAPK8IP2 | 3.856906 | 6.70E-19 | 2.81E-18 |
| IFT140 | 1.199854 | 1.00E-13 | 2.49E-13 |
| TTC23 | 1.107525 | 1.36E-17 | 4.96E-17 |
| SPHK1 | 2.849998 | 8.75E-05 | 0.000117 |
| RFLNA | 5.32714 | 7.46E-10 | 1.40E-09 |
| RAB10 | 1.022591 | 1.52E-20 | 8.11E-20 |
| HS1BP3 | 1.044207 | 1.74E-19 | 7.91E-19 |
| RBM15B | 1.013432 | 6.26E-23 | 5.25E-22 |
| TPRKB | 1.215099 | 6.45E-26 | 1.44E-24 |
| NCOR2 | 1.243819 | 1.06E-19 | 4.94E-19 |
| BRI3BP | 1.209493 | 3.81E-19 | 1.65E-18 |
| LY6E | -1.40267 | 3.07E-18 | 1.19E-17 |
| DENND6B | 1.483077 | 3.81E-19 | 1.65E-18 |
| SLC26A6 | 3.264918 | 1.25E-29 | 1.44E-26 |
| CBFA2T2 | 1.896098 | 1.16E-25 | 2.32E-24 |
| DPP3 | 1.024686 | 3.82E-20 | 1.90E-19 |
| COX6B1 | 1.190102 | 6.13E-19 | 2.58E-18 |
| NSUN5 | 1.522244 | 9.19E-27 | 3.22E-25 |
| DHX35 | 1.073225 | 9.69E-21 | 5.33E-20 |
| IL11 | 4.706871 | 7.00E-09 | 1.23E-08 |
| HSPA6 | 1.931063 | 0.000793 | 0.001001 |
| PUF60 | 1.5136 | 1.35E-26 | 4.33E-25 |
| CCDC130 | 1.289112 | 1.08E-24 | 1.50E-23 |
| CEP89 | 1.496024 | 2.29E-26 | 6.54E-25 |
| MIIP | 1.311502 | 6.60E-21 | 3.74E-20 |
| NUP93 | 1.38978 | 3.70E-24 | 4.31E-23 |
| C20orf204 | 5.691179 | 3.79E-24 | 4.40E-23 |
| KDM5B | 1.473827 | 1.38E-16 | 4.48E-16 |
| MFGE8 | 1.879664 | 1.72E-17 | 6.20E-17 |
| RNF39 | 1.700983 | 3.96E-05 | 5.44E-05 |
| LRRC14 | 2.136651 | 4.88E-30 | 1.08E-26 |
| RPL22L1 | 1.990466 | 1.30E-16 | 4.22E-16 |
| TUBA1B | 1.707167 | 1.04E-17 | 3.85E-17 |
| GAL3ST1 | 3.294795 | 0.000122 | 0.000162 |
| ABCB9 | 1.639728 | 3.88E-14 | 9.99E-14 |
| CFHR3 | -1.08429 | 1.29E-08 | 2.23E-08 |
| ARMC12 | 1.664289 | 7.65E-12 | 1.64E-11 |
| EGFL8 | 1.909623 | 6.81E-17 | 2.29E-16 |
| IPO9 | 1.424134 | 1.14E-25 | 2.29E-24 |
| SYTL1 | 1.043168 | 0.002551 | 0.003117 |
| MIOX | 4.851295 | 1.25E-11 | 2.64E-11 |
| CTU1 | 1.353442 | 3.33E-19 | 1.45E-18 |
| GLB1L | 1.363366 | 1.00E-13 | 2.49E-13 |
| CYTL1 | 2.252209 | 1.38E-11 | 2.91E-11 |
| RANGRF | 1.048248 | 5.33E-07 | 8.29E-07 |
| ASB6 | 1.044363 | 6.26E-23 | 5.25E-22 |
| CDK16 | 1.614526 | 3.10E-26 | 8.28E-25 |
| CXorf40B | 1.096371 | 7.65E-24 | 8.20E-23 |
| FAM117B | 1.106524 | 1.70E-05 | 2.39E-05 |
| DAB2 | 1.069053 | 0.008943 | 0.010472 |
| ARF1 | 1.047819 | 9.58E-24 | 1.00E-22 |
| ERP27 | 3.059103 | 0.000542 | 0.00069 |
| EEF1E1 | 1.333524 | 8.44E-18 | 3.14E-17 |
| PLEKHG5 | 1.552105 | 1.76E-17 | 6.32E-17 |
| LYRM4 | 1.445827 | 6.92E-24 | 7.55E-23 |
| RAPSN | 2.024123 | 4.63E-15 | 1.29E-14 |
| FOXO1 | -1.07571 | 4.28E-15 | 1.19E-14 |
| SLC25A51 | 1.074518 | 2.15E-17 | 7.65E-17 |
| FAM57A | 1.349179 | 3.13E-08 | 5.26E-08 |
| BATF | 1.543613 | 0.000362 | 0.000466 |
| TTLL4 | 2.168041 | 2.66E-19 | 1.18E-18 |
| ARHGEF1 | 1.055284 | 1.45E-20 | 7.79E-20 |
| TMEM229B | 1.068287 | 3.98E-05 | 5.47E-05 |
| CYP2C9 | -1.28053 | 2.02E-17 | 7.20E-17 |
| ZFAND2A | 1.076375 | 3.73E-15 | 1.05E-14 |
| PORCN | 1.633016 | 4.05E-22 | 2.87E-21 |
| ZNF605 | 1.820973 | 3.65E-24 | 4.27E-23 |
| NCS1 | 1.574139 | 2.87E-06 | 4.24E-06 |
| TSEN2 | 1.265362 | 9.34E-24 | 9.80E-23 |
| GINS1 | 3.498317 | 3.95E-25 | 6.33E-24 |
| ZMYM3 | 1.263388 | 3.56E-20 | 1.78E-19 |
| LAGE3 | 1.761416 | 2.15E-23 | 2.04E-22 |
| DUSP7 | 1.156119 | 1.84E-19 | 8.33E-19 |
| ZNF862 | 1.594632 | 1.52E-19 | 6.97E-19 |
| ZBTB22 | 1.220791 | 2.23E-23 | 2.11E-22 |
| MORN2 | 1.216554 | 3.29E-19 | 1.44E-18 |
| NRAS | 1.044759 | 7.64E-17 | 2.55E-16 |
| C3orf70 | 1.37035 | 1.52E-12 | 3.45E-12 |
| PPP1R14D | 5.012895 | 7.23E-13 | 1.68E-12 |
| CCDC61 | 1.228998 | 3.18E-24 | 3.76E-23 |
| CNIH4 | 1.751165 | 1.23E-27 | 8.36E-26 |
| RHOQ | 1.055453 | 9.73E-10 | 1.81E-09 |
| PRIM1 | 1.984405 | 4.14E-21 | 2.43E-20 |
| UCHL5 | 1.060211 | 3.21E-20 | 1.63E-19 |
| LZTS3 | 1.339699 | 4.68E-15 | 1.30E-14 |
| SPIRE2 | 1.890913 | 1.49E-24 | 1.97E-23 |
| BLVRA | 1.882255 | 5.93E-14 | 1.50E-13 |
| ZNF91 | 1.156368 | 1.76E-15 | 5.11E-15 |
| ANKS3 | 1.729183 | 5.30E-26 | 1.25E-24 |
| GPATCH8 | 1.047581 | 1.96E-13 | 4.78E-13 |
| HPS4 | 1.235725 | 3.39E-21 | 2.02E-20 |
| CREB3L1 | 3.126386 | 1.23E-05 | 1.74E-05 |
| GABRA3 | 7.018165 | 2.80E-06 | 4.14E-06 |
| ZNF606 | 1.296485 | 1.18E-13 | 2.92E-13 |
| ITGA5 | 1.287055 | 6.76E-14 | 1.70E-13 |
| CENPF | 4.697776 | 4.06E-28 | 4.50E-26 |
| ZNF71 | 1.798187 | 1.44E-17 | 5.21E-17 |
| ZNF608 | 1.085674 | 9.85E-06 | 1.41E-05 |
| PIEZO2 | 1.856866 | 4.00E-15 | 1.12E-14 |
| RABEP2 | 1.074099 | 6.14E-17 | 2.07E-16 |
| C6orf47 | 1.258091 | 3.03E-18 | 1.18E-17 |
| PYCR1 | 3.236659 | 9.35E-06 | 1.34E-05 |
| CENPE | 3.936098 | 5.54E-27 | 2.28E-25 |
| ADGRG2 | 2.150353 | 0.005372 | 0.006401 |
| LIG3 | 1.287442 | 1.80E-24 | 2.33E-23 |
| POLR3K | 1.148505 | 2.44E-21 | 1.49E-20 |
| TMEM9 | 1.420775 | 7.78E-25 | 1.12E-23 |
| AKR1B10 | 4.014172 | 1.73E-11 | 3.63E-11 |
| ZNF318 | 1.2382 | 1.14E-16 | 3.75E-16 |
| ONECUT2 | 1.101205 | 3.02E-09 | 5.41E-09 |
| ITGB4 | 2.372617 | 1.85E-10 | 3.62E-10 |
| PDXK | 1.33777 | 3.47E-23 | 3.11E-22 |
| MRPL17 | 1.086924 | 1.16E-21 | 7.53E-21 |
| C6orf89 | 1.0183 | 2.23E-24 | 2.79E-23 |
| APIP | 1.158051 | 3.30E-22 | 2.37E-21 |
| BNIPL | 2.384067 | 1.75E-21 | 1.10E-20 |
| PLPP7 | 2.192009 | 6.29E-07 | 9.74E-07 |
| CD177 | 3.50325 | 0.00124 | 0.001544 |
| CYP2C8 | -2.15993 | 6.04E-26 | 1.38E-24 |
| SFRP4 | 3.590695 | 3.73E-14 | 9.63E-14 |
| TBC1D7 | 1.463024 | 4.48E-27 | 1.97E-25 |
| SMYD5 | 1.394131 | 1.31E-23 | 1.32E-22 |
| LSM14B | 1.187947 | 1.16E-19 | 5.38E-19 |
| SSR3 | 1.057039 | 1.52E-25 | 2.89E-24 |
| TRIM16 | 3.616661 | 4.96E-23 | 4.27E-22 |
| GRPR | 4.614784 | 1.04E-09 | 1.93E-09 |
| PSMG3 | 1.678901 | 1.54E-25 | 2.91E-24 |
| GABRD | 5.131622 | 7.34E-30 | 1.28E-26 |
| VBP1 | 1.209625 | 3.01E-25 | 5.13E-24 |
| TIMM50 | 1.146365 | 6.33E-27 | 2.45E-25 |
| MPHOSPH6 | 1.02226 | 5.65E-17 | 1.91E-16 |
| C8A | -1.30318 | 1.40E-21 | 8.96E-21 |
| TAF5 | 1.041661 | 1.58E-20 | 8.37E-20 |
| HIST3H2A | 3.394857 | 0.007907 | 0.009295 |
| CPNE5 | 1.558985 | 1.52E-10 | 2.97E-10 |
| TMUB2 | 1.006769 | 9.48E-23 | 7.65E-22 |
| YJEFN3 | 2.482297 | 8.63E-19 | 3.57E-18 |
| GIT1 | 1.663467 | 2.48E-27 | 1.35E-25 |
| TCFL5 | 1.222519 | 1.30E-21 | 8.39E-21 |
| TMEM132A | 3.304651 | 1.06E-19 | 4.94E-19 |
| TMEM169 | 1.427343 | 2.98E-12 | 6.59E-12 |
| MC1R | 1.851085 | 6.51E-15 | 1.78E-14 |
| TRAIP | 3.535029 | 4.53E-28 | 4.65E-26 |
| SFT2D2 | 1.156793 | 2.41E-20 | 1.25E-19 |
| FZD2 | 1.997701 | 1.95E-05 | 2.73E-05 |
| RPL37 | 1.203179 | 1.10E-18 | 4.50E-18 |
| TMEM120B | 1.909643 | 4.57E-29 | 2.48E-26 |
| CLTRN | -1.83694 | 6.16E-21 | 3.51E-20 |
| LBX2 | 1.322135 | 2.90E-07 | 4.59E-07 |
| PRRX2 | 3.802074 | 6.92E-07 | 1.07E-06 |
| RANBP17 | 1.779602 | 0.000434 | 0.000556 |
| KIF4A | 4.697371 | 5.55E-28 | 5.13E-26 |
| ZSCAN29 | 1.079915 | 9.96E-19 | 4.10E-18 |
| C19orf53 | 1.221807 | 3.78E-23 | 3.34E-22 |
| CENPQ | 1.704681 | 3.93E-18 | 1.51E-17 |
| RHEB | 1.215202 | 2.98E-25 | 5.08E-24 |
| DCLK2 | 1.251285 | 2.77E-05 | 3.84E-05 |
| PFKM | 1.381398 | 3.07E-06 | 4.53E-06 |
| ADRA2C | 4.031048 | 7.92E-12 | 1.69E-11 |
| CCND2 | 1.586698 | 2.30E-08 | 3.90E-08 |
| GPR4 | 1.011882 | 2.77E-12 | 6.16E-12 |
| LENG8 | 1.511741 | 2.75E-19 | 1.22E-18 |
| ZNF282 | 1.404879 | 2.80E-27 | 1.45E-25 |
| RASSF7 | 1.338459 | 4.03E-16 | 1.24E-15 |
| NAA40 | 1.808921 | 2.77E-24 | 3.34E-23 |
| HDAC4 | 1.659395 | 9.14E-23 | 7.39E-22 |
| HOXD8 | 3.69385 | 6.75E-21 | 3.82E-20 |
| SNAPIN | 1.34628 | 1.65E-28 | 3.39E-26 |
| POLR1A | 1.492516 | 3.87E-26 | 9.76E-25 |
| ATP8B1 | 1.190759 | 4.64E-11 | 9.46E-11 |
| AP3M2 | 1.626641 | 1.67E-21 | 1.06E-20 |
| ZNF678 | 1.270673 | 6.95E-13 | 1.61E-12 |
| GAS8 | 1.206407 | 8.80E-18 | 3.27E-17 |
| TMEM44 | 1.294126 | 4.35E-13 | 1.03E-12 |
| C1R | -1.22901 | 9.14E-23 | 7.39E-22 |
| GLI1 | 2.12288 | 3.26E-10 | 6.25E-10 |
| NOP2 | 1.172539 | 1.01E-19 | 4.74E-19 |
| MACROD2 | 1.339473 | 0.00299 | 0.003636 |
| AIMP1 | 1.011258 | 4.75E-24 | 5.34E-23 |
| TICRR | 4.449294 | 4.36E-27 | 1.95E-25 |
| SUCO | 1.818736 | 1.89E-25 | 3.49E-24 |
| ABCA2 | 1.020797 | 1.32E-09 | 2.43E-09 |
| CEP97 | 1.075352 | 6.14E-11 | 1.24E-10 |
| DISP1 | 1.063209 | 2.50E-08 | 4.23E-08 |
| CCDC17 | 1.26417 | 5.17E-11 | 1.05E-10 |
| IGSF1 | 3.087089 | 0.000779 | 0.000984 |
| ERV3-1 | 1.513523 | 2.23E-11 | 4.63E-11 |
| VOPP1 | 1.138871 | 3.68E-17 | 1.27E-16 |
| CEP350 | 1.144016 | 1.90E-18 | 7.53E-18 |
| KCTD1 | 1.541367 | 3.13E-15 | 8.83E-15 |
| SLC22A5 | 1.458997 | 1.70E-23 | 1.66E-22 |
| AP1S3 | 1.194578 | 1.28E-07 | 2.07E-07 |
| RHOT2 | 1.08022 | 4.81E-24 | 5.40E-23 |
| CREB3L4 | 1.597858 | 5.87E-25 | 8.97E-24 |
| ABLIM2 | 2.112905 | 3.93E-12 | 8.62E-12 |
| TRPV2 | 1.100788 | 1.07E-05 | 1.52E-05 |
| MRM2 | 1.009507 | 4.65E-26 | 1.12E-24 |
| GHR | -1.84875 | 1.52E-21 | 9.68E-21 |
| MGAT5 | 1.615284 | 9.60E-17 | 3.17E-16 |
| NOXA1 | 1.005745 | 4.92E-11 | 9.99E-11 |
| IGFBP3 | -1.28369 | 2.64E-20 | 1.36E-19 |
| BOLA2B | 2.306783 | 4.48E-22 | 3.15E-21 |
| EME2 | 1.877938 | 4.35E-24 | 4.95E-23 |
| PLEKHF2 | 1.111636 | 1.93E-18 | 7.65E-18 |
| CCL28 | 1.835501 | 0.000587 | 0.000746 |
| DQX1 | 6.807089 | 2.14E-09 | 3.88E-09 |
| ENY2 | 1.073213 | 3.33E-20 | 1.68E-19 |
| TSNAX | 1.110426 | 1.29E-24 | 1.74E-23 |
| ZIK1 | 1.568153 | 0.033265 | 0.037454 |
| VMP1 | 1.036384 | 1.06E-18 | 4.36E-18 |
| C1QTNF3 | 4.61776 | 3.84E-17 | 1.32E-16 |
| HDAC7 | 1.407469 | 7.39E-15 | 2.02E-14 |
| GYS1 | 1.146402 | 3.57E-18 | 1.38E-17 |
| TMEM38B | 1.250936 | 8.83E-21 | 4.89E-20 |
| CDK3 | 1.696267 | 7.66E-14 | 1.92E-13 |
| MT1F | -2.38476 | 3.39E-23 | 3.05E-22 |
| UNC13B | 1.266268 | 1.27E-13 | 3.14E-13 |
| LRRC42 | 1.180639 | 4.45E-19 | 1.91E-18 |
| CEBPA | 1.298142 | 2.30E-10 | 4.46E-10 |
| FMO1 | 2.952447 | 0.003981 | 0.004791 |
| UTP20 | 1.073141 | 1.97E-15 | 5.67E-15 |
| ZNF345 | 1.098149 | 3.00E-14 | 7.81E-14 |
| BCL2L12 | 1.466422 | 6.65E-23 | 5.54E-22 |
| MESP2 | 4.065101 | 3.13E-25 | 5.28E-24 |
| THBS1 | -1.01657 | 1.49E-09 | 2.74E-09 |
| C1orf159 | 1.330869 | 1.30E-22 | 1.02E-21 |
| PLEKHJ1 | 1.189045 | 4.09E-20 | 2.02E-19 |
| NR2C2AP | 1.993758 | 5.05E-28 | 4.88E-26 |
| PRC1 | 3.994172 | 2.42E-28 | 3.60E-26 |
| COLEC12 | 3.1601 | 3.86E-09 | 6.87E-09 |
| RBPMS2 | 1.077891 | 2.74E-09 | 4.92E-09 |
| SHARPIN | 1.573038 | 9.69E-27 | 3.33E-25 |
| EEF1D | 1.189703 | 3.07E-20 | 1.56E-19 |
| TMEM119 | 2.375851 | 0.00031 | 0.0004 |
| ISL2 | 4.751215 | 7.58E-14 | 1.90E-13 |
| CLDN15 | 2.403808 | 1.25E-14 | 3.33E-14 |
| UPK3BL1 | 1.579799 | 0.009189 | 0.010746 |
| B9D1 | 1.642371 | 1.30E-19 | 5.99E-19 |
| GNAZ | 4.265691 | 4.50E-19 | 1.93E-18 |
| MPP6 | 1.112257 | 4.33E-12 | 9.44E-12 |
| HOXB13 | 6.991439 | 3.68E-07 | 5.77E-07 |
| SBSPON | 3.01809 | 6.84E-11 | 1.38E-10 |
| ZNF138 | 1.187067 | 2.35E-21 | 1.45E-20 |
| SLC39A3 | 1.160306 | 2.31E-23 | 2.18E-22 |
| C19orf18 | 1.135366 | 0.025447 | 0.028864 |
| ACP6 | 1.298536 | 2.41E-20 | 1.25E-19 |
| HKDC1 | 2.319664 | 5.80E-11 | 1.17E-10 |
| ZNF239 | 2.197457 | 0.000452 | 0.000578 |
| DMPK | 1.482299 | 4.40E-17 | 1.51E-16 |
| CD63 | 1.089944 | 2.82E-19 | 1.24E-18 |
| NEK3 | 1.236147 | 8.15E-10 | 1.52E-09 |
| RIPPLY3 | 2.602044 | 0.005617 | 0.006679 |
| IL1RAP | -1.29176 | 3.91E-16 | 1.21E-15 |
| PGBD5 | 1.446448 | 0.004866 | 0.005813 |
| GNPAT | 1.520565 | 1.98E-27 | 1.16E-25 |
| GOLGA8A | 2.362139 | 5.34E-14 | 1.36E-13 |
| DOK4 | 1.024209 | 1.94E-12 | 4.34E-12 |
| PIK3IP1 | 1.210312 | 2.14E-10 | 4.16E-10 |
| ALDH16A1 | 1.002878 | 3.83E-13 | 9.07E-13 |
| AGAP6 | 1.418206 | 2.44E-17 | 8.63E-17 |
| CLIC3 | 2.038342 | 0.031188 | 0.035185 |
| RALA | 1.029001 | 1.37E-23 | 1.38E-22 |
| ATP1B3 | 1.551636 | 1.73E-13 | 4.22E-13 |
| HGFAC | -1.2546 | 1.40E-14 | 3.73E-14 |
| CDCA7L | 1.592116 | 1.29E-06 | 1.95E-06 |
| PPP2R3B | 1.716788 | 8.37E-27 | 2.98E-25 |
| SCAMP3 | 1.926976 | 3.71E-29 | 2.27E-26 |
| VGLL4 | 1.056759 | 3.89E-08 | 6.49E-08 |
| CHD3 | 1.668893 | 4.26E-10 | 8.10E-10 |
| FAM83D | 3.150307 | 7.78E-25 | 1.12E-23 |
| ZNF782 | 1.120377 | 4.86E-15 | 1.35E-14 |
| ALKBH2 | 1.327663 | 9.58E-20 | 4.50E-19 |
| GPC3 | 6.534823 | 1.38E-21 | 8.86E-21 |
| TMEM80 | 1.308626 | 5.81E-23 | 4.92E-22 |
| CXCL17 | 4.933831 | 1.06E-06 | 1.61E-06 |
| B3GNTL1 | 2.116918 | 9.19E-27 | 3.22E-25 |
| TMEM258 | 1.182574 | 6.68E-25 | 9.97E-24 |
| SLC29A1 | 1.422679 | 1.18E-13 | 2.92E-13 |
| COX19 | 1.542712 | 6.79E-26 | 1.51E-24 |
| ZBTB3 | 1.011087 | 6.52E-20 | 3.14E-19 |
| CLEC2D | 1.082548 | 1.19E-07 | 1.92E-07 |
| INAVA | 3.187408 | 6.53E-05 | 8.83E-05 |
| ANKS6 | 2.065472 | 1.43E-09 | 2.62E-09 |
| SLC30A3 | 5.713631 | 3.30E-14 | 8.56E-14 |
| MAPRE1 | 1.054551 | 5.56E-12 | 1.20E-11 |
| GPR161 | 1.802954 | 8.66E-11 | 1.73E-10 |
| TFCP2 | 1.001322 | 1.28E-17 | 4.67E-17 |
| HSF1 | 1.456387 | 3.17E-25 | 5.33E-24 |
| LIFR | -2.18671 | 4.79E-25 | 7.47E-24 |
| CACTIN | 1.009745 | 1.37E-23 | 1.38E-22 |
| ARHGEF19 | 1.28267 | 1.21E-06 | 1.84E-06 |
| TECPR1 | 1.375795 | 5.58E-25 | 8.57E-24 |
| TMSB15A | 3.192409 | 1.27E-11 | 2.68E-11 |
| ZNF418 | 1.264545 | 0.002028 | 0.002494 |
| MCU | 1.005304 | 8.77E-16 | 2.61E-15 |
| TTC27 | 1.112754 | 4.91E-25 | 7.65E-24 |
| PTPDC1 | 1.606128 | 1.21E-18 | 4.93E-18 |
| TMEM144 | 1.261664 | 0.000548 | 0.000698 |
| AMDHD2 | 1.219219 | 1.28E-20 | 6.91E-20 |
| C11orf96 | -1.36217 | 9.08E-10 | 1.69E-09 |
| IGF2BP3 | 5.664024 | 1.66E-12 | 3.74E-12 |
| FBXO44 | 1.373281 | 2.06E-19 | 9.26E-19 |
| AMY2B | 1.21292 | 3.39E-06 | 4.99E-06 |
| LANCL1 | 1.093487 | 1.02E-17 | 3.77E-17 |
| HCN2 | 3.881482 | 5.41E-16 | 1.65E-15 |
| NDUFS8 | 1.093547 | 2.30E-20 | 1.20E-19 |
| MAPKBP1 | 1.285926 | 3.40E-20 | 1.71E-19 |
| ZNF850 | 1.044472 | 2.38E-06 | 3.54E-06 |
| SAP30 | 1.292846 | 1.01E-17 | 3.73E-17 |
| CCHCR1 | 2.092466 | 2.38E-28 | 3.60E-26 |
| HMBS | 1.095126 | 4.39E-23 | 3.81E-22 |
| AP5Z1 | 1.779145 | 7.11E-29 | 2.77E-26 |
| CD200 | 2.512462 | 6.38E-22 | 4.36E-21 |
| RRP7A | 1.0793 | 1.13E-17 | 4.13E-17 |
| MRAS | 1.679421 | 3.79E-13 | 9.00E-13 |
| TMEM270 | 2.643261 | 0.000375 | 0.000482 |
| KSR1 | 1.414244 | 2.77E-11 | 5.72E-11 |
| ZNF485 | 1.275323 | 1.83E-17 | 6.57E-17 |
| OSR1 | 2.471465 | 3.69E-07 | 5.79E-07 |
| SREBF2 | 1.245609 | 4.68E-17 | 1.60E-16 |
| ZNF174 | 1.336483 | 1.01E-26 | 3.44E-25 |
| SH3BP1 | 1.243559 | 0.000146 | 0.000193 |
| RPL14 | 1.13094 | 9.59E-18 | 3.55E-17 |
| ZNF85 | 2.103522 | 4.60E-11 | 9.38E-11 |
| OPRL1 | 1.274106 | 9.13E-07 | 1.40E-06 |
| AGXT2 | -1.25425 | 2.47E-18 | 9.67E-18 |
| DDR1 | 2.026764 | 0.000284 | 0.000369 |
| NIPAL3 | 1.096846 | 3.00E-16 | 9.38E-16 |
| FAM171A2 | 2.912241 | 1.78E-16 | 5.71E-16 |
| PRSS50 | 1.844605 | 7.50E-05 | 0.000101 |
| PUSL1 | 1.406713 | 7.95E-21 | 4.44E-20 |
| TM4SF19 | 3.073038 | 1.30E-09 | 2.40E-09 |
| GGCT | 1.200031 | 1.92E-23 | 1.86E-22 |
| BICD1 | 1.952409 | 1.70E-13 | 4.14E-13 |
| TUBGCP6 | 1.249357 | 1.41E-19 | 6.46E-19 |
| CA11 | 1.242522 | 7.74E-05 | 0.000104 |
| TMTC2 | 1.240494 | 0.005837 | 0.00693 |
| PEX6 | 1.118237 | 5.16E-15 | 1.42E-14 |
| THAP3 | 1.141448 | 1.56E-22 | 1.20E-21 |
| RPL6 | 1.045163 | 1.09E-20 | 5.95E-20 |
| KIZ | 1.477761 | 4.28E-23 | 3.73E-22 |
| TCEAL1 | 1.041608 | 4.67E-13 | 1.10E-12 |
| NEK8 | 1.172545 | 2.61E-18 | 1.02E-17 |
| MAT1A | -1.23797 | 2.22E-21 | 1.37E-20 |
| TSPYL2 | 1.101921 | 2.24E-10 | 4.36E-10 |
| MEF2D | 1.243643 | 5.47E-23 | 4.67E-22 |
| TRIP4 | 1.01068 | 4.30E-24 | 4.90E-23 |
| TIGD6 | 1.186383 | 1.14E-26 | 3.80E-25 |
| CRYBG2 | 3.092717 | 5.21E-22 | 3.61E-21 |
| VIPR1 | -2.93259 | 3.31E-28 | 4.02E-26 |
| STK35 | 1.340375 | 8.95E-20 | 4.23E-19 |
| RP9 | 1.135309 | 3.55E-22 | 2.54E-21 |
| BCAT2 | 1.400312 | 2.26E-18 | 8.89E-18 |
| SLC1A5 | 1.688436 | 0.004123 | 0.004956 |
| TMEM184B | 1.671385 | 1.39E-25 | 2.68E-24 |
| IDUA | 1.865197 | 1.02E-25 | 2.07E-24 |
| CAPS | 1.798043 | 3.35E-21 | 2.00E-20 |
| YIPF3 | 1.146614 | 5.69E-27 | 2.31E-25 |
| PRR29 | 1.324485 | 1.89E-15 | 5.47E-15 |
| CLCN2 | 1.736934 | 6.18E-24 | 6.80E-23 |
| CYTH3 | 1.417181 | 9.03E-16 | 2.68E-15 |
| HBB | -1.62951 | 1.47E-16 | 4.74E-16 |
| UBE2A | 1.31947 | 5.26E-27 | 2.22E-25 |
| AGBL5 | 1.494076 | 1.41E-23 | 1.41E-22 |
| ZMAT5 | 1.153642 | 1.92E-23 | 1.86E-22 |
| ZNF112 | 1.145817 | 8.00E-07 | 1.23E-06 |
| CSGALNACT1 | 1.327551 | 1.53E-06 | 2.30E-06 |
| KIAA1324 | 2.423198 | 1.20E-07 | 1.95E-07 |
| DTX2 | 1.036239 | 5.75E-20 | 2.79E-19 |
| CKS1B | 1.912743 | 1.43E-27 | 9.50E-26 |
| CD163L1 | 1.317038 | 1.14E-07 | 1.85E-07 |
| HAX1 | 1.148644 | 1.97E-25 | 3.58E-24 |
| CHST1 | 2.127177 | 5.48E-10 | 1.03E-09 |
| TRIM62 | 1.136613 | 3.45E-15 | 9.71E-15 |
| WASF2 | 1.354446 | 1.11E-19 | 5.16E-19 |
| STX6 | 1.496979 | 1.40E-24 | 1.87E-23 |
| NAA20 | 1.289213 | 7.54E-26 | 1.64E-24 |
| OIP5 | 3.036279 | 6.58E-24 | 7.20E-23 |
| LRFN3 | 1.005884 | 2.41E-14 | 6.32E-14 |
| MRTO4 | 1.054984 | 1.24E-20 | 6.69E-20 |
| DNMT1 | 1.906233 | 7.50E-21 | 4.21E-20 |
| IKZF4 | 1.028789 | 1.12E-12 | 2.55E-12 |
| HOGA1 | -1.205 | 3.61E-18 | 1.39E-17 |
| KAZALD1 | 2.434257 | 2.32E-14 | 6.10E-14 |
| C1QTNF12 | 2.331427 | 1.53E-13 | 3.76E-13 |
| SLC44A5 | 3.121486 | 0.005659 | 0.006728 |
| PHF19 | 2.141734 | 9.14E-23 | 7.39E-22 |
| MROH1 | 1.970307 | 2.11E-26 | 6.15E-25 |
| ZBTB45 | 1.498298 | 1.49E-27 | 9.64E-26 |
| PAQR5 | 2.613602 | 0.000543 | 0.000692 |
| PIDD1 | 1.444182 | 1.43E-24 | 1.91E-23 |
| JAG2 | 2.364662 | 3.69E-23 | 3.28E-22 |
| TOMM40L | 2.35842 | 4.12E-30 | 1.08E-26 |
| NDUFAF6 | 1.20862 | 1.61E-18 | 6.45E-18 |
| STKLD1 | 1.066628 | 3.08E-13 | 7.35E-13 |
| NDUFS6 | 1.267276 | 3.42E-22 | 2.45E-21 |
| GPSM1 | 2.61526 | 1.37E-15 | 4.02E-15 |
| TRIM66 | 1.411815 | 2.83E-17 | 9.92E-17 |
| SLC22A10 | -1.71585 | 4.33E-16 | 1.33E-15 |
| AHRR | 2.655396 | 2.58E-06 | 3.83E-06 |
| FAM241B | 1.746115 | 2.43E-11 | 5.03E-11 |
| RGL3 | 1.511845 | 2.41E-14 | 6.32E-14 |
| ZNF740 | 1.510414 | 4.97E-25 | 7.74E-24 |
| NDST2 | 1.025397 | 4.04E-21 | 2.37E-20 |
| CDK4 | 1.382142 | 8.35E-19 | 3.47E-18 |
| PDGFD | 1.680541 | 0.002449 | 0.002995 |
| TRIM16L | 3.085758 | 2.21E-13 | 5.35E-13 |
| AAK1 | 1.294957 | 9.26E-20 | 4.37E-19 |
| TBX2 | 1.543436 | 6.57E-14 | 1.66E-13 |
| USP27X | 1.10059 | 3.01E-05 | 4.18E-05 |
| FASTK | 1.339836 | 3.35E-26 | 8.78E-25 |
| ATIC | 1.268862 | 1.56E-23 | 1.54E-22 |
| MICB | 1.915426 | 1.08E-15 | 3.20E-15 |
| PBXIP1 | 1.050383 | 1.89E-20 | 9.92E-20 |
| MSLN | 3.548801 | 0.00533 | 0.006351 |
| CCDC78 | 3.055745 | 5.43E-18 | 2.06E-17 |
| CCDC22 | 1.055794 | 3.79E-24 | 4.40E-23 |
| OPHN1 | 1.570581 | 3.50E-09 | 6.26E-09 |
| RRP15 | 1.240041 | 5.59E-22 | 3.86E-21 |
| SIRT7 | 1.664527 | 7.04E-27 | 2.64E-25 |
| CCT5 | 1.385255 | 1.08E-23 | 1.11E-22 |
| C6 | -1.43425 | 1.38E-21 | 8.86E-21 |
| C9orf152 | 2.440557 | 0.003596 | 0.004345 |
| LAS1L | 1.102559 | 6.09E-27 | 2.40E-25 |
| SKA1 | 5.210785 | 1.42E-28 | 3.25E-26 |
| IP6K2 | 1.260731 | 1.17E-23 | 1.19E-22 |
| LPCAT4 | 1.500263 | 1.57E-10 | 3.07E-10 |
| G6PD | 3.372185 | 6.03E-25 | 9.16E-24 |
| ZNF296 | 2.852851 | 8.35E-18 | 3.10E-17 |
| ENTPD6 | 1.493246 | 7.12E-25 | 1.06E-23 |
| SCAF1 | 1.124726 | 1.00E-25 | 2.06E-24 |
| ATG16L2 | 1.025053 | 2.00E-13 | 4.86E-13 |
| CD1D | -1.00811 | 3.35E-15 | 9.43E-15 |
| MSH2 | 2.011174 | 8.59E-26 | 1.81E-24 |
| KCNQ1 | 1.765787 | 0.011344 | 0.013168 |
| CPLX2 | 9.775599 | 7.61E-15 | 2.07E-14 |
| SMPX | 3.877401 | 4.30E-09 | 7.64E-09 |
| BTG3 | 1.294336 | 9.90E-17 | 3.26E-16 |
| BIRC3 | 1.429417 | 0.001097 | 0.001371 |
| GMPS | 1.269855 | 3.78E-23 | 3.34E-22 |
| PEAK1 | 1.019858 | 1.11E-11 | 2.36E-11 |
| C15orf61 | 1.044132 | 1.62E-22 | 1.24E-21 |
| KIAA0100 | 1.548026 | 1.43E-22 | 1.12E-21 |
| CDK2 | 1.065195 | 8.63E-12 | 1.84E-11 |
| RSRP1 | 1.55437 | 5.56E-20 | 2.71E-19 |
| PROSER3 | 1.489219 | 1.24E-15 | 3.66E-15 |
| ATP5MF | 1.308405 | 1.78E-25 | 3.32E-24 |
| TRMU | 1.571157 | 9.95E-27 | 3.41E-25 |
| SMARCE1 | 1.00431 | 1.19E-22 | 9.47E-22 |
| HCFC1R1 | 1.203166 | 3.08E-19 | 1.35E-18 |
| TNFRSF10C | 1.342086 | 0.000122 | 0.000161 |
| KRTCAP2 | 1.800577 | 5.18E-29 | 2.50E-26 |
| CCDC167 | 1.458509 | 5.81E-21 | 3.32E-20 |
| IFT52 | 1.50248 | 8.56E-24 | 9.06E-23 |
| ZSCAN5A | 1.026693 | 3.29E-16 | 1.02E-15 |
| TMEM234 | 1.291979 | 2.16E-22 | 1.60E-21 |
| H2AFJ | 1.18628 | 1.65E-13 | 4.04E-13 |
| ABL2 | 1.321163 | 2.10E-20 | 1.10E-19 |
| STIP1 | 1.603837 | 1.37E-26 | 4.37E-25 |
| IFFO1 | 1.271474 | 2.46E-23 | 2.30E-22 |
| HARS2 | 1.23942 | 8.15E-27 | 2.95E-25 |
| KIF18A | 4.139527 | 7.94E-26 | 1.70E-24 |
| ATP6V1H | 1.218197 | 5.81E-23 | 4.92E-22 |
| TCHP | 1.402958 | 1.92E-23 | 1.86E-22 |
| ZNF836 | 1.240721 | 1.01E-18 | 4.14E-18 |
| APH1A | 1.005969 | 1.00E-21 | 6.60E-21 |
| NLRP1 | 1.400604 | 1.31E-09 | 2.42E-09 |
| FANCE | 2.383948 | 1.99E-23 | 1.92E-22 |
| IMPDH2 | 1.499543 | 3.89E-24 | 4.49E-23 |
| SCGB3A1 | -1.55823 | 2.34E-09 | 4.22E-09 |
| C12orf65 | 1.011735 | 3.43E-24 | 4.02E-23 |
| CD320 | 1.398697 | 1.19E-20 | 6.48E-20 |
| SPATA33 | 1.168748 | 1.49E-19 | 6.82E-19 |
| LEMD2 | 1.436238 | 1.78E-27 | 1.09E-25 |
| NOA1 | 1.131993 | 6.41E-23 | 5.37E-22 |
| HEG1 | 1.014227 | 2.79E-06 | 4.12E-06 |
| TMEM8B | 1.279873 | 1.82E-21 | 1.14E-20 |
| LYPD1 | 4.644478 | 1.41E-17 | 5.11E-17 |
| CTSA | 1.543074 | 7.49E-25 | 1.10E-23 |
| NHLRC1 | 1.290048 | 2.71E-14 | 7.06E-14 |
| NPY1R | -1.71623 | 1.64E-22 | 1.26E-21 |
| CPSF6 | 1.232908 | 4.07E-23 | 3.56E-22 |
| BYSL | 1.177899 | 1.45E-18 | 5.82E-18 |
| FDX2 | 1.293767 | 1.17E-19 | 5.44E-19 |
| C20orf96 | 1.707132 | 2.79E-25 | 4.80E-24 |
| ZNF320 | 2.238953 | 5.28E-06 | 7.67E-06 |
| RAB3A | 1.524765 | 3.34E-18 | 1.29E-17 |
| RGS10 | 1.245157 | 4.86E-08 | 8.05E-08 |
| FAM81A | 2.930374 | 7.13E-16 | 2.15E-15 |
| GNA14 | -1.17927 | 1.11E-18 | 4.54E-18 |
| OGG1 | 1.396436 | 7.28E-24 | 7.88E-23 |
| SLC5A9 | 1.20821 | 2.84E-06 | 4.20E-06 |
| POFUT2 | 1.001703 | 5.31E-15 | 1.47E-14 |
| FARSB | 1.215849 | 4.43E-25 | 6.99E-24 |
| APOLD1 | 1.192232 | 8.35E-09 | 1.46E-08 |
| RPS21 | 1.518479 | 7.06E-20 | 3.38E-19 |
| PPOX | 1.808773 | 1.44E-29 | 1.44E-26 |
| ARHGEF10 | 1.066292 | 0.000384 | 0.000494 |
| MSC | 3.294569 | 0.009105 | 0.010656 |
| FHL3 | 1.427672 | 7.69E-16 | 2.31E-15 |
| SPAG4 | 2.054052 | 7.99E-15 | 2.17E-14 |
| BLOC1S3 | 1.655523 | 6.11E-29 | 2.56E-26 |
| P2RY11 | 1.030516 | 6.95E-13 | 1.61E-12 |
| TNNC2 | 1.309797 | 2.51E-07 | 3.99E-07 |
| C9orf24 | 1.104221 | 0.000598 | 0.000759 |
| RAC3 | 1.613134 | 2.61E-12 | 5.81E-12 |
| CSNK2B | 1.203898 | 1.79E-21 | 1.13E-20 |
| SPC24 | 4.149261 | 4.17E-28 | 4.56E-26 |
| DLGAP5 | 4.496677 | 2.55E-27 | 1.37E-25 |
| CTXN1 | 3.118553 | 1.39E-12 | 3.17E-12 |
| SFN | 4.457248 | 3.83E-16 | 1.19E-15 |
| JTB | 1.02539 | 3.31E-23 | 2.99E-22 |
| URM1 | 1.096663 | 3.68E-22 | 2.63E-21 |
| EDARADD | 2.964575 | 5.70E-05 | 7.74E-05 |
| GGA2 | 1.088276 | 3.03E-20 | 1.54E-19 |
| FBXO41 | 1.659597 | 0.000538 | 0.000686 |
| GREB1L | 1.494076 | 3.56E-13 | 8.46E-13 |
| UTP14A | 1.299186 | 2.07E-24 | 2.61E-23 |
| ZNF532 | 1.722003 | 8.26E-12 | 1.77E-11 |
| ANKRD65 | 3.189072 | 1.85E-09 | 3.37E-09 |
| TRIM47 | 1.600531 | 3.67E-12 | 8.07E-12 |
| UIMC1 | 1.094863 | 1.42E-22 | 1.10E-21 |
| LCNL1 | 4.079973 | 0.038265 | 0.04291 |
| ZNF704 | 1.412555 | 9.10E-11 | 1.82E-10 |
| FGFR4 | 1.466063 | 3.10E-18 | 1.20E-17 |
| MYRF | 1.84029 | 5.26E-15 | 1.45E-14 |
| PCSK5 | 1.214822 | 7.39E-08 | 1.21E-07 |
| ADPRHL1 | 1.405692 | 1.18E-05 | 1.68E-05 |
| OPA3 | 1.019045 | 1.70E-22 | 1.30E-21 |
| RNFT2 | 3.406511 | 1.10E-21 | 7.19E-21 |
| CENPS | 1.137869 | 1.44E-14 | 3.84E-14 |
| ZNF7 | 1.5933 | 4.47E-28 | 4.65E-26 |
| ARMC9 | 1.796241 | 5.47E-15 | 1.51E-14 |
| CAP2 | 2.878983 | 7.98E-25 | 1.15E-23 |
| PET117 | 1.042096 | 2.49E-19 | 1.11E-18 |
| BEX3 | 1.248549 | 3.70E-05 | 5.09E-05 |
| ARMC2 | 1.260352 | 6.12E-18 | 2.31E-17 |
| PSPC1 | 1.014529 | 5.31E-19 | 2.25E-18 |
| SLC5A6 | 1.067219 | 6.10E-08 | 1.01E-07 |
| CCL14 | -1.35713 | 1.41E-19 | 6.46E-19 |
| WDR12 | 1.00356 | 2.68E-20 | 1.37E-19 |
| TAF9 | 1.241908 | 1.87E-22 | 1.42E-21 |
| STXBP1 | 1.280169 | 2.11E-06 | 3.15E-06 |
| BCAT1 | 2.462469 | 4.83E-17 | 1.65E-16 |
| SF3B4 | 1.759261 | 3.52E-27 | 1.70E-25 |
| RAB4B | 1.000489 | 5.57E-16 | 1.69E-15 |
| ZNF599 | 1.120491 | 1.95E-13 | 4.73E-13 |
| KHDRBS3 | 1.123427 | 6.02E-10 | 1.13E-09 |
| PCBP4 | 1.7123 | 2.96E-23 | 2.71E-22 |
| TBC1D10C | 1.040664 | 0.000521 | 0.000665 |
| GOLGA8B | 2.245142 | 9.46E-22 | 6.23E-21 |
| TRAPPC4 | 1.04983 | 1.65E-18 | 6.59E-18 |
| HTR3A | 6.049049 | 0.000548 | 0.000698 |
| CBX6 | 1.218659 | 0.000297 | 0.000384 |
| CENPL | 3.102394 | 3.31E-28 | 4.02E-26 |
| BCL6B | 1.385157 | 5.77E-17 | 1.95E-16 |
| POLE2 | 2.331222 | 5.74E-23 | 4.88E-22 |
| STOML1 | 1.346211 | 2.55E-25 | 4.44E-24 |
| FOCAD | 1.39397 | 3.69E-23 | 3.28E-22 |
| HOMER3 | 2.000666 | 1.62E-14 | 4.29E-14 |
| MPZL1 | 1.570162 | 5.40E-22 | 3.73E-21 |
| BMT2 | 1.081606 | 7.46E-15 | 2.03E-14 |
| ZNF764 | 1.231934 | 5.17E-25 | 7.98E-24 |
| SLC9A1 | 1.065096 | 5.54E-08 | 9.16E-08 |
| KRT12 | 8.036004 | 2.48E-06 | 3.69E-06 |
| ZNF222 | 1.017336 | 1.86E-09 | 3.40E-09 |
| RASSF3 | 1.260985 | 2.46E-09 | 4.44E-09 |
| COX7B2 | 9.868374 | 6.87E-10 | 1.29E-09 |
| NLE1 | 1.462964 | 4.66E-23 | 4.03E-22 |
| NDST1 | 1.397771 | 1.44E-20 | 7.71E-20 |
| PACS1 | 1.188964 | 4.41E-15 | 1.23E-14 |
| ZNF3 | 1.070995 | 2.05E-25 | 3.71E-24 |
| WISP2 | -1.2286 | 7.51E-06 | 1.08E-05 |
| NSMCE2 | 2.035645 | 6.37E-29 | 2.56E-26 |
| CCDC187 | 4.125719 | 2.84E-08 | 4.78E-08 |
| MYO15B | 2.05159 | 2.49E-19 | 1.11E-18 |
| FASN | 1.653046 | 1.74E-12 | 3.92E-12 |
| TLR5 | 1.28581 | 6.73E-05 | 9.10E-05 |
| RIMS3 | 1.829181 | 8.26E-12 | 1.77E-11 |
| DSTYK | 1.732188 | 3.60E-23 | 3.21E-22 |
| MAPT | 3.728369 | 3.34E-25 | 5.55E-24 |
| UBXN2B | 1.078024 | 2.60E-14 | 6.81E-14 |
| MEX3D | 1.207406 | 2.03E-14 | 5.36E-14 |
| PFDN2 | 1.220627 | 1.32E-22 | 1.03E-21 |
| SGSM2 | 1.270744 | 1.12E-16 | 3.68E-16 |
| CENPX | 1.6994 | 2.23E-20 | 1.16E-19 |
| F2RL3 | 2.953886 | 1.95E-16 | 6.24E-16 |
| SLC25A39 | 1.412758 | 5.70E-28 | 5.13E-26 |
| ZIC2 | 7.497798 | 1.78E-24 | 2.30E-23 |
| TNFSF9 | 2.0472 | 0.000332 | 0.000428 |
| SLCO1B3 | -1.46492 | 6.72E-16 | 2.03E-15 |
| ACRBP | 1.000302 | 6.94E-05 | 9.38E-05 |
| GPR182 | -2.82975 | 2.09E-23 | 2.00E-22 |
| CSRNP1 | -1.62917 | 1.32E-22 | 1.03E-21 |
| TAL2 | 3.345631 | 7.59E-15 | 2.07E-14 |
| GTF3C3 | 1.082282 | 1.10E-21 | 7.19E-21 |
| PAGE2B | 6.857373 | 4.92E-07 | 7.67E-07 |
| MT1M | -1.72543 | 4.33E-23 | 3.77E-22 |
| ARSG | 1.142777 | 1.27E-10 | 2.50E-10 |
| GGA3 | 1.400821 | 1.97E-25 | 3.58E-24 |
| C17orf53 | 2.678828 | 2.52E-23 | 2.35E-22 |
| RPS5 | 1.396294 | 1.24E-21 | 8.04E-21 |
| MYO18B | 7.894855 | 5.23E-17 | 1.77E-16 |
| KRTAP5-6 | 2.346576 | 3.46E-11 | 7.10E-11 |
| PRKRIP1 | 1.513158 | 1.72E-28 | 3.39E-26 |
| SLC25A29 | 1.570588 | 8.70E-22 | 5.79E-21 |
| RBPJL | 8.602199 | 0.001852 | 0.002282 |
| IL17RB | 1.175775 | 1.07E-07 | 1.73E-07 |
| NDUFA3 | 1.026477 | 5.85E-15 | 1.61E-14 |
| SCNM1 | 2.029782 | 3.13E-28 | 4.01E-26 |
| OSBPL3 | 2.449764 | 3.68E-22 | 2.63E-21 |
| PPM1M | 1.092778 | 8.81E-11 | 1.76E-10 |
| CPT1C | 1.664019 | 1.18E-13 | 2.92E-13 |
| WDR70 | 1.101408 | 3.97E-27 | 1.84E-25 |
| SIX1 | 5.662527 | 4.08E-23 | 3.57E-22 |
| PPDPF | 1.353987 | 1.30E-10 | 2.56E-10 |
| SMIM31 | 4.281881 | 0.009447 | 0.011037 |
| MEIS2 | 1.190645 | 4.66E-07 | 7.27E-07 |
| TMEM68 | 1.154475 | 2.44E-21 | 1.49E-20 |
| DBF4B | 2.305002 | 1.37E-25 | 2.66E-24 |
| STK25 | 1.14583 | 3.92E-26 | 9.87E-25 |
| UTP6 | 1.091858 | 4.14E-24 | 4.73E-23 |
| HMGN1 | 1.292963 | 3.64E-20 | 1.82E-19 |
| ACSM1 | 1.918951 | 0.000302 | 0.000391 |
| CDCA2 | 3.967566 | 5.95E-25 | 9.06E-24 |
| MYPOP | 1.365942 | 1.82E-25 | 3.38E-24 |
| PPIL3 | 1.060883 | 9.37E-20 | 4.41E-19 |
| WDR55 | 1.003494 | 1.99E-24 | 2.53E-23 |
| HRH1 | 1.295531 | 2.92E-07 | 4.62E-07 |
| TMEM240 | 1.508178 | 8.94E-10 | 1.67E-09 |
| STK32C | 1.020223 | 5.97E-10 | 1.12E-09 |
| NPIPA5 | 1.123085 | 0.0003 | 0.000388 |
| GGH | 1.519053 | 2.74E-10 | 5.27E-10 |
| ETV2 | 1.377577 | 1.38E-17 | 5.01E-17 |
| TTC9C | 1.100291 | 5.88E-26 | 1.35E-24 |
| MSH5 | 2.977283 | 1.82E-21 | 1.14E-20 |
| SLC39A4 | 2.349669 | 0.014548 | 0.016757 |
| KCTD7 | 2.001459 | 3.39E-24 | 3.97E-23 |
| TPM1 | 1.132356 | 7.63E-14 | 1.92E-13 |
| MCC | -1.09608 | 8.09E-16 | 2.42E-15 |
| ADAMDEC1 | 2.127007 | 3.13E-07 | 4.94E-07 |
| BOLA1 | 1.438861 | 3.13E-25 | 5.28E-24 |
| CYP1A2 | -2.70823 | 4.83E-24 | 5.42E-23 |
| ARHGAP4 | 1.679642 | 1.27E-11 | 2.68E-11 |
| KLC2 | 1.845154 | 8.67E-24 | 9.16E-23 |
| LPAR2 | 1.717423 | 0.000749 | 0.000947 |
| AKR1D1 | -1.30681 | 8.77E-16 | 2.61E-15 |
| CPNE1 | 1.338011 | 2.07E-20 | 1.08E-19 |
| UTP25 | 1.03541 | 9.50E-17 | 3.14E-16 |
| GPIHBP1 | 1.452879 | 3.67E-10 | 7.01E-10 |
| STAU2 | 1.015457 | 1.70E-18 | 6.79E-18 |
| LRRC39 | 1.940692 | 7.70E-14 | 1.93E-13 |
| PIMREG | 3.989278 | 1.61E-17 | 5.79E-17 |
| TMEM199 | 1.096396 | 6.20E-26 | 1.40E-24 |
| MMP9 | 2.720729 | 2.14E-08 | 3.64E-08 |
| TOMM20L | 1.115488 | 1.89E-08 | 3.23E-08 |
| CCNL2 | 1.340052 | 8.17E-18 | 3.04E-17 |
| BDKRB2 | 1.967835 | 8.58E-08 | 1.40E-07 |
| PACSIN2 | 1.119865 | 2.02E-16 | 6.43E-16 |
| SUV39H2 | 1.456906 | 8.29E-25 | 1.18E-23 |
| GDPD1 | 1.878497 | 1.89E-17 | 6.78E-17 |
| ZNF430 | 1.07516 | 0.001315 | 0.001636 |
| KAT2A | 2.142235 | 1.06E-27 | 7.60E-26 |
| TYMP | 1.023739 | 1.52E-06 | 2.29E-06 |
| HSPA12A | 1.649982 | 8.63E-07 | 1.32E-06 |
| E2F2 | 3.718069 | 3.43E-25 | 5.67E-24 |
| SHISAL2A | 2.469606 | 0.002776 | 0.003383 |
| CDC123 | 1.084702 | 3.34E-25 | 5.55E-24 |
| DYNC2LI1 | 1.023919 | 2.41E-20 | 1.25E-19 |
| ABHD3 | 1.019382 | 1.10E-09 | 2.04E-09 |
| PHKG1 | 1.45041 | 9.26E-20 | 4.37E-19 |
| CENPW | 3.317643 | 8.45E-28 | 6.67E-26 |
| SCARA5 | -1.01148 | 4.08E-17 | 1.40E-16 |
| GLIS2 | 1.757863 | 2.26E-06 | 3.36E-06 |
| FCN2 | -4.39667 | 5.64E-29 | 2.53E-26 |
| MTHFSD | 1.038635 | 2.52E-23 | 2.35E-22 |
| EVPL | 3.594165 | 0.009165 | 0.010721 |
| HIST1H2AG | 2.678812 | 5.01E-08 | 8.30E-08 |
| B3GALT4 | 1.074673 | 0.025447 | 0.028864 |
| GNAI1 | 1.014646 | 1.36E-08 | 2.34E-08 |
| DNAJB11 | 1.510987 | 1.00E-28 | 2.81E-26 |
| SLC39A13 | 1.347166 | 4.30E-26 | 1.06E-24 |
| CADM4 | 1.021687 | 0.001882 | 0.002318 |
| SDF2L1 | 1.481545 | 1.81E-20 | 9.51E-20 |
| GRK3 | 1.519591 | 0.000106 | 0.000141 |
| MICAL1 | 1.680498 | 6.82E-14 | 1.72E-13 |
| C11orf98 | 1.11091 | 2.97E-20 | 1.51E-19 |
| S100P | 6.449663 | 1.13E-07 | 1.84E-07 |
| UBXN11 | 1.498624 | 3.03E-18 | 1.18E-17 |
| SEMA5B | 3.054072 | 4.13E-27 | 1.88E-25 |
| TTF2 | 1.740866 | 4.62E-18 | 1.77E-17 |
| POGK | 1.39978 | 6.09E-20 | 2.95E-19 |
| DUSP23 | 1.262389 | 7.11E-18 | 2.67E-17 |
| NCDN | 1.356338 | 2.93E-20 | 1.50E-19 |
| C17orf80 | 1.253681 | 2.25E-23 | 2.13E-22 |
| VPS37C | 1.202059 | 2.50E-22 | 1.83E-21 |
| BCLAF3 | 1.151031 | 2.68E-17 | 9.44E-17 |
| CHCHD3 | 1.108662 | 1.61E-21 | 1.02E-20 |
| COIL | 1.068152 | 4.85E-22 | 3.38E-21 |
| SULF1 | 2.186425 | 0.006482 | 0.007668 |
| ZNF512B | 1.631053 | 6.49E-23 | 5.43E-22 |
| HSP90AB1 | 1.634118 | 3.53E-26 | 9.14E-25 |
| BRF2 | 1.126861 | 2.05E-14 | 5.41E-14 |
| PACS2 | 1.427756 | 9.53E-26 | 1.98E-24 |
| CD300E | -1.0809 | 4.22E-09 | 7.50E-09 |
| FBXW10 | 4.770681 | 2.24E-09 | 4.06E-09 |
| CCL13 | 2.587983 | 2.62E-06 | 3.89E-06 |
| KIF23 | 4.112442 | 8.04E-26 | 1.72E-24 |
| HTT | 1.015996 | 4.74E-20 | 2.33E-19 |
| GPRC5B | 1.013279 | 0.034087 | 0.038346 |
| TGFB2 | 2.008395 | 9.02E-05 | 0.000121 |
| COX4I2 | 3.399559 | 2.98E-27 | 1.51E-25 |
| CYP17A1 | 4.898637 | 2.78E-05 | 3.86E-05 |
| CEP83 | 1.229239 | 5.35E-21 | 3.09E-20 |
| CAPN8 | 3.579271 | 2.89E-12 | 6.40E-12 |
| ZDHHC11 | 1.25348 | 0.00016 | 0.00021 |
| ATR | 1.0369 | 1.97E-16 | 6.30E-16 |
| PSCA | 4.961838 | 1.77E-12 | 4.00E-12 |
| ERVK3-1 | 1.316672 | 5.16E-26 | 1.23E-24 |
| DPEP1 | 2.995749 | 0.006802 | 0.008035 |
| SLC7A11 | 4.885429 | 1.65E-19 | 7.55E-19 |
| CASQ2 | 2.028407 | 7.03E-11 | 1.41E-10 |
| VPS39 | 1.047647 | 2.11E-22 | 1.57E-21 |
| MDGA1 | 2.898812 | 2.78E-08 | 4.69E-08 |
| CFL1 | 1.150639 | 9.42E-25 | 1.33E-23 |
| RPS2 | 1.1146 | 3.59E-15 | 1.01E-14 |
| ST14 | 1.583798 | 0.000609 | 0.000773 |
| SOCS3 | -1.65666 | 4.00E-10 | 7.63E-10 |
| FBXO46 | 1.300942 | 7.47E-20 | 3.56E-19 |
| MED25 | 1.238268 | 7.68E-25 | 1.12E-23 |
| CYP2C19 | -3.01325 | 3.19E-17 | 1.11E-16 |
| SDHAF1 | 1.025195 | 3.72E-17 | 1.29E-16 |
| ZKSCAN5 | 1.284112 | 6.68E-27 | 2.52E-25 |
| VPS37D | 1.222774 | 6.73E-12 | 1.45E-11 |
| ZNHIT3 | 1.090619 | 6.12E-26 | 1.39E-24 |
| LOXL4 | 1.654037 | 0.000123 | 0.000163 |
| FOXS1 | 3.566372 | 5.08E-23 | 4.36E-22 |
| NOTCH3 | 2.780411 | 5.89E-23 | 4.97E-22 |
| ITGB1BP2 | 2.012046 | 7.50E-18 | 2.81E-17 |
| BAG6 | 1.060452 | 1.20E-24 | 1.64E-23 |
| STRC | 8.63862 | 2.88E-13 | 6.88E-13 |
| ZNF589 | 1.476558 | 2.25E-20 | 1.17E-19 |
| KRT86 | 2.80151 | 9.35E-07 | 1.43E-06 |
| PPIL2 | 1.042289 | 7.99E-20 | 3.80E-19 |
| SORT1 | 1.846919 | 3.08E-17 | 1.08E-16 |
| MXD3 | 3.337674 | 2.01E-29 | 1.44E-26 |
| ANAPC7 | 1.55204 | 4.63E-29 | 2.48E-26 |
| ZIC5 | 7.732515 | 1.26E-20 | 6.82E-20 |
| PPP1R14B | 1.459758 | 1.06E-20 | 5.82E-20 |
| ABRAXAS1 | 1.134303 | 1.78E-13 | 4.33E-13 |
| BRMS1 | 1.325872 | 2.80E-24 | 3.37E-23 |
| HYAL3 | 1.141723 | 1.01E-06 | 1.55E-06 |
| THEM5 | 2.378137 | 3.65E-23 | 3.25E-22 |
| GNAT1 | 2.065511 | 3.64E-10 | 6.95E-10 |
| TMEM198 | 2.076214 | 3.68E-19 | 1.60E-18 |
| TP53BP1 | 1.207814 | 2.57E-10 | 4.96E-10 |
| SCLY | 1.165947 | 1.86E-14 | 4.94E-14 |
| COG1 | 1.055327 | 1.57E-23 | 1.56E-22 |
| TRNP1 | 2.354202 | 0.000106 | 0.000141 |
| COMMD4 | 1.400051 | 1.83E-26 | 5.47E-25 |
| DRG1 | 1.02032 | 3.39E-24 | 3.97E-23 |
| XPOT | 1.250711 | 9.02E-19 | 3.73E-18 |
| CDCA7 | 4.503052 | 3.46E-16 | 1.08E-15 |
| MAST3 | 1.557571 | 2.20E-23 | 2.09E-22 |
| SMC3 | 1.04315 | 1.84E-12 | 4.13E-12 |
| TPR | 1.341724 | 9.80E-20 | 4.60E-19 |
| TLX1 | 3.47851 | 4.74E-16 | 1.45E-15 |
| CYB5RL | 1.638266 | 2.47E-20 | 1.27E-19 |
| HIST1H2AI | 4.389699 | 4.56E-17 | 1.56E-16 |
| DYRK2 | 1.348382 | 5.87E-14 | 1.49E-13 |
| TNFAIP2 | 1.017991 | 0.000104 | 0.000139 |
| FAM178B | 5.524525 | 0.000933 | 0.001172 |
| SAPCD1 | 3.539406 | 1.61E-23 | 1.59E-22 |
| OSBPL7 | 1.578769 | 5.61E-10 | 1.06E-09 |
| RFTN2 | 1.210807 | 1.05E-05 | 1.50E-05 |
| PCP2 | 1.509267 | 1.18E-07 | 1.90E-07 |
| PANX2 | 1.322401 | 0.005707 | 0.006782 |
| SLC22A11 | 4.806921 | 4.56E-06 | 6.65E-06 |
| RCOR2 | 3.249684 | 2.96E-06 | 4.38E-06 |
| IQCE | 1.846051 | 3.21E-25 | 5.38E-24 |
| AFAP1L1 | 1.195566 | 6.77E-15 | 1.85E-14 |
| HGF | -1.38493 | 3.52E-20 | 1.77E-19 |
| AKAP8L | 1.428924 | 4.42E-27 | 1.95E-25 |
| HSDL1 | 1.408065 | 1.65E-24 | 2.15E-23 |
| XPO1 | 1.111593 | 4.90E-22 | 3.42E-21 |
| KPNB1 | 1.102432 | 1.07E-19 | 5.00E-19 |
| PI4KB | 1.481844 | 1.67E-26 | 5.10E-25 |
| C11orf45 | 1.482852 | 2.93E-05 | 4.06E-05 |
| RALGAPB | 1.096889 | 1.78E-19 | 8.07E-19 |
| PILRB | 1.603811 | 1.11E-10 | 2.19E-10 |
| PIGZ | 1.70884 | 5.04E-17 | 1.71E-16 |
| ZBTB26 | 1.348528 | 2.54E-15 | 7.23E-15 |
| FBXO2 | 1.300176 | 0.007708 | 0.009067 |
| DOLK | 1.036632 | 8.77E-24 | 9.26E-23 |
| PPP1R3B | -1.03048 | 1.10E-11 | 2.32E-11 |
| TRIM56 | 1.064101 | 1.06E-19 | 4.94E-19 |
| USF1 | 1.454414 | 1.32E-28 | 3.21E-26 |
| HMGN4 | 1.311017 | 1.50E-15 | 4.38E-15 |
| TTC36 | -2.29954 | 4.52E-24 | 5.12E-23 |
| MAN1B1 | 1.033834 | 1.65E-23 | 1.63E-22 |
| ZNF552 | 1.359034 | 1.10E-19 | 5.10E-19 |
| HHIPL2 | 7.161748 | 1.12E-19 | 5.19E-19 |
| CCDC15 | 1.579895 | 1.09E-18 | 4.45E-18 |
| SEC61A1 | 1.063372 | 1.38E-22 | 1.08E-21 |
| KBTBD11 | -1.70373 | 1.81E-20 | 9.51E-20 |
| ZNF749 | 1.132523 | 1.15E-13 | 2.85E-13 |
| POLRMT | 1.090404 | 3.99E-24 | 4.58E-23 |
| MTFP1 | 1.489228 | 3.93E-19 | 1.70E-18 |
| CEP152 | 2.083005 | 1.23E-21 | 7.95E-21 |
| CDK5R1 | 2.0077 | 1.57E-17 | 5.64E-17 |
| IFFO2 | 1.184192 | 2.89E-11 | 5.96E-11 |
| CD244 | -1.06305 | 2.32E-13 | 5.59E-13 |
| ATP6V1E2 | 1.402659 | 2.92E-14 | 7.59E-14 |
| SLC12A7 | 1.396175 | 3.43E-23 | 3.08E-22 |
| HSD17B7 | 1.198149 | 2.15E-14 | 5.67E-14 |
| CCDC159 | 1.215839 | 3.60E-23 | 3.21E-22 |
| IRF3 | 1.360867 | 9.34E-24 | 9.80E-23 |
| HEY1 | 2.041161 | 9.46E-22 | 6.23E-21 |
| HMGXB3 | 1.530969 | 2.00E-27 | 1.16E-25 |
| RPL28 | 1.327839 | 1.12E-18 | 4.59E-18 |
| C17orf49 | 1.06364 | 1.26E-16 | 4.10E-16 |
| TAF10 | 1.230063 | 3.48E-26 | 9.04E-25 |
| ZNF2 | 1.185709 | 4.10E-25 | 6.53E-24 |
| DNASE1L2 | 3.217402 | 5.26E-18 | 2.00E-17 |
| UBN1 | 1.173857 | 7.86E-21 | 4.39E-20 |
| CRLF1 | 3.188206 | 1.02E-15 | 3.02E-15 |
| KLKB1 | -1.07175 | 5.73E-18 | 2.17E-17 |
| SYNGR2 | 1.193224 | 1.05E-21 | 6.88E-21 |
| TMEM266 | 2.711084 | 1.11E-18 | 4.54E-18 |
| ZNF468 | 1.7886 | 2.76E-07 | 4.37E-07 |
| HIST1H4C | 1.008244 | 5.19E-06 | 7.55E-06 |
| GLS | 1.793062 | 3.87E-12 | 8.48E-12 |
| TCTN2 | 1.421385 | 1.92E-06 | 2.86E-06 |
| HABP4 | 1.151073 | 1.51E-11 | 3.18E-11 |
| RAD54B | 2.324131 | 4.24E-26 | 1.05E-24 |
| SMG9 | 1.840789 | 3.18E-28 | 4.01E-26 |
| HIST1H3D | 2.854495 | 1.04E-16 | 3.43E-16 |
| TRMT12 | 1.079276 | 8.14E-21 | 4.53E-20 |
| TDP1 | 1.049777 | 5.19E-19 | 2.20E-18 |
| KISS1R | 5.004782 | 1.50E-07 | 2.41E-07 |
| DCAF16 | 1.490904 | 8.74E-17 | 2.90E-16 |
| PZP | -3.77305 | 2.27E-21 | 1.40E-20 |
| GPD1L | 1.534166 | 5.88E-09 | 1.04E-08 |
| DBNDD1 | 2.047228 | 1.91E-15 | 5.51E-15 |
| ADAM12 | 2.951967 | 6.36E-06 | 9.19E-06 |
| ZNF814 | 1.127183 | 1.90E-12 | 4.27E-12 |
| G6PC3 | 1.383004 | 3.39E-24 | 3.97E-23 |
| ATF6B | 1.267422 | 6.53E-22 | 4.45E-21 |
| UBOX5 | 1.051519 | 2.37E-23 | 2.23E-22 |
| GMEB2 | 1.308899 | 2.51E-26 | 6.93E-25 |
| HIST1H2BN | 2.186397 | 1.74E-19 | 7.91E-19 |
| KCNJ4 | 2.060393 | 0.000988 | 0.00124 |
| USP46 | 1.095235 | 1.85E-14 | 4.89E-14 |
| WDHD1 | 2.763232 | 1.33E-24 | 1.78E-23 |
| MEP1B | -1.35654 | 9.43E-15 | 2.55E-14 |
| AMER1 | 1.15817 | 1.73E-06 | 2.59E-06 |
| KLHL7 | 1.027699 | 7.47E-20 | 3.56E-19 |
| DEAF1 | 1.422069 | 3.47E-27 | 1.69E-25 |
| EFTUD2 | 1.196783 | 4.53E-26 | 1.10E-24 |
| CGREF1 | 2.547622 | 8.72E-15 | 2.36E-14 |
| TPM2 | 2.579094 | 1.48E-23 | 1.48E-22 |
| TCIRG1 | 1.348405 | 3.06E-16 | 9.57E-16 |
| ARHGAP19 | 1.218496 | 4.16E-19 | 1.79E-18 |
| KIAA0556 | 1.546719 | 2.75E-22 | 2.00E-21 |
| ZNF283 | 1.255268 | 7.06E-11 | 1.42E-10 |
| EIF3B | 1.24875 | 2.54E-26 | 6.97E-25 |
| RCN2 | 1.472379 | 3.46E-22 | 2.48E-21 |
| VEGFB | 1.318205 | 5.30E-05 | 7.22E-05 |
| CLDN18 | 5.292041 | 4.65E-06 | 6.78E-06 |
| ANKRD2 | 2.198273 | 0.000322 | 0.000416 |
| PDZK1IP1 | 3.438336 | 5.88E-05 | 7.98E-05 |
| ST5 | 1.057249 | 8.15E-10 | 1.52E-09 |
| SEMA6C | 2.129527 | 7.08E-21 | 3.99E-20 |
| ABCC1 | 1.751092 | 3.82E-06 | 5.60E-06 |
| C15orf48 | 2.925422 | 4.12E-07 | 6.46E-07 |
| CIB2 | 2.317655 | 4.83E-10 | 9.16E-10 |
| DCTN2 | 1.321558 | 2.52E-28 | 3.60E-26 |
| FBXW9 | 1.283174 | 2.24E-22 | 1.66E-21 |
| NRGN | 1.117377 | 1.77E-07 | 2.84E-07 |
| NEU1 | 1.822881 | 3.40E-28 | 4.05E-26 |
| ZMYND15 | 1.189334 | 7.66E-05 | 0.000103 |
| TMEM164 | 2.002107 | 1.15E-19 | 5.33E-19 |
| NPR2 | 1.198148 | 0.000471 | 0.000602 |
| XPR1 | 1.331334 | 5.43E-19 | 2.30E-18 |
| COMMD2 | 1.153176 | 2.53E-21 | 1.54E-20 |
| TBXA2R | -1.17964 | 2.61E-20 | 1.34E-19 |
| PCNA | 1.760906 | 3.47E-25 | 5.71E-24 |
| UBL7 | 1.123107 | 1.07E-24 | 1.49E-23 |
| HIST1H2BE | 2.354839 | 3.55E-14 | 9.17E-14 |
| RBP2 | 4.984714 | 4.18E-05 | 5.73E-05 |
| MAD2L1 | 2.930027 | 2.45E-25 | 4.32E-24 |
| ATP2B4 | 1.120474 | 1.40E-08 | 2.40E-08 |
| RPL38 | 1.439219 | 2.47E-21 | 1.51E-20 |
| STK3 | 1.107724 | 1.73E-20 | 9.13E-20 |
| NFKBIL1 | 1.399192 | 5.30E-25 | 8.18E-24 |
| GTF2E1 | 1.249613 | 1.85E-22 | 1.40E-21 |
| ZNF529 | 1.129107 | 4.20E-15 | 1.17E-14 |
| FRMD8 | 1.075161 | 7.34E-18 | 2.75E-17 |
| EPPK1 | 4.091428 | 9.95E-11 | 1.98E-10 |
| FRMD3 | 2.467998 | 1.17E-11 | 2.48E-11 |
| CTAG2 | 7.602987 | 1.36E-08 | 2.35E-08 |
| ELP1 | 1.039677 | 2.45E-10 | 4.74E-10 |
| RPTOR | 1.301991 | 4.43E-25 | 6.99E-24 |
| NUP62 | 1.19124 | 5.75E-20 | 2.79E-19 |
| S100A1 | 3.375022 | 7.33E-12 | 1.57E-11 |
| SLC39A1 | 1.424562 | 9.16E-26 | 1.91E-24 |
| STK36 | 1.324664 | 8.75E-20 | 4.14E-19 |
| TIMM17A | 1.047658 | 1.27E-19 | 5.87E-19 |
| PAGE4 | 8.88022 | 4.28E-07 | 6.70E-07 |
| SMIM32 | 5.431954 | 0.001211 | 0.001509 |
| CWC27 | 1.142977 | 7.68E-25 | 1.12E-23 |
| SPATA21 | 2.914464 | 3.42E-09 | 6.11E-09 |
| EXOG | 1.444491 | 1.20E-21 | 7.78E-21 |
| CCNA2 | 4.207815 | 3.92E-27 | 1.83E-25 |
| IFT172 | 1.310842 | 4.28E-20 | 2.11E-19 |
| PHF1 | 1.06433 | 8.65E-20 | 4.10E-19 |
| PKD1 | 1.30969 | 7.73E-19 | 3.22E-18 |
| PLLP | 1.013215 | 0.010914 | 0.012679 |
| HDAC5 | 1.221509 | 1.08E-21 | 7.04E-21 |
| ANGPT2 | 1.734857 | 2.11E-14 | 5.56E-14 |
| PDIA2 | 6.757734 | 1.94E-14 | 5.12E-14 |
| FAM86C1 | 1.238451 | 1.23E-23 | 1.25E-22 |
| LAMA4 | 2.19907 | 1.97E-25 | 3.58E-24 |
| RAP2A | 1.720433 | 8.53E-21 | 4.72E-20 |
| PPME1 | 1.146172 | 4.47E-26 | 1.09E-24 |
| C8B | -1.00773 | 2.58E-19 | 1.14E-18 |
| ADORA2A | 1.299588 | 1.40E-11 | 2.95E-11 |
| RNF43 | 1.774939 | 5.97E-08 | 9.86E-08 |
| ABCB6 | 1.040774 | 1.25E-10 | 2.47E-10 |
| MPZ | 3.534541 | 1.33E-10 | 2.62E-10 |
| EGF | 5.593952 | 1.10E-05 | 1.57E-05 |
| KIAA0232 | 1.040859 | 2.97E-18 | 1.15E-17 |
| ZBTB8OS | 1.014155 | 1.85E-22 | 1.40E-21 |
| RARG | 1.064021 | 1.02E-07 | 1.67E-07 |
| TOX2 | 1.28558 | 0.0005 | 0.000638 |
| RBBP4 | 1.085988 | 1.98E-18 | 7.85E-18 |
| GAREM2 | 2.925641 | 1.35E-14 | 3.59E-14 |
| LIN37 | 1.645653 | 1.23E-28 | 3.14E-26 |
| BICRA | 1.244494 | 5.04E-25 | 7.82E-24 |
| AUNIP | 1.738002 | 4.57E-11 | 9.31E-11 |
| MCUB | 1.22125 | 0.001859 | 0.00229 |
| PRR36 | 3.215461 | 1.54E-14 | 4.09E-14 |
| MB | 2.090026 | 0.011584 | 0.01344 |
| POC1A | 1.784875 | 1.03E-23 | 1.07E-22 |
| NOL7 | 1.301 | 1.02E-27 | 7.51E-26 |
| BUD31 | 1.083273 | 1.57E-24 | 2.06E-23 |
| ZSCAN26 | 1.189269 | 2.77E-17 | 9.73E-17 |
| RCOR3 | 1.399034 | 1.74E-22 | 1.33E-21 |
| HDAC11 | 2.401813 | 1.68E-27 | 1.05E-25 |
| COL5A3 | 1.911602 | 6.66E-10 | 1.25E-09 |
| LY96 | 1.578607 | 0.005837 | 0.00693 |
| DUSP12 | 1.605608 | 1.88E-26 | 5.58E-25 |
| ERFE | 3.434028 | 1.33E-24 | 1.78E-23 |
| SIRT6 | 1.273792 | 1.63E-23 | 1.61E-22 |
| RBM8A | 1.137481 | 1.03E-25 | 2.09E-24 |
| FAM200A | 1.263813 | 3.61E-25 | 5.88E-24 |
| FADS3 | 1.318341 | 5.62E-20 | 2.73E-19 |
| SOCS2 | -1.55983 | 1.17E-16 | 3.82E-16 |
| CCDC14 | 1.644612 | 1.53E-18 | 6.12E-18 |
| IQCB1 | 1.332306 | 2.58E-19 | 1.14E-18 |
| LUC7L | 1.138442 | 1.34E-18 | 5.41E-18 |
| 3-Mar | 1.583234 | 4.75E-09 | 8.41E-09 |
| OMP | 1.01441 | 0.015894 | 0.018271 |
| LYPD8 | 5.71819 | 7.81E-14 | 1.96E-13 |
| WASHC5 | 1.389248 | 3.39E-21 | 2.02E-20 |
| ME3 | 1.595171 | 3.70E-15 | 1.04E-14 |
| CEP290 | 1.431339 | 1.39E-16 | 4.52E-16 |
| PLPPR1 | 2.294629 | 1.33E-11 | 2.81E-11 |
| SEMA3G | 1.741113 | 3.52E-15 | 9.90E-15 |
| NCKIPSD | 1.19323 | 9.07E-25 | 1.29E-23 |
| ZNF16 | 1.453605 | 2.51E-26 | 6.93E-25 |
| PRKD2 | 1.192858 | 9.22E-24 | 9.70E-23 |
| REM1 | 1.217666 | 3.08E-06 | 4.56E-06 |
| ZSCAN12 | 1.212452 | 5.55E-13 | 1.30E-12 |
| LZTS1 | 2.068823 | 6.74E-17 | 2.26E-16 |
| PLIN2 | -1.02528 | 1.68E-12 | 3.79E-12 |
| TMEM255A | 1.758602 | 0.004674 | 0.005592 |
| LMNA | 1.462122 | 2.92E-23 | 2.68E-22 |
| SOX2 | 4.409603 | 0.000866 | 0.001091 |
| CRLF2 | 4.220902 | 1.05E-06 | 1.61E-06 |
| XRCC4 | 1.17157 | 1.53E-18 | 6.12E-18 |
| THOC6 | 1.128839 | 1.21E-21 | 7.86E-21 |
| NAV3 | 2.966925 | 1.21E-05 | 1.73E-05 |
| FSTL5 | 4.495642 | 0.025997 | 0.029479 |
| KIF7 | 1.916304 | 3.20E-11 | 6.58E-11 |
| NOX1 | 1.505125 | 9.70E-15 | 2.62E-14 |
| MRM1 | 1.308454 | 4.84E-23 | 4.17E-22 |
| CD151 | 1.355608 | 1.78E-16 | 5.71E-16 |
| NADSYN1 | 1.026926 | 1.07E-21 | 6.96E-21 |
| TOX3 | 1.020215 | 0.001606 | 0.001987 |
| ABL1 | 1.109302 | 1.31E-15 | 3.83E-15 |
| NREP | 1.410354 | 4.66E-08 | 7.73E-08 |
| THOC5 | 1.412982 | 6.20E-26 | 1.40E-24 |
| PTRH2 | 1.111026 | 5.67E-21 | 3.25E-20 |
| AGPAT1 | 1.320089 | 2.02E-24 | 2.55E-23 |
| HELLS | 3.198438 | 3.84E-24 | 4.44E-23 |
| PRMT1 | 1.214396 | 1.04E-21 | 6.81E-21 |
| FOXD2 | 2.794803 | 2.36E-19 | 1.05E-18 |
| FCGR2A | 1.140486 | 1.32E-05 | 1.87E-05 |
| CABYR | 4.878058 | 9.79E-16 | 2.90E-15 |
| ITGAE | 1.620539 | 3.57E-17 | 1.24E-16 |
| WNK4 | 4.331338 | 3.01E-15 | 8.50E-15 |
| ADCY4 | 1.206401 | 6.53E-13 | 1.52E-12 |
| CCL25 | 7.289122 | 1.67E-10 | 3.28E-10 |
| ATP8B2 | 1.319899 | 9.70E-08 | 1.58E-07 |
| GOLGA3 | 1.294123 | 3.18E-26 | 8.43E-25 |
| NRCAM | 3.079641 | 0.000107 | 0.000142 |
| BBS7 | 1.052114 | 2.23E-11 | 4.63E-11 |
| APOF | -2.37887 | 2.26E-26 | 6.50E-25 |
| EDC3 | 1.408117 | 2.20E-27 | 1.26E-25 |
| LIG1 | 1.84066 | 9.54E-25 | 1.34E-23 |
| SERPINB6 | 1.006754 | 3.53E-17 | 1.22E-16 |
| PTDSS2 | 1.324913 | 2.15E-23 | 2.04E-22 |
| ACTR5 | 1.310993 | 2.28E-23 | 2.15E-22 |
| SQSTM1 | 1.736409 | 9.02E-17 | 2.99E-16 |
| GPR153 | 1.089775 | 4.87E-05 | 6.65E-05 |
| ADM2 | 2.831467 | 6.47E-17 | 2.17E-16 |
| ZSCAN21 | 1.051455 | 5.74E-21 | 3.29E-20 |
| ATP5F1E | 1.104577 | 2.61E-20 | 1.34E-19 |
| GATD1 | 1.172589 | 7.91E-22 | 5.33E-21 |
| METTL4 | 1.023367 | 8.74E-17 | 2.90E-16 |
| FCN3 | -3.61352 | 1.09E-27 | 7.73E-26 |
| RPL23A | 1.309376 | 1.11E-19 | 5.16E-19 |
| EFNA3 | 2.783471 | 3.03E-22 | 2.19E-21 |
| CBX5 | 1.22137 | 1.27E-15 | 3.72E-15 |
| GLI4 | 2.172458 | 8.12E-28 | 6.45E-26 |
| TMA7 | 1.022726 | 3.72E-19 | 1.61E-18 |
| ATP6V1C1 | 1.649646 | 1.00E-24 | 1.41E-23 |
| MT1H | -2.39216 | 2.28E-23 | 2.15E-22 |
| STMND1 | 3.829458 | 8.47E-11 | 1.70E-10 |
| B3GNT5 | 2.381573 | 1.43E-10 | 2.82E-10 |
| SAA2-SAA4 | -1.80328 | 1.04E-05 | 1.48E-05 |
| TNPO2 | 1.20923 | 1.64E-25 | 3.10E-24 |
| FAM118A | 1.53353 | 5.98E-16 | 1.81E-15 |
| PRXL2B | 1.600509 | 8.65E-17 | 2.88E-16 |
| HSPH1 | 1.329905 | 2.55E-19 | 1.13E-18 |
| NT5C | 1.242622 | 8.44E-19 | 3.50E-18 |
| FAM122B | 1.460398 | 9.47E-21 | 5.21E-20 |
| KIF22 | 1.279689 | 1.10E-20 | 6.01E-20 |
| TMEM117 | 1.209642 | 3.96E-15 | 1.11E-14 |
| RAMP1 | 1.669132 | 4.82E-09 | 8.54E-09 |
| EID2B | 1.683528 | 3.33E-20 | 1.68E-19 |
| EBF1 | 2.826119 | 4.55E-25 | 7.14E-24 |
| NUDT14 | 1.330992 | 8.70E-13 | 2.01E-12 |
| RHNO1 | 1.846558 | 1.47E-24 | 1.95E-23 |
| NENF | 1.322306 | 5.37E-20 | 2.62E-19 |
| PRDM15 | 1.909588 | 3.87E-26 | 9.76E-25 |
| SUB1 | 1.317488 | 1.80E-26 | 5.43E-25 |
| EPB41L1 | 1.691953 | 0.008141 | 0.009559 |
| CCL26 | 3.790784 | 2.51E-09 | 4.53E-09 |
| STRBP | 1.120713 | 2.44E-21 | 1.49E-20 |
| FKBP9 | 1.203862 | 2.41E-19 | 1.07E-18 |
| ZNF761 | 1.548763 | 9.77E-13 | 2.24E-12 |
| IL11RA | 1.023728 | 3.34E-13 | 7.95E-13 |
| TBC1D31 | 2.036297 | 6.42E-27 | 2.47E-25 |
| MAGED2 | 1.402695 | 3.85E-25 | 6.21E-24 |
| SHBG | -1.2787 | 1.97E-14 | 5.21E-14 |
| MEX3B | 1.497222 | 1.27E-07 | 2.05E-07 |
| SRARP | 5.919684 | 0.000157 | 0.000207 |
| NOD1 | 1.027988 | 4.82E-14 | 1.23E-13 |
| ASPHD1 | 3.334377 | 0.000214 | 0.00028 |
| STRA8 | 4.612377 | 9.07E-12 | 1.93E-11 |
| MPV17L2 | 1.256349 | 6.11E-23 | 5.15E-22 |
| RAB29 | 1.019861 | 8.72E-15 | 2.36E-14 |
| TBCB | 1.297584 | 2.56E-22 | 1.87E-21 |
| CLN6 | 1.473331 | 1.28E-26 | 4.16E-25 |
| BCORL1 | 1.435949 | 6.45E-14 | 1.63E-13 |
| TAB1 | 1.004173 | 7.52E-23 | 6.17E-22 |
| LRRC69 | 2.525484 | 1.50E-19 | 6.90E-19 |
| MEGF8 | 1.386458 | 1.54E-19 | 7.04E-19 |
| FBXW8 | 1.047254 | 1.01E-17 | 3.73E-17 |
| RPL30 | 1.424228 | 3.86E-21 | 2.27E-20 |
| ITPA | 1.312939 | 2.62E-22 | 1.91E-21 |
| TYRP1 | 4.960195 | 0.000375 | 0.000483 |
| SPP2 | -1.09406 | 2.76E-14 | 7.19E-14 |
| CCDC163 | 2.164114 | 1.38E-22 | 1.08E-21 |
| TMEM106C | 2.162962 | 4.19E-26 | 1.04E-24 |
| ENO1 | 1.163949 | 1.92E-12 | 4.31E-12 |
| RAI1 | 1.089585 | 5.46E-09 | 9.65E-09 |
| NUDT1 | 2.464746 | 1.83E-26 | 5.47E-25 |
| RARRES1 | 2.226482 | 8.30E-06 | 1.19E-05 |
| MTR | 1.400688 | 2.77E-16 | 8.69E-16 |
| TRMT112 | 1.075764 | 5.02E-23 | 4.31E-22 |
| SART1 | 1.067764 | 3.44E-26 | 8.96E-25 |
| XXYLT1 | 1.588327 | 6.74E-20 | 3.24E-19 |
| PHF20L1 | 1.063482 | 5.14E-19 | 2.18E-18 |
| FAM104A | 1.026139 | 6.60E-21 | 3.74E-20 |
| SYNGAP1 | 1.072947 | 1.68E-08 | 2.88E-08 |
| ZNF436 | 1.227624 | 1.99E-19 | 8.96E-19 |
| ITLN2 | 2.382252 | 0.0048 | 0.005736 |
| CLN3 | 1.704354 | 7.73E-27 | 2.83E-25 |
| GSDMD | 1.262469 | 2.96E-22 | 2.14E-21 |
| FCGBP | 2.304765 | 0.002449 | 0.002995 |
| OFD1 | 1.279584 | 5.11E-21 | 2.96E-20 |
| TONSL | 2.645032 | 1.65E-28 | 3.39E-26 |
| CYP4A22 | -1.61582 | 1.17E-20 | 6.35E-20 |
| NOL11 | 1.018923 | 5.34E-23 | 4.56E-22 |
| CTSK | 2.796963 | 9.30E-07 | 1.42E-06 |
| CUL4B | 1.048723 | 1.22E-20 | 6.62E-20 |
| TEPSIN | 1.528959 | 1.05E-27 | 7.59E-26 |
| IFRD1 | 1.198855 | 1.65E-17 | 5.95E-17 |
| GINS2 | 2.47553 | 4.10E-22 | 2.90E-21 |
| C2orf92 | 1.625253 | 2.71E-17 | 9.53E-17 |
| NUP107 | 1.383674 | 1.01E-22 | 8.10E-22 |
| CDKN2B | 1.974919 | 4.38E-18 | 1.68E-17 |
| MAP1S | 1.070211 | 2.31E-18 | 9.08E-18 |
| RUFY1 | 1.155042 | 2.39E-25 | 4.22E-24 |
| GPR176 | 1.236426 | 2.72E-09 | 4.89E-09 |
| C2CD3 | 1.061204 | 2.85E-16 | 8.94E-16 |
| TUBG1 | 1.769563 | 2.51E-26 | 6.93E-25 |
| DPH2 | 1.446182 | 4.66E-25 | 7.30E-24 |
| CCDC142 | 1.63351 | 2.75E-25 | 4.74E-24 |
| PRAMEF4 | 5.960356 | 0.000327 | 0.000422 |
| CASTOR1 | 1.162784 | 2.66E-09 | 4.78E-09 |
| WDR60 | 1.037737 | 1.13E-14 | 3.03E-14 |
| CEP250 | 1.77538 | 1.85E-26 | 5.52E-25 |
| PHF21A | 1.262406 | 7.73E-19 | 3.22E-18 |
| TEKT5 | 2.094167 | 2.17E-08 | 3.69E-08 |
| ADGRG1 | 1.845539 | 8.66E-09 | 1.51E-08 |
| ZNF232 | 1.191057 | 3.11E-20 | 1.58E-19 |
| ZNF74 | 1.709822 | 2.27E-25 | 4.06E-24 |
| DKK2 | 2.418863 | 9.83E-06 | 1.41E-05 |
| ARL6IP6 | 1.238972 | 5.91E-12 | 1.28E-11 |
| CHGA | 8.060408 | 7.34E-13 | 1.70E-12 |
| MRNIP | 1.575178 | 3.95E-20 | 1.96E-19 |
| CALML6 | 2.385523 | 1.62E-14 | 4.30E-14 |
| CLDN5 | 1.053572 | 0.004286 | 0.005145 |
| EGLN3 | 2.213404 | 0.000555 | 0.000706 |
| ACTL6A | 1.303531 | 3.86E-20 | 1.92E-19 |
| RPS19 | 1.458705 | 4.25E-19 | 1.83E-18 |
| TMEM151A | 3.218275 | 0.000494 | 0.00063 |
| GALNT10 | 1.719143 | 2.38E-24 | 2.94E-23 |
| C6orf136 | 1.200284 | 5.73E-25 | 8.76E-24 |
| C19orf33 | 2.960933 | 0.023048 | 0.026228 |
| PABPC1 | 1.601711 | 3.64E-20 | 1.82E-19 |
| LIN7B | 1.2356 | 1.41E-16 | 4.56E-16 |
| PALM | 1.294304 | 0.032262 | 0.036368 |
| KDELR3 | 1.642308 | 3.11E-09 | 5.57E-09 |
| BCL2L14 | 1.663225 | 0.017162 | 0.019681 |
| SSB | 1.02676 | 2.47E-20 | 1.27E-19 |
| PDE6D | 1.055865 | 5.61E-23 | 4.77E-22 |
| CLASRP | 1.326442 | 3.43E-25 | 5.67E-24 |
| ZWINT | 3.200659 | 6.28E-26 | 1.41E-24 |
| ZNF316 | 1.181685 | 1.04E-23 | 1.08E-22 |
| CIZ1 | 1.373718 | 2.63E-24 | 3.19E-23 |
| ARL2 | 1.663956 | 2.72E-19 | 1.21E-18 |
| MINDY1 | 1.073398 | 1.54E-16 | 4.98E-16 |
| NQO1 | 5.311194 | 6.82E-16 | 2.06E-15 |
| PAPSS1 | 1.260899 | 7.56E-17 | 2.53E-16 |
| AOC2 | 1.171656 | 1.73E-09 | 3.15E-09 |
| FKBP14 | 1.1506 | 4.64E-14 | 1.19E-13 |
| GALNT18 | 1.399035 | 1.42E-07 | 2.29E-07 |
| ALDH8A1 | -1.34969 | 7.98E-25 | 1.15E-23 |
| NT5C3A | 1.099713 | 2.24E-17 | 7.96E-17 |
| RTN4RL2 | 1.427473 | 9.48E-07 | 1.45E-06 |
| FLAD1 | 1.656922 | 1.85E-29 | 1.44E-26 |
| ANXA11 | 1.022305 | 5.74E-19 | 2.42E-18 |
| TMEM79 | 1.758184 | 9.29E-28 | 7.16E-26 |
| AGAP1 | 1.585745 | 3.90E-21 | 2.30E-20 |
| BCAS4 | 2.374391 | 1.58E-17 | 5.70E-17 |
| NIN | 1.382032 | 1.47E-16 | 4.74E-16 |
| SCMH1 | 1.165138 | 2.97E-16 | 9.29E-16 |
| TSSK6 | 2.080947 | 3.34E-25 | 5.55E-24 |
| ADA | 1.502233 | 1.19E-17 | 4.35E-17 |
| ITGA6 | 2.059504 | 1.59E-23 | 1.57E-22 |
| SLC22A15 | 2.416763 | 3.35E-07 | 5.28E-07 |
| IL1R2 | 1.39536 | 3.45E-09 | 6.17E-09 |
| CNNM4 | 1.110406 | 1.83E-15 | 5.31E-15 |
| DEGS1 | 1.182576 | 6.59E-18 | 2.49E-17 |
| SEC13 | 1.021737 | 4.24E-26 | 1.05E-24 |
| KCNIP2 | 1.499944 | 1.32E-08 | 2.27E-08 |
| MYLIP | 1.103967 | 9.76E-08 | 1.59E-07 |
| ARHGEF16 | 1.65902 | 1.22E-05 | 1.74E-05 |
| NDUFA4L2 | 3.604664 | 2.02E-28 | 3.44E-26 |
| WHRN | 2.151606 | 1.56E-22 | 1.20E-21 |
| FAM49B | 1.528921 | 1.65E-19 | 7.51E-19 |
| RGL2 | 1.370558 | 3.77E-21 | 2.23E-20 |
| TESC | 3.397221 | 1.06E-05 | 1.51E-05 |
| CELSR2 | 1.103784 | 4.54E-07 | 7.09E-07 |
| TRIM71 | 9.256188 | 5.92E-16 | 1.80E-15 |
| FDPS | 1.427009 | 2.59E-16 | 8.18E-16 |
| INAFM1 | 1.104673 | 1.13E-13 | 2.80E-13 |
| IFT80 | 1.765255 | 3.26E-19 | 1.42E-18 |
| SSR4 | 1.009165 | 5.97E-15 | 1.64E-14 |
| TRIP10 | 1.283114 | 8.07E-15 | 2.19E-14 |
| EXOSC5 | 1.308643 | 6.57E-23 | 5.48E-22 |
| DCAF4 | 1.258399 | 9.95E-23 | 8.00E-22 |
| SUMO2 | 1.029261 | 8.60E-23 | 7.00E-22 |
| TTYH2 | 1.788568 | 3.97E-10 | 7.57E-10 |
| USP1 | 1.170925 | 6.89E-16 | 2.08E-15 |
| DDIT4 | 1.07143 | 6.49E-05 | 8.78E-05 |
| RASD2 | 3.329808 | 8.60E-27 | 3.05E-25 |
| CREB3L2 | 1.229772 | 2.66E-18 | 1.04E-17 |
| KIAA0513 | 1.286032 | 5.16E-15 | 1.42E-14 |
| LPCAT1 | 2.086581 | 1.78E-16 | 5.71E-16 |
| MNS1 | 3.266206 | 5.34E-14 | 1.36E-13 |
| PXYLP1 | 1.400906 | 1.32E-10 | 2.60E-10 |
| HTATIP2 | 1.195216 | 5.47E-15 | 1.51E-14 |
| MDFI | 3.09808 | 2.70E-12 | 6.01E-12 |
| IL1RL1 | -2.77083 | 2.86E-14 | 7.45E-14 |
| MYL9 | 1.570025 | 0.03461 | 0.038913 |
| GRN | 1.209499 | 9.80E-21 | 5.39E-20 |
| DOC2B | 1.466274 | 1.49E-06 | 2.25E-06 |
| SPTBN5 | 1.294159 | 8.60E-09 | 1.50E-08 |
| ACAD11 | -1.1017 | 7.76E-15 | 2.11E-14 |
| FAM222B | 1.005548 | 1.21E-15 | 3.55E-15 |
| TMPRSS9 | 1.62392 | 0.001367 | 0.001697 |
| TRIM25 | 1.483528 | 3.23E-21 | 1.94E-20 |
| MKKS | 1.171521 | 3.40E-20 | 1.71E-19 |
| ADAT2 | 1.508094 | 8.63E-19 | 3.57E-18 |
| BCL7A | 1.068107 | 5.76E-14 | 1.46E-13 |
| MAPK7 | 1.01045 | 2.03E-15 | 5.83E-15 |
| HGH1 | 1.679428 | 3.13E-25 | 5.28E-24 |
| CLSPN | 3.599726 | 4.73E-22 | 3.31E-21 |
| CSRP2 | 1.374538 | 1.70E-07 | 2.73E-07 |
| N4BP3 | 2.692399 | 1.13E-20 | 6.14E-20 |
| RTN2 | 1.913512 | 6.45E-11 | 1.30E-10 |
| PDGFA | 2.596397 | 3.44E-19 | 1.50E-18 |
| TGM3 | 5.963885 | 2.55E-22 | 1.86E-21 |
| RBM24 | 3.201836 | 1.50E-16 | 4.84E-16 |
| IGF2BP2 | 2.936124 | 1.22E-07 | 1.97E-07 |
| NAT8L | 4.279733 | 7.70E-06 | 1.11E-05 |
| UBAP2L | 1.696887 | 1.90E-26 | 5.63E-25 |
| CLEC16A | 1.143599 | 6.23E-20 | 3.01E-19 |
| ZNF207 | 1.174589 | 3.52E-24 | 4.12E-23 |
| KIF18B | 4.861427 | 7.58E-28 | 6.22E-26 |
| ALCAM | 1.037636 | 3.33E-14 | 8.63E-14 |
| AZGP1 | -1.06248 | 2.14E-18 | 8.44E-18 |
| HPS3 | 1.084203 | 2.23E-12 | 4.98E-12 |
| DPH7 | 1.608682 | 9.78E-26 | 2.02E-24 |
| C1orf74 | 1.479739 | 5.67E-21 | 3.25E-20 |
| ZNF26 | 1.324609 | 4.85E-20 | 2.38E-19 |
| PFDN6 | 1.671802 | 2.45E-27 | 1.35E-25 |
| HES6 | 2.085798 | 2.32E-24 | 2.88E-23 |
| WDR5 | 1.210397 | 8.35E-24 | 8.86E-23 |
| ZNF541 | 2.649349 | 1.01E-09 | 1.87E-09 |
| RPL35A | 1.145632 | 2.79E-19 | 1.23E-18 |
| SNTA1 | 1.296973 | 5.69E-16 | 1.73E-15 |
| C1orf216 | 1.199011 | 1.02E-16 | 3.37E-16 |
| TRIM50 | 6.099377 | 7.09E-07 | 1.09E-06 |
| MAZ | 1.479326 | 7.22E-20 | 3.45E-19 |
| CDKN2A | 4.65432 | 1.87E-25 | 3.46E-24 |
| E2F7 | 4.285471 | 2.60E-24 | 3.17E-23 |
| ZYX | 1.078749 | 8.78E-13 | 2.02E-12 |
| ODR4 | 1.231666 | 7.07E-23 | 5.86E-22 |
| RFXANK | 1.721051 | 1.25E-26 | 4.10E-25 |
| TEAD3 | 1.274568 | 2.58E-20 | 1.33E-19 |
| KAAG1 | 2.549843 | 3.15E-05 | 4.35E-05 |
| SLC35E2B | 1.187821 | 1.84E-13 | 4.49E-13 |
| FAM161B | 1.050654 | 2.26E-15 | 6.46E-15 |
| HEXD | 1.038337 | 3.11E-17 | 1.09E-16 |
| GADD45GIP1 | 1.126993 | 6.48E-16 | 1.96E-15 |
| SSX1 | 9.636572 | 8.83E-11 | 1.76E-10 |
| MATN3 | 6.004678 | 6.75E-14 | 1.70E-13 |
| ARV1 | 1.097655 | 1.73E-21 | 1.09E-20 |
| PCGF1 | 1.244781 | 1.76E-28 | 3.39E-26 |
| SYMPK | 1.075535 | 7.46E-24 | 8.04E-23 |
| GGA1 | 1.135563 | 4.87E-24 | 5.46E-23 |
| RILPL1 | 1.158709 | 2.14E-18 | 8.44E-18 |
| OLFML2A | 3.07882 | 5.51E-25 | 8.49E-24 |
| ICA1 | 1.06412 | 0.003443 | 0.004167 |
| ITPKA | 3.254963 | 5.55E-18 | 2.10E-17 |
| SRSF12 | 1.910398 | 1.67E-08 | 2.86E-08 |
| THEM4 | 1.307889 | 1.04E-24 | 1.45E-23 |
| ZNF28 | 1.701938 | 2.67E-06 | 3.96E-06 |
| PHLDB1 | 1.146475 | 0.000625 | 0.000793 |
| TMEM69 | 1.043899 | 1.56E-20 | 8.29E-20 |
| INPPL1 | 1.307307 | 4.82E-21 | 2.80E-20 |
| APOBEC3B | 2.463004 | 1.11E-11 | 2.36E-11 |
| CBLL1 | 1.012991 | 1.00E-19 | 4.69E-19 |
| TRAPPC9 | 1.114586 | 1.47E-21 | 9.37E-21 |
| TNFRSF9 | 2.560296 | 0.000211 | 0.000275 |
| CPEB3 | -1.67136 | 9.00E-24 | 9.47E-23 |
| AVIL | 2.627962 | 4.80E-12 | 1.04E-11 |
| PIAS4 | 1.001046 | 6.46E-22 | 4.40E-21 |
| PDCD11 | 1.188425 | 9.95E-23 | 8.00E-22 |
| SMG5 | 2.005931 | 2.80E-27 | 1.45E-25 |
| C16orf74 | 1.858701 | 1.63E-05 | 2.30E-05 |
| GLA | 1.809361 | 1.20E-24 | 1.64E-23 |
| KIF20B | 1.756433 | 2.04E-16 | 6.49E-16 |
| VNN2 | 2.349832 | 0.00289 | 0.003516 |
| DDIT3 | 1.506553 | 3.18E-17 | 1.11E-16 |
| FTSJ1 | 1.208885 | 2.52E-25 | 4.41E-24 |
| HES4 | 1.889875 | 1.32E-13 | 3.26E-13 |
| COPS6 | 1.103571 | 3.39E-26 | 8.88E-25 |
| RNF181 | 1.016172 | 2.50E-20 | 1.29E-19 |
| MORC2 | 1.256905 | 3.66E-25 | 5.93E-24 |
| TP73 | 4.102942 | 2.96E-23 | 2.71E-22 |
| BEST1 | 1.505088 | 5.03E-07 | 7.83E-07 |
| NPM2 | 2.462039 | 3.16E-06 | 4.66E-06 |
| DUS4L | 1.253081 | 2.68E-25 | 4.66E-24 |
| HNRNPA3 | 1.015295 | 7.82E-22 | 5.27E-21 |
| MTMR11 | 2.524995 | 5.98E-16 | 1.81E-15 |
| RNF215 | 1.415167 | 1.64E-27 | 1.03E-25 |
| ENO3 | -1.08504 | 1.05E-13 | 2.61E-13 |
| PARS2 | 1.034851 | 8.65E-20 | 4.10E-19 |
| NME1 | 1.785041 | 4.09E-24 | 4.68E-23 |
| WASHC1 | 1.383698 | 3.48E-20 | 1.75E-19 |
| KLHDC3 | 1.100658 | 6.85E-22 | 4.66E-21 |
| BCS1L | 1.050793 | 7.21E-25 | 1.07E-23 |
| SFI1 | 1.94991 | 3.86E-22 | 2.75E-21 |
| STMN3 | 1.63436 | 0.004455 | 0.005339 |
| NDUFB9 | 1.308406 | 5.07E-20 | 2.48E-19 |
| USP14 | 1.138276 | 2.04E-23 | 1.96E-22 |
| LARS | 1.356923 | 5.33E-28 | 5.01E-26 |
| IPO13 | 1.114931 | 2.32E-24 | 2.88E-23 |
| TFAP4 | 1.318831 | 2.56E-20 | 1.32E-19 |
| MICALL1 | 1.169702 | 3.82E-20 | 1.90E-19 |
| WDR91 | 1.403828 | 1.86E-19 | 8.42E-19 |
| MARCKS | 1.353735 | 3.04E-10 | 5.83E-10 |
| KDM1A | 1.000926 | 5.94E-22 | 4.08E-21 |
| PRAF2 | 1.228657 | 1.43E-18 | 5.76E-18 |
| IKBIP | 1.275049 | 2.68E-17 | 9.44E-17 |
| MDK | 4.34479 | 1.00E-24 | 1.41E-23 |
| KITLG | 1.899602 | 3.08E-12 | 6.82E-12 |
| MCTS1 | 1.028037 | 4.84E-23 | 4.17E-22 |
| ESPL1 | 2.174154 | 1.47E-14 | 3.90E-14 |
| ARHGAP18 | 1.042685 | 4.71E-09 | 8.35E-09 |
| SEMA7A | 1.848927 | 9.40E-12 | 2.00E-11 |
| ZNF555 | 1.53347 | 4.49E-23 | 3.89E-22 |
| ACACA | 1.491746 | 6.37E-20 | 3.08E-19 |
| UBD | 3.043178 | 4.29E-18 | 1.64E-17 |
| TMEM14A | 1.134734 | 3.12E-14 | 8.10E-14 |
| CDR2L | 1.281265 | 0.012464 | 0.014424 |
| CD99L2 | 1.023655 | 1.96E-14 | 5.17E-14 |
| ZNF551 | 1.051401 | 6.29E-07 | 9.74E-07 |
| NPEPPS | 1.058856 | 2.07E-15 | 5.94E-15 |
| SPIN2B | 1.005637 | 6.29E-16 | 1.90E-15 |
| EMILIN2 | 1.471256 | 1.67E-07 | 2.68E-07 |
| RHOC | 1.208629 | 3.72E-19 | 1.61E-18 |
| C17orf75 | 1.046024 | 1.43E-22 | 1.12E-21 |
| PSME3 | 1.169085 | 7.10E-24 | 7.72E-23 |
| ZNF446 | 1.223608 | 7.61E-23 | 6.24E-22 |
| ZNF432 | 1.483876 | 5.65E-17 | 1.91E-16 |
| HJURP | 4.669989 | 6.71E-28 | 5.68E-26 |
| ANKRD29 | 2.283936 | 1.50E-14 | 3.98E-14 |
| EMC2 | 1.220633 | 4.52E-24 | 5.12E-23 |
| MOGAT3 | 1.667227 | 2.31E-07 | 3.67E-07 |
| NOCT | -1.13304 | 7.79E-11 | 1.56E-10 |
| DNAJB5 | 1.024113 | 5.97E-15 | 1.64E-14 |
| SAA1 | -1.59235 | 2.16E-06 | 3.22E-06 |
| TUBE1 | -1.19662 | 8.33E-21 | 4.63E-20 |
| FRRS1L | 6.003718 | 8.00E-08 | 1.31E-07 |
| URB2 | 1.111436 | 3.95E-14 | 1.02E-13 |
| CTLA4 | 1.714908 | 6.29E-06 | 9.09E-06 |
| FBL | 1.557455 | 8.92E-23 | 7.25E-22 |
| ABCA4 | 1.857412 | 3.29E-10 | 6.29E-10 |
| CHRNA1 | 3.377505 | 0.001289 | 0.001603 |
| SNAPC2 | 1.168367 | 1.52E-23 | 1.51E-22 |
| ESR1 | -1.94173 | 1.04E-22 | 8.36E-22 |
| MTMR3 | 1.199314 | 1.84E-16 | 5.88E-16 |
| CHCHD7 | 1.098854 | 4.40E-19 | 1.89E-18 |
| WASHC2A | 1.370649 | 9.82E-24 | 1.02E-22 |
| PUS7L | 1.077453 | 1.97E-16 | 6.30E-16 |
| SSX2IP | 1.322603 | 2.01E-08 | 3.42E-08 |
| NME7 | 1.081943 | 4.98E-17 | 1.69E-16 |
| BCL9L | 1.360549 | 2.81E-07 | 4.45E-07 |
| U2SURP | 1.171457 | 4.45E-19 | 1.91E-18 |
| KCTD17 | 2.348266 | 5.74E-10 | 1.08E-09 |
| TMEM237 | 1.579905 | 1.00E-19 | 4.69E-19 |
| TMEM160 | 1.211131 | 5.74E-15 | 1.58E-14 |
| TLCD1 | 2.231977 | 1.02E-24 | 1.42E-23 |
| OSR2 | 4.8656 | 2.66E-13 | 6.38E-13 |
| ZNF669 | 1.522388 | 1.28E-14 | 3.43E-14 |
| PIGG | 1.133639 | 2.41E-22 | 1.77E-21 |
| H2AFY | 1.32349 | 8.14E-24 | 8.67E-23 |
| UBE2O | 1.415124 | 1.02E-27 | 7.51E-26 |
| E2F5 | 1.899621 | 2.75E-19 | 1.22E-18 |
| C17orf113 | 1.26516 | 2.80E-05 | 3.89E-05 |
| PNCK | 6.783361 | 4.66E-09 | 8.26E-09 |
| RAB3B | 4.329747 | 8.00E-18 | 2.98E-17 |
| H1F0 | 1.367939 | 1.00E-19 | 4.69E-19 |
| RPL39 | 1.212185 | 6.01E-17 | 2.03E-16 |
| NECTIN1 | 1.963544 | 1.41E-19 | 6.46E-19 |
| SASS6 | 1.804105 | 4.85E-20 | 2.38E-19 |
| CCT3 | 1.810898 | 5.25E-29 | 2.50E-26 |
| CLHC1 | 1.288024 | 8.07E-14 | 2.02E-13 |
| TEAD4 | 1.464149 | 2.02E-07 | 3.23E-07 |
| DNAJC9 | 1.539731 | 3.03E-20 | 1.54E-19 |
| ZMYM1 | 1.100003 | 5.76E-14 | 1.46E-13 |
| CHFR | 1.045604 | 1.06E-14 | 2.84E-14 |
| FAM168B | 1.185424 | 9.96E-19 | 4.10E-18 |
| IER5 | 1.224294 | 9.60E-15 | 2.59E-14 |
| LSM7 | 1.249753 | 8.05E-21 | 4.48E-20 |
| CTNNA2 | 6.254786 | 1.87E-06 | 2.80E-06 |
| CCNB1 | 4.027226 | 2.73E-28 | 3.82E-26 |
| ZNF781 | 2.287973 | 5.79E-07 | 8.98E-07 |
| MSS51 | 1.948376 | 6.53E-21 | 3.71E-20 |
| CCDC7 | 1.129369 | 6.48E-16 | 1.96E-15 |
| CYP4F2 | -1.03355 | 6.21E-15 | 1.70E-14 |
| CHD7 | 1.145013 | 2.61E-12 | 5.81E-12 |
| PGC | 11.17373 | 2.87E-12 | 6.36E-12 |
| UGT2B7 | -1.28143 | 6.96E-18 | 2.62E-17 |
| NMB | 2.85231 | 1.27E-25 | 2.51E-24 |
| CCL15 | 1.1611 | 7.47E-13 | 1.73E-12 |
| FN3KRP | 1.049964 | 3.24E-17 | 1.13E-16 |
| FIBP | 1.181234 | 1.12E-26 | 3.76E-25 |
| TYK2 | 1.028319 | 2.85E-23 | 2.63E-22 |
| MAMSTR | 2.752481 | 2.02E-24 | 2.55E-23 |
| SLC4A11 | 3.606901 | 1.51E-10 | 2.96E-10 |
| SFR1 | 1.206572 | 2.98E-17 | 1.04E-16 |
| GPI | 1.021478 | 1.93E-16 | 6.18E-16 |
| HSPB8 | 2.022237 | 0.009573 | 0.011179 |
| FAM53B | 1.602342 | 5.02E-20 | 2.46E-19 |
| CLEC2L | 7.594794 | 4.76E-08 | 7.89E-08 |
| SNRPD1 | 1.399362 | 1.38E-22 | 1.08E-21 |
| GXYLT2 | 3.213584 | 1.11E-06 | 1.69E-06 |
| PKP4 | 1.038131 | 3.85E-19 | 1.67E-18 |
| SLC45A1 | 1.586369 | 2.23E-16 | 7.09E-16 |
| ALDH3A1 | 5.871452 | 0.000343 | 0.000443 |
| TAP1 | 1.544189 | 6.51E-15 | 1.78E-14 |
| NHP2 | 1.410458 | 2.51E-26 | 6.93E-25 |
| WDYHV1 | 1.76921 | 3.34E-24 | 3.94E-23 |
| TMEM14C | 1.070031 | 3.47E-25 | 5.71E-24 |
| RAB11FIP3 | 1.036485 | 9.19E-18 | 3.41E-17 |
| KCNQ4 | 1.639477 | 0.028591 | 0.032327 |
| PLVAP | 3.480185 | 8.94E-30 | 1.28E-26 |
| HHIP | -2.67259 | 3.63E-26 | 9.34E-25 |
| TM4SF5 | 1.249442 | 0.000184 | 0.000242 |
| TK1 | 3.048702 | 5.30E-26 | 1.25E-24 |
| SLC12A8 | 1.23411 | 3.24E-08 | 5.44E-08 |
| RELL2 | 1.996899 | 3.47E-18 | 1.34E-17 |
| FUT2 | 3.691166 | 4.89E-16 | 1.49E-15 |
| NDUFAF8 | 1.181299 | 1.13E-14 | 3.03E-14 |
| RCCD1 | 1.429251 | 5.30E-26 | 1.25E-24 |
| NIPSNAP2 | 1.414596 | 3.15E-19 | 1.38E-18 |
| WDR88 | 1.738783 | 5.83E-17 | 1.97E-16 |
| AXIN1 | 1.041836 | 1.48E-14 | 3.94E-14 |
| CD46 | 1.12532 | 1.83E-17 | 6.57E-17 |
| MIF4GD | 1.244075 | 6.08E-22 | 4.17E-21 |
| RAB23 | 1.233865 | 9.57E-08 | 1.56E-07 |
| MPP3 | 2.668993 | 5.11E-15 | 1.41E-14 |
| PHF6 | 1.25299 | 5.83E-17 | 1.97E-16 |
| SLC46A1 | 1.188519 | 2.66E-18 | 1.04E-17 |
| HSPA14 | 1.175473 | 2.59E-22 | 1.89E-21 |
| ATG10 | 1.022617 | 1.11E-22 | 8.85E-22 |
| PLEKHG4 | 3.072188 | 3.18E-08 | 5.33E-08 |
| CCDC28B | 2.964913 | 2.63E-24 | 3.19E-23 |
| FAP | 3.060851 | 1.97E-12 | 4.42E-12 |
| IRF2BP2 | 1.136232 | 5.46E-22 | 3.77E-21 |
| TEF | 1.032211 | 2.59E-11 | 5.36E-11 |
| EMX1 | 4.562804 | 4.54E-10 | 8.62E-10 |
| ZNF813 | 1.737446 | 2.93E-05 | 4.07E-05 |
| MTMR2 | 1.204606 | 2.99E-11 | 6.16E-11 |
| CD4 | -1.2519 | 1.84E-18 | 7.31E-18 |
| B4GALT3 | 1.430231 | 2.07E-25 | 3.75E-24 |
| RNF220 | 1.140478 | 2.48E-25 | 4.37E-24 |
| ITGB5 | 1.01861 | 1.97E-15 | 5.67E-15 |
| GLS2 | -1.41924 | 1.24E-15 | 3.66E-15 |
| GNB1L | 1.658374 | 5.74E-24 | 6.35E-23 |
| SLC6A9 | 2.815957 | 4.04E-15 | 1.13E-14 |
| BAG2 | 1.409729 | 1.76E-16 | 5.66E-16 |
| ABCF2 | 1.349716 | 2.75E-26 | 7.42E-25 |
| AACS | 1.728119 | 1.45E-19 | 6.68E-19 |
| FAM13A | -1.23676 | 8.48E-17 | 2.82E-16 |
| ANKMY1 | 1.156449 | 1.23E-23 | 1.25E-22 |
| NUP43 | 1.240184 | 1.43E-22 | 1.12E-21 |
| CIART | 2.152522 | 1.71E-15 | 4.96E-15 |
| FBXO5 | 1.004251 | 0.000305 | 0.000395 |
| CSNK1G1 | 1.225223 | 1.87E-20 | 9.82E-20 |
| CSAG1 | 5.115011 | 1.67E-09 | 3.06E-09 |
| ZNF391 | 2.216381 | 0.007964 | 0.009359 |
| ERICH5 | 2.218128 | 3.86E-07 | 6.06E-07 |
| TMEM201 | 2.018071 | 6.33E-27 | 2.45E-25 |
| ZNF419 | 1.172431 | 9.43E-13 | 2.17E-12 |
| MAMLD1 | 1.354553 | 0.023876 | 0.027136 |
| DTNA | 2.242328 | 9.06E-15 | 2.45E-14 |
| DUOX1 | 3.117501 | 5.88E-20 | 2.85E-19 |
| DEPDC1 | 4.588703 | 9.69E-27 | 3.33E-25 |
| NRARP | 1.575493 | 3.36E-11 | 6.90E-11 |
| SH2B2 | 1.512505 | 1.60E-15 | 4.64E-15 |
| TBX6 | 1.438136 | 8.42E-16 | 2.51E-15 |
| HNRNPUL2 | 1.024366 | 1.65E-23 | 1.63E-22 |
| PLCH2 | 2.692034 | 0.008879 | 0.010398 |
| NBEAL2 | 1.262998 | 1.26E-13 | 3.11E-13 |
| IGDCC3 | 6.912105 | 1.14E-10 | 2.27E-10 |
| SIRPG | 1.311286 | 0.00281 | 0.003423 |
| SLC38A2 | -1.01427 | 1.82E-12 | 4.09E-12 |
| PTOV1 | 1.020749 | 9.25E-23 | 7.47E-22 |
| DTYMK | 2.001067 | 4.78E-28 | 4.77E-26 |
| PEG10 | 5.270011 | 0.009641 | 0.011255 |
| DBF4 | 1.636104 | 2.71E-21 | 1.65E-20 |
| ADH4 | -1.81934 | 8.53E-21 | 4.72E-20 |
| PHLDA1 | -1.83777 | 5.14E-16 | 1.57E-15 |
| C19orf25 | 1.47028 | 2.24E-25 | 4.02E-24 |
| SAA2 | -1.86361 | 1.92E-06 | 2.86E-06 |
| ANKRD49 | 1.041939 | 2.01E-20 | 1.05E-19 |
| FBXO32 | 2.344026 | 2.17E-18 | 8.53E-18 |
| CALR | 1.016986 | 1.05E-19 | 4.89E-19 |
| RABL6 | 1.467575 | 2.28E-23 | 2.15E-22 |
| HIP1R | 1.186579 | 6.55E-16 | 1.98E-15 |
| NCCRP1 | 2.71086 | 0.006575 | 0.007775 |
| ESCO2 | 2.365951 | 2.30E-16 | 7.30E-16 |
| BCAM | 1.730171 | 2.15E-12 | 4.81E-12 |
| ZNF117 | 1.540723 | 4.10E-11 | 8.38E-11 |
| HSF2 | 1.292878 | 2.69E-18 | 1.05E-17 |
| TIPIN | 1.415957 | 1.89E-22 | 1.43E-21 |
| CASP3 | 1.011805 | 1.98E-17 | 7.05E-17 |
| PCDHB13 | 1.471186 | 0.035568 | 0.039959 |
| C19orf54 | 1.196249 | 7.94E-24 | 8.47E-23 |
| ASH1L | 1.209691 | 1.22E-12 | 2.78E-12 |
| FOXRED2 | 1.404098 | 1.30E-14 | 3.46E-14 |
| BAMBI | 1.233233 | 0.000178 | 0.000234 |
| STXBP5 | 1.036746 | 8.13E-08 | 1.33E-07 |
| CDK18 | 1.170942 | 0.002879 | 0.003503 |
| PI3 | 2.587422 | 0.000522 | 0.000666 |
| RBL1 | 2.007747 | 2.87E-20 | 1.46E-19 |
| MLLT11 | 2.023823 | 6.63E-06 | 9.56E-06 |
| ZP3 | 2.24388 | 1.22E-16 | 3.98E-16 |
| TMEM178A | 1.470059 | 9.09E-06 | 1.30E-05 |
| PRKCI | 1.000904 | 2.43E-12 | 5.43E-12 |
| PLEKHH1 | 2.092632 | 5.67E-19 | 2.40E-18 |
| COL8A1 | 2.38687 | 8.80E-10 | 1.64E-09 |
| HSF4 | 2.433977 | 1.61E-24 | 2.11E-23 |
| CHML | 2.465216 | 5.02E-19 | 2.14E-18 |
| RBBP7 | 1.211599 | 3.05E-25 | 5.18E-24 |
| LAMB1 | 1.43517 | 1.48E-06 | 2.24E-06 |
| ABHD17C | 1.409815 | 1.74E-13 | 4.25E-13 |
| LPCAT2 | 1.403846 | 0.000235 | 0.000306 |
| TWIST1 | 2.93422 | 0.003579 | 0.004326 |
| VARS | 1.421776 | 4.49E-25 | 7.06E-24 |
| DBNDD2 | 1.695367 | 1.33E-11 | 2.81E-11 |
| TCTEX1D2 | 1.651148 | 4.82E-15 | 1.34E-14 |
| VSTM4 | 1.399606 | 5.41E-05 | 7.37E-05 |
| BANF1 | 1.163276 | 7.84E-26 | 1.68E-24 |
| SYTL5 | -1.01106 | 9.28E-14 | 2.32E-13 |
| FLYWCH2 | 1.419728 | 7.03E-17 | 2.36E-16 |
| CSTA | 1.231536 | 0.010315 | 0.012013 |
| CYP3A4 | -1.15293 | 1.69E-15 | 4.92E-15 |
| NPFFR2 | 6.986521 | 6.45E-08 | 1.06E-07 |
| ZNF202 | 1.034651 | 1.27E-17 | 4.62E-17 |
| MIEN1 | 1.156854 | 1.53E-24 | 2.02E-23 |
| HHATL | 6.967955 | 0.005924 | 0.007031 |
| SLC35F6 | 1.252357 | 6.90E-23 | 5.73E-22 |
| DOHH | 1.040579 | 7.00E-21 | 3.95E-20 |
| ABHD12 | 1.398442 | 1.92E-25 | 3.52E-24 |
| DGKH | 1.532768 | 3.75E-16 | 1.16E-15 |
| ALG3 | 1.248504 | 2.11E-26 | 6.15E-25 |
| PGBD1 | 1.702578 | 1.45E-14 | 3.87E-14 |
| PPIB | 1.103034 | 2.53E-24 | 3.10E-23 |
| RPS27 | 1.276456 | 1.38E-18 | 5.59E-18 |
| RND3 | -1.84927 | 7.42E-21 | 4.16E-20 |
| RRP36 | 1.207798 | 6.04E-26 | 1.38E-24 |
| COL21A1 | 2.19031 | 6.19E-09 | 1.09E-08 |
| POLR1C | 1.058794 | 2.33E-21 | 1.43E-20 |
| SLC12A9 | 1.283136 | 7.00E-21 | 3.95E-20 |
| ACSL4 | 2.655243 | 3.57E-12 | 7.87E-12 |
| DDIAS | 2.315305 | 2.56E-21 | 1.56E-20 |
| ERMP1 | 1.377864 | 4.24E-16 | 1.31E-15 |
| RNASE1 | 1.480272 | 1.93E-09 | 3.52E-09 |
| MANF | 1.620934 | 6.18E-23 | 5.20E-22 |
| GMDS | 1.073375 | 3.91E-10 | 7.45E-10 |
| HIST1H1E | 2.129532 | 6.15E-14 | 1.56E-13 |
| MYBL1 | 1.930574 | 5.31E-17 | 1.80E-16 |
| UXS1 | 1.603448 | 1.67E-26 | 5.10E-25 |
| USP36 | 1.080953 | 5.08E-19 | 2.16E-18 |
| HDGFL2 | 1.343096 | 6.59E-27 | 2.52E-25 |
| RAMAC | 1.125913 | 1.92E-24 | 2.45E-23 |
| ATXN7L3 | 1.429399 | 1.21E-23 | 1.23E-22 |
| CXCR3 | 1.189497 | 2.78E-05 | 3.86E-05 |
| ITM2A | 1.272021 | 0.00189 | 0.002327 |
| EIF3E | 1.266633 | 4.46E-16 | 1.37E-15 |
| PRKDC | 1.514472 | 2.87E-20 | 1.46E-19 |
| EFNA1 | 1.424491 | 2.68E-17 | 9.44E-17 |
| TPX2 | 2.913901 | 1.93E-21 | 1.20E-20 |
| C5orf30 | 2.449307 | 1.42E-07 | 2.29E-07 |
| GPS1 | 1.177244 | 1.26E-26 | 4.15E-25 |
| PQLC2 | 1.102905 | 6.55E-19 | 2.75E-18 |
| KRI1 | 1.205277 | 6.53E-26 | 1.46E-24 |
| SAMM50 | 1.153612 | 5.14E-23 | 4.40E-22 |
| PLEKHG2 | 1.822492 | 2.88E-21 | 1.74E-20 |
| EDRF1 | 1.034861 | 2.30E-16 | 7.30E-16 |
| NRBP2 | 1.547138 | 6.82E-14 | 1.72E-13 |
| STEAP4 | -1.09324 | 1.38E-17 | 5.01E-17 |
| VIL1 | 1.433432 | 0.010098 | 0.011768 |
| AL159163.1 | 1.161715 | 4.21E-05 | 5.77E-05 |
| MECOM | 1.946663 | 8.22E-14 | 2.06E-13 |
| TMSB10 | 1.961784 | 5.91E-12 | 1.28E-11 |
| BEND3 | 1.908888 | 2.17E-18 | 8.53E-18 |
| VAT1 | 1.44382 | 1.17E-17 | 4.30E-17 |
| BRD8 | 1.331907 | 1.85E-24 | 2.37E-23 |
| PHLDB3 | 1.009642 | 5.04E-18 | 1.92E-17 |
| APLN | 4.622208 | 1.08E-28 | 2.88E-26 |
| GNL1 | 1.013797 | 1.64E-22 | 1.26E-21 |
| CYB561D1 | 1.881225 | 3.68E-21 | 2.18E-20 |
| FAM43A | 1.015606 | 0.000112 | 0.000149 |
| HIF1AN | 1.288387 | 6.34E-23 | 5.31E-22 |
| PRIM2 | 1.88723 | 4.42E-27 | 1.95E-25 |
| TKT | 2.146212 | 5.82E-20 | 2.82E-19 |
| GNS | 1.024769 | 3.56E-19 | 1.55E-18 |
| ASPA | -1.25673 | 1.83E-17 | 6.57E-17 |
| RPLP1 | 1.285263 | 3.43E-21 | 2.05E-20 |
| CDC25C | 5.028265 | 2.62E-28 | 3.71E-26 |
| MTHFD1L | 2.17854 | 2.35E-22 | 1.73E-21 |
| RNASEH2A | 2.573036 | 7.48E-28 | 6.17E-26 |
| UNC45A | 1.188491 | 1.13E-24 | 1.55E-23 |
| TMEM189 | 1.157409 | 2.03E-18 | 8.01E-18 |
| PYCR3 | 1.630597 | 9.42E-25 | 1.33E-23 |
| OLFM2 | 1.074364 | 2.93E-05 | 4.07E-05 |
| ZNF618 | 1.764819 | 2.01E-20 | 1.05E-19 |
| USP49 | 1.965496 | 5.93E-27 | 2.38E-25 |
| BGLAP | 2.418008 | 4.44E-21 | 2.59E-20 |
| METTL6 | 1.462141 | 6.71E-28 | 5.68E-26 |
| ZNF43 | 2.079944 | 8.02E-08 | 1.31E-07 |
| 6-Sep | 1.076862 | 1.19E-10 | 2.36E-10 |
| CETN2 | 1.249398 | 1.65E-24 | 2.15E-23 |
| FMNL2 | 1.292266 | 0.033062 | 0.037238 |
| ATP10A | 2.120533 | 0.000358 | 0.000462 |
| DNASE1 | 1.764154 | 1.19E-22 | 9.47E-22 |
| ENTPD2 | 2.400722 | 3.97E-12 | 8.69E-12 |
| FAM219B | 1.291359 | 1.55E-17 | 5.59E-17 |
| TEDC2 | 3.489824 | 6.00E-27 | 2.39E-25 |
| CUL9 | 1.508855 | 2.14E-27 | 1.24E-25 |
| PGF | 1.843288 | 3.66E-13 | 8.68E-13 |
| S100A3 | 2.547818 | 1.34E-17 | 4.88E-17 |
| METTL18 | 1.523727 | 4.61E-25 | 7.23E-24 |
| TBX4 | 8.875693 | 7.18E-16 | 2.16E-15 |
| EPDR1 | 1.229474 | 2.86E-08 | 4.81E-08 |
| CDAN1 | 1.320969 | 5.95E-25 | 9.06E-24 |
| TMEM136 | 2.03554 | 9.59E-16 | 2.85E-15 |
| FKBP1A | 1.164278 | 2.89E-23 | 2.66E-22 |
| KIFC2 | 2.635961 | 3.18E-24 | 3.76E-23 |
| HAUS6 | 1.039914 | 8.88E-11 | 1.77E-10 |
| ZBED8 | 1.972357 | 1.29E-23 | 1.30E-22 |
| PRR7 | 1.889333 | 5.39E-14 | 1.37E-13 |
| RBM34 | 1.268398 | 3.20E-21 | 1.92E-20 |
| ADM5 | 1.164642 | 6.06E-12 | 1.31E-11 |
| IQCH | 2.112949 | 0.00059 | 0.000749 |
| RAMP2 | 1.138802 | 2.35E-10 | 4.56E-10 |
| NCF2 | 1.054888 | 0.011789 | 0.013665 |
| DCN | -1.6672 | 1.56E-19 | 7.11E-19 |
| LMNB2 | 2.087141 | 6.76E-25 | 1.01E-23 |
| ZNF93 | 1.938997 | 3.74E-07 | 5.87E-07 |
| ZNF746 | 1.240633 | 7.42E-21 | 4.16E-20 |
| AVL9 | 1.16076 | 9.80E-21 | 5.39E-20 |
| CTHRC1 | 4.695255 | 1.84E-21 | 1.15E-20 |
| ALB | -1.27141 | 6.53E-21 | 3.71E-20 |
| SYNGR3 | 2.631361 | 3.52E-05 | 4.86E-05 |
| C4orf48 | 2.566856 | 1.35E-10 | 2.66E-10 |
| APEX2 | 1.014667 | 7.45E-22 | 5.04E-21 |
| SNAI2 | 1.73855 | 1.00E-06 | 1.53E-06 |
| RGP1 | 1.23012 | 1.19E-20 | 6.48E-20 |
| NDE1 | 1.312635 | 4.64E-14 | 1.19E-13 |
| BCAS1 | 3.195358 | 5.52E-05 | 7.51E-05 |
| WDR4 | 1.59301 | 7.59E-25 | 1.11E-23 |
| C19orf81 | 5.531304 | 4.06E-08 | 6.78E-08 |
| SNRNP70 | 1.446005 | 5.73E-26 | 1.33E-24 |
| SLC25A35 | 1.448558 | 3.93E-19 | 1.70E-18 |
| WSCD1 | 2.088568 | 3.45E-09 | 6.17E-09 |
| UBQLN4 | 1.579128 | 2.39E-25 | 4.22E-24 |
| DAZAP1 | 1.012234 | 6.94E-25 | 1.03E-23 |
| CASP2 | 1.530935 | 8.14E-21 | 4.53E-20 |
| SOX4 | 2.015579 | 3.10E-07 | 4.89E-07 |
| ZDHHC13 | 1.424834 | 7.35E-05 | 9.91E-05 |
| HLA-G | 1.163789 | 1.18E-07 | 1.91E-07 |
| PLK4 | 2.909274 | 3.38E-25 | 5.61E-24 |
| ZMIZ2 | 1.395028 | 3.14E-24 | 3.73E-23 |
| PTPRM | 1.11079 | 5.47E-06 | 7.94E-06 |
| TLE6 | 1.876416 | 9.08E-10 | 1.69E-09 |
| MT1X | -2.0435 | 2.81E-21 | 1.70E-20 |
| CNOT6 | 1.284007 | 1.04E-18 | 4.27E-18 |
| HOXB7 | 2.419392 | 3.55E-09 | 6.35E-09 |
| CPED1 | -1.42297 | 1.78E-19 | 8.07E-19 |
| PLAC9 | 1.623418 | 0.001088 | 0.00136 |
| MPC2 | 1.038414 | 1.58E-21 | 1.00E-20 |
| LINGO1 | 3.273077 | 1.61E-15 | 4.68E-15 |
| WASHC2C | 1.155436 | 7.72E-22 | 5.21E-21 |
| MAGOHB | 1.0746 | 2.09E-22 | 1.55E-21 |
| C14orf180 | -2.11595 | 4.78E-26 | 1.15E-24 |
| SPTSSA | 1.077408 | 3.80E-12 | 8.34E-12 |
| PIP4P2 | 1.173574 | 8.75E-05 | 0.000117 |
| BOD1 | 1.195011 | 2.83E-25 | 4.86E-24 |
| DNAJC5 | 1.411833 | 4.69E-24 | 5.29E-23 |
| BROX | 1.265143 | 4.35E-24 | 4.95E-23 |
| VPS16 | 1.147215 | 6.17E-27 | 2.42E-25 |
| RPS19BP1 | 1.129268 | 1.90E-21 | 1.19E-20 |
| TOMM40 | 1.524813 | 4.96E-26 | 1.19E-24 |
| C1orf35 | 1.974158 | 1.58E-28 | 3.39E-26 |
| CNOT3 | 1.245562 | 1.52E-25 | 2.89E-24 |
| SYCE2 | 1.340071 | 5.45E-14 | 1.39E-13 |
| DNAL4 | 1.161661 | 9.80E-22 | 6.45E-21 |
| ACADSB | -1.13915 | 1.46E-18 | 5.87E-18 |
| ZNF396 | 1.137924 | 2.67E-15 | 7.58E-15 |
| CDH11 | 2.262516 | 3.37E-06 | 4.97E-06 |
| LYG1 | 1.765102 | 3.26E-19 | 1.42E-18 |
| SNF8 | 1.231231 | 4.59E-26 | 1.11E-24 |
| TRAF7 | 1.177411 | 2.13E-19 | 9.55E-19 |
| C5orf34 | 3.129868 | 5.70E-28 | 5.13E-26 |
| PRAME | 7.70997 | 3.39E-06 | 4.99E-06 |
| PTCD1 | 1.418485 | 2.35E-27 | 1.32E-25 |
| NOL4L | 1.089294 | 3.93E-11 | 8.05E-11 |
| MAP7D2 | 4.327599 | 0.007184 | 0.00847 |
| NPIPB15 | 1.352541 | 8.05E-07 | 1.24E-06 |
| TMEM209 | 1.160079 | 2.91E-19 | 1.28E-18 |
| ITGAM | 1.125947 | 0.018833 | 0.021549 |
| DEFB132 | 3.187069 | 0.002488 | 0.003042 |
| UBALD2 | 1.189539 | 8.62E-14 | 2.16E-13 |
| PYCARD | 1.595545 | 7.65E-06 | 1.10E-05 |
| ZNF648 | 2.787341 | 0.000105 | 0.00014 |
| MASP1 | -1.0353 | 8.75E-20 | 4.14E-19 |
| ADAM23 | 3.857918 | 2.05E-06 | 3.06E-06 |
| EOGT | 1.052098 | 4.08E-15 | 1.14E-14 |
| OSBP2 | 2.482173 | 2.40E-15 | 6.84E-15 |
| PTTG1 | 4.40059 | 1.45E-27 | 9.53E-26 |
| COLCA2 | 3.563313 | 3.21E-20 | 1.63E-19 |
| SNX22 | 2.505742 | 1.46E-18 | 5.87E-18 |
| DRD4 | 2.792546 | 1.06E-14 | 2.84E-14 |
| RGS5 | 2.091593 | 1.51E-18 | 6.06E-18 |
| STAB2 | -4.30849 | 2.35E-28 | 3.60E-26 |
| PIGM | 1.120262 | 6.99E-23 | 5.79E-22 |
| PRRC2C | 1.124066 | 5.80E-15 | 1.59E-14 |
| ETFDH | -1.0909 | 8.20E-22 | 5.49E-21 |
| CRIP3 | 2.815211 | 7.54E-22 | 5.10E-21 |
| HECTD2 | 1.312134 | 4.29E-12 | 9.37E-12 |
| ZNF692 | 2.329829 | 1.38E-28 | 3.25E-26 |
| SLC6A8 | 3.652698 | 7.21E-13 | 1.67E-12 |
| SEMA6B | 1.0524 | 8.41E-12 | 1.79E-11 |
| PLEKHM2 | 1.037708 | 6.20E-19 | 2.61E-18 |
| SMTN | 1.098819 | 3.02E-13 | 7.22E-13 |
| SH3D21 | 1.892879 | 8.13E-17 | 2.71E-16 |
| POLQ | 3.781529 | 3.17E-25 | 5.33E-24 |
| CDH13 | 2.974222 | 2.97E-28 | 3.94E-26 |
| TCTE3 | 1.571015 | 4.88E-17 | 1.66E-16 |
| MICA | 1.430866 | 1.49E-20 | 7.96E-20 |
| PLCB3 | 1.23498 | 1.86E-21 | 1.16E-20 |
| ST6GALNAC2 | 1.732204 | 4.05E-05 | 5.56E-05 |
| TULP4 | 1.10163 | 8.93E-17 | 2.96E-16 |
| CDKAL1 | 1.066976 | 2.63E-19 | 1.17E-18 |
| KLHL23 | 1.735896 | 5.61E-18 | 2.13E-17 |
| IRF5 | 1.245307 | 4.35E-17 | 1.49E-16 |
| UBE3D | 1.05713 | 4.30E-14 | 1.10E-13 |
| PRKD1 | 1.468218 | 0.001408 | 0.001746 |
| IQCC | 2.16277 | 6.59E-27 | 2.52E-25 |
| CDC7 | 2.788124 | 3.18E-24 | 3.76E-23 |
| RPL10L | 6.185699 | 8.79E-07 | 1.35E-06 |
| ZNF726 | 1.988234 | 3.12E-07 | 4.92E-07 |
| MANBAL | 1.350101 | 5.86E-28 | 5.20E-26 |
| ZNF449 | 1.386399 | 1.02E-21 | 6.66E-21 |
| B4GALNT1 | 4.897101 | 1.50E-21 | 9.58E-21 |
| ASXL1 | 1.334943 | 1.26E-23 | 1.27E-22 |
| SUN1 | 1.156465 | 8.53E-18 | 3.17E-17 |
| ACTN4 | 1.02434 | 3.85E-18 | 1.48E-17 |
| KDM3B | 1.066491 | 1.06E-16 | 3.50E-16 |
| GRK6 | 1.444472 | 7.30E-25 | 1.07E-23 |
| C17orf58 | 1.357599 | 6.18E-25 | 9.38E-24 |
| E4F1 | 1.392443 | 3.43E-27 | 1.68E-25 |
| DPT | -1.39632 | 1.35E-16 | 4.39E-16 |
| NDUFA7 | 1.042141 | 1.96E-17 | 6.98E-17 |
| CUL7 | 1.82554 | 6.77E-27 | 2.55E-25 |
| DAB2IP | 1.341244 | 6.51E-14 | 1.64E-13 |
| DDX11 | 1.996299 | 1.31E-18 | 5.30E-18 |
| EFCAB13 | 1.118028 | 2.05E-07 | 3.27E-07 |
| KCNU1 | 8.198613 | 1.42E-05 | 2.01E-05 |
| TMEM200A | 1.704026 | 0.000151 | 0.000199 |
| FLYWCH1 | 1.285548 | 1.22E-22 | 9.69E-22 |
| MTCH1 | 1.129395 | 3.02E-24 | 3.61E-23 |
| PNISR | 1.010317 | 2.55E-11 | 5.28E-11 |
| LYAR | 1.006605 | 3.83E-16 | 1.19E-15 |
| POLH | 1.075348 | 1.15E-13 | 2.85E-13 |
| SLC6A14 | 6.481254 | 0.000519 | 0.000662 |
| TRIM65 | 1.72496 | 2.30E-25 | 4.08E-24 |
| ZNF182 | 1.061116 | 1.19E-18 | 4.83E-18 |
| CCDC107 | 1.425416 | 1.13E-24 | 1.55E-23 |
| PPP1R35 | 1.691919 | 5.30E-26 | 1.25E-24 |
| NFKB2 | 1.290095 | 7.34E-18 | 2.75E-17 |
| PCED1A | 1.494031 | 1.15E-23 | 1.18E-22 |
| ITGA2 | 2.722479 | 2.68E-14 | 6.99E-14 |
| SRPK1 | 1.243733 | 6.48E-19 | 2.72E-18 |
| ATP6V1B1 | 2.723246 | 2.15E-11 | 4.47E-11 |
| RNPEPL1 | 1.212281 | 1.07E-23 | 1.10E-22 |
| FANCM | 1.35107 | 1.86E-18 | 7.38E-18 |
| PMS2 | 1.099448 | 8.17E-13 | 1.89E-12 |
| TMC7 | 2.849972 | 7.43E-23 | 6.10E-22 |
| ZNF554 | 1.062079 | 1.61E-19 | 7.35E-19 |
| ZFYVE26 | 1.259923 | 5.61E-21 | 3.22E-20 |
| CXCL14 | -2.74119 | 1.09E-26 | 3.67E-25 |
| TTC9 | 2.335849 | 4.07E-12 | 8.91E-12 |
| GEMIN7 | 1.260463 | 6.03E-24 | 6.64E-23 |
| BMP7 | 4.736981 | 0.00017 | 0.000224 |
| S100A6 | 1.840727 | 7.24E-05 | 9.76E-05 |
| ASB16 | 2.785326 | 4.44E-23 | 3.85E-22 |
| TACC2 | 1.332869 | 6.90E-20 | 3.31E-19 |
| FBF1 | 2.125684 | 6.36E-26 | 1.43E-24 |
| TP53BP2 | 1.155112 | 4.76E-13 | 1.12E-12 |
| NR4A2 | -1.24568 | 1.64E-10 | 3.22E-10 |
| XRCC3 | 2.339118 | 1.01E-27 | 7.50E-26 |
| YWHAH | 1.038608 | 6.81E-18 | 2.56E-17 |
| CARNS1 | 1.878986 | 0.00832 | 0.009765 |
| CMSS1 | 1.283392 | 5.10E-25 | 7.89E-24 |
| RAB3IP | 1.062293 | 2.78E-09 | 5.00E-09 |
| PLEKHA8 | 1.34385 | 1.06E-20 | 5.82E-20 |
| KCNH6 | 5.61725 | 1.33E-06 | 2.01E-06 |
| DONSON | 1.664713 | 1.08E-23 | 1.11E-22 |
| ATN1 | 1.223608 | 1.02E-18 | 4.18E-18 |
| ADAMTS12 | 1.400702 | 0.00091 | 0.001144 |
| PROZ | -1.06966 | 1.13E-13 | 2.80E-13 |
| HMGB2 | 2.105357 | 7.01E-24 | 7.63E-23 |
| RUVBL1 | 1.287289 | 6.88E-26 | 1.52E-24 |
| ERBB3 | 1.443135 | 1.65E-21 | 1.04E-20 |
| SLC29A2 | 1.646388 | 7.25E-17 | 2.43E-16 |
| LRRC45 | 1.606009 | 2.58E-20 | 1.33E-19 |
| IL27RA | 1.220625 | 3.50E-09 | 6.26E-09 |
| GCN1 | 1.198504 | 3.98E-23 | 3.49E-22 |
| ADCY6 | 1.869475 | 3.47E-25 | 5.71E-24 |
| NOS2 | 2.049054 | 5.21E-13 | 1.22E-12 |
| TEN1-CDK3 | 1.305832 | 2.80E-11 | 5.77E-11 |
| SRCIN1 | 1.07985 | 1.49E-07 | 2.40E-07 |
| BMP4 | 2.426482 | 3.34E-08 | 5.59E-08 |
| ANKAR | 1.346247 | 6.01E-22 | 4.13E-21 |
| SMAD2 | 1.05422 | 5.27E-22 | 3.65E-21 |
| B3GALNT2 | 1.321046 | 1.50E-12 | 3.39E-12 |
| KCTD15 | 1.057428 | 0.008847 | 0.010362 |
| CDCA4 | 2.145944 | 5.44E-26 | 1.28E-24 |
| ALKBH6 | 1.872166 | 2.29E-26 | 6.54E-25 |
| TMED3 | 1.90838 | 0.000184 | 0.000242 |
| REPIN1 | 1.420278 | 6.88E-26 | 1.52E-24 |
| ARHGEF3 | 1.495408 | 8.73E-09 | 1.52E-08 |
| SINHCAF | 1.145252 | 0.00062 | 0.000787 |
| TPRN | 1.312712 | 5.52E-12 | 1.19E-11 |
| CSTF2 | 1.583139 | 7.19E-24 | 7.80E-23 |
| ANAPC11 | 1.362086 | 3.88E-23 | 3.41E-22 |
| MOSPD2 | 1.000953 | 7.63E-14 | 1.92E-13 |
| FKTN | 1.037988 | 1.91E-15 | 5.51E-15 |
| PPT2 | 1.04778 | 3.70E-10 | 7.06E-10 |
| THOC3 | 1.211973 | 6.38E-22 | 4.36E-21 |
| ZNF266 | 1.221011 | 1.30E-10 | 2.56E-10 |
| ZNF628 | 1.222023 | 2.12E-23 | 2.02E-22 |
| MAP6 | 1.287058 | 0.004336 | 0.005203 |
| PPP1R3F | 1.167146 | 1.46E-09 | 2.68E-09 |
| SPNS1 | 1.315871 | 2.15E-14 | 5.67E-14 |
| UBR5 | 1.330169 | 1.81E-20 | 9.51E-20 |
| C7 | -1.48802 | 7.10E-17 | 2.38E-16 |
| CENPT | 1.287002 | 8.93E-26 | 1.87E-24 |
| TUFT1 | 1.554554 | 4.35E-17 | 1.49E-16 |
| CHST10 | 1.958818 | 3.43E-06 | 5.05E-06 |
| HEYL | 1.540099 | 4.50E-05 | 6.16E-05 |
| DHX57 | 1.475929 | 1.09E-26 | 3.67E-25 |
| MFSD13A | 1.371823 | 4.43E-20 | 2.18E-19 |
| TWNK | 1.367242 | 5.25E-19 | 2.23E-18 |
| CYC1 | 1.275336 | 8.60E-23 | 7.00E-22 |
| FBN3 | 4.035503 | 2.57E-08 | 4.34E-08 |
| PAGR1 | 1.048881 | 8.08E-20 | 3.84E-19 |
| C6orf48 | 1.892177 | 3.75E-25 | 6.07E-24 |
| PHTF2 | 1.185157 | 9.69E-16 | 2.87E-15 |
| RAN | 1.209038 | 1.83E-22 | 1.39E-21 |
| LRRC37B | 1.436224 | 6.74E-18 | 2.54E-17 |
| ZSCAN9 | 1.664804 | 1.97E-25 | 3.58E-24 |
| ENAH | 2.277571 | 2.31E-23 | 2.18E-22 |
| FKBPL | 1.538837 | 1.02E-25 | 2.07E-24 |
| NDRG1 | 1.903125 | 7.08E-12 | 1.52E-11 |
| CASK | 1.218994 | 5.59E-17 | 1.89E-16 |
| GATA2 | 1.116715 | 3.29E-05 | 4.54E-05 |
| FMNL3 | 1.38324 | 4.41E-15 | 1.23E-14 |
| CNTNAP4 | 5.310765 | 0.028635 | 0.032374 |
| ORAI2 | 1.157752 | 3.01E-05 | 4.18E-05 |
| MRO | -1.69639 | 6.67E-20 | 3.21E-19 |
| ZNF563 | 1.496169 | 3.18E-19 | 1.39E-18 |
| NFRKB | 1.252037 | 6.18E-23 | 5.20E-22 |
| LASP1 | 1.219699 | 9.47E-21 | 5.21E-20 |
| SQLE | 2.291146 | 5.04E-18 | 1.92E-17 |
| CFAP53 | 2.05825 | 8.92E-19 | 3.69E-18 |
| DARS2 | 1.513332 | 1.21E-23 | 1.23E-22 |
| KYAT1 | 1.05578 | 3.13E-16 | 9.76E-16 |
| DTX3 | 1.29831 | 0.000263 | 0.000342 |
| TRAF5 | 2.19483 | 6.92E-21 | 3.91E-20 |
| DSN1 | 1.951011 | 3.71E-27 | 1.78E-25 |
| FANCF | 1.149789 | 7.34E-23 | 6.04E-22 |
| RPL9 | 1.017727 | 1.66E-13 | 4.07E-13 |
| PCDH17 | 2.002626 | 2.33E-19 | 1.04E-18 |
| LAD1 | 1.696933 | 2.63E-05 | 3.66E-05 |
| RNF157 | 3.218546 | 4.31E-17 | 1.48E-16 |
| TRIB1 | -1.38312 | 1.84E-19 | 8.33E-19 |
| SSC4D | 2.271563 | 2.36E-13 | 5.69E-13 |
| VSIG4 | -1.19752 | 2.49E-13 | 6.00E-13 |
| GDAP1 | 1.721845 | 4.76E-11 | 9.68E-11 |
| AKR1B15 | 4.337253 | 6.01E-09 | 1.06E-08 |
| BCAP31 | 1.444989 | 1.07E-25 | 2.16E-24 |
| EIF2B4 | 1.046174 | 1.32E-25 | 2.58E-24 |
| OIT3 | -2.67068 | 7.53E-27 | 2.78E-25 |
| TSNARE1 | 1.414165 | 3.35E-21 | 2.00E-20 |
| PPP2R5B | 1.047507 | 1.82E-21 | 1.14E-20 |
| TMEM147 | 1.468213 | 1.11E-22 | 8.85E-22 |
| GPX7 | 1.532409 | 0.000302 | 0.000391 |
| RASGEF1B | -1.21536 | 8.22E-17 | 2.74E-16 |
| SLC2A5 | 4.953349 | 2.44E-12 | 5.45E-12 |
| RPS10 | 1.40684 | 1.84E-19 | 8.33E-19 |
| FBLL1 | 2.002283 | 0.002393 | 0.002929 |
| DYNC1I1 | 4.420425 | 2.21E-10 | 4.29E-10 |
| GFI1 | 1.359576 | 0.000175 | 0.000229 |
| PIP4K2C | 1.018264 | 9.69E-18 | 3.58E-17 |
| ANO9 | 2.414916 | 7.58E-05 | 0.000102 |
| MAPKAPK2 | 1.216592 | 8.53E-21 | 4.72E-20 |
| SEZ6 | 7.664058 | 2.54E-12 | 5.66E-12 |
| ZNF785 | 1.496542 | 8.29E-25 | 1.18E-23 |
| TBC1D30 | 1.386962 | 0.000185 | 0.000243 |
| LPGAT1 | 1.404082 | 2.17E-17 | 7.72E-17 |
| PTGES | 3.428041 | 0.014798 | 0.017038 |
| LAMC1 | 2.118244 | 1.87E-23 | 1.82E-22 |
| C19orf48 | 1.999521 | 1.63E-24 | 2.13E-23 |
| TCF19 | 3.497909 | 1.37E-26 | 4.37E-25 |
| HK1 | 1.008103 | 0.004998 | 0.005966 |
| TATDN1 | 1.379636 | 1.33E-24 | 1.78E-23 |
| ZNF124 | 1.33188 | 3.23E-09 | 5.77E-09 |
| HAUS5 | 1.705794 | 1.15E-27 | 8.02E-26 |
| SMYD3 | 2.188125 | 4.05E-25 | 6.47E-24 |
| NEDD8 | 1.000631 | 5.10E-25 | 7.89E-24 |
| TMPO | 1.014966 | 4.59E-13 | 1.08E-12 |
| DPYSL3 | 1.150484 | 0.00927 | 0.010834 |
| BCR | 1.013695 | 3.40E-19 | 1.48E-18 |
| QSOX2 | 1.224852 | 3.99E-20 | 1.98E-19 |
| NUPL2 | 1.239248 | 1.11E-25 | 2.24E-24 |
| PPP1R1B | 4.647164 | 3.17E-06 | 4.68E-06 |
| DMXL2 | 1.007095 | 1.23E-10 | 2.43E-10 |
| MED15 | 1.122227 | 5.94E-21 | 3.40E-20 |
| CDH24 | 2.910761 | 5.47E-27 | 2.28E-25 |
| LMCD1 | 1.040085 | 4.00E-11 | 8.18E-11 |
| CELSR1 | 1.489289 | 2.58E-06 | 3.83E-06 |
| SSR2 | 1.844638 | 8.87E-29 | 2.81E-26 |
| REXO4 | 1.214924 | 7.30E-25 | 1.07E-23 |
| CDKL5 | 1.070612 | 4.78E-07 | 7.46E-07 |
| C1orf109 | 1.078744 | 2.47E-16 | 7.82E-16 |
| AP3B1 | 1.25896 | 2.80E-24 | 3.37E-23 |
| NCAPH2 | 1.538793 | 1.20E-25 | 2.39E-24 |
| DUSP15 | 2.324414 | 0.002045 | 0.002513 |
| HSPBAP1 | 1.070907 | 2.84E-18 | 1.11E-17 |
| EMCN | 1.155126 | 2.65E-10 | 5.12E-10 |
| RIPK2 | 1.263241 | 5.33E-12 | 1.15E-11 |
| NPNT | 2.598312 | 0.000107 | 0.000143 |
| PIGX | 1.061654 | 1.19E-22 | 9.47E-22 |
| AIF1L | 2.190642 | 7.60E-13 | 1.76E-12 |
| FLVCR1 | 2.863303 | 2.14E-28 | 3.44E-26 |
| ZNF229 | 1.73256 | 0.034505 | 0.038805 |
| TUBA1A | 1.202837 | 4.50E-08 | 7.48E-08 |
| NCBP2 | 1.097896 | 4.66E-23 | 4.03E-22 |
| NUAK1 | 1.576915 | 2.94E-18 | 1.14E-17 |
| JSRP1 | 1.959519 | 0.000294 | 0.000381 |
| BAX | 1.527077 | 7.52E-23 | 6.17E-22 |
| WDR92 | 1.181808 | 5.35E-21 | 3.09E-20 |
| QSER1 | 1.047275 | 2.25E-13 | 5.44E-13 |
| NUDT2 | 1.232556 | 4.35E-19 | 1.87E-18 |
| TBC1D22A | 1.070047 | 1.27E-21 | 8.22E-21 |
| THSD7A | 1.408781 | 3.96E-06 | 5.80E-06 |
| MCMDC2 | 1.884157 | 1.81E-20 | 9.51E-20 |
| AGRN | 1.646343 | 1.97E-15 | 5.67E-15 |
| ITGA7 | 1.170018 | 4.31E-13 | 1.02E-12 |
| DAXX | 1.282463 | 1.47E-24 | 1.95E-23 |
| RPP40 | 1.289531 | 6.08E-21 | 3.47E-20 |
| UBE2C | 4.87396 | 7.90E-28 | 6.39E-26 |
| MAFG | 1.977408 | 7.43E-23 | 6.10E-22 |
| XRCC1 | 1.455723 | 2.35E-27 | 1.32E-25 |
| ZFC3H1 | 1.252991 | 1.14E-16 | 3.75E-16 |
| MUS81 | 1.200978 | 1.39E-26 | 4.39E-25 |
| JPT1 | 1.952607 | 9.00E-24 | 9.47E-23 |
| NPC2 | 1.048889 | 1.89E-15 | 5.47E-15 |
| SMAGP | 1.453923 | 2.72E-15 | 7.73E-15 |
| ZNF676 | 3.592539 | 0.041389 | 0.046277 |
| ADAMTS10 | 1.647983 | 1.02E-09 | 1.89E-09 |
| PDCL3 | 1.19818 | 1.99E-24 | 2.53E-23 |
| GAS2L3 | 1.928301 | 3.92E-14 | 1.01E-13 |
| MYBPHL | 6.197583 | 2.32E-12 | 5.19E-12 |
| HSD17B3 | 1.757559 | 7.82E-09 | 1.37E-08 |
| LOX | 2.345371 | 9.00E-12 | 1.92E-11 |
| CPE | 1.982562 | 3.68E-11 | 7.55E-11 |
| GOLM1 | 1.924597 | 2.67E-10 | 5.15E-10 |
| TRIM52 | 1.4228 | 9.46E-22 | 6.23E-21 |
| NRIP2 | 1.823547 | 6.82E-20 | 3.27E-19 |
| CETP | -2.24486 | 2.59E-22 | 1.89E-21 |
| SAMD4B | 1.295206 | 4.66E-27 | 2.03E-25 |
| RDH16 | -1.48621 | 1.45E-19 | 6.68E-19 |
| C6orf226 | 1.152194 | 6.21E-17 | 2.09E-16 |
| MFSD14C | 1.01 | 2.26E-14 | 5.93E-14 |
| ZNF547 | 1.508661 | 2.56E-14 | 6.68E-14 |
| ARRDC2 | 1.636551 | 3.65E-11 | 7.49E-11 |
| AGAP9 | 1.016956 | 8.59E-06 | 1.23E-05 |
| IMPDH1 | 1.648644 | 7.85E-07 | 1.21E-06 |
| STK11IP | 1.450471 | 6.57E-23 | 5.48E-22 |
| PAQR6 | 2.219052 | 5.20E-17 | 1.76E-16 |
| NSD1 | 1.291742 | 1.27E-20 | 6.84E-20 |
| RASEF | 1.855637 | 0.000157 | 0.000207 |
| MAL2 | 1.291263 | 1.66E-06 | 2.50E-06 |
| LAIR2 | 2.546583 | 2.48E-09 | 4.48E-09 |
| ZNF76 | 1.229813 | 4.08E-26 | 1.02E-24 |
| THRSP | -1.00629 | 9.26E-13 | 2.13E-12 |
| RXRB | 1.178769 | 1.28E-25 | 2.52E-24 |
| PRR11 | 3.262667 | 9.82E-24 | 1.02E-22 |
| BHMT | -1.07162 | 2.19E-13 | 5.30E-13 |
| TUBA3D | 1.727285 | 0.000763 | 0.000965 |
| ITFG2 | 1.145398 | 3.95E-20 | 1.96E-19 |
| TNNI3 | 4.090793 | 0.000122 | 0.000162 |
| CLIC1 | 1.608771 | 3.93E-18 | 1.51E-17 |
| NCAPD2 | 2.187694 | 4.64E-24 | 5.23E-23 |
| HIST1H2AD | 2.669896 | 4.19E-13 | 9.91E-13 |
| PCED1B | 1.125223 | 9.11E-05 | 0.000122 |
| MRE11 | 1.167009 | 1.02E-18 | 4.18E-18 |
| SLF1 | 1.103175 | 6.88E-14 | 1.74E-13 |
| ARPC5 | 1.343859 | 7.64E-26 | 1.65E-24 |
| NKAP | 1.077308 | 2.58E-26 | 7.05E-25 |
| NASP | 1.247233 | 9.58E-20 | 4.50E-19 |
| SH2B1 | 1.213216 | 1.08E-23 | 1.11E-22 |
| FLOT1 | 1.097297 | 4.24E-21 | 2.48E-20 |
| SH3PXD2A | 1.163146 | 8.17E-13 | 1.89E-12 |
| KCNC3 | 1.315845 | 9.45E-09 | 1.64E-08 |
| UPF3A | 1.1545 | 6.46E-22 | 4.40E-21 |
| RCE1 | 1.206577 | 4.48E-20 | 2.21E-19 |
| AATF | 1.294012 | 1.21E-26 | 4.03E-25 |
| PIK3R6 | 1.452926 | 9.68E-13 | 2.22E-12 |
| MVD | 1.515828 | 7.26E-18 | 2.72E-17 |
| PPM1F | 1.622519 | 1.33E-26 | 4.30E-25 |
| RPSA | 1.531827 | 5.14E-22 | 3.57E-21 |
| NPC1 | 1.408733 | 1.77E-20 | 9.32E-20 |
| BBC3 | 1.492785 | 1.16E-19 | 5.38E-19 |
| PAQR9 | 1.146767 | 5.11E-10 | 9.66E-10 |
| BTN2A1 | 1.121041 | 2.42E-16 | 7.67E-16 |
| RTBDN | 6.123378 | 6.16E-11 | 1.24E-10 |
| CRELD1 | 1.01023 | 4.33E-23 | 3.77E-22 |
| TMEM74 | 2.333766 | 3.59E-11 | 7.36E-11 |
| RBP7 | 2.499361 | 1.63E-21 | 1.03E-20 |
| KRT81 | 2.789352 | 4.69E-05 | 6.41E-05 |
| CAPN2 | 1.419192 | 3.60E-17 | 1.25E-16 |
| TES | 1.349363 | 1.02E-09 | 1.89E-09 |
| TMCO3 | 1.966917 | 3.29E-20 | 1.66E-19 |
| HIST1H3C | 2.190596 | 1.26E-10 | 2.50E-10 |
| ANKRD13B | 2.445194 | 7.36E-22 | 4.99E-21 |
| ACTN2 | 5.278942 | 3.77E-09 | 6.73E-09 |
| KCNN4 | 1.584073 | 7.77E-06 | 1.12E-05 |
| TIA1 | 1.239678 | 4.58E-17 | 1.57E-16 |
| MTX3 | 1.080099 | 5.19E-19 | 2.20E-18 |
| REN | 2.557881 | 0.001046 | 0.001309 |
| PCDHGA4 | 2.156696 | 0.003342 | 0.004049 |
| LGALS1 | 1.349165 | 3.02E-09 | 5.41E-09 |
| TP53I11 | 1.357442 | 2.21E-14 | 5.82E-14 |
| KLB | 1.010064 | 7.18E-07 | 1.11E-06 |
| DMWD | 1.237646 | 1.66E-25 | 3.13E-24 |
| TEAD2 | 2.123324 | 3.32E-15 | 9.35E-15 |
| MMP12 | 4.928723 | 7.18E-10 | 1.35E-09 |
| UGGT1 | 1.270359 | 2.30E-21 | 1.41E-20 |
| COLEC10 | -3.4178 | 1.27E-28 | 3.14E-26 |
| COL12A1 | 1.112504 | 0.034296 | 0.038576 |
| ZNF530 | 1.828693 | 1.14E-16 | 3.75E-16 |
| HIST1H2BF | 3.201786 | 2.74E-11 | 5.65E-11 |
| STX4 | 1.158098 | 3.26E-25 | 5.44E-24 |
| JCHAIN | -1.12286 | 1.70E-13 | 4.14E-13 |
| PLG | -1.15095 | 1.74E-19 | 7.91E-19 |
| CSPG4 | 2.854228 | 1.48E-25 | 2.83E-24 |
| SOX18 | 1.381673 | 2.68E-16 | 8.44E-16 |
| RPS3 | 1.151924 | 9.70E-17 | 3.20E-16 |
| EXO5 | 1.075774 | 1.53E-16 | 4.94E-16 |
| LY6H | 4.999836 | 3.84E-23 | 3.38E-22 |
| ALDOA | 1.811157 | 2.08E-19 | 9.35E-19 |
| SUV39H1 | 1.56715 | 7.15E-26 | 1.58E-24 |
| WIZ | 1.176057 | 1.67E-24 | 2.17E-23 |
| COLQ | 1.159341 | 5.74E-10 | 1.08E-09 |
| POP1 | 1.378777 | 4.19E-24 | 4.78E-23 |
| PARVB | 1.384532 | 7.54E-08 | 1.24E-07 |
| TRIM45 | 3.066118 | 1.92E-25 | 3.52E-24 |
| CCM2L | 1.13563 | 6.97E-15 | 1.91E-14 |
| TRIM39 | 1.248446 | 7.43E-27 | 2.75E-25 |
| PRRC2A | 1.312325 | 2.47E-20 | 1.27E-19 |
| MAFA | 7.131902 | 4.89E-14 | 1.25E-13 |
| TSPAN5 | 2.896774 | 2.15E-07 | 3.42E-07 |
| PNMA5 | 7.608562 | 0.000284 | 0.000369 |
| TIGD7 | 1.849027 | 4.54E-21 | 2.65E-20 |
| PES1 | 1.070458 | 2.04E-21 | 1.27E-20 |
| TTC7A | 1.098144 | 2.76E-14 | 7.19E-14 |
| IL6 | -1.43476 | 3.11E-05 | 4.31E-05 |
| WDCP | 1.37922 | 1.03E-25 | 2.09E-24 |
| ARHGAP22 | 2.047891 | 3.95E-16 | 1.22E-15 |
| LRRC20 | 1.282603 | 1.28E-16 | 4.18E-16 |
| POMT2 | 1.262161 | 5.14E-22 | 3.57E-21 |
| ZNF212 | 1.022959 | 2.24E-22 | 1.66E-21 |
| CYB5R1 | 1.336324 | 1.52E-25 | 2.89E-24 |
| TAF6 | 1.653912 | 9.02E-22 | 5.97E-21 |
| MFSD4A | 1.406275 | 0.00496 | 0.005921 |
| TRMT1 | 1.545014 | 4.42E-27 | 1.95E-25 |
| PRAG1 | 1.422034 | 4.97E-07 | 7.74E-07 |
| CLGN | 2.060589 | 2.65E-05 | 3.68E-05 |
| SMARCD1 | 1.377799 | 7.30E-25 | 1.07E-23 |
| ZNF384 | 1.160695 | 6.95E-13 | 1.61E-12 |
| SCIMP | -1.01652 | 5.71E-12 | 1.23E-11 |
| GMPPA | 1.055794 | 1.45E-24 | 1.93E-23 |
| SIX5 | 1.164564 | 3.79E-16 | 1.17E-15 |
| PLCD4 | 1.805931 | 1.23E-18 | 4.98E-18 |
| NSL1 | 1.065345 | 4.61E-23 | 3.99E-22 |
| GAB2 | 1.335752 | 6.51E-15 | 1.78E-14 |
| RBM39 | 1.022023 | 1.94E-22 | 1.46E-21 |
| RHBDF2 | 1.747503 | 1.67E-18 | 6.65E-18 |
| GLMP | 1.617455 | 4.56E-22 | 3.20E-21 |
| ZSWIM1 | 1.375815 | 2.30E-25 | 4.08E-24 |
| GNPDA2 | 1.052755 | 1.31E-10 | 2.58E-10 |
| ZNF426 | 1.093131 | 5.90E-06 | 8.54E-06 |
| EIF5A2 | 2.109171 | 2.85E-06 | 4.22E-06 |
| VSIG10L | 2.660363 | 2.49E-11 | 5.15E-11 |
| KIF12 | 1.34107 | 0.038694 | 0.043372 |
| CIC | 1.116431 | 1.74E-18 | 6.94E-18 |
| JRKL | 1.633916 | 5.02E-19 | 2.14E-18 |
| ACBD6 | 1.757009 | 3.40E-28 | 4.05E-26 |
| PRPF31 | 1.109688 | 3.66E-25 | 5.93E-24 |
| TSPAN15 | 1.997581 | 4.03E-11 | 8.25E-11 |
| FAM180A | -1.82217 | 1.27E-22 | 9.99E-22 |
| GPD1 | -1.05427 | 4.63E-13 | 1.09E-12 |
| IYD | -1.01098 | 2.25E-13 | 5.44E-13 |
| NEURL1B | 1.025801 | 5.54E-09 | 9.79E-09 |
| DBP | 1.203223 | 2.04E-12 | 4.57E-12 |
| TGIF2 | 1.048439 | 1.64E-09 | 3.00E-09 |
| ZBED5 | 1.268208 | 8.75E-20 | 4.14E-19 |
| FER1L6 | 6.910953 | 1.08E-08 | 1.88E-08 |
| POU5F1 | 2.704226 | 3.69E-18 | 1.42E-17 |
| DGKZ | 1.334536 | 1.11E-23 | 1.14E-22 |
| WBP1 | 1.098146 | 1.43E-18 | 5.76E-18 |
| H2AFZ | 1.875475 | 2.29E-26 | 6.54E-25 |
| C12orf45 | 1.076271 | 1.52E-19 | 6.97E-19 |
| SLCO2A1 | 1.775512 | 2.16E-07 | 3.45E-07 |
| MTERF3 | 1.310377 | 6.50E-24 | 7.13E-23 |
| ZDHHC23 | 1.106874 | 3.04E-09 | 5.45E-09 |
| RBM45 | 1.047434 | 2.36E-25 | 4.18E-24 |
| C1orf105 | 1.703689 | 7.28E-10 | 1.36E-09 |
| DDX51 | 1.101604 | 7.75E-24 | 8.28E-23 |
| NAT10 | 1.039932 | 1.94E-24 | 2.48E-23 |
| ZNF572 | 2.094651 | 2.09E-20 | 1.09E-19 |
| POLR3F | 1.163071 | 2.94E-25 | 5.03E-24 |
| LBR | 1.159377 | 2.60E-16 | 8.21E-16 |
| ZNF550 | 1.618452 | 7.08E-14 | 1.78E-13 |
| ALG12 | 1.00925 | 2.15E-14 | 5.67E-14 |
| C3orf67 | 1.178738 | 3.08E-05 | 4.27E-05 |
| ZNF302 | 1.049311 | 4.11E-16 | 1.27E-15 |
| CKS2 | 2.06227 | 4.51E-22 | 3.16E-21 |
| ARHGAP27 | 1.032363 | 1.24E-07 | 2.01E-07 |
| FLT1 | 1.027421 | 2.78E-10 | 5.36E-10 |
| YAE1 | 1.207832 | 9.14E-23 | 7.39E-22 |
| ZNF512 | 1.539293 | 1.92E-24 | 2.45E-23 |
| C16orf91 | 1.021662 | 1.32E-20 | 7.15E-20 |
| ASIC1 | 4.087933 | 4.10E-14 | 1.05E-13 |
| EPS8L3 | 5.590769 | 2.94E-19 | 1.29E-18 |
| INO80C | 1.105307 | 9.13E-16 | 2.71E-15 |
| PLAU | 2.0275 | 4.04E-15 | 1.13E-14 |
| ZFP62 | 1.674616 | 3.22E-26 | 8.53E-25 |
| MAGEA1 | 9.989469 | 1.26E-13 | 3.11E-13 |
| ALDH2 | -1.11864 | 1.47E-22 | 1.14E-21 |
| KRBA1 | 1.783795 | 7.14E-10 | 1.34E-09 |
| PRKCD | 1.413025 | 8.94E-16 | 2.66E-15 |
| GRIN2D | 1.745612 | 4.51E-06 | 6.59E-06 |
| CHADL | 1.361869 | 0.004979 | 0.005943 |
| PTPRG | 1.484253 | 1.16E-15 | 3.42E-15 |
| MZT2A | 1.074352 | 3.83E-12 | 8.41E-12 |
| POU2F1 | 1.09359 | 6.13E-19 | 2.58E-18 |
| METTL5 | 1.176767 | 4.84E-26 | 1.16E-24 |
| ZNF337 | 1.500393 | 2.15E-12 | 4.81E-12 |
| GBP2 | 1.957738 | 9.02E-19 | 3.73E-18 |
| SMARCC1 | 1.173822 | 2.88E-19 | 1.27E-18 |
| PPP2R2C | 5.52929 | 1.99E-06 | 2.97E-06 |
| CD2BP2 | 1.209119 | 6.65E-23 | 5.54E-22 |
| ATP6V1E1 | 1.076304 | 3.47E-25 | 5.71E-24 |
| EHD3 | -1.47285 | 9.30E-17 | 3.08E-16 |
| AHCTF1 | 1.207825 | 6.30E-20 | 3.04E-19 |
| ZNRD1 | 1.218672 | 4.96E-20 | 2.44E-19 |
| MEIS3 | 1.128249 | 0.013309 | 0.01537 |
| ATP6AP1L | 1.09029 | 2.33E-11 | 4.83E-11 |
| ZNF496 | 1.259807 | 1.96E-06 | 2.93E-06 |
| TDRD10 | 1.538621 | 2.59E-05 | 3.61E-05 |
| C18orf21 | 1.065851 | 5.06E-24 | 5.65E-23 |
| PTK7 | 2.979068 | 1.99E-08 | 3.40E-08 |
| FAM199X | 1.055724 | 1.32E-17 | 4.81E-17 |
| CACNB3 | 1.858056 | 2.42E-15 | 6.90E-15 |
| MYO7B | 1.883324 | 0.000294 | 0.000381 |
| ZNF852 | 1.16449 | 1.69E-15 | 4.92E-15 |
| MCM5 | 2.110274 | 3.84E-24 | 4.44E-23 |
| RASAL2 | 1.438349 | 1.26E-13 | 3.11E-13 |
| FBXO43 | 4.790395 | 2.11E-28 | 3.44E-26 |
| PMS1 | 1.130505 | 3.39E-21 | 2.02E-20 |
| MMS22L | 2.057124 | 1.82E-17 | 6.51E-17 |
| ZSWIM5 | 2.680536 | 7.46E-12 | 1.60E-11 |
| NKTR | 1.219908 | 9.43E-13 | 2.17E-12 |
| PRR5L | 1.810675 | 0.000742 | 0.000938 |
| ARFGEF3 | 3.703365 | 4.66E-08 | 7.73E-08 |
| CTNNA1 | 1.132016 | 2.23E-24 | 2.79E-23 |
| AARSD1 | 1.405151 | 1.71E-25 | 3.20E-24 |
| BUD23 | 1.038583 | 1.30E-25 | 2.55E-24 |
| FIZ1 | 1.00707 | 4.78E-23 | 4.12E-22 |
| SAA4 | -1.31203 | 1.88E-18 | 7.45E-18 |
| RPS20 | 1.298026 | 1.10E-16 | 3.61E-16 |
| TRIL | 1.452517 | 2.49E-15 | 7.10E-15 |
| KLRF1 | -1.19297 | 9.59E-16 | 2.85E-15 |
| INKA1 | 2.085576 | 8.09E-25 | 1.16E-23 |
| ALDH6A1 | -1.17588 | 9.37E-20 | 4.41E-19 |
| ZNF792 | 1.580172 | 6.68E-21 | 3.78E-20 |
| MAGEA8 | 8.150879 | 3.93E-07 | 6.16E-07 |
| UFD1 | 1.29508 | 2.89E-28 | 3.94E-26 |
| ZNF354B | 1.236392 | 1.91E-17 | 6.85E-17 |
| ONECUT1 | 1.105566 | 0.00015 | 0.000198 |
| CLTCL1 | 1.712262 | 5.25E-19 | 2.23E-18 |
| SLITRK3 | -2.10176 | 1.75E-17 | 6.28E-17 |
| CKAP5 | 1.137077 | 3.36E-20 | 1.69E-19 |
| RPS16 | 1.237336 | 3.98E-19 | 1.72E-18 |
| FAM162B | 2.090486 | 1.96E-18 | 7.77E-18 |
| BMPER | -3.92398 | 8.99E-29 | 2.81E-26 |
| SLC22A23 | 1.398708 | 2.36E-17 | 8.37E-17 |
| MED24 | 1.218843 | 3.74E-24 | 4.36E-23 |
| B4GALNT2 | 8.039584 | 2.65E-09 | 4.78E-09 |
| AMPD3 | 1.091081 | 7.02E-05 | 9.47E-05 |
| MRGBP | 1.554783 | 2.93E-28 | 3.94E-26 |
| TWF2 | 1.015835 | 3.44E-11 | 7.07E-11 |
| COA1 | 1.043319 | 1.13E-23 | 1.15E-22 |
| NUP155 | 1.426686 | 4.81E-24 | 5.40E-23 |
| GLYAT | -1.46076 | 1.24E-18 | 5.04E-18 |
| HOMER1 | 1.260296 | 0.000444 | 0.000568 |
| FUS | 1.368055 | 3.83E-23 | 3.38E-22 |
| NOL8 | 1.031181 | 2.58E-19 | 1.14E-18 |
| SLC38A6 | 1.81323 | 4.02E-27 | 1.86E-25 |
| RCC2 | 1.47107 | 2.21E-18 | 8.71E-18 |
| PIK3R2 | 1.882465 | 2.52E-19 | 1.12E-18 |
| BIRC5 | 4.814625 | 2.35E-28 | 3.60E-26 |
| SHC1 | 1.541731 | 5.37E-26 | 1.26E-24 |
| CIITA | 1.070709 | 1.61E-05 | 2.27E-05 |
| SLC38A7 | 1.031665 | 3.95E-20 | 1.96E-19 |
| CDCA3 | 4.048528 | 1.84E-28 | 3.44E-26 |
| GATAD2B | 1.197414 | 2.17E-21 | 1.34E-20 |
| ADGRG5 | 1.772033 | 0.016653 | 0.019111 |
| CNTNAP1 | 2.290528 | 1.36E-13 | 3.34E-13 |
| STIL | 3.152294 | 2.79E-26 | 7.50E-25 |
| DHX34 | 1.816215 | 1.49E-27 | 9.64E-26 |
| MCM6 | 2.464251 | 2.13E-25 | 3.84E-24 |
| CNTD1 | 1.695211 | 9.04E-21 | 4.99E-20 |
| SCRIB | 1.97927 | 8.37E-27 | 2.98E-25 |
| SNAP47 | 1.19908 | 9.57E-22 | 6.30E-21 |
| SLC38A5 | 2.130904 | 3.89E-06 | 5.70E-06 |
| CCL20 | 1.944409 | 9.83E-08 | 1.60E-07 |
| FGF12 | 2.509103 | 2.65E-10 | 5.12E-10 |
| CXCL12 | -2.05117 | 1.08E-24 | 1.50E-23 |
| POLR2G | 1.24595 | 3.97E-26 | 9.98E-25 |
| EXOSC9 | 1.190136 | 3.10E-24 | 3.69E-23 |
| CENPB | 1.049599 | 1.59E-24 | 2.09E-23 |
| ARL4A | 1.477344 | 6.95E-11 | 1.40E-10 |
| CCDC134 | 1.31507 | 2.25E-20 | 1.17E-19 |
| PLAT | 1.113985 | 0.002229 | 0.002735 |
| PDAP1 | 1.036604 | 5.33E-27 | 2.23E-25 |
| CSNK2A1 | 1.032274 | 1.59E-20 | 8.46E-20 |
| FCGR2B | -1.10985 | 1.30E-18 | 5.25E-18 |
| DDHD1 | 1.110181 | 1.98E-09 | 3.60E-09 |
| RNF31 | 1.066278 | 2.34E-13 | 5.64E-13 |
| HID1 | 1.815618 | 5.88E-05 | 7.98E-05 |
| HSPA5 | 1.071416 | 1.04E-19 | 4.84E-19 |
| MED7 | 1.018626 | 3.43E-24 | 4.02E-23 |
| MFSD2A | -1.76794 | 2.32E-17 | 8.20E-17 |
| MLST8 | 1.045507 | 4.72E-23 | 4.08E-22 |
| CLTA | 1.189944 | 2.28E-23 | 2.15E-22 |
| MED22 | 1.627207 | 3.90E-25 | 6.27E-24 |
| MRPL13 | 1.338423 | 2.29E-24 | 2.85E-23 |
| LDLRAD1 | 4.569365 | 2.57E-15 | 7.31E-15 |
| SFRP1 | -1.59287 | 6.14E-22 | 4.21E-21 |
| CCDC196 | -1.42703 | 9.97E-18 | 3.68E-17 |
| NOL12 | 1.803528 | 3.00E-27 | 1.52E-25 |
| C12orf49 | 1.610973 | 1.11E-15 | 3.29E-15 |
| ABCF1 | 1.004129 | 1.20E-21 | 7.78E-21 |
| MVB12A | 1.180666 | 5.25E-20 | 2.56E-19 |
| PM20D2 | 1.349249 | 1.21E-09 | 2.23E-09 |
| SLC1A4 | 1.388176 | 6.03E-15 | 1.66E-14 |
| GNA12 | 1.327691 | 3.01E-19 | 1.32E-18 |
| YWHAZ | 1.311309 | 1.22E-16 | 3.98E-16 |
| MPP7 | 1.373141 | 1.62E-06 | 2.44E-06 |
| ZBTB37 | 1.15743 | 6.82E-20 | 3.27E-19 |
| GABPB2 | 1.244926 | 7.90E-19 | 3.29E-18 |
| UNC119 | 1.300574 | 8.70E-14 | 2.18E-13 |
| FITM2 | 1.163439 | 6.01E-17 | 2.03E-16 |
| DEPTOR | 1.017977 | 5.75E-13 | 1.34E-12 |
| FSD1L | 1.89034 | 3.80E-17 | 1.31E-16 |
| C14orf93 | 1.468559 | 6.00E-27 | 2.39E-25 |
| CEP112 | 1.209614 | 5.41E-15 | 1.49E-14 |
| ADSS | 1.030036 | 2.47E-17 | 8.72E-17 |
| RAB11FIP4 | 2.507286 | 2.09E-22 | 1.55E-21 |
| WDR27 | 1.530642 | 4.09E-17 | 1.41E-16 |
| CCNB2 | 4.49351 | 9.55E-28 | 7.23E-26 |
| SEMA3B | 2.174583 | 6.74E-07 | 1.04E-06 |
| BRCA2 | 2.056943 | 4.81E-19 | 2.05E-18 |
| DLGAP4 | 1.267492 | 8.60E-22 | 5.73E-21 |
| CYR61 | -1.35363 | 1.35E-14 | 3.59E-14 |
| ZNF195 | 1.219049 | 2.44E-21 | 1.49E-20 |
| DPYSL4 | 2.459117 | 0.000417 | 0.000535 |
| NRSN2 | 2.063217 | 0.001019 | 0.001277 |
| POLN | 1.742292 | 1.01E-11 | 2.16E-11 |
| SHROOM4 | 1.166253 | 7.36E-10 | 1.38E-09 |
| SNX29 | 1.100716 | 4.60E-12 | 1.00E-11 |
| KDM5C | 1.00199 | 2.49E-15 | 7.10E-15 |
| CAPN9 | 3.51492 | 2.09E-11 | 4.35E-11 |
| OSER1 | 1.013575 | 4.75E-24 | 5.34E-23 |
| GADD45B | -1.37855 | 6.51E-15 | 1.78E-14 |
| CORO2A | 1.355512 | 4.54E-06 | 6.62E-06 |
| H2AFX | 2.172701 | 8.35E-24 | 8.86E-23 |
| PIP5K1C | 1.341962 | 3.88E-23 | 3.41E-22 |
| TMEM98 | 1.878492 | 4.85E-13 | 1.14E-12 |
| SYT8 | 3.792272 | 0.014961 | 0.017221 |
| PTPN7 | 1.059303 | 6.13E-05 | 8.30E-05 |
| WDSUB1 | 1.177526 | 7.11E-18 | 2.67E-17 |
| BOLA2-SMG1P6 | 1.353278 | 2.01E-15 | 5.78E-15 |
| CAMLG | 1.071725 | 3.21E-25 | 5.38E-24 |
| ERI3 | 1.05337 | 2.29E-24 | 2.85E-23 |
| COL22A1 | 5.236141 | 6.09E-07 | 9.44E-07 |
| PARM1 | 1.802371 | 3.27E-07 | 5.15E-07 |
| BMP8B | 2.12609 | 0.004922 | 0.005879 |
| MARK2 | 1.01364 | 8.93E-17 | 2.96E-16 |
| C2orf68 | 1.06747 | 7.37E-24 | 7.97E-23 |
| SIGLEC14 | -1.08259 | 1.42E-06 | 2.14E-06 |
| CRTAP | 1.200617 | 9.22E-19 | 3.81E-18 |
| HPDL | 3.024171 | 1.75E-16 | 5.63E-16 |
| PROCA1 | 2.170766 | 3.12E-21 | 1.88E-20 |
| SMC4 | 2.062192 | 1.55E-13 | 3.79E-13 |
| ADGRG7 | -2.0534 | 1.78E-24 | 2.30E-23 |
| PUS1 | 1.307392 | 3.47E-23 | 3.11E-22 |
| KATNAL2 | 1.137899 | 1.03E-07 | 1.68E-07 |
| ST6GALNAC4 | 1.653704 | 3.80E-12 | 8.34E-12 |
| MRPL47 | 1.011555 | 6.68E-21 | 3.78E-20 |
| SLF2 | 1.345093 | 1.03E-18 | 4.22E-18 |
| ALOX12 | 1.336353 | 1.04E-12 | 2.38E-12 |
| FNBP4 | 1.091584 | 1.01E-15 | 2.99E-15 |
| TOR3A | 1.495215 | 1.30E-18 | 5.25E-18 |
| HDDC2 | 1.026687 | 5.24E-14 | 1.34E-13 |
| TIMM9 | 1.046916 | 9.03E-23 | 7.32E-22 |
| PIF1 | 3.486794 | 1.78E-24 | 2.30E-23 |
| SLBP | 1.279029 | 4.10E-25 | 6.53E-24 |
| CLSTN1 | 1.366638 | 6.56E-09 | 1.15E-08 |
| STARD3 | 1.09492 | 3.74E-24 | 4.36E-23 |
| RPS6KC1 | 1.314903 | 6.08E-21 | 3.47E-20 |
| SPSB2 | 2.189186 | 4.13E-27 | 1.88E-25 |
| DIS3L2 | 1.012807 | 1.56E-26 | 4.82E-25 |
| LOXL1 | 2.171809 | 0.001006 | 0.001261 |
| KLHL30 | 3.399086 | 1.52E-08 | 2.61E-08 |
| COPG2 | 1.308555 | 2.56E-20 | 1.32E-19 |
| TIGD1 | 2.514737 | 6.12E-26 | 1.39E-24 |
| DTL | 3.985564 | 3.35E-26 | 8.78E-25 |
| PNMA3 | 4.27844 | 5.46E-08 | 9.04E-08 |
| C1orf198 | 1.789723 | 3.60E-20 | 1.80E-19 |
| SPINDOC | 1.959968 | 2.53E-20 | 1.30E-19 |
| GMPPB | 1.170051 | 8.29E-23 | 6.77E-22 |
| DUS1L | 1.187675 | 1.35E-25 | 2.64E-24 |
| LRRN2 | 2.563544 | 3.55E-07 | 5.58E-07 |
| ELMOD3 | 1.121365 | 1.38E-24 | 1.84E-23 |
| SOGA1 | 2.230067 | 1.35E-21 | 8.66E-21 |
| SNRPD2 | 1.676346 | 6.68E-25 | 9.97E-24 |
| SP5 | 3.141964 | 6.06E-12 | 1.31E-11 |
| WFS1 | 1.665485 | 1.56E-26 | 4.82E-25 |
| GPSM2 | 2.398571 | 1.00E-24 | 1.41E-23 |
| HGS | 1.594091 | 3.22E-22 | 2.32E-21 |
| SAE1 | 1.529647 | 7.84E-27 | 2.85E-25 |
| CAPN15 | 1.172747 | 4.09E-20 | 2.02E-19 |
| MFAP2 | 2.640216 | 0.000882 | 0.00111 |
| COCH | 5.039711 | 2.91E-13 | 6.97E-13 |
| ZSCAN16 | 1.794161 | 1.18E-24 | 1.62E-23 |
| CDKN2AIPNL | 1.617158 | 4.54E-27 | 1.99E-25 |
| FBXO45 | 1.153418 | 9.70E-24 | 1.01E-22 |
| BRPF3 | 1.162551 | 4.47E-13 | 1.05E-12 |
| C5orf46 | 4.47999 | 1.44E-17 | 5.22E-17 |
| SOWAHA | 2.480436 | 4.98E-17 | 1.69E-16 |
| SELENON | 1.226626 | 3.60E-17 | 1.25E-16 |
| DCUN1D2 | 1.037413 | 1.09E-16 | 3.57E-16 |
| DGKD | 1.30375 | 4.07E-19 | 1.75E-18 |
| RPS6KA4 | 1.161323 | 1.89E-22 | 1.43E-21 |
| PRR19 | 2.666681 | 9.25E-11 | 1.84E-10 |
| ADAM9 | 1.571046 | 1.13E-09 | 2.08E-09 |
| ZNF385A | 1.054987 | 1.71E-06 | 2.56E-06 |
| TCF15 | 2.878419 | 5.55E-25 | 8.54E-24 |
| DPF3 | -1.14132 | 1.26E-14 | 3.36E-14 |
| REEP2 | 3.78459 | 1.40E-18 | 5.64E-18 |
| CRHBP | -3.98069 | 4.23E-28 | 4.56E-26 |
| PIGF | 1.017768 | 1.19E-21 | 7.70E-21 |
| C2orf16 | 1.344098 | 3.26E-10 | 6.25E-10 |
| ZNF526 | 1.346961 | 3.22E-28 | 4.02E-26 |
| REG3A | 11.19517 | 3.83E-09 | 6.83E-09 |
| PEA15 | 1.834112 | 3.57E-27 | 1.72E-25 |
| TTLL3 | 1.695111 | 5.09E-18 | 1.94E-17 |
| TCEANC2 | 1.250671 | 3.35E-21 | 2.00E-20 |
| HSPG2 | 1.336489 | 1.35E-05 | 1.91E-05 |
| TBL2 | 1.070536 | 1.67E-24 | 2.17E-23 |
| PLOD3 | 1.654105 | 6.33E-27 | 2.45E-25 |
| PRTFDC1 | 1.961382 | 5.96E-12 | 1.29E-11 |
| CDK1 | 4.239388 | 7.09E-28 | 5.92E-26 |
| SLC35B2 | 1.23531 | 4.30E-22 | 3.03E-21 |
| NOMO3 | 1.024232 | 0.001463 | 0.001813 |
| EED | 1.255517 | 1.10E-24 | 1.52E-23 |
| CD109 | 3.152994 | 4.03E-16 | 1.24E-15 |
| ZNF506 | 1.310176 | 2.31E-07 | 3.67E-07 |
| C5orf58 | 3.819273 | 5.99E-05 | 8.12E-05 |
| HOXB3 | 1.249866 | 0.000938 | 0.001178 |
| RNF219 | 1.027472 | 2.06E-13 | 4.99E-13 |
| SLC25A6 | 1.13195 | 2.29E-17 | 8.12E-17 |
| MITD1 | 1.185883 | 3.78E-23 | 3.34E-22 |
| ATP13A1 | 1.276051 | 2.54E-26 | 6.97E-25 |
| COX6C | 1.452686 | 7.33E-21 | 4.12E-20 |
| HM13 | 1.266917 | 2.68E-26 | 7.29E-25 |
| ANKRD13D | 1.467986 | 4.65E-19 | 1.99E-18 |
| LTB | 1.635728 | 0.000297 | 0.000384 |
| DMBT1 | 7.19408 | 5.11E-06 | 7.43E-06 |
| CCAR1 | 1.088147 | 5.31E-19 | 2.25E-18 |
| ABHD11 | 1.29065 | 9.59E-16 | 2.85E-15 |
| ZBTB34 | 1.19853 | 1.31E-14 | 3.49E-14 |
| ATP6V0B | 1.030194 | 1.14E-18 | 4.63E-18 |
| ARFGAP1 | 1.350538 | 3.92E-27 | 1.83E-25 |
| PLXNA2 | 1.334378 | 1.42E-20 | 7.62E-20 |
| SERPINA11 | -1.47983 | 1.36E-20 | 7.30E-20 |
| TFAP2A | 2.967708 | 5.48E-08 | 9.07E-08 |
| PTGDR2 | 2.672432 | 6.01E-12 | 1.30E-11 |
| MBOAT7 | 1.247644 | 2.35E-24 | 2.91E-23 |
| P3H1 | 1.292892 | 2.60E-24 | 3.17E-23 |
| TMEM161A | 1.115978 | 4.44E-23 | 3.85E-22 |
| TNFRSF18 | 2.561731 | 9.11E-14 | 2.27E-13 |
| ZMYND19 | 1.369637 | 6.36E-26 | 1.43E-24 |
| TRIM54 | 5.109791 | 2.25E-10 | 4.37E-10 |
| ANXA10 | -1.30643 | 1.45E-16 | 4.70E-16 |
| NGFR | -1.33621 | 8.26E-18 | 3.07E-17 |
| ARHGEF28 | 1.217964 | 3.84E-08 | 6.41E-08 |
| PQBP1 | 1.158528 | 9.34E-24 | 9.80E-23 |
| USP54 | 1.850573 | 1.65E-09 | 3.02E-09 |
| DLL4 | 1.756678 | 9.02E-22 | 5.97E-21 |
| UBAP2 | 1.328091 | 4.34E-21 | 2.54E-20 |
| FSCN1 | 1.108809 | 0.001954 | 0.002405 |
| YDJC | 1.326132 | 4.35E-22 | 3.06E-21 |
| MCM7 | 2.168309 | 1.39E-26 | 4.39E-25 |
| CDKN3 | 4.619531 | 9.90E-29 | 2.81E-26 |
| IFI27L1 | 1.625187 | 4.67E-22 | 3.27E-21 |
| ABCC5 | 1.746626 | 5.88E-24 | 6.50E-23 |
| RET | -1.52478 | 2.42E-15 | 6.90E-15 |
| PRELID3A | 2.043759 | 1.04E-07 | 1.70E-07 |
| BTNL9 | 1.23686 | 7.31E-06 | 1.05E-05 |
| ILF3 | 1.548646 | 1.95E-27 | 1.15E-25 |
| SNRPB | 1.820133 | 1.04E-26 | 3.52E-25 |
| TBC1D16 | 2.37407 | 1.11E-27 | 7.79E-26 |
| DHDH | 2.677023 | 3.88E-10 | 7.39E-10 |
| IQCG | 1.273088 | 5.25E-16 | 1.60E-15 |
| C1orf131 | 1.398884 | 1.29E-21 | 8.31E-21 |
| LAMTOR1 | 1.101421 | 2.55E-25 | 4.44E-24 |
| CEP55 | 3.908425 | 3.85E-25 | 6.21E-24 |
| RFC2 | 1.162892 | 2.50E-22 | 1.83E-21 |
| DOCK6 | 1.479733 | 7.64E-26 | 1.65E-24 |
| DEPDC1B | 4.029775 | 4.04E-21 | 2.37E-20 |
| MAGEA6 | 8.895572 | 1.75E-05 | 2.47E-05 |
| TMEM267 | 1.376629 | 2.22E-22 | 1.64E-21 |
| DOK7 | 2.10553 | 0.000367 | 0.000472 |
| DDX41 | 1.31398 | 8.75E-29 | 2.81E-26 |
| HKR1 | 1.165717 | 4.91E-15 | 1.36E-14 |
| PRCC | 1.593973 | 1.27E-28 | 3.14E-26 |
| DNPH1 | 1.092676 | 1.61E-18 | 6.45E-18 |
| ZNF500 | 1.216511 | 2.30E-25 | 4.08E-24 |
| C20orf27 | 1.481058 | 2.46E-23 | 2.30E-22 |
| CLIC5 | 2.11602 | 2.96E-08 | 4.98E-08 |
| NOV | 1.891165 | 2.18E-09 | 3.95E-09 |
| UBE2S | 2.644895 | 8.71E-27 | 3.08E-25 |
| CXXC1 | 1.07697 | 1.31E-23 | 1.32E-22 |
| GTF2IRD1 | 1.822133 | 5.67E-21 | 3.25E-20 |
| SEMA4F | 2.078276 | 2.04E-13 | 4.95E-13 |
| DDX39A | 2.117835 | 2.88E-27 | 1.48E-25 |
| DKK1 | 6.431558 | 3.31E-08 | 5.55E-08 |
| DIAPH3 | 4.261997 | 1.54E-26 | 4.80E-25 |
| ATP1A2 | 2.742096 | 0.000412 | 0.000529 |
| ZNF493 | 1.094101 | 3.72E-09 | 6.63E-09 |
| PLAC8L1 | 2.092775 | 1.37E-15 | 4.00E-15 |
| CSPG5 | 3.246434 | 1.30E-21 | 8.39E-21 |
| OLA1 | 1.206127 | 1.21E-22 | 9.57E-22 |
| AL365205.1 | 1.370766 | 8.65E-20 | 4.10E-19 |
| SNRPC | 1.521861 | 1.62E-27 | 1.02E-25 |
| CLEC4G | -4.96477 | 2.54E-29 | 1.72E-26 |
| CHST11 | 1.393589 | 0.008023 | 0.009427 |
| FBN1 | 1.176563 | 0.002011 | 0.002474 |
| BUB1 | 4.278571 | 4.42E-27 | 1.95E-25 |
| CAPNS1 | 1.10214 | 5.81E-23 | 4.92E-22 |
| RELB | 1.170748 | 1.19E-11 | 2.52E-11 |
| CST1 | 8.400849 | 3.12E-08 | 5.24E-08 |
| STRIP1 | 1.00527 | 2.66E-17 | 9.35E-17 |
| AJM1 | 2.564397 | 2.69E-27 | 1.42E-25 |
| CFP | -2.83936 | 1.56E-26 | 4.82E-25 |
| HAUS7 | 1.598382 | 4.77E-15 | 1.32E-14 |
| MDM4 | 1.465352 | 4.19E-21 | 2.46E-20 |
| RAD1 | 1.280279 | 8.93E-26 | 1.87E-24 |
| CHAF1B | 3.340119 | 6.03E-24 | 6.64E-23 |
| SRGAP2 | 1.523156 | 2.64E-20 | 1.36E-19 |
| RTN4RL1 | 1.508104 | 0.00028 | 0.000364 |
| RNPC3 | 1.581592 | 3.00E-20 | 1.53E-19 |
| LPL | 2.961704 | 1.86E-21 | 1.16E-20 |
| PKIA | 2.934849 | 0.004024 | 0.004842 |
| FERMT1 | 3.011826 | 1.75E-06 | 2.62E-06 |
| GSDMB | 1.436088 | 1.66E-13 | 4.07E-13 |
| GSTO2 | 1.534738 | 0.001202 | 0.001498 |
| CKAP2L | 3.987685 | 1.20E-25 | 2.39E-24 |
| SETMAR | 1.010768 | 2.63E-19 | 1.17E-18 |
| ODAM | 8.335885 | 3.95E-06 | 5.79E-06 |
| ZNF587 | 1.340671 | 7.24E-16 | 2.18E-15 |
| AKT1S1 | 1.082898 | 1.28E-25 | 2.52E-24 |
| EXOSC4 | 1.573982 | 2.15E-19 | 9.64E-19 |
| PLA2G1B | 2.947647 | 2.58E-09 | 4.64E-09 |
| RPS6KL1 | 2.596207 | 5.05E-21 | 2.92E-20 |
| PDLIM7 | 1.943199 | 1.99E-22 | 1.49E-21 |
| PTBP3 | 1.032425 | 3.95E-14 | 1.02E-13 |
| CYBC1 | 1.184489 | 2.19E-22 | 1.62E-21 |
| ZNF425 | 1.266774 | 4.07E-12 | 8.91E-12 |
| ADAMTS7 | 1.553105 | 3.68E-16 | 1.14E-15 |
| MRPL21 | 1.218897 | 8.40E-22 | 5.61E-21 |
| MED20 | 1.299794 | 2.12E-24 | 2.67E-23 |
| ZNF778 | 1.194733 | 1.13E-21 | 7.36E-21 |
| FAM83G | 1.11484 | 4.33E-10 | 8.23E-10 |
| KCNN3 | 2.138943 | 8.05E-21 | 4.48E-20 |
| DUSP1 | -1.05261 | 6.88E-14 | 1.74E-13 |
| SALL2 | 2.249224 | 0.008411 | 0.009868 |
| AP002495.1 | 1.477783 | 3.44E-20 | 1.73E-19 |
| FAIM | 1.40677 | 3.08E-17 | 1.08E-16 |
| TRIP13 | 4.250577 | 1.74E-28 | 3.39E-26 |
| PFN4 | 2.21944 | 6.85E-16 | 2.06E-15 |
| DDOST | 1.16432 | 3.94E-24 | 4.54E-23 |
| ABHD12B | 1.770658 | 7.20E-05 | 9.71E-05 |
| DHRS13 | 1.300232 | 4.20E-11 | 8.58E-11 |
| TTLL1 | 1.569626 | 6.77E-22 | 4.61E-21 |
| SUGP1 | 1.075152 | 1.19E-27 | 8.20E-26 |
| XPO6 | 1.044157 | 1.83E-22 | 1.39E-21 |
| RPS24 | 1.062029 | 1.03E-11 | 2.19E-11 |
| NAT14 | 1.906679 | 2.52E-17 | 8.89E-17 |
| ENGASE | 1.631898 | 1.57E-24 | 2.06E-23 |
| PMF1 | 1.332987 | 2.50E-22 | 1.83E-21 |
| CTSV | 5.396759 | 1.92E-11 | 4.02E-11 |
| ANXA9 | 1.029465 | 2.43E-08 | 4.12E-08 |
| GPR89B | 1.01225 | 2.06E-17 | 7.35E-17 |
| ABHD4 | 1.447224 | 2.09E-23 | 2.00E-22 |
| CD3D | 1.165999 | 0.00374 | 0.004511 |
| TRMT10B | 1.070104 | 3.73E-20 | 1.86E-19 |
| ATF3 | -1.1881 | 1.07E-11 | 2.27E-11 |
| SEC11C | 1.114331 | 2.49E-15 | 7.10E-15 |
| BORCS8 | 1.27814 | 7.44E-26 | 1.63E-24 |
| NRXN3 | 2.039778 | 0.001129 | 0.001409 |
| STC1 | 1.712481 | 5.21E-13 | 1.22E-12 |
| C18orf54 | 2.204541 | 2.52E-17 | 8.89E-17 |
| PAFAH1B3 | 2.676107 | 2.23E-19 | 9.96E-19 |
| RBMX | 1.168929 | 7.39E-25 | 1.08E-23 |
| GOLPH3L | 1.618595 | 1.04E-25 | 2.11E-24 |
| MCM2 | 3.170194 | 5.80E-25 | 8.86E-24 |
| RNF8 | 1.379808 | 1.49E-24 | 1.97E-23 |
| ABCC4 | 2.306015 | 1.11E-09 | 2.05E-09 |
| ZNF286A | 1.119104 | 5.94E-11 | 1.20E-10 |
| CENPK | 3.391497 | 4.64E-24 | 5.23E-23 |
| NDC80 | 4.050915 | 2.26E-28 | 3.59E-26 |
| SMARCAL1 | 1.156728 | 3.56E-25 | 5.83E-24 |
| DCLRE1C | 1.510099 | 2.12E-16 | 6.75E-16 |
| GNAL | 2.641218 | 2.89E-17 | 1.01E-16 |
| ORMDL2 | 1.267121 | 5.81E-26 | 1.34E-24 |
| SPTY2D1OS | 1.75182 | 8.91E-22 | 5.91E-21 |
| BSN | 1.90588 | 1.82E-11 | 3.81E-11 |
| SGSM3 | 1.120896 | 8.81E-26 | 1.85E-24 |
| CHAF1A | 2.173987 | 5.69E-27 | 2.31E-25 |
| CCL23 | -2.35648 | 4.04E-24 | 4.63E-23 |
| ARHGEF18 | 1.286353 | 7.21E-13 | 1.67E-12 |
| PTHLH | 2.802604 | 2.49E-05 | 3.48E-05 |
| CDH16 | 3.122034 | 0.009182 | 0.010739 |
| ZNF184 | 1.204238 | 2.59E-15 | 7.37E-15 |
| PBLD | -1.13228 | 1.55E-17 | 5.59E-17 |
| SOX12 | 2.117127 | 3.11E-19 | 1.37E-18 |
| ALG6 | 1.122879 | 2.70E-24 | 3.26E-23 |
| SLC25A47 | -2.15909 | 7.34E-23 | 6.04E-22 |
| HAO2 | -1.77651 | 1.58E-20 | 8.37E-20 |
| SAMD10 | 1.57448 | 3.47E-23 | 3.11E-22 |
| AATK | 2.038613 | 0.000474 | 0.000605 |
| DKK3 | 1.773272 | 0.000102 | 0.000136 |
| CEACAM20 | 5.57927 | 4.94E-07 | 7.70E-07 |
| HIST1H2BJ | 2.426735 | 3.03E-13 | 7.25E-13 |
| DKK4 | 5.811736 | 0.005829 | 0.006922 |
| MAP3K10 | 1.366738 | 8.81E-22 | 5.85E-21 |
| EEF1A2 | 4.362892 | 3.86E-08 | 6.45E-08 |
| RBM12B | 1.359125 | 1.75E-16 | 5.61E-16 |
| C15orf41 | 1.62217 | 6.65E-23 | 5.54E-22 |
| TESMIN | 2.988145 | 1.00E-19 | 4.69E-19 |
| PLPP3 | -1.02212 | 3.08E-17 | 1.08E-16 |
| SEM1 | 1.168376 | 6.38E-22 | 4.36E-21 |
| SPDEF | 3.58993 | 0.038361 | 0.043014 |
| KIF15 | 4.144382 | 2.72E-25 | 4.71E-24 |
| PSMD14 | 1.205251 | 1.99E-24 | 2.53E-23 |
| UCK2 | 2.348627 | 2.02E-28 | 3.44E-26 |
| PXMP4 | 1.285012 | 6.77E-22 | 4.61E-21 |
| BSG | 1.639675 | 5.16E-26 | 1.23E-24 |
| TMEM45B | 1.595024 | 6.40E-08 | 1.05E-07 |
| TSPOAP1 | 1.067819 | 0.000101 | 0.000135 |
| EFCAB12 | 1.716031 | 0.001015 | 0.001271 |
| MRVI1 | 1.489436 | 1.93E-07 | 3.09E-07 |
| USHBP1 | 1.073084 | 9.28E-14 | 2.32E-13 |
| GRB7 | 1.457996 | 2.48E-08 | 4.20E-08 |
| SKP2 | 1.395785 | 5.71E-12 | 1.23E-11 |
| TOP1MT | 1.214491 | 3.64E-16 | 1.13E-15 |
| IL4I1 | 2.217289 | 4.63E-08 | 7.68E-08 |
| CHD1L | 1.358443 | 2.88E-21 | 1.74E-20 |
| DEF8 | 1.256417 | 6.26E-24 | 6.87E-23 |
| ITGB3BP | 1.548985 | 2.88E-21 | 1.74E-20 |
| NUP210 | 1.572214 | 1.59E-19 | 7.27E-19 |
| SPARC | 1.639185 | 2.35E-16 | 7.44E-16 |
| NDUFA1 | 1.228897 | 2.75E-23 | 2.54E-22 |
| FTSJ3 | 1.237552 | 1.89E-25 | 3.49E-24 |
| RHBDD3 | 1.26632 | 5.02E-23 | 4.31E-22 |
| ZNF687 | 1.684113 | 2.01E-22 | 1.51E-21 |
| ELFN2 | 4.254098 | 9.24E-13 | 2.13E-12 |
| ZRANB2 | 1.081144 | 1.93E-19 | 8.68E-19 |
| NOTCH4 | 1.125157 | 2.35E-15 | 6.71E-15 |
| KCNJ2 | 1.133532 | 2.25E-07 | 3.58E-07 |
| TRMT2B | 1.042721 | 2.78E-15 | 7.87E-15 |
| STT3A | 1.161213 | 8.19E-23 | 6.69E-22 |
| METTL27 | 1.595811 | 1.41E-08 | 2.42E-08 |
| PRX | 1.610085 | 2.58E-18 | 1.01E-17 |
| E2F4 | 1.206904 | 1.27E-22 | 1.00E-21 |
| MELTF | 2.482301 | 2.19E-05 | 3.06E-05 |
| PLA2G4C | 1.796298 | 6.42E-16 | 1.94E-15 |
| JAG1 | 1.45126 | 4.51E-07 | 7.05E-07 |
| HSPB11 | 1.206277 | 3.78E-23 | 3.34E-22 |
| MCHR1 | 1.808943 | 0.001501 | 0.001859 |
| MTMR7 | 2.535184 | 1.88E-05 | 2.63E-05 |
| STRN4 | 1.266744 | 3.70E-24 | 4.31E-23 |
| GLMN | 1.465461 | 1.09E-25 | 2.19E-24 |
| C6orf52 | 1.663067 | 5.45E-05 | 7.42E-05 |
| COG5 | 1.224314 | 1.11E-17 | 4.09E-17 |
| ARL16 | 1.807807 | 5.05E-27 | 2.15E-25 |
| AHSA1 | 1.03921 | 3.10E-24 | 3.69E-23 |
| ABCD1 | 1.629157 | 5.05E-21 | 2.92E-20 |
| MUC13 | 5.711279 | 1.22E-12 | 2.78E-12 |
| SNX8 | 1.386234 | 1.28E-22 | 1.01E-21 |
| TMEM74B | 2.110027 | 4.37E-12 | 9.52E-12 |
| ZNF234 | 1.258451 | 5.59E-17 | 1.89E-16 |
| AGAP4 | 1.518505 | 3.75E-16 | 1.16E-15 |
| NRG2 | 2.879786 | 1.43E-09 | 2.62E-09 |
| FAM131A | 1.030616 | 6.76E-14 | 1.70E-13 |
| CENPO | 2.305563 | 2.58E-23 | 2.40E-22 |
| PLEKHS1 | 4.74155 | 3.59E-05 | 4.95E-05 |
| OXT | -1.01078 | 4.71E-14 | 1.20E-13 |
| TGS1 | 1.020503 | 2.24E-15 | 6.40E-15 |
| EIF3H | 1.259963 | 1.17E-21 | 7.61E-21 |
| NUF2 | 4.992571 | 1.74E-28 | 3.39E-26 |
| TRIB2 | 1.530947 | 7.17E-09 | 1.26E-08 |
| NIPAL2 | 1.577417 | 2.41E-14 | 6.32E-14 |
| MAGEB17 | 7.471722 | 1.62E-13 | 3.96E-13 |
| DUSP6 | -1.08862 | 8.07E-15 | 2.19E-14 |
| FGB | -1.05801 | 1.53E-16 | 4.94E-16 |
| TUBB | 1.234051 | 5.20E-18 | 1.98E-17 |
| DPCD | 1.459087 | 9.14E-21 | 5.05E-20 |
| SPC25 | 3.876645 | 4.19E-27 | 1.90E-25 |
| PPCDC | 1.478258 | 7.68E-25 | 1.12E-23 |
| PTGES2 | 1.211906 | 8.10E-22 | 5.44E-21 |
| RPL13A | 1.103081 | 2.63E-16 | 8.28E-16 |
| SIX2 | 7.312682 | 2.43E-21 | 1.49E-20 |
| CEP41 | 1.481927 | 1.03E-22 | 8.27E-22 |
| NABP2 | 1.576105 | 2.59E-27 | 1.38E-25 |
| DPF2 | 1.285638 | 3.47E-25 | 5.71E-24 |
| CCDC93 | 1.667757 | 1.15E-24 | 1.59E-23 |
| THY1 | 3.130186 | 1.05E-26 | 3.55E-25 |
| NRG1 | -1.28012 | 5.44E-12 | 1.18E-11 |
| AKR1C2 | 1.624406 | 5.01E-06 | 7.29E-06 |
| TUBB2B | 1.464382 | 4.69E-07 | 7.32E-07 |
| RAD21 | 1.323878 | 1.52E-20 | 8.11E-20 |
| MELK | 4.548726 | 4.98E-28 | 4.88E-26 |
| UBE2T | 3.919653 | 6.28E-29 | 2.56E-26 |
| TMEM65 | 1.719861 | 2.68E-16 | 8.44E-16 |
| BORA | 1.770583 | 1.36E-16 | 4.43E-16 |
| COL6A3 | 1.640087 | 0.000103 | 0.000138 |
| ASPG | -1.5029 | 5.59E-17 | 1.89E-16 |
| SGMS2 | -1.06295 | 1.68E-05 | 2.37E-05 |
| EGFL7 | 1.151079 | 2.80E-08 | 4.72E-08 |
| ZSCAN32 | 1.048389 | 5.86E-13 | 1.37E-12 |
| GPATCH3 | 1.126978 | 2.95E-24 | 3.53E-23 |
| TJAP1 | 1.37596 | 1.02E-25 | 2.07E-24 |
| MRPL33 | 1.083884 | 6.30E-22 | 4.32E-21 |
| WDR13 | 1.195075 | 1.03E-15 | 3.04E-15 |
| TMEM154 | -1.18646 | 1.12E-09 | 2.07E-09 |
| B3GALNT1 | 1.745126 | 4.54E-10 | 8.62E-10 |
| TBC1D10B | 1.139149 | 1.37E-23 | 1.38E-22 |
| GPAT2 | 2.394897 | 2.26E-10 | 4.39E-10 |
| C3orf85 | -1.17395 | 5.15E-14 | 1.31E-13 |
| IFI30 | 1.123348 | 1.11E-08 | 1.92E-08 |
| SLC7A10 | 7.127326 | 7.61E-07 | 1.17E-06 |
| RPIA | 1.26502 | 2.63E-24 | 3.19E-23 |
| ASNA1 | 1.248765 | 2.51E-26 | 6.93E-25 |
| ELOC | 1.436664 | 7.84E-26 | 1.68E-24 |
| HSPA4 | 1.230241 | 4.84E-26 | 1.16E-24 |
| MFSD5 | 1.327054 | 8.70E-26 | 1.83E-24 |
| PNKP | 1.093624 | 3.55E-22 | 2.54E-21 |
| FAM53C | 1.01066 | 2.49E-18 | 9.77E-18 |
| MAP3K7CL | 1.275266 | 1.60E-15 | 4.64E-15 |
| CPLX1 | 2.132492 | 1.75E-08 | 3.00E-08 |
| SPPL2B | 1.197367 | 1.01E-23 | 1.05E-22 |
| DCAF13 | 1.689566 | 5.96E-26 | 1.36E-24 |
| KPTN | 1.287573 | 2.33E-22 | 1.71E-21 |
| DTD1 | 1.274509 | 2.02E-12 | 4.53E-12 |
| CBX2 | 3.45998 | 2.15E-24 | 2.70E-23 |
| GTF2H2C | 1.193504 | 1.51E-15 | 4.40E-15 |
| STPG3 | 2.146616 | 4.76E-11 | 9.68E-11 |
| MASTL | 1.283062 | 1.76E-17 | 6.32E-17 |
| RAB3D | 2.289553 | 1.44E-05 | 2.03E-05 |
| LGI2 | 2.241406 | 1.67E-08 | 2.86E-08 |
| CACNG4 | 4.818873 | 0.000971 | 0.001218 |
| RECQL5 | 1.572475 | 2.11E-28 | 3.44E-26 |
| HMGXB4 | 1.036201 | 9.70E-15 | 2.62E-14 |
| S100A4 | 1.156573 | 0.000223 | 0.00029 |
| SLC27A5 | -1.006 | 4.34E-14 | 1.11E-13 |
| EVI5L | 1.242924 | 2.33E-22 | 1.71E-21 |
| GIGYF1 | 1.34464 | 1.08E-24 | 1.50E-23 |
| DYNLRB1 | 1.336418 | 1.02E-25 | 2.07E-24 |
| ZNF461 | 1.259118 | 1.91E-16 | 6.13E-16 |
| PLXNA3 | 2.123267 | 3.59E-15 | 1.01E-14 |
| CPNE7 | 3.306259 | 7.20E-12 | 1.55E-11 |
| TULP3 | 1.403113 | 1.52E-20 | 8.11E-20 |
| LMNB1 | 2.014673 | 3.52E-15 | 9.90E-15 |
| SNRPA | 1.421393 | 6.68E-27 | 2.52E-25 |
| DHX37 | 1.307898 | 2.12E-23 | 2.02E-22 |
| KCNJ14 | 1.706176 | 4.51E-16 | 1.38E-15 |
| MCRIP1 | 1.358349 | 4.89E-16 | 1.49E-15 |
| ADSL | 1.143333 | 6.03E-24 | 6.64E-23 |
| ACSM3 | -1.42463 | 3.51E-21 | 2.09E-20 |
| NAPB | 1.197117 | 6.34E-23 | 5.31E-22 |
| CCT2 | 1.134828 | 2.06E-22 | 1.54E-21 |
| BHLHA15 | 1.590537 | 0.000372 | 0.000479 |
| EMID1 | 1.786732 | 0.000643 | 0.000815 |
| FAAP24 | 2.144748 | 5.73E-26 | 1.33E-24 |
| AQP8 | 4.294749 | 6.46E-08 | 1.06E-07 |
| SOCS7 | 2.236914 | 8.62E-25 | 1.23E-23 |
| DROSHA | 1.296022 | 1.89E-25 | 3.49E-24 |
| FOXN4 | 3.106783 | 0.001048 | 0.001312 |
| KIAA1522 | 2.204647 | 2.91E-24 | 3.50E-23 |
| IFT22 | 1.149063 | 6.00E-19 | 2.53E-18 |
| ENPP2 | 1.125424 | 9.16E-05 | 0.000123 |
| FIGNL2 | 2.311355 | 1.07E-10 | 2.12E-10 |
| ZNF707 | 1.660755 | 2.55E-27 | 1.37E-25 |
| SMOX | 1.426663 | 1.08E-07 | 1.75E-07 |
| PLCB1 | 2.274001 | 4.77E-14 | 1.22E-13 |
| LIX1L | 1.185546 | 4.99E-18 | 1.90E-17 |
| PDGFRB | 1.823863 | 7.33E-17 | 2.45E-16 |
| MAP2K2 | 1.318561 | 3.14E-26 | 8.36E-25 |
| CPLANE1 | 1.126376 | 7.66E-10 | 1.43E-09 |
| KMT2D | 1.202303 | 2.89E-14 | 7.52E-14 |
| C3orf62 | 1.312238 | 1.89E-22 | 1.43E-21 |
| B3GAT3 | 1.199433 | 6.76E-21 | 3.82E-20 |
| MUSTN1 | 2.721978 | 5.56E-07 | 8.64E-07 |
| CAVIN3 | 1.970169 | 5.10E-14 | 1.30E-13 |
| ACE | 1.63276 | 1.28E-15 | 3.76E-15 |
| BIN1 | 1.125208 | 5.63E-16 | 1.71E-15 |
| ALYREF | 1.484935 | 2.33E-21 | 1.43E-20 |
| HRCT1 | 3.41354 | 1.09E-17 | 4.01E-17 |
| PAPLN | 2.080433 | 0.009676 | 0.011292 |
| USP21 | 1.869523 | 1.87E-27 | 1.13E-25 |
| FBP1 | -1.5053 | 4.76E-21 | 2.77E-20 |
| RPS28 | 1.09427 | 1.88E-14 | 4.98E-14 |
| ICK | 1.435149 | 5.55E-13 | 1.30E-12 |
| CHTF18 | 2.430722 | 3.66E-27 | 1.76E-25 |
| ZNF491 | 1.340314 | 7.08E-13 | 1.64E-12 |
| ZNF738 | 2.393354 | 5.76E-12 | 1.24E-11 |
| SLC18B1 | 1.067857 | 1.22E-10 | 2.41E-10 |
| CNN1 | 1.499204 | 5.90E-06 | 8.54E-06 |
| ARMC1 | 1.190801 | 1.68E-23 | 1.65E-22 |
| WRAP73 | 1.137706 | 5.14E-23 | 4.40E-22 |
| FBXL18 | 2.067728 | 4.19E-26 | 1.04E-24 |
| LYNX1 | 1.18437 | 4.36E-05 | 5.97E-05 |
| HLA-DOB | 1.693398 | 3.74E-05 | 5.14E-05 |
| LYPLAL1 | 1.099294 | 2.81E-18 | 1.09E-17 |
| ANLN | 4.486868 | 7.63E-27 | 2.80E-25 |
| ZNF445 | 1.068899 | 2.75E-15 | 7.80E-15 |
| BRICD5 | 2.042355 | 1.54E-21 | 9.79E-21 |
| SBK1 | 1.83025 | 0.000438 | 0.000561 |
| ANO8 | 1.16994 | 3.48E-12 | 7.67E-12 |
| RBM6 | 1.105999 | 1.38E-22 | 1.08E-21 |
| ECM1 | -2.34755 | 4.24E-27 | 1.92E-25 |
| PTH1R | -2.59953 | 1.59E-27 | 1.01E-25 |
| RRM1 | 1.37321 | 1.84E-21 | 1.15E-20 |
| SATB2 | 1.32353 | 2.76E-13 | 6.61E-13 |
| VMAC | 1.276097 | 2.04E-23 | 1.96E-22 |
| PRR12 | 1.37895 | 2.52E-18 | 9.88E-18 |
| LIMK1 | 1.950195 | 7.65E-19 | 3.19E-18 |
| 8-Sep | 1.506213 | 2.93E-22 | 2.12E-21 |
| HOXA10 | 6.42304 | 1.50E-17 | 5.41E-17 |
| ARHGEF37 | 2.096777 | 5.10E-12 | 1.11E-11 |
| ACSS1 | 1.598081 | 1.27E-05 | 1.80E-05 |
| PDE5A | 1.362347 | 1.58E-06 | 2.39E-06 |
| SSNA1 | 1.191539 | 8.70E-22 | 5.79E-21 |
| NELL2 | 1.181521 | 0.003681 | 0.004445 |
| CEP162 | 1.187778 | 2.47E-15 | 7.04E-15 |
| PKN3 | 2.104033 | 1.94E-22 | 1.46E-21 |
| ZNF501 | 1.307136 | 1.15E-06 | 1.75E-06 |
| UPK3B | 1.232122 | 0.003935 | 0.004739 |
| NSF | 1.21428 | 7.64E-26 | 1.65E-24 |
| MTHFR | 1.153877 | 1.64E-16 | 5.29E-16 |
| SF3A2 | 1.618025 | 1.03E-25 | 2.09E-24 |
| C3orf18 | 1.387996 | 3.13E-15 | 8.83E-15 |
| ABHD17A | 1.115125 | 5.27E-23 | 4.51E-22 |
| PARD6G | 1.376478 | 1.23E-17 | 4.48E-17 |
| SYT3 | 5.462219 | 0.000215 | 0.00028 |
| SAAL1 | 1.264362 | 3.02E-24 | 3.61E-23 |
| FAM219A | 1.453162 | 2.07E-25 | 3.75E-24 |
| C21orf58 | 3.003671 | 5.54E-27 | 2.28E-25 |
| LUC7L3 | 1.199927 | 1.32E-21 | 8.47E-21 |
| RAD9A | 1.641794 | 4.30E-27 | 1.94E-25 |
| WNT3A | 8.534663 | 1.29E-12 | 2.93E-12 |
| DYNC1H1 | 1.213763 | 1.99E-23 | 1.92E-22 |
| BAIAP2L2 | 3.655664 | 2.44E-19 | 1.09E-18 |
| CSAG3 | 2.154335 | 0.004488 | 0.005379 |
| STX3 | 1.084851 | 2.65E-07 | 4.20E-07 |
| SIGLEC15 | 2.203803 | 0.006579 | 0.007777 |
| DNAJB2 | 1.158681 | 8.45E-24 | 8.95E-23 |
| CLEC4M | -5.20415 | 2.57E-30 | 1.08E-26 |
| TBCC | 1.393352 | 1.42E-25 | 2.74E-24 |
| NAV1 | 1.303067 | 1.72E-10 | 3.37E-10 |
| SCPEP1 | 1.169523 | 5.91E-05 | 8.02E-05 |
| MARCKSL1 | 1.347308 | 1.46E-07 | 2.35E-07 |
| ADH1B | -1.2556 | 3.38E-17 | 1.18E-16 |
| BAIAP2L1 | 1.102554 | 4.97E-12 | 1.08E-11 |
| CAMK2B | -1.0205 | 3.22E-13 | 7.68E-13 |
| RPS27A | 1.103745 | 1.67E-18 | 6.65E-18 |
| METTL2A | 1.016391 | 7.19E-24 | 7.80E-23 |
| PIH1D1 | 1.015295 | 4.30E-22 | 3.03E-21 |
| IKBKE | 1.86881 | 1.30E-11 | 2.74E-11 |
| OPN1SW | 1.136002 | 1.98E-09 | 3.60E-09 |
| DNAJC2 | 1.050556 | 8.43E-21 | 4.68E-20 |
| EXOC4 | 1.004021 | 1.14E-24 | 1.57E-23 |
| HYPK | 1.154382 | 7.59E-21 | 4.25E-20 |
| EPHB2 | 3.152881 | 3.73E-08 | 6.24E-08 |
| STXBP4 | 1.800198 | 1.58E-20 | 8.37E-20 |
| ASPM | 4.475134 | 1.98E-27 | 1.16E-25 |
| ZKSCAN8 | 1.166311 | 1.24E-13 | 3.06E-13 |
| SGO1 | 3.830696 | 1.76E-26 | 5.31E-25 |
| SCGB2A1 | 3.738094 | 0.002238 | 0.002746 |
| TMCO1 | 1.222555 | 1.23E-27 | 8.36E-26 |
| PTK2 | 1.354811 | 1.97E-23 | 1.90E-22 |
| RCBTB1 | 1.102564 | 4.33E-12 | 9.44E-12 |
| APBB3 | 1.326294 | 1.99E-16 | 6.36E-16 |
| B4GALT7 | 1.404302 | 4.79E-27 | 2.07E-25 |
| RNF144A | 1.920898 | 2.15E-13 | 5.21E-13 |
| CCDC13 | 3.398722 | 0.004319 | 0.005183 |
| GPM6A | -2.55777 | 6.42E-26 | 1.44E-24 |
| KRT23 | 2.135864 | 0.001254 | 0.001561 |
| CYREN | 1.092456 | 3.17E-18 | 1.23E-17 |
| ZNF703 | 2.051677 | 5.63E-09 | 9.93E-09 |
| KIAA0895L | 1.559153 | 8.26E-18 | 3.07E-17 |
| CCDC102B | 1.958172 | 7.90E-20 | 3.76E-19 |
| PIK3R3 | 1.280855 | 1.90E-18 | 7.53E-18 |
| PYGO2 | 1.923413 | 4.38E-29 | 2.48E-26 |
| ALOX15B | 3.677623 | 7.72E-07 | 1.19E-06 |
| CKB | 2.716322 | 3.20E-06 | 4.71E-06 |
| C7orf43 | 1.026991 | 4.77E-18 | 1.82E-17 |
| SMPD4 | 1.253396 | 2.14E-22 | 1.58E-21 |
| MSTO1 | 2.345521 | 4.75E-30 | 1.08E-26 |
| SYNJ2 | 1.211037 | 8.08E-10 | 1.51E-09 |
| SLC26A11 | 1.400423 | 2.92E-23 | 2.68E-22 |
| VEGFD | 3.201947 | 1.79E-06 | 2.69E-06 |
| MBLAC1 | 1.448525 | 5.11E-21 | 2.96E-20 |
| CCDC97 | 1.455065 | 3.62E-26 | 9.34E-25 |
| PARP10 | 1.007382 | 2.13E-13 | 5.17E-13 |
| KIAA1211L | 1.982843 | 1.15E-17 | 4.22E-17 |
| HYLS1 | 1.031895 | 4.03E-14 | 1.03E-13 |
| MESP1 | 2.596058 | 3.18E-24 | 3.76E-23 |
| EXOSC8 | 1.018779 | 2.47E-21 | 1.51E-20 |
| ORC6 | 3.614602 | 1.73E-26 | 5.25E-25 |
| COL5A1 | 1.697205 | 0.003168 | 0.003845 |
| 5-Sep | 2.746554 | 7.65E-19 | 3.19E-18 |
| RNF125 | -1.27947 | 1.48E-17 | 5.36E-17 |
| NXPH4 | 6.404468 | 3.84E-26 | 9.76E-25 |
| TPD52L2 | 1.471075 | 6.71E-28 | 5.68E-26 |
| ISX | 7.767258 | 7.97E-13 | 1.84E-12 |
| ESM1 | 4.918761 | 2.23E-27 | 1.27E-25 |
| MYO1A | 3.73322 | 2.78E-14 | 7.25E-14 |
| LILRA5 | -1.55112 | 1.99E-16 | 6.36E-16 |
| REG1A | 10.79379 | 2.49E-08 | 4.22E-08 |
| ESS2 | 1.158696 | 4.03E-26 | 1.01E-24 |
| TRAF1 | 1.229061 | 7.11E-15 | 1.94E-14 |
| TRABD2A | 2.001767 | 0.030996 | 0.034972 |
| GREB1 | 2.130265 | 8.75E-05 | 0.000117 |
| NOMO1 | 1.063951 | 1.52E-21 | 9.68E-21 |
| PLN | 2.205317 | 0.028501 | 0.032229 |
| FXYD3 | 3.717891 | 0.014746 | 0.016982 |
| ZNF783 | 1.57962 | 4.69E-16 | 1.44E-15 |
| IL2RG | 1.224042 | 0.017047 | 0.019557 |
| MAST2 | 1.756194 | 2.86E-26 | 7.69E-25 |
| GYS2 | -1.62004 | 4.34E-21 | 2.54E-20 |
| CRYGS | 2.855079 | 2.15E-23 | 2.04E-22 |
| TRAF3 | 1.193726 | 8.77E-16 | 2.61E-15 |
| CC2D1B | 1.076225 | 4.26E-25 | 6.77E-24 |
| EHF | 2.304048 | 0.0108 | 0.012552 |
| LARP4B | 1.105855 | 1.58E-22 | 1.22E-21 |
| ASPH | 1.644842 | 1.55E-12 | 3.51E-12 |
| LSM4 | 1.618974 | 5.69E-27 | 2.31E-25 |
| SLC25A43 | 1.129382 | 1.71E-16 | 5.50E-16 |
| CD5L | -2.50617 | 5.66E-22 | 3.90E-21 |
| DNAJC6 | 3.285802 | 1.97E-21 | 1.23E-20 |
| SLC7A1 | 1.678059 | 2.47E-05 | 3.44E-05 |
| SAC3D1 | 1.923429 | 6.95E-27 | 2.61E-25 |
| CORO7 | 1.007778 | 3.30E-12 | 7.30E-12 |
| PARD3B | 1.142182 | 5.40E-07 | 8.39E-07 |
| IGSF21 | 1.48079 | 9.58E-10 | 1.78E-09 |
| DDX59 | 1.196598 | 1.73E-20 | 9.13E-20 |
| ATP9A | 1.267198 | 3.84E-11 | 7.86E-11 |
| RANGAP1 | 1.094042 | 1.45E-14 | 3.87E-14 |
| HIST1H1C | 2.007247 | 8.93E-12 | 1.90E-11 |
| ZNF518A | 1.105539 | 1.25E-10 | 2.47E-10 |
| DNMT3A | 1.996224 | 7.94E-24 | 8.47E-23 |
| BBOX1 | -1.1596 | 4.39E-13 | 1.03E-12 |
| PLEKHH3 | 1.064428 | 3.51E-12 | 7.74E-12 |
| EIF3K | 1.09647 | 2.20E-23 | 2.09E-22 |
| KIF11 | 2.991762 | 9.53E-26 | 1.98E-24 |
| PKD2L1 | 1.43425 | 0.013964 | 0.016101 |
| PIEZO1 | 1.08069 | 4.86E-14 | 1.24E-13 |
| AC068580.4 | 2.131111 | 2.70E-12 | 6.01E-12 |
| COG2 | 1.688498 | 1.52E-28 | 3.39E-26 |
| TRIM24 | 1.210737 | 1.61E-16 | 5.19E-16 |
| GPR137 | 1.210761 | 2.40E-23 | 2.25E-22 |
| CASP8 | 1.040119 | 1.52E-15 | 4.42E-15 |
| PCDHB8 | 3.271997 | 0.000486 | 0.000621 |
| CCDC88A | 1.364855 | 7.28E-14 | 1.83E-13 |
| SLCO3A1 | 1.107386 | 0.001501 | 0.001859 |
| LBHD1 | 1.395052 | 2.17E-21 | 1.34E-20 |
| PELI3 | 1.166192 | 2.23E-12 | 4.98E-12 |
| TNFSF15 | 2.620403 | 6.61E-09 | 1.16E-08 |
| RING1 | 1.074018 | 6.73E-23 | 5.60E-22 |
| FAM133A | 6.140632 | 0.002221 | 0.002725 |
| UBAP1L | 1.022184 | 2.64E-09 | 4.75E-09 |
| DIRAS1 | 4.288859 | 1.75E-08 | 2.99E-08 |
| CEP164 | 1.225897 | 4.44E-21 | 2.59E-20 |
| CIT | 1.237734 | 3.44E-07 | 5.41E-07 |
| UGT1A10 | 6.060761 | 0.011635 | 0.013496 |
| SPIN3 | 1.238345 | 1.96E-17 | 6.98E-17 |
| DUSP22 | 1.027581 | 4.72E-18 | 1.80E-17 |
| FGFR3 | 1.448762 | 3.35E-06 | 4.94E-06 |
| ZNF765 | 1.05034 | 9.08E-12 | 1.93E-11 |
| HOOK2 | 1.061539 | 7.03E-10 | 1.32E-09 |
| TELO2 | 1.332355 | 1.52E-26 | 4.76E-25 |
| RIN1 | 1.207361 | 2.81E-07 | 4.45E-07 |
| SLIT3 | 1.044996 | 0.007878 | 0.009263 |
| SNORC | 2.245932 | 7.42E-07 | 1.14E-06 |
| SEZ6L2 | 4.004238 | 3.10E-06 | 4.58E-06 |
| CENPU | 2.916942 | 8.09E-25 | 1.16E-23 |
| AAGAB | 1.080898 | 2.47E-24 | 3.04E-23 |
| TM4SF20 | 8.7715 | 1.96E-06 | 2.93E-06 |
| UBE2Z | 1.050151 | 2.61E-20 | 1.34E-19 |
| PERM1 | 2.150757 | 8.15E-08 | 1.33E-07 |
| CYHR1 | 1.507576 | 7.23E-27 | 2.69E-25 |
| HLTF | 1.257634 | 1.87E-15 | 5.41E-15 |
| HAS3 | 1.273829 | 1.02E-12 | 2.34E-12 |
| CSNK1E | 1.369031 | 8.20E-22 | 5.49E-21 |
| PPP2R5A | 1.082701 | 2.90E-20 | 1.48E-19 |
| C12orf75 | 3.021155 | 2.07E-09 | 3.76E-09 |
| SLC35B1 | 1.011961 | 1.35E-26 | 4.33E-25 |
| IL32 | 1.304268 | 2.19E-07 | 3.49E-07 |
| ZC3H8 | 1.297371 | 2.04E-22 | 1.52E-21 |
| GSTCD | 1.146097 | 1.96E-10 | 3.82E-10 |
| EML2 | 1.201687 | 1.28E-16 | 4.18E-16 |
| EFCAB11 | 1.117862 | 1.70E-19 | 7.75E-19 |
| NSMAF | 1.192162 | 5.05E-14 | 1.29E-13 |
| RFWD3 | 1.204333 | 7.80E-17 | 2.60E-16 |
| BCL9 | 2.079736 | 2.14E-22 | 1.58E-21 |
| RNF113A | 1.084223 | 8.19E-23 | 6.69E-22 |
| CHEK1 | 2.43282 | 3.85E-25 | 6.21E-24 |
| CELSR3 | 4.16243 | 2.23E-27 | 1.27E-25 |
| PPP2R5D | 1.190077 | 1.69E-21 | 1.07E-20 |
| PCSK1 | 7.398097 | 4.22E-05 | 5.79E-05 |
| BPGM | 1.296445 | 2.04E-24 | 2.58E-23 |
| EPRS | 1.454478 | 1.37E-25 | 2.66E-24 |
| NME2 | 1.486551 | 1.71E-21 | 1.08E-20 |
| TMEM91 | 1.489318 | 9.16E-12 | 1.95E-11 |
| SLC2A4 | 1.021931 | 0.001768 | 0.002182 |
| DNAAF5 | 1.250569 | 1.29E-23 | 1.30E-22 |
| ANO10 | 1.458579 | 1.52E-22 | 1.18E-21 |
| WIPF2 | 1.029425 | 2.13E-19 | 9.55E-19 |
| SYCE3 | 1.418873 | 4.60E-08 | 7.64E-08 |
| CHN1 | 1.861162 | 2.09E-13 | 5.08E-13 |
| INTS1 | 1.187528 | 1.59E-23 | 1.57E-22 |
| RGS19 | 1.115865 | 5.15E-10 | 9.74E-10 |
| RNF216 | 1.18256 | 6.26E-23 | 5.25E-22 |
| UBE2Q1 | 1.427909 | 4.06E-28 | 4.50E-26 |
| CYTH2 | 1.248288 | 7.25E-23 | 5.99E-22 |
| CDK5RAP3 | 1.395557 | 2.35E-26 | 6.64E-25 |
| UNKL | 1.731943 | 3.95E-20 | 1.96E-19 |
| BACH2 | -1.01294 | 4.58E-09 | 8.12E-09 |
| HAUS3 | 1.217954 | 3.09E-21 | 1.86E-20 |
| RNF213 | 1.434443 | 7.08E-19 | 2.96E-18 |
| ATP6V0D2 | 3.572125 | 3.37E-12 | 7.43E-12 |
| LPIN3 | 1.085182 | 5.90E-11 | 1.19E-10 |
| BORCS6 | 1.279262 | 3.00E-22 | 2.16E-21 |
| RPLP2 | 1.286205 | 7.65E-19 | 3.19E-18 |
| PDPK1 | 1.042065 | 2.73E-14 | 7.12E-14 |
| ADH1C | -1.02837 | 1.14E-12 | 2.60E-12 |
| ZNF343 | 1.08902 | 1.47E-20 | 7.88E-20 |
| CALCRL | 1.108424 | 1.12E-05 | 1.60E-05 |
| PCDHB10 | 2.018246 | 1.27E-06 | 1.93E-06 |
| COPS7B | 1.319807 | 1.42E-25 | 2.74E-24 |
| ATP11A | 1.084226 | 1.55E-08 | 2.67E-08 |
| IFI27L2 | 1.57519 | 1.70E-07 | 2.73E-07 |
| TNNI2 | 3.153282 | 1.57E-11 | 3.29E-11 |
| IRX3 | 3.192421 | 9.73E-09 | 1.69E-08 |
| SKIV2L | 1.150515 | 3.21E-25 | 5.38E-24 |
| MGP | 1.700149 | 9.11E-05 | 0.000122 |
| HHAT | 1.499228 | 4.28E-15 | 1.19E-14 |
| MRPL55 | 1.27363 | 8.95E-20 | 4.23E-19 |
| CYP2R1 | 1.377086 | 5.30E-26 | 1.25E-24 |
| HIST1H4H | 3.259177 | 7.46E-18 | 2.79E-17 |
| ZNF416 | 1.076864 | 3.22E-15 | 9.08E-15 |
| ZNF431 | 1.841018 | 7.92E-12 | 1.69E-11 |
| BRIP1 | 2.144205 | 7.25E-17 | 2.43E-16 |
| PTP4A3 | 3.144288 | 7.43E-23 | 6.10E-22 |
| FAM183A | 2.885383 | 4.93E-05 | 6.73E-05 |
| TACC3 | 2.82497 | 7.44E-26 | 1.63E-24 |
| SPIN4 | 1.339772 | 3.60E-08 | 6.03E-08 |
| AFP | 7.584383 | 2.40E-06 | 3.56E-06 |
| NUPR1 | 1.676208 | 5.55E-14 | 1.41E-13 |
| CCDC18 | 1.16203 | 2.95E-12 | 6.54E-12 |
| HDAC10 | 1.181122 | 8.46E-20 | 4.01E-19 |
| IFT81 | 1.549469 | 3.65E-18 | 1.41E-17 |
| CEP135 | 1.039948 | 6.49E-08 | 1.07E-07 |
| CNDP1 | -2.67904 | 1.45E-22 | 1.13E-21 |
| RFX5 | 1.812846 | 2.23E-23 | 2.11E-22 |
| MCTP1 | 2.173651 | 2.16E-10 | 4.19E-10 |
| SPACA9 | 1.233944 | 3.19E-16 | 9.95E-16 |
| CCDC80 | 2.410829 | 0.002561 | 0.003128 |
| CMTM4 | 2.059924 | 2.85E-19 | 1.26E-18 |
| LRR1 | 1.353793 | 1.06E-22 | 8.45E-22 |
| SLC10A3 | 1.183558 | 2.34E-13 | 5.64E-13 |
| IGF2BP1 | 7.666682 | 2.86E-16 | 8.96E-16 |
| NDUFAF2 | 1.29862 | 5.89E-23 | 4.97E-22 |
| NAA80 | 1.165829 | 1.23E-18 | 4.98E-18 |
| CAMSAP2 | 1.327841 | 2.77E-16 | 8.69E-16 |
| RALGPS1 | 1.47108 | 1.92E-06 | 2.86E-06 |
| AXIN2 | 2.770207 | 0.02806 | 0.031751 |
| GLRX3 | 1.131854 | 7.68E-25 | 1.12E-23 |
| PMVK | 1.26893 | 1.54E-22 | 1.19E-21 |
| DGKQ | 1.467654 | 4.12E-23 | 3.60E-22 |
| RPL3L | 1.031604 | 0.000496 | 0.000633 |
| TIMM17B | 1.163375 | 3.02E-24 | 3.61E-23 |
| INKA2 | 1.900915 | 1.70E-22 | 1.30E-21 |
| LMOD1 | 1.832043 | 5.58E-05 | 7.59E-05 |
| VN1R1 | 2.065507 | 4.05E-11 | 8.28E-11 |
| MEA1 | 1.431325 | 3.18E-28 | 4.01E-26 |
| NUDCD3 | 1.0028 | 1.28E-24 | 1.73E-23 |
| ZBED6CL | 1.759862 | 4.93E-17 | 1.68E-16 |
| PECAM1 | 1.268413 | 1.39E-17 | 5.06E-17 |
| COL4A1 | 2.44597 | 1.76E-22 | 1.34E-21 |
| SHANK3 | 1.133036 | 3.13E-15 | 8.83E-15 |
| KLHL12 | 1.487913 | 5.54E-27 | 2.28E-25 |
| BZW2 | 1.369898 | 6.45E-21 | 3.67E-20 |
| FAM124B | 1.52 | 5.85E-05 | 7.94E-05 |
| COL1A2 | 2.148969 | 2.69E-06 | 3.99E-06 |
| SELENOM | 2.364467 | 2.81E-07 | 4.45E-07 |
| CEP95 | 1.272484 | 1.56E-21 | 9.90E-21 |
| TREM2 | 2.160639 | 4.91E-13 | 1.15E-12 |
| INPP5J | 2.90448 | 2.70E-11 | 5.59E-11 |
| KIF3A | 1.352922 | 5.19E-19 | 2.20E-18 |
| GRAMD1A | 2.13779 | 3.56E-20 | 1.78E-19 |
| DIPK1B | 1.576746 | 1.31E-12 | 2.98E-12 |
| TMEM184A | 1.091307 | 2.22E-07 | 3.53E-07 |
| DIRAS3 | -2.21429 | 2.04E-22 | 1.52E-21 |
| SAYSD1 | 1.236382 | 8.39E-23 | 6.84E-22 |
| CAPG | 1.920016 | 1.95E-11 | 4.07E-11 |
| GAL | 3.707689 | 0.015201 | 0.017491 |
| FOXP4 | 1.171554 | 4.06E-18 | 1.56E-17 |
| THUMPD2 | 1.128419 | 3.11E-19 | 1.37E-18 |
| SEC61G | 1.228938 | 1.45E-21 | 9.27E-21 |
| ARHGAP11A | 3.305468 | 1.02E-25 | 2.07E-24 |
| CASC3 | 1.314276 | 3.95E-25 | 6.33E-24 |
| APEX1 | 1.035256 | 1.71E-24 | 2.23E-23 |
| NPAS1 | 1.197236 | 0.002677 | 0.003265 |
| NAALADL1 | 2.955201 | 2.17E-08 | 3.69E-08 |
| CYP27B1 | 2.279369 | 3.87E-16 | 1.20E-15 |
| YBEY | 1.029177 | 5.34E-14 | 1.36E-13 |
| PPP1CC | 1.167483 | 5.88E-24 | 6.50E-23 |
| SLC22A1 | -1.87444 | 4.38E-20 | 2.16E-19 |
| BOP1 | 2.24713 | 2.38E-26 | 6.72E-25 |
| MFF | 1.026301 | 2.48E-25 | 4.37E-24 |
| UGT2B10 | -1.09718 | 9.18E-13 | 2.11E-12 |
| KIAA1841 | 2.254967 | 8.04E-26 | 1.72E-24 |
| TMEM61 | 3.210433 | 0.000139 | 0.000185 |
| SPATC1L | 3.588429 | 1.84E-18 | 7.31E-18 |
| TRIM11 | 1.801718 | 3.34E-27 | 1.64E-25 |
| SNX27 | 1.456488 | 2.55E-23 | 2.37E-22 |
| JPH1 | 2.604989 | 0.00145 | 0.001798 |
| SLC2A6 | 1.75661 | 9.57E-08 | 1.56E-07 |
| RNF24 | 1.738009 | 1.02E-18 | 4.18E-18 |
| TMEM216 | 1.032326 | 1.26E-15 | 3.69E-15 |
| VASH1 | 1.045509 | 2.47E-08 | 4.18E-08 |
| MTG1 | 1.313181 | 1.31E-19 | 6.06E-19 |
| HSF2BP | 2.862761 | 2.74E-21 | 1.66E-20 |
| ELOVL1 | 1.452884 | 2.75E-26 | 7.42E-25 |
| EFCAB7 | 1.28529 | 4.50E-15 | 1.25E-14 |
| BTN3A1 | 1.091148 | 3.39E-11 | 6.96E-11 |
| SYP | 2.423462 | 3.99E-20 | 1.98E-19 |
| NOL3 | 1.509725 | 1.76E-13 | 4.29E-13 |
| MED12 | 1.313723 | 2.94E-18 | 1.14E-17 |
| C2orf15 | 1.739171 | 2.46E-06 | 3.65E-06 |
| UPF3B | 1.581086 | 6.34E-25 | 9.57E-24 |
| MAPK8IP3 | 1.673693 | 3.11E-19 | 1.37E-18 |
| MZT1 | 1.497052 | 2.37E-23 | 2.23E-22 |
| RAB42 | 1.156033 | 0.031966 | 0.036044 |
| FAM168A | 1.025981 | 2.60E-16 | 8.21E-16 |
| HNRNPA1P48 | 1.290479 | 4.72E-18 | 1.80E-17 |
| SMG7 | 1.19482 | 1.97E-24 | 2.51E-23 |
| MICALL2 | 1.122236 | 5.92E-09 | 1.04E-08 |
| KIF2C | 4.730565 | 2.11E-28 | 3.44E-26 |
| XYLT2 | 1.273815 | 1.03E-23 | 1.07E-22 |
| AGBL3 | 1.602136 | 5.19E-20 | 2.54E-19 |
| PCDHB2 | 3.668034 | 1.78E-09 | 3.25E-09 |
| SMO | 1.464086 | 2.09E-14 | 5.51E-14 |
| SEMA3F | 1.920567 | 8.94E-30 | 1.28E-26 |
| CHST2 | 1.242356 | 3.79E-07 | 5.95E-07 |
| AMN1 | 1.122859 | 3.59E-15 | 1.01E-14 |
| RFC1 | 1.137098 | 1.75E-20 | 9.22E-20 |
| TIMELESS | 1.482188 | 6.76E-21 | 3.82E-20 |
| ENOPH1 | 1.090793 | 2.78E-21 | 1.68E-20 |
| EMD | 1.024537 | 2.49E-23 | 2.32E-22 |
| RIOX1 | 1.169021 | 1.04E-23 | 1.08E-22 |
| YIF1B | 1.285194 | 6.18E-23 | 5.20E-22 |
| PTPRF | 1.004003 | 5.86E-12 | 1.27E-11 |
| FANCA | 1.69201 | 3.77E-18 | 1.45E-17 |
| RACGAP1 | 3.18134 | 2.52E-28 | 3.60E-26 |
| TARBP1 | 2.479683 | 3.52E-27 | 1.70E-25 |
| STX10 | 1.511015 | 2.84E-27 | 1.47E-25 |
| FTL | 1.012826 | 7.28E-07 | 1.12E-06 |
| NCBP2-AS2 | 1.012706 | 3.89E-18 | 1.50E-17 |
| MAP1A | 2.511103 | 3.30E-11 | 6.79E-11 |
| ZFP41 | 2.55577 | 9.04E-28 | 7.01E-26 |
| SIGLEC11 | -1.73807 | 7.91E-18 | 2.95E-17 |
| RNF44 | 1.443264 | 2.83E-20 | 1.45E-19 |
| ERCC6L | 3.991403 | 1.46E-26 | 4.59E-25 |
| KCNJ11 | 2.44069 | 1.13E-09 | 2.10E-09 |
| BUD13 | 1.11081 | 1.08E-24 | 1.50E-23 |
| GPRIN1 | 3.263582 | 6.69E-22 | 4.56E-21 |
| FAM228B | 1.274724 | 9.68E-13 | 2.22E-12 |
| LARGE2 | 2.017166 | 0.007907 | 0.009295 |
| TROAP | 4.960045 | 2.14E-28 | 3.44E-26 |
| GMNN | 2.885139 | 2.97E-28 | 3.94E-26 |
| UAP1L1 | 2.109915 | 0.00029 | 0.000376 |
| SHISA4 | 2.041618 | 3.50E-13 | 8.31E-13 |
| LPA | -1.80332 | 2.38E-22 | 1.75E-21 |
| C1QTNF6 | 1.888611 | 2.53E-21 | 1.54E-20 |
| SNRPF | 1.074997 | 1.41E-17 | 5.11E-17 |
| MUTYH | 1.216906 | 3.59E-21 | 2.14E-20 |
| ZNF514 | 1.548717 | 1.58E-20 | 8.37E-20 |
| TMEM163 | 3.981692 | 0.044589 | 0.049712 |
| FGFBP3 | 1.176855 | 2.87E-10 | 5.52E-10 |
| RPS17 | 1.007731 | 4.32E-15 | 1.20E-14 |
| LSM2 | 1.534928 | 2.24E-25 | 4.02E-24 |
| GNRH1 | 1.706132 | 1.05E-16 | 3.47E-16 |
| PSRC1 | 2.351615 | 8.08E-20 | 3.84E-19 |
| NPIPA1 | 1.402588 | 8.90E-16 | 2.65E-15 |
| NPM1 | 1.392806 | 3.94E-24 | 4.54E-23 |
| PRRX1 | 3.191998 | 5.61E-18 | 2.13E-17 |
| ECE2 | 1.041839 | 4.68E-17 | 1.60E-16 |
| CCDC137 | 1.678704 | 3.21E-27 | 1.59E-25 |
| ATP5MC1 | 1.309169 | 1.67E-21 | 1.06E-20 |
| C3orf33 | 1.061915 | 1.54E-18 | 6.18E-18 |
| P3H4 | 2.276457 | 2.47E-20 | 1.27E-19 |
| SLC45A4 | 2.673021 | 2.76E-13 | 6.61E-13 |
| PTMA | 1.082153 | 2.01E-22 | 1.51E-21 |
| AHNAK2 | 2.954522 | 0.031477 | 0.035506 |
| GLRX2 | 1.015892 | 9.59E-18 | 3.55E-17 |
| PNMA1 | 1.651036 | 1.63E-14 | 4.33E-14 |
| UQCRHL | 1.347824 | 1.05E-19 | 4.89E-19 |
| PDCD2L | 1.491776 | 9.00E-24 | 9.47E-23 |
| A4GALT | 1.644272 | 2.15E-12 | 4.81E-12 |
| CDC6 | 3.976295 | 4.60E-27 | 2.01E-25 |
| MRPS12 | 1.347125 | 2.23E-23 | 2.11E-22 |
| H2AFY2 | 1.654202 | 0.001349 | 0.001677 |
| ZCCHC9 | 1.100154 | 9.25E-21 | 5.10E-20 |
| GINS4 | 2.541841 | 4.88E-17 | 1.66E-16 |
| THRA | 1.238256 | 3.46E-16 | 1.08E-15 |
| VAMP1 | 1.673837 | 6.74E-20 | 3.24E-19 |
| NT5DC3 | 1.135672 | 8.10E-13 | 1.87E-12 |
| HAMP | -3.21486 | 6.51E-25 | 9.77E-24 |
| SARNP | 1.112607 | 1.34E-18 | 5.41E-18 |
| PARP1 | 1.349487 | 6.82E-23 | 5.67E-22 |
| TSTA3 | 1.206223 | 9.59E-18 | 3.55E-17 |
| CSNK1D | 1.125657 | 2.02E-23 | 1.94E-22 |
| XRCC2 | 3.676544 | 2.24E-25 | 4.02E-24 |
| NUP85 | 1.215794 | 2.02E-24 | 2.55E-23 |
| SMPD2 | 1.234533 | 1.20E-19 | 5.56E-19 |
| LGI4 | 1.355988 | 0.002561 | 0.003128 |
| TANGO2 | 1.10757 | 1.24E-17 | 4.53E-17 |
| APOBEC3H | 1.173997 | 2.21E-07 | 3.51E-07 |
| SYNGR4 | 2.951499 | 9.67E-12 | 2.06E-11 |
| ZFP69 | 1.084124 | 5.16E-15 | 1.42E-14 |
| GNB5 | 1.226244 | 1.71E-13 | 4.18E-13 |
| POLG2 | 1.679248 | 1.25E-26 | 4.10E-25 |
| NUMBL | 1.229476 | 2.72E-10 | 5.23E-10 |
| UCKL1 | 1.181616 | 1.80E-23 | 1.76E-22 |
| SLC22A12 | 5.633947 | 0.000676 | 0.000856 |
| NAT9 | 1.864681 | 9.24E-29 | 2.81E-26 |
| COL5A2 | 1.63953 | 4.06E-09 | 7.23E-09 |
| RPS8 | 1.06882 | 1.54E-16 | 4.98E-16 |
| HIST1H2BK | 1.611587 | 3.66E-14 | 9.46E-14 |
| LRFN1 | 1.704971 | 0.000469 | 0.0006 |
| SSBP4 | 1.256404 | 1.19E-20 | 6.48E-20 |
| MSL1 | 1.00838 | 1.56E-20 | 8.29E-20 |
| NVL | 1.672319 | 5.05E-28 | 4.88E-26 |
| CYP21A2 | 1.075816 | 7.70E-05 | 0.000104 |
| CSPP1 | 1.477599 | 1.41E-16 | 4.56E-16 |
| GPATCH1 | 1.30981 | 8.15E-26 | 1.73E-24 |
| TNFSF4 | 2.42407 | 1.01E-18 | 4.14E-18 |
| ATP1A1 | 1.374697 | 2.76E-14 | 7.19E-14 |
| RBM4 | 1.08399 | 4.00E-17 | 1.38E-16 |
| POC5 | 1.352801 | 1.58E-22 | 1.22E-21 |

DE differentially expressed, HCC hepatocellular carcinoma, FC Fold Change, FDR false discovery rate
