# Supplementary material for: Identification of a five-immune gene model as an independent prognostic factor in hepatocellular carcinoma
Source: BMC Cancer. 2021 Mar 16;21:278. doi: 10.1186/s12885-021-08012-2 (PMC7962305; doi:10.1186/s12885-021-08012-2)
Supplement: Supplementary file 3 — Additional file 3: Table S3. DE immune genes in HCC [file 12885_2021_8012_MOESM3_ESM.docx]

**Table S3** DE immune genes in HCC

| ID | logFC | pValue | FDR |
| --- | --- | --- | --- |
| AZGP1 | -1.06248 | 2.14E-18 | 8.44E-18 |
| CALR | 1.016986 | 1.05E-19 | 4.89E-19 |
| CANX | 1.102288 | 2.00E-21 | 1.24E-20 |
| CD1D | -1.00811 | 3.35E-15 | 9.43E-15 |
| CD4 | -1.2519 | 1.84E-18 | 7.31E-18 |
| HLA-DMA | 1.330973 | 4.11E-05 | 5.64E-05 |
| HLA-DOB | 1.693398 | 3.74E-05 | 5.14E-05 |
| HLA-F | 1.175781 | 1.95E-08 | 3.33E-08 |
| HLA-G | 1.163789 | 1.18E-07 | 1.91E-07 |
| HSPA1A | 1.410242 | 5.02E-12 | 1.09E-11 |
| HSPA1B | 1.331347 | 4.69E-08 | 7.79E-08 |
| HSPA1L | 1.0444 | 1.89E-13 | 4.61E-13 |
| HSPA2 | 1.02681 | 5.10E-07 | 7.93E-07 |
| HSPA4 | 1.230241 | 4.84E-26 | 1.16E-24 |
| HSPA5 | 1.071416 | 1.04E-19 | 4.84E-19 |
| HSPA6 | 1.931063 | 0.000793 | 0.001001 |
| HSP90AA1 | 1.057758 | 6.55E-19 | 2.75E-18 |
| HSP90AB1 | 1.634118 | 3.53E-26 | 9.14E-25 |
| LTA | 1.275401 | 1.78E-05 | 2.51E-05 |
| CIITA | 1.070709 | 1.61E-05 | 2.27E-05 |
| MICA | 1.430866 | 1.49E-20 | 7.96E-20 |
| MICB | 1.915426 | 1.08E-15 | 3.20E-15 |
| NFYA | 1.330314 | 5.49E-19 | 2.32E-18 |
| PSMC4 | 1.293546 | 1.27E-28 | 3.14E-26 |
| PSMD2 | 1.147132 | 1.11E-24 | 1.54E-23 |
| PSMD4 | 1.708901 | 2.02E-28 | 3.44E-26 |
| PSMD10 | 1.361273 | 5.51E-26 | 1.29E-24 |
| RELB | 1.170748 | 1.19E-11 | 2.52E-11 |
| RFX5 | 1.812846 | 2.23E-23 | 2.11E-22 |
| TAP1 | 1.544189 | 6.51E-15 | 1.78E-14 |
| TAP2 | 1.246924 | 8.17E-16 | 2.44E-15 |
| THBS1 | -1.01657 | 1.49E-09 | 2.74E-09 |
| AP3B1 | 1.25896 | 2.80E-24 | 3.37E-23 |
| RFXANK | 1.721051 | 1.25E-26 | 4.10E-25 |
| PSME3 | 1.169085 | 7.10E-24 | 7.72E-23 |
| PSMD14 | 1.205251 | 1.99E-24 | 2.53E-23 |
| CLEC4M | -5.20415 | 2.57E-30 | 1.08E-26 |
| IFI30 | 1.123348 | 1.11E-08 | 1.92E-08 |
| ULBP2 | 2.041193 | 0.001139 | 0.001421 |
| PDIA2 | 6.757734 | 1.94E-14 | 5.12E-14 |
| HAMP | -3.21486 | 6.51E-25 | 9.77E-24 |
| PI3 | 2.587422 | 0.000522 | 0.000666 |
| PPBP | -1.42162 | 1.80E-17 | 6.47E-17 |
| CXCL14 | -2.74119 | 1.09E-26 | 3.67E-25 |
| CXCL12 | -2.05117 | 1.08E-24 | 1.50E-23 |
| CCL13 | 2.587983 | 2.62E-06 | 3.89E-06 |
| CCL8 | 1.007602 | 0.00037 | 0.000477 |
| TMSB10 | 1.961784 | 5.91E-12 | 1.28E-11 |
| LCN2 | 5.190369 | 1.91E-10 | 3.73E-10 |
| COLEC10 | -3.4178 | 1.27E-28 | 3.14E-26 |
| S100A12 | -2.35595 | 4.41E-17 | 1.51E-16 |
| CCR10 | 1.953652 | 7.20E-10 | 1.35E-09 |
| MMP12 | 4.928723 | 7.18E-10 | 1.35E-09 |
| TMSB15A | 3.192409 | 1.27E-11 | 2.68E-11 |
| S100A13 | 1.060436 | 4.62E-05 | 6.32E-05 |
| S100A6 | 1.840727 | 7.24E-05 | 9.76E-05 |
| S100P | 6.449663 | 1.13E-07 | 1.84E-07 |
| S100A10 | 1.914281 | 1.88E-18 | 7.45E-18 |
| DEFB132 | 3.187069 | 0.002488 | 0.003042 |
| LCN12 | 1.060613 | 1.64E-10 | 3.22E-10 |
| S100A11 | 1.707509 | 2.44E-05 | 3.41E-05 |
| S100A3 | 2.547818 | 1.34E-17 | 4.88E-17 |
| S100A1 | 3.375022 | 7.33E-12 | 1.57E-11 |
| COLEC12 | 3.1601 | 3.86E-09 | 6.87E-09 |
| MAVS | 1.548938 | 1.23E-24 | 1.67E-23 |
| IL6 | -1.43476 | 3.11E-05 | 4.31E-05 |
| TGFB1 | 1.303598 | 0.00252 | 0.003079 |
| MMP9 | 2.720729 | 2.14E-08 | 3.64E-08 |
| FABP6 | 5.416595 | 4.64E-06 | 6.77E-06 |
| MBL2 | -1.27189 | 7.42E-14 | 1.87E-13 |
| PLAU | 2.0275 | 4.04E-15 | 1.13E-14 |
| IL1B | -1.06356 | 2.07E-10 | 4.03E-10 |
| PAEP | 10.25909 | 2.23E-11 | 4.63E-11 |
| IRF3 | 1.360867 | 9.34E-24 | 9.80E-23 |
| LPA | -1.80332 | 2.38E-22 | 1.75E-21 |
| NOX4 | 3.487096 | 1.94E-28 | 3.44E-26 |
| FABP5 | 2.386572 | 2.91E-19 | 1.28E-18 |
| FABP4 | 3.262002 | 4.89E-09 | 8.66E-09 |
| RBP7 | 2.499361 | 1.63E-21 | 1.03E-20 |
| DUOX1 | 3.117501 | 5.88E-20 | 2.85E-19 |
| RBP2 | 4.984714 | 4.18E-05 | 5.73E-05 |
| CETP | -2.24486 | 2.59E-22 | 1.89E-21 |
| LCNL1 | 4.079973 | 0.038265 | 0.04291 |
| PI15 | 4.830642 | 1.88E-12 | 4.22E-12 |
| NOX1 | 1.505125 | 9.70E-15 | 2.62E-14 |
| APOD | 1.826481 | 3.76E-05 | 5.17E-05 |
| ORM2 | -1.0585 | 2.57E-15 | 7.30E-15 |
| ORM1 | -1.30454 | 3.46E-14 | 8.95E-14 |
| PML | 1.061959 | 9.29E-18 | 3.44E-17 |
| ISG20L2 | 1.250603 | 1.85E-24 | 2.37E-23 |
| TFRC | 1.578697 | 1.93E-16 | 6.18E-16 |
| IDO1 | 1.450023 | 0.000361 | 0.000465 |
| SOCS3 | -1.65666 | 4.00E-10 | 7.63E-10 |
| CCL20 | 1.944409 | 9.83E-08 | 1.60E-07 |
| PPIA | 1.300985 | 9.81E-28 | 7.34E-26 |
| ZYX | 1.078749 | 8.78E-13 | 2.02E-12 |
| PGC | 11.17373 | 2.87E-12 | 6.36E-12 |
| IKBKE | 1.86881 | 1.30E-11 | 2.74E-11 |
| FCN2 | -4.39667 | 5.64E-29 | 2.53E-26 |
| MAPT | 3.728369 | 3.34E-25 | 5.55E-24 |
| LYZ | 2.742507 | 2.09E-07 | 3.34E-07 |
| KLKB1 | -1.07175 | 5.73E-18 | 2.17E-17 |
| ITGAV | 1.428586 | 7.54E-09 | 1.32E-08 |
| IRF5 | 1.245307 | 4.35E-17 | 1.49E-16 |
| CACYBP | 1.748309 | 6.00E-27 | 2.39E-25 |
| NOD1 | 1.027988 | 4.82E-14 | 1.23E-13 |
| MAPK3 | 1.378505 | 5.73E-26 | 1.33E-24 |
| GRN | 1.209499 | 9.80E-21 | 5.39E-20 |
| GNAI1 | 1.014646 | 1.36E-08 | 2.34E-08 |
| WNT5A | 1.404518 | 0.022607 | 0.02574 |
| ADAR | 1.026746 | 4.15E-18 | 1.59E-17 |
| TYK2 | 1.028319 | 2.85E-23 | 2.63E-22 |
| NOS2 | 2.049054 | 5.21E-13 | 1.22E-12 |
| TRAF3 | 1.193726 | 8.77E-16 | 2.61E-15 |
| TPM2 | 2.579094 | 1.48E-23 | 1.48E-22 |
| SRC | 1.862442 | 2.16E-10 | 4.19E-10 |
| ROBO3 | 1.503188 | 2.58E-09 | 4.64E-09 |
| DLL4 | 1.756678 | 9.02E-22 | 5.97E-21 |
| DMBT1 | 7.19408 | 5.11E-06 | 7.43E-06 |
| SKIV2L | 1.150515 | 3.21E-25 | 5.38E-24 |
| DCK | 1.344975 | 1.32E-15 | 3.87E-15 |
| DAXX | 1.282463 | 1.47E-24 | 1.95E-23 |
| EED | 1.255517 | 1.10E-24 | 1.52E-23 |
| APOBEC3H | 1.173997 | 2.21E-07 | 3.51E-07 |
| SPINK5 | 3.864661 | 7.88E-17 | 2.63E-16 |
| MARCO | -4.2941 | 9.04E-28 | 7.01E-26 |
| CCL28 | 1.835501 | 0.000587 | 0.000746 |
| LTB4R | 1.197559 | 4.00E-10 | 7.63E-10 |
| TRIM27 | 1.178554 | 2.30E-25 | 4.08E-24 |
| SYTL1 | 1.043168 | 0.002551 | 0.003117 |
| PTGS2 | -1.70841 | 5.31E-17 | 1.80E-16 |
| MASP1 | -1.0353 | 8.75E-20 | 4.14E-19 |
| MAP2K2 | 1.318561 | 3.14E-26 | 8.36E-25 |
| NDRG1 | 1.903125 | 7.08E-12 | 1.52E-11 |
| LANCL1 | 1.093487 | 1.02E-17 | 3.77E-17 |
| ABCC4 | 2.306015 | 1.11E-09 | 2.05E-09 |
| HGF | -1.38493 | 3.52E-20 | 1.77E-19 |
| HDAC1 | 1.026001 | 6.77E-19 | 2.84E-18 |
| BACH2 | -1.01294 | 4.58E-09 | 8.12E-09 |
| STAB2 | -4.30849 | 2.35E-28 | 3.60E-26 |
| PDGFRB | 1.823863 | 7.33E-17 | 2.45E-16 |
| PDCD1 | 2.1504 | 0.01699 | 0.019493 |
| PCSK1 | 7.398097 | 4.22E-05 | 5.79E-05 |
| ARG2 | 2.019573 | 7.62E-05 | 0.000103 |
| BIRC5 | 4.814625 | 2.35E-28 | 3.60E-26 |
| VIM | 1.039764 | 1.08E-08 | 1.88E-08 |
| GBP2 | 1.957738 | 9.02E-19 | 3.73E-18 |
| ALB | -1.27141 | 6.53E-21 | 3.71E-20 |
| AGER | 1.25595 | 1.99E-15 | 5.72E-15 |
| TNFSF4 | 2.42407 | 1.01E-18 | 4.14E-18 |
| ACTA1 | 2.073989 | 7.24E-11 | 1.45E-10 |
| CCL15 | 1.1611 | 7.47E-13 | 1.73E-12 |
| CCL14 | -1.35713 | 1.41E-19 | 6.46E-19 |
| CCL26 | 3.790784 | 2.51E-09 | 4.53E-09 |
| CCL23 | -2.35648 | 4.04E-24 | 4.63E-23 |
| CCL25 | 7.289122 | 1.67E-10 | 3.28E-10 |
| PPARG | 1.208227 | 6.56E-11 | 1.32E-10 |
| MIF | 1.301775 | 2.78E-13 | 6.67E-13 |
| PTK2 | 1.354811 | 1.97E-23 | 1.90E-22 |
| VDR | 1.55964 | 1.87E-05 | 2.62E-05 |
| RAC3 | 1.613134 | 2.61E-12 | 5.81E-12 |
| NFATC4 | 2.011999 | 1.21E-12 | 2.76E-12 |
| HRAS | 1.585403 | 7.59E-25 | 1.11E-23 |
| NRAS | 1.044759 | 7.64E-17 | 2.55E-16 |
| FOS | -2.41925 | 6.68E-21 | 3.78E-20 |
| IKBKG | 1.508548 | 9.70E-15 | 2.62E-14 |
| NFKBIE | 1.133919 | 3.31E-10 | 6.34E-10 |
| PIK3R2 | 1.882465 | 2.52E-19 | 1.12E-18 |
| PIK3R3 | 1.280855 | 1.90E-18 | 7.53E-18 |
| FCGR2B | -1.10985 | 1.30E-18 | 5.25E-18 |
| CKLF | 1.626423 | 7.16E-23 | 5.92E-22 |
| CXCL17 | 4.933831 | 1.06E-06 | 1.61E-06 |
| CYR61 | -1.35363 | 1.35E-14 | 3.59E-14 |
| SAA1 | -1.59235 | 2.16E-06 | 3.22E-06 |
| SAA2 | -1.86361 | 1.92E-06 | 2.86E-06 |
| SEMA3B | 2.174583 | 6.74E-07 | 1.04E-06 |
| SEMA3F | 1.920567 | 8.94E-30 | 1.28E-26 |
| SEMA3G | 1.741113 | 3.52E-15 | 9.90E-15 |
| SEMA4F | 2.078276 | 2.04E-13 | 4.95E-13 |
| SEMA5B | 3.054072 | 4.13E-27 | 1.88E-25 |
| SEMA6B | 1.0524 | 8.41E-12 | 1.79E-11 |
| SEMA6C | 2.129527 | 7.08E-21 | 3.99E-20 |
| SEMA7A | 1.848927 | 9.40E-12 | 2.00E-11 |
| TYMP | 1.023739 | 1.52E-06 | 2.29E-06 |
| CXCR3 | 1.189497 | 2.78E-05 | 3.86E-05 |
| EDNRA | 1.110924 | 0.000584 | 0.000743 |
| FPR1 | -1.17497 | 2.27E-12 | 5.06E-12 |
| PLXNA1 | 2.023359 | 1.53E-17 | 5.53E-17 |
| PLXNA2 | 1.334378 | 1.42E-20 | 7.62E-20 |
| PLXNA3 | 2.123267 | 3.59E-15 | 1.01E-14 |
| PLXNC1 | 2.441815 | 1.58E-15 | 4.60E-15 |
| PLXND1 | 1.447657 | 5.61E-18 | 2.13E-17 |
| ROBO1 | 3.534811 | 7.16E-19 | 2.99E-18 |
| ADM2 | 2.831467 | 6.47E-17 | 2.17E-16 |
| AMH | 3.293923 | 6.50E-08 | 1.07E-07 |
| APLN | 4.622208 | 1.08E-28 | 2.88E-26 |
| BMP4 | 2.426482 | 3.34E-08 | 5.59E-08 |
| BMP5 | -1.72627 | 2.06E-21 | 1.28E-20 |
| BMP7 | 4.736981 | 0.00017 | 0.000224 |
| BMP8B | 2.12609 | 0.004922 | 0.005879 |
| CD320 | 1.398697 | 1.19E-20 | 6.48E-20 |
| CD70 | 2.420479 | 1.61E-05 | 2.27E-05 |
| CHGA | 8.060408 | 7.34E-13 | 1.70E-12 |
| CMTM3 | 1.276785 | 1.35E-07 | 2.18E-07 |
| CMTM4 | 2.059924 | 2.85E-19 | 1.26E-18 |
| CSPG5 | 3.246434 | 1.30E-21 | 8.39E-21 |
| DKK1 | 6.431558 | 3.31E-08 | 5.55E-08 |
| EGF | 5.593952 | 1.10E-05 | 1.57E-05 |
| ESM1 | 4.918761 | 2.23E-27 | 1.27E-25 |
| FGF12 | 2.509103 | 2.65E-10 | 5.12E-10 |
| FGF13 | 2.505838 | 1.98E-09 | 3.60E-09 |
| FIGNL2 | 2.311355 | 1.07E-10 | 2.12E-10 |
| GAL | 3.707689 | 0.015201 | 0.017491 |
| GDF11 | 1.18641 | 0.025046 | 0.028421 |
| GDF2 | -4.23731 | 1.12E-32 | 1.44E-28 |
| GMFB | 1.023249 | 1.61E-16 | 5.19E-16 |
| GNRH1 | 1.706132 | 1.05E-16 | 3.47E-16 |
| GPI | 1.021478 | 1.93E-16 | 6.18E-16 |
| IL11 | 4.706871 | 7.00E-09 | 1.23E-08 |
| IL17D | 3.841742 | 9.47E-13 | 2.18E-12 |
| IL1RN | -1.51967 | 2.19E-11 | 4.56E-11 |
| IL32 | 1.304268 | 2.19E-07 | 3.49E-07 |
| IL34 | 1.415326 | 5.27E-05 | 7.18E-05 |
| INHA | 5.42 | 4.72E-07 | 7.36E-07 |
| INS-IGF2 | -3.65141 | 5.02E-30 | 1.08E-26 |
| JAG1 | 1.45126 | 4.51E-07 | 7.05E-07 |
| JAG2 | 2.364662 | 3.69E-23 | 3.28E-22 |
| KITLG | 1.899602 | 3.08E-12 | 6.82E-12 |
| LTB | 1.635728 | 0.000297 | 0.000384 |
| LTBP2 | 1.908051 | 6.44E-08 | 1.06E-07 |
| MDK | 4.34479 | 1.00E-24 | 1.41E-23 |
| NENF | 1.322306 | 5.37E-20 | 2.62E-19 |
| NMB | 2.85231 | 1.27E-25 | 2.51E-24 |
| NOV | 1.891165 | 2.18E-09 | 3.95E-09 |
| NRG1 | -1.28012 | 5.44E-12 | 1.18E-11 |
| NRG2 | 2.879786 | 1.43E-09 | 2.62E-09 |
| NTF3 | -2.86819 | 1.89E-27 | 1.14E-25 |
| OSGIN1 | 1.50324 | 1.32E-07 | 2.13E-07 |
| OXT | -1.01078 | 4.71E-14 | 1.20E-13 |
| PDGFA | 2.596397 | 3.44E-19 | 1.50E-18 |
| PDGFB | 1.568964 | 1.84E-18 | 7.31E-18 |
| PDGFD | 1.680541 | 0.002449 | 0.002995 |
| PDGFRL | 2.365731 | 8.68E-16 | 2.59E-15 |
| PGF | 1.843288 | 3.66E-13 | 8.68E-13 |
| PTHLH | 2.802604 | 2.49E-05 | 3.48E-05 |
| RABEP2 | 1.074099 | 6.14E-17 | 2.07E-16 |
| REG1A | 10.79379 | 2.49E-08 | 4.22E-08 |
| SCGB3A1 | -1.55823 | 2.34E-09 | 4.22E-09 |
| SECTM1 | 1.31209 | 9.90E-06 | 1.42E-05 |
| SPP1 | 5.006114 | 2.72E-07 | 4.31E-07 |
| STC1 | 1.712481 | 5.21E-13 | 1.22E-12 |
| STC2 | 2.8251 | 1.14E-18 | 4.63E-18 |
| TDGF1 | 3.023943 | 3.96E-05 | 5.44E-05 |
| TGFB2 | 2.008395 | 9.02E-05 | 0.000121 |
| TNFSF15 | 2.620403 | 6.61E-09 | 1.16E-08 |
| TNFSF9 | 2.0472 | 0.000332 | 0.000428 |
| TSLP | -1.77599 | 1.59E-20 | 8.46E-20 |
| UCN | 1.905396 | 8.59E-16 | 2.56E-15 |
| UTS2 | 2.58767 | 0.001018 | 0.001275 |
| VEGFB | 1.318205 | 5.30E-05 | 7.22E-05 |
| VGF | 2.784247 | 5.53E-08 | 9.14E-08 |
| ACVRL1 | 1.321845 | 2.58E-18 | 1.01E-17 |
| ANGPT1 | 1.547875 | 3.95E-08 | 6.58E-08 |
| ANGPTL6 | -2.41451 | 3.51E-29 | 2.26E-26 |
| AVPR1A | -1.81508 | 2.83E-15 | 8.02E-15 |
| BRD8 | 1.331907 | 1.85E-24 | 2.37E-23 |
| CALCRL | 1.108424 | 1.12E-05 | 1.60E-05 |
| CRLF1 | 3.188206 | 1.02E-15 | 3.02E-15 |
| CRLF2 | 4.220902 | 1.05E-06 | 1.61E-06 |
| CSF2RA | 1.002201 | 0.010499 | 0.012217 |
| ESR1 | -1.94173 | 1.04E-22 | 8.36E-22 |
| FGFR3 | 1.448762 | 3.35E-06 | 4.94E-06 |
| FGFR4 | 1.466063 | 3.10E-18 | 1.20E-17 |
| FLT1 | 1.027421 | 2.78E-10 | 5.36E-10 |
| GALR3 | 1.958779 | 1.44E-05 | 2.04E-05 |
| GHR | -1.84875 | 1.52E-21 | 9.68E-21 |
| GHRHR | 4.203929 | 6.61E-06 | 9.54E-06 |
| GIPR | 2.752406 | 0.00343 | 0.004151 |
| HTR3A | 6.049049 | 0.000548 | 0.000698 |
| IL11RA | 1.023728 | 3.34E-13 | 7.95E-13 |
| IL17RB | 1.175775 | 1.07E-07 | 1.73E-07 |
| IL1R2 | 1.39536 | 3.45E-09 | 6.17E-09 |
| IL1RAP | -1.29176 | 3.91E-16 | 1.21E-15 |
| IL1RL1 | -2.77083 | 2.86E-14 | 7.45E-14 |
| IL22RA1 | 1.132872 | 0.001141 | 0.001424 |
| IL27RA | 1.220625 | 3.50E-09 | 6.26E-09 |
| IL2RG | 1.224042 | 0.017047 | 0.019557 |
| IL3RA | 1.825694 | 3.14E-24 | 3.73E-23 |
| LIFR | -2.18671 | 4.79E-25 | 7.47E-24 |
| MC1R | 1.851085 | 6.51E-15 | 1.78E-14 |
| MCHR1 | 1.808943 | 0.001501 | 0.001859 |
| NGFR | -1.33621 | 8.26E-18 | 3.07E-17 |
| NR2C2 | 1.219943 | 2.68E-12 | 5.96E-12 |
| NR4A1 | -1.51633 | 3.37E-14 | 8.71E-14 |
| NR4A2 | -1.24568 | 1.64E-10 | 3.22E-10 |
| NR4A3 | -1.65012 | 2.23E-13 | 5.40E-13 |
| NR6A1 | 1.97935 | 1.80E-22 | 1.37E-21 |
| OPRL1 | 1.274106 | 9.13E-07 | 1.40E-06 |
| OXTR | 2.22937 | 1.42E-07 | 2.29E-07 |
| PPARD | 1.042313 | 1.51E-11 | 3.18E-11 |
| PTH1R | -2.59953 | 1.59E-27 | 1.01E-25 |
| RARG | 1.064021 | 1.02E-07 | 1.67E-07 |
| RXRB | 1.178769 | 1.28E-25 | 2.52E-24 |
| SORT1 | 1.846919 | 3.08E-17 | 1.08E-16 |
| THRA | 1.238256 | 3.46E-16 | 1.08E-15 |
| TNFRSF10C | 1.342086 | 0.000122 | 0.000161 |
| TNFRSF11A | 1.526322 | 0.000297 | 0.000384 |
| TNFRSF18 | 2.561731 | 9.11E-14 | 2.27E-13 |
| TNFRSF21 | 1.332744 | 0.000119 | 0.000158 |
| TNFRSF25 | 2.149708 | 3.26E-22 | 2.34E-21 |
| TNFRSF4 | 2.877453 | 6.79E-26 | 1.51E-24 |
| TNFRSF9 | 2.560296 | 0.000211 | 0.000275 |
| VIPR1 | -2.93259 | 3.31E-28 | 4.02E-26 |
| PAK1 | 1.264339 | 2.19E-15 | 6.28E-15 |
| PLCG1 | 1.664063 | 1.77E-21 | 1.11E-20 |
| SHC1 | 1.541731 | 5.37E-26 | 1.26E-24 |
| CD244 | -1.06305 | 2.32E-13 | 5.59E-13 |
| PRKCA | 1.475494 | 2.33E-19 | 1.04E-18 |
| CASP3 | 1.011805 | 1.98E-17 | 7.05E-17 |
| CD3D | 1.165999 | 0.00374 | 0.004511 |
| NCK2 | 1.296592 | 0.003093 | 0.003757 |
| PAK4 | 1.139853 | 1.88E-18 | 7.45E-18 |
| CTLA4 | 1.714908 | 6.29E-06 | 9.09E-06 |
| CDK4 | 1.382142 | 8.35E-19 | 3.47E-18 |
| PDK1 | 1.10265 | 3.65E-08 | 6.11E-08 |

DE differentially expressed, HCC hepatocellular carcinoma, FC Fold Change, FDR false discovery rate
