# Supplementary material for: Identification of a five-immune gene model as an independent prognostic factor in hepatocellular carcinoma
Source: BMC Cancer. 2021 Mar 16;21:278. doi: 10.1186/s12885-021-08012-2 (PMC7962305; doi:10.1186/s12885-021-08012-2)
Supplement: Supplementary file 4 — Additional file 4: Table S4. Significantly enriched GO terms [file 12885_2021_8012_MOESM4_ESM.docx]

**Table S4** Significantly enriched GO terms

| ONTOLOGY | ID | Description | GeneRatio | BgRatio | pvalue | p.adjust | qvalue | geneID |
| --- | --- | --- | --- | --- | --- | --- | --- | --- |
| BP | GO:0006955 | immune response | 0.16 | 445/18493 | 1.16E-26 | 2.05E-23 | 2.89E-23 | IL27RA/ TNFSF15/ VIPR1/ HLA-DMA/ CXCL12/ HAMP/ LTB4R/ SEMA7A/ CCR10/ IL1RAP/ IL1B/ LTB/ APLN/ LTA/ CIITA/ HLA-F/ TNFRSF9/ TNFRSF10C/ PPBP/ NGFR/ GBP2/ IL1R2/ TNFRSF21/ TNFRSF25/ CCL8/ IL32/ CD70/ TNFRSF4/ CCL28/ CCL26/ CCL25/ CCL23/ CCL20/ TNFRSF18/ IL2RG/ CD4/ THBS1/ HLA-DOB/ SECTM1/ IL6/ TNFSF4/ IL1RL1/ IL1RN/ CTLA4/ TNFSF9/ CCL15/ IKBKE/ CCL13/ CCL14/ FCGR2B/ CXCL14/ IKBKG |
| BP | GO:0006954 | inflammatory response | 0.141538 | 414/18493 | 3.26E-23 | 5.75E-20 | 4.06E-20 | TNFRSF21/ PTGS2/ TNFRSF25/ FPR1/ CCL8/ CXCR3/ IL34/ CXCL12/ TNFRSF4/ TGFB1/ CCL26/ MIF/ IL17D/ FOS/ CCL25/ TNFRSF11A/ NOD1/ CCL23/ CCL20/ LTB4R/ SEMA7A/ IL1RAP/ TNFRSF18/ IL1B/ NFATC4/ THBS1/ SPP1/ CIITA/ NOX4/ IL6/ UCN/ RELB/ NOX1/ LYZ/ GAL/ AGER/ CCL15/ S100A12/ TNFRSF9/ ORM1/ TNFRSF10C/ CCL13/ CCL14/ PPBP/ IKBKG/ NGFR |
| BP | GO:0008284 | positive regulation of cell proliferation | 0.141538 | 259/18493 | 1.39E-19 | 2.45E-16 | 1.15E-16 | FGFR4/ HRAS/ FGFR3/ PDGFB/ PDGFA/ PGF/ PTH1R/ CRLF1/ NMB/ CXCR3/ ESM1/ VIPR1/ CALR/ IL34/ GHRHR/ TGFB1/ IL11/ TGFB2/ PTK2/ TNFRSF11A/ REG1A/ ITGAV/ TDGF1/ SHC1/ PAK1/ PDGFD/ EGF/ THBS1/ NRG1/ APLN/ IL6/ RARG/ TNFSF4/ FLT1/ NTF3/ NOX1/ LIFR/ BIRC5/ HGF/ CDK4/ S100A13/ PTHLH/ CCL14/ HDAC1/ AVPR1A/ PDGFRB |
| BP | GO:0007165 | signal transduction | 0.221538 | 90/18493 | 3.94E-19 | 6.94E-16 | 2.45E-16 | HRAS/ S100A6/ ACVRL1/ PGF/ PPARG/ TNFSF15/ FGF13/ FGF12/ CXCL12/ PDCD1/ EDNRA/ TNFRSF11A/ NOD1/ PAK4/ IL1B/ SHC1/ ZYX/ APLN/ NRG2/ LTB/ LTA/ BRD8/ HSP90AA1/ ESR1/ INHA/ CDK4/ NCK2/ TNFRSF10C/ GRN/ DLL4/ PDGFRB/ MAVS/ TNFRSF21/ CD244/ TNFRSF25/ OXT/ FPR1/ CCL8/ KITLG/ CD70/ NMB/ MDK/ SRC/ CCL26/ VDR/ CCL23/ CCL20/ TNFRSF18/ IL2RG/ CD4/ EGF/ HTR3A/ HLA-DOB/ TRAF3/ PIK3R2/ SECTM1/ TNFSF4/ GNRH1/ NTF3/ IL1RL1/ NOX1/ S100A11/ NR4A2/ NR4A1/ TNFSF9/ CCL15/ GMFB/ CCL13/ CXCL14/ PLCG1/ FCGR2B/ PLAU |
| BP | GO:0070374 | positive regulation of ERK1 and ERK2 cascade | 0.086154 | 303/18493 | 3.27E-17 | 5.76E-14 | 1.63E-14 | FGFR4/ HRAS/ FGFR3/ PDGFB/ PDGFA/ CCL8/ TGFB1/ SRC/ CCL26/ MIF/ CCL25/ NOD1/ CCL23/ CCL20/ SEMA7A/ ANGPT1/ PDGFD/ PRKCA/ NOX4/ BMP4/ IL6/ MAP2K2/ CCL15/ VEGFB/ CCL13/ CCL14/ MAPK3/ PDGFRB |
| BP | GO:0030335 | positive regulation of cell migration | 0.086154 | 122/18493 | 1.20E-16 | 2.00E-13 | 4.61E-14 | HRAS/ PDGFB/ PDGFA/ TGFB1/ CCL26/ SEMA5B/ PTK2/ SEMA3G/ ITGAV/ SEMA3F/ SEMA7A/ TDGF1/ SEMA3B/ HSPA5/ PDGFD/ PAK1/ THBS1/ CYR61/ PRKCA/ BMP4/ FLT1/ NTF3/ HGF/ SEMA6B/ SEMA6C/ SEMA4F/ PDGFRB/ PLAU |
| BP | GO:0007267 | cell-cell signaling | 0.098462 | 264/18493 | 1.30E-16 | 2.00E-13 | 3.95E-14 | FGFR3/ PDGFA/ PGF/ CCL8/ NMB/ FGF13/ CD70/ FGF12/ CCL26/ IL11/ TGFB2/ NOV/ PCSK1/ TNFRSF11A/ CCL23/ CCL20/ IL1B/ SEMA3B/ ZYX/ LTB/ LTA/ CYR61/ NTF3/ GNRH1/ INHA/ TNFSF9/ CCL15/ PTHLH/ AMH/ CCL13/ CXCL14/ SEMA4F |
| BP | GO:0001525 | angiogenesis | 0.089231 | 266/18493 | 2.18E-15 | 3.92E-12 | 6.92E-13 | GDF2/ ACVRL1/ PTGS2/ PDGFA/ PGF/ JAG1/ CXCR3/ ESM1/ TGFB2/ NOV/ ANGPTL6/ PTK2/ TYMP/ APOD/ ITGAV/ ANGPT1/ SHC1/ CALCRL/ PLXND1/ EGF/ PRKCA/ FLT1/ NOX1/ STAB2/ VEGFB/ GPI/ CXCL17/ DLL4/ ADM2 |
| BP | GO:0000165 | MAPK cascade | 0.089231 | 283/18493 | 1.37E-13 | 2.42E-10 | 3.80E-11 | FGFR4/ HRAS/ FGFR3/ PDGFB/ PDGFA/ KITLG/ FGF13/ TGFB1/ PTK2/ PSMD2/ IL1B/ IL2RG/ PSMD4/ ANGPT1/ SHC1/ PAK1/ NRG1/ EGF/ NRG2/ CSF2RA/ MAP2K2/ NRAS/ PSMD14/ PSMC4/ PSMD10/ MAPK3/ PDGFRB/ PSME3/ IL3RA |
| BP | GO:0033209 | tumor necrosis factor-mediated signaling pathway | 0.061538 | 205/18493 | 8.72E-13 | 1.54E-09 | 2.17E-10 | TNFRSF21/ TNFSF4/ TNFRSF25/ TNFSF15/ CD70/ TNFRSF4/ TNFRSF9/ TNFRSF10C/ PSMD14/ TNFRSF11A/ PSMC4/ PSMD10/ TNFRSF18/ PSMD2/ PSME3/ PSMD4/ NGFR/ LTB/ LTA/ TRAF3 |
| BP | GO:0006935 | chemotaxis | 0.061538 | 206/18493 | 1.62E-12 | 2.85E-09 | 3.66E-10 | HRAS/ FPR1/ CCL8/ CXCR3/ CCL28/ CCL15/ CXCL12/ CCL26/ CCL25/ CXCL17/ TYMP/ CCL13/ CCL23/ CXCL14/ CCL20/ CCR10/ CMTM4/ CMTM3/ PLAU/ CYR61 |
| BP | GO:0002548 | monocyte chemotaxis | 0.04 | 216/18493 | 1.19E-11 | 2.11E-08 | 2.48E-09 | IL6/ FLT1/ PDGFB/ CCL8/ CCL15/ S100A12/ CCL26/ CCL25/ CCL13/ CCL23/ CCL14/ TNFRSF11A/ CCL20 |
| BP | GO:0060326 | cell chemotaxis | 0.046154 | 472/18493 | 1.53E-11 | 2.70E-08 | 2.93E-09 | PDGFB/ FPR1/ CCL8/ HGF/ CXCL12/ CCL28/ CCL15/ NOV/ CCL25/ CCL13/ CCL14/ CCL20/ CXCL14/ SAA2/ PDGFRB |
| BP | GO:0043401 | steroid hormone mediated signaling pathway | 0.043077 | 344/18493 | 3.80E-11 | 6.71E-08 | 6.77E-09 | BMP4/ PPARD/ THRA/ RARG/ RXRB/ PPARG/ NR6A1/ ESR1/ NR4A2/ NR4A1/ NR4A3/ NR2C2/ VDR/ BMP7 |
| BP | GO:0000187 | activation of MAPK activity | 0.052308 | 89/18493 | 1.80E-10 | 3.18E-07 | 2.99E-08 | WNT5A/ NTF3/ MAP2K2/ FPR1/ HGF/ CXCL17/ NOD1/ SAA1/ TDGF1/ MAPK3/ IKBKG/ IL1B/ SHC1/ THBS1/ EGF/ NRG1/ GHR |
| BP | GO:0030593 | neutrophil chemotaxis | 0.043077 | 89/18493 | 2.72E-10 | 4.80E-07 | 4.24E-08 | CCL8/ CCL15/ TGFB2/ S100A12/ CCL26/ CCL25/ CCL13/ CCL23/ CCL14/ CCL20/ PPBP/ SAA1/ CKLF/ IL1B |
| BP | GO:0071526 | semaphorin-plexin signaling pathway | 0.033846 | 451/18493 | 3.34E-10 | 5.90E-07 | 4.90E-08 | SEMA5B/ PLXNA3/ SEMA6B/ SEMA6C/ SEMA3G/ SEMA4F/ PLXNA2/ SEMA3F/ SEMA7A/ SEMA3B/ PLXND1 |
| BP | GO:0050731 | positive regulation of peptidyl-tyrosine phosphorylation | 0.046154 | 126/18493 | 4.13E-10 | 7.28E-07 | 5.71E-08 | IL6/ NTF3/ PDGFB/ KITLG/ HGF/ SRC/ TGFB1/ IL11/ MIF/ VEGFB/ TDGF1/ CD4/ ANGPT1/ NRG1/ GHR |
| BP | GO:0050919 | negative chemotaxis | 0.033846 | 94/18493 | 4.66E-10 | 8.21E-07 | 6.11E-08 | SEMA5B/ PLXNA3/ SEMA6B/ SEMA6C/ SEMA3G/ PDGFA/ SEMA4F/ ITGAV/ SEMA3F/ SEMA7A/ SEMA3B |
| BP | GO:0048843 | negative regulation of axon extension involved in axon guidance | 0.030769 | 134/18493 | 6.77E-10 | 1.19E-06 | 8.44E-08 | WNT5A/ SEMA5B/ PLXNA3/ SEMA6B/ SEMA6C/ SEMA3G/ SEMA4F/ SEMA3F/ SEMA7A/ SEMA3B |
| BP | GO:0043406 | positive regulation of MAP kinase activity | 0.04 | 354/18493 | 9.05E-10 | 1.60E-06 | 1.07E-07 | NOX4/ HRAS/ FLT1/ PDGFB/ PDGFA/ KITLG/ PDGFRB/ PDGFD/ EGF/ TGFB1/ SRC/ S100A12/ MIF |
| BP | GO:0048247 | lymphocyte chemotaxis | 0.030769 | 139/18493 | 1.45E-09 | 2.55E-06 | 1.64E-07 | CCL25/ CCL13/ CCL23/ CCL14/ CCL20/ SAA1/ CKLF/ CCL8/ CCL15/ CCL26 |
| BP | GO:0071356 | cellular response to tumor necrosis factor | 0.049231 | 183/18493 | 2.52E-09 | 4.45E-06 | 2.73E-07 | TNFRSF21/ IL6/ CCL8/ CCL15/ CCL26/ LCN2/ CCL25/ CCL13/ CCL23/ CCL14/ CCL20/ HDAC1/ HAMP/ TDGF1/ FABP4/ THBS1 |
| BP | GO:0048146 | positive regulation of fibroblast proliferation | 0.036923 | 342/18493 | 4.53E-09 | 7.98E-06 | 4.70E-07 | WNT5A/ S100A6/ UTS2/ PDGFB/ PDGFA/ PML/ ESR1/ PDGFRB/ PDGFD/ CDK4/ TGFB1/ MIF |
| BP | GO:0042981 | regulation of apoptotic process | 0.064615 | 59/18493 | 4.56E-09 | 8.05E-06 | 4.55E-07 | TNFRSF21/ GDF2/ NTF3/ TNFRSF25/ ESR1/ BIRC5/ INHA/ CALR/ TNFRSF4/ TGFB1/ TNFRSF9/ TNFRSF10C/ TNFRSF11A/ NOD1/ GDF11/ NDRG1/ NGFR/ BMP7/ BMP8B/ BMP5/ TRAF3 |
| BP | GO:0071346 | cellular response to interferon-gamma | 0.036923 | 91/18493 | 8.30E-09 | 1.46E-05 | 7.95E-07 | CIITA/ WNT5A/ CCL25/ CCL13/ CCL23/ CCL14/ CCL20/ TDGF1/ CCL8/ NOS2/ CCL15/ CCL26 |
| BP | GO:0050918 | positive chemotaxis | 0.030769 | 198/18493 | 1.32E-08 | 2.32E-05 | 1.22E-06 | BMP4/ NTF3/ SAA2/ PDGFB/ SAA1/ ANGPT1/ HGF/ CXCL12/ CCL15/ MIF |
| BP | GO:0045087 | innate immune response | 0.089231 | 175/18493 | 1.49E-08 | 2.64E-05 | 1.33E-06 | MAVS/ MBL2/ CD244/ PPARG/ PML/ APOBEC3H/ IL34/ SRC/ MIF/ MARCO/ PTK2/ NOD1/ SAA1/ IL1RAP/ TRAF3/ DEFB132/ RELB/ TRIM27/ COLEC12/ AGER/ CLEC4M/ CD1D/ S100A12/ TYK2/ LCN2/ CHGA/ IKBKG/ DMBT1/ ADAR |
| BP | GO:0042127 | regulation of cell proliferation | 0.058462 | 178/18493 | 1.57E-08 | 2.77E-05 | 1.35E-06 | TNFRSF21/ TNFRSF25/ S100A11/ JAG2/ JAG1/ INHA/ TNFRSF4/ SRC/ TYK2/ TNFRSF10C/ PTK2/ TFRC/ PPBP/ TNFRSF18/ NDRG1/ SCGB3A1/ NOS2/ NGFR/ PLAU |
| BP | GO:0032355 | response to estradiol | 0.043077 | 344/18493 | 1.67E-08 | 2.94E-05 | 1.39E-06 | UCN/ PTGS2/ OPRL1/ OXT/ ESR1/ OXTR/ CALR/ TGFB1/ GPI/ CASP3/ PDGFRB/ CD4/ BMP7/ GHR |
| BP | GO:0042493 | response to drug | 0.073846 | 384/18493 | 1.99E-08 | 3.50E-05 | 1.60E-06 | IL6/ UTS2/ HSP90AA1/ PTGS2/ PGF/ PPARG/ OXTR/ CALR/ GAL/ CDK4/ MDK/ TGFB1/ SRC/ TGFB2/ LCN2/ VEGFB/ AMH/ FOS/ CASP3/ HDAC1/ APOD/ ABCC4/ THBS1/ LTA |
| BP | GO:0001666 | response to hypoxia | 0.055385 | 125/18493 | 3.14E-08 | 5.53E-05 | 2.44E-06 | NOX4/ UTS2/ ACVRL1/ PGF/ PML/ NR4A2/ AGER/ CXCL12/ TGFB1/ TGFB2/ VEGFB/ EDNRA/ CASP3/ PAK1/ NOS2/ THBS1/ LTA/ PLAU |
| BP | GO:0045944 | positive regulation of transcription from RNA polymerase II promoter | 0.141538 | 333/18493 | 3.48E-08 | 6.13E-05 | 2.62E-06 | MAVS/ WNT5A/ GDF2/ HRAS/ THRA/ ACVRL1/ NR6A1/ PPARG/ PML/ JAG1/ CXCR3/ RFXANK/ NR2C2/ TGFB1/ IL11/ FOS/ VDR/ MC1R/ GALR3/ IL1B/ NFATC4/ NRG1/ BRD8/ CYR61/ PIK3R2/ BMP4/ CIITA/ IL6/ UCN/ RARG/ CD3D/ RXRB/ RELB/ NR4A2/ ESR1/ NR4A1/ NR4A3/ HGF/ GAL/ NCK2/ IRF5/ HDAC1/ MAPK3/ IKBKG/ BMP7/ BMP5 |
| BP | GO:0007204 | positive regulation of cytosolic calcium ion concentration | 0.049231 | 132/18493 | 3.89E-08 | 6.86E-05 | 2.85E-06 | MCHR1/ UTS2/ OPRL1/ OXT/ PTH1R/ ESR1/ FPR1/ OXTR/ NMB/ CXCR3/ CCL28/ EDNRA/ SAA1/ GIPR/ CCR10/ AVPR1A |
| BP | GO:0008285 | negative regulation of cell proliferation | 0.083077 | 133/18493 | 4.24E-08 | 7.48E-05 | 3.02E-06 | HRAS/ ACVRL1/ PTGS2/ PTH1R/ PML/ TGFB1/ TGFB2/ AZGP1/ VDR/ CCL23/ IL1B/ NDRG1/ NOX4/ BMP4/ IL6/ RARG/ GNRH1/ S100A11/ PTHLH/ TNFRSF9/ NCK2/ HDAC1/ DLL4/ GDF11/ BMP7/ BMP5/ FABP6 |
| BP | GO:0070098 | chemokine-mediated signaling pathway | 0.036923 | 372/18493 | 9.09E-08 | 1.60E-04 | 6.29E-06 | CCL25/ CCL13/ CCL23/ CCL14/ PPBP/ CCL20/ CCR10/ CCL8/ CXCR3/ CXCL12/ CCL15/ CCL26 |
| BP | GO:0050729 | positive regulation of inflammatory response | 0.036923 | 139/18493 | 1.22E-07 | 2.15E-04 | 8.22E-06 | WNT5A/ CCL13/ CCL23/ CCL14/ TNFSF4/ IL1RL1/ CCL8/ FABP4/ CCL15/ CCL26/ IL17RB/ S100A12 |
| BP | GO:0048661 | positive regulation of smooth muscle cell proliferation | 0.033846 | 327/18493 | 1.73E-07 | 3.05E-04 | 1.13E-05 | BMP4/ IL6/ PDGFB/ PTGS2/ NOX1/ PDGFRB/ ABCC4/ PDGFD/ CALCRL/ NR4A3/ THBS1 |
| BP | GO:0043066 | negative regulation of apoptotic process | 0.086154 | 105/18493 | 1.81E-07 | 3.20E-04 | 1.16E-05 | WNT5A/ PPARD/ MMP9/ PDCD1/ SRC/ MIF/ PTK2/ CASP3/ ALB/ TDGF1/ TNFRSF18/ ANGPT1/ HSPA5/ THBS1/ CYR61/ BMP4/ IL6/ UCN/ RARG/ GNRH1/ SOCS3/ BIRC5/ VEGFB/ HDAC1/ PSMD10/ PDGFRB/ NGFR/ ADAR |
| BP | GO:0051781 | positive regulation of cell division | 0.030769 | 150/18493 | 2.08E-07 | 3.67E-04 | 1.30E-05 | VEGFB/ PPBP/ PDGFB/ PDGFA/ PGF/ IL1B/ PDGFD/ MDK/ TGFB1/ TGFB2 |
| BP | GO:0060395 | SMAD protein signal transduction | 0.033846 | 21/18493 | 2.38E-07 | 4.20E-04 | 1.45E-05 | BMP4/ FOS/ GDF2/ VIM/ GDF11/ INHA/ BMP7/ TGFB1/ BMP8B/ BMP5/ TGFB2 |
| BP | GO:0010862 | positive regulation of pathway-restricted SMAD protein phosphorylation | 0.030769 | 14/18493 | 2.52E-07 | 4.44E-04 | 1.49E-05 | BMP4/ GDF2/ ACVRL1/ GDF11/ INHA/ BMP7/ TGFB1/ BMP8B/ BMP5/ TGFB2 |
| BP | GO:0021785 | branchiomotor neuron axon guidance | 0.018462 | 33/18493 | 2.84E-07 | 5.01E-04 | 1.65E-05 | PLXNC1/ PLXNA3/ PLXNA1/ PLXNA2/ SEMA3F/ PLXND1 |
| BP | GO:0007166 | cell surface receptor signaling pathway | 0.064615 | 305/18493 | 3.04E-07 | 5.37E-04 | 1.72E-05 | MCHR1/ HRAS/ IL27RA/ CD3D/ TNFRSF25/ PTH1R/ LIFR/ OXTR/ INHA/ CXCR3/ VIPR1/ AGER/ GHRHR/ MIF/ MARCO/ CCL25/ IL17D/ GIPR/ CD4/ CALCRL/ BRD8 |
| BP | GO:0007411 | axon guidance | 0.049231 | 178/18493 | 3.77E-07 | 6.65E-04 | 2.09E-05 | WNT5A/ HRAS/ NR4A3/ CXCL12/ TGFB2/ NRAS/ PTK2/ SEMA6C/ ROBO1/ SEMA4F/ SEMA3F/ MAPK3/ SEMA3B/ ROBO3/ NGFR/ BMP7 |
| BP | GO:0014068 | positive regulation of phosphatidylinositol 3-kinase signaling | 0.033846 | 498/18493 | 3.77E-07 | 6.66E-04 | 2.04E-05 | PTK2/ PPARD/ FLT1/ PDGFB/ PDGFA/ PDGFRB/ ANGPT1/ PDGFD/ HGF/ NRG1/ TGFB2 |
| BP | GO:0032496 | response to lipopolysaccharide | 0.049231 | 157/18493 | 5.63E-07 | 9.92E-04 | 2.98E-05 | TNFRSF21/ PTGS2/ GNRH1/ TNFRSF25/ IDO1/ TNFRSF4/ TNFRSF9/ FOS/ CASP3/ TNFRSF10C/ TNFRSF11A/ PPBP/ HDAC1/ TNFRSF18/ NGFR/ LTA |
| CC | GO:0005615 | extracellular space | 0.338462 | 209/18493 | 2.94E-44 | 3.92E-41 | 8.63E-42 | UTS2/ GDF2/ MICA/ PDGFB/ LTBP2/ MASP1/ PDGFA/ PGF/ MMP9/ PGC/ TNFSF15/ FGF12/ CXCL12/ TGFB1/ IL11/ TGFB2/ AZGP1/ APOD/ SEMA7A/ TDGF1/ IL1B/ PDGFD/ STC2/ PTHLH/ VEGFB/ PPBP/ STC1/ WNT5A/ CCL8/ CD70/ IL32/ CALR/ IL34/ IL17D/ ALB/ KLKB1/ CKLF/ EGF/ SPP1/ BMP4/ UCN/ GNRH1/ IL1RN/ S100A11/ HGF/ S100A13/ LCN2/ CXCL17/ CXCL14/ PPIA/ BMP7/ BMP5/ PLAU/ BMP8B/ VGF/ SAA2/ SAA1/ SEMA3G/ HAMP/ SEMA3F/ ANGPT1/ CETP/ SEMA3B/ NRG1/ APLN/ LTB/ NRG2/ LTA/ GHR/ ACTA1/ LYZ/ GAL/ TNFRSF9/ AMH/ CHGA/ SEMA4F/ GRN/ ULBP2/ SCGB3A1/ MBL2/ OXT/ CRLF1/ KITLG/ CCL28/ MIF/ CCL26/ CCL25/ PCSK1/ CCL23/ CCL20/ NENF/ THBS1/ SECTM1/ IL6/ TNFSF4/ FLT1/ TNFSF9/ CCL15/ ORM1/ GPI/ TSLP/ CCL13/ CCL14/ DKK1/ TFRC/ GDF11/ CMTM4/ CMTM3/ ORM2/ DMBT1 |
| CC | GO:0005576 | extracellular region | 0.323077 | 134/18493 | 2.74E-33 | 3.65E-30 | 8.04E-31 | UTS2/ GDF2/ PDGFB/ MASP1/ PDGFA/ PGF/ MMP9/ FGF13/ CSPG5/ JAG1/ CXCL12/ TGFB1/ IL11/ TGFB2/ IL17RB/ NOV/ AZGP1/ LPA/ APOD/ IL1B/ PDGFD/ STC2/ COLEC10/ CLEC4M/ PTHLH/ VEGFB/ PPBP/ PDGFRL/ PAEP/ WNT5A/ IL1R2/ FGFR4/ FGFR3/ IFI30/ ESM1/ CALR/ IL34/ MDK/ IL17D/ ALB/ KLKB1/ CKLF/ EGF/ SPP1/ BMP4/ DEFB132/ GNRH1/ NTF3/ MAP2K2/ HGF/ AGER/ S100A12/ LCN2/ CXCL17/ CXCL14/ PPIA/ ADM2/ BMP7/ BMP5/ PLAU/ INS-IGF2/ SPINK5/ HAMP/ SAA1/ IL1RAP/ ANGPT1/ CETP/ NRG1/ APLN/ NRG2/ CSF2RA/ GHR/ CYR61/ HSP90AA1/ LYZ/ INHA/ GAL/ MMP12/ AMH/ NGFR/ MBL2/ TNFRSF25/ CRLF2/ OXT/ CRLF1/ KITLG/ NMB/ CCL28/ MIF/ CCL25/ CCL23/ CCL20/ FCN2/ TNFRSF18/ THBS1/ IL6/ LCN12/ ORM1/ CCL13/ CCL14/ DKK1/ TFRC/ GDF11/ ORM2/ DMBT1 |
| CC | GO:0009986 | cell surface | 0.113846 | 214/18493 | 8.83E-12 | 1.18E-08 | 2.59E-09 | WNT5A/ HSP90AB1/ MBL2/ MICB/ FGFR3/ ACVRL1/ MICA/ PDGFB/ PDGFA/ CSPG5/ CALR/ HLA-DMA/ TNFRSF4/ GHRHR/ TGFB1/ MIF/ IL17RB/ HSPA2/ ROBO1/ ITGAV/ TDGF1/ CCR10/ HSPA5/ THBS1/ GHR/ CIITA/ DEFB132/ HSP90AA1/ TNFSF4/ CD1D/ HLA-F/ TFRC/ ULBP2/ SORT1/ PDGFRB/ NGFR/ PLAU |
| CC | GO:0005887 | integral component of plasma membrane | 0.193846 | 439/18493 | 1.87E-11 | 2.49E-08 | 5.48E-09 | MCHR1/ PLXNA3/ ACVRL1/ MICA/ IL27RA/ PLXNA1/ PLXNA2/ JAG2/ TNFSF15/ CSPG5/ CXCR3/ JAG1/ VIPR1/ IL17RB/ MARCO/ EDNRA/ TNFRSF11A/ MC1R/ LTB4R/ ROBO1/ GALR3/ CCR10/ IL1RAP/ CALCRL/ ZYX/ NRG1/ CSF2RA/ GHR/ LIFR/ IL11RA/ CLEC4M/ TNFRSF9/ TNFRSF10C/ SEMA4F/ NGFR/ TNFRSF21/ PLXNC1/ FGFR4/ FGFR3/ TNFRSF25/ PTH1R/ LANCL1/ FPR1/ OXTR/ CD70/ TNFRSF4/ ITGAV/ TAP1/ TNFRSF18/ IL2RG/ PLXND1/ TNFSF4/ FLT1/ OPRL1/ CTLA4/ TRIM27/ STAB2/ AGER/ CD1D/ SEMA6B/ SEMA6C/ TFRC/ AVPR1A |
| CC | GO:0031093 | platelet alpha granule lumen | 0.036923 | 251/18493 | 2.65E-09 | 3.52E-06 | 7.75E-07 | VEGFB/ ORM1/ PPBP/ PDGFB/ ALB/ PDGFA/ HGF/ THBS1/ EGF/ TGFB1/ ORM2/ TGFB2 |
| CC | GO:0005886 | plasma membrane | 0.36 | 485/18493 | 2.57E-08 | 3.42E-05 | 7.53E-06 | HRAS/ PLXNA3/ MICB/ MICA/ PLXNA1/ PLXNA2/ TNFSF15/ JAG2/ FGF13/ JAG1/ VIPR1/ GHRHR/ TGFB1/ IL17RB/ MARCO/ AZGP1/ MC1R/ ROBO1/ LTB4R/ SEMA7A/ GALR3/ TDGF1/ CCR10/ CD3D/ LIFR/ COLEC12/ IL11RA/ HLA-G/ HLA-F/ CD320/ DLL4/ PDGFRB/ WNT5A/ IL1R2/ FGFR4/ PLXNC1/ IL22RA1/ FGFR3/ GNAI1/ PTH1R/ IFI30/ CD70/ SRC/ PTK2/ RAC3/ KLKB1/ ITGAV/ NDRG1/ EGF/ PLXND1/ IL1RL1/ IL1RN/ AGER/ S100A12/ PLCG1/ AVPR1A/ SYTL1/ IL3RA/ PLAU/ MCHR1/ ACVRL1/ IL27RA/ DUOX1/ CXCR3/ PDCD1/ EDNRA/ CASP3/ TNFRSF11A/ MAPT/ IL1RAP/ SHC1/ ANGPT1/ CALCRL/ PAK1/ ZYX/ NRG2/ LTB/ LTA/ CSF2RA/ GHR/ PRKCA/ HSP90AA1/ ESR1/ TNFRSF10C/ SEMA4F/ GIPR/ SORT1/ NGFR/ TNFRSF21/ CD244/ TNFRSF25/ CRLF2/ VIM/ FPR1/ OXTR/ KITLG/ TNFRSF4/ TNFRSF18/ IL2RG/ CD4/ HSPA5/ HTR3A/ SECTM1/ TNFSF4/ FLT1/ OPRL1/ NOX1/ CTLA4/ STAB2/ TNFSF9/ CD1D/ NRAS/ GPI/ DKK1/ TFRC/ FCGR2B/ ABCC4 |
| CC | GO:0009897 | external side of plasma membrane | 0.055385 | 144/18493 | 2.57E-07 | 3.42E-04 | 7.53E-05 | CD244/ IL6/ IL1RL1/ CTLA4/ CXCR3/ STAB2/ CALR/ CXCL12/ PDCD1/ TNFRSF9/ TNFRSF11A/ TFRC/ ITGAV/ SEMA7A/ CD4/ IL2RG/ THBS1/ NRG1 |
| MF | GO:0008083 | growth factor activity | 0.138462 | 488/18493 | 6.28E-39 | 9.20E-36 | 3.57E-36 | GDF2/ PDGFB/ PDGFA/ PGF/ JAG2/ KITLG/ FGF13/ OSGIN1/ JAG1/ CSPG5/ FGF12/ VGF/ IL34/ CXCL12/ MDK/ TGFB1/ IL11/ TGFB2/ NOV/ TYMP/ REG1A/ TDGF1/ PDGFD/ EGF/ NENF/ NRG1/ NRG2/ BMP4/ IL6/ NTF3/ HGF/ INHA/ GMFB/ VEGFB/ GPI/ AMH/ DKK1/ RABEP2/ CD320/ PPBP/ GRN/ GDF11/ BMP7/ BMP8B/ BMP5 |
| MF | GO:0005125 | cytokine activity | 0.107692 | 196/18493 | 6.65E-25 | 9.75E-22 | 1.89E-22 | WNT5A/ GDF2/ CRLF1/ TNFSF15/ KITLG/ CD70/ IL32/ IL34/ TGFB1/ IL11/ MIF/ TGFB2/ IL17D/ IL1B/ NRG1/ LTB/ LTA/ SPP1/ BMP4/ SECTM1/ IL6/ TNFSF4/ IL1RN/ INHA/ TNFSF9/ TSLP/ GPI/ GRN/ GDF11/ SCGB3A1/ CMTM4/ BMP7/ CMTM3/ BMP8B/ BMP5 |
| MF | GO:0005102 | receptor binding | 0.092308 | 33/18493 | 2.55E-11 | 3.74E-08 | 4.83E-09 | MBL2/ PLXNC1/ UTS2/ TNFSF15/ CD70/ HSPA1A/ HSPA1B/ CXCL12/ SRC/ MIF/ TGFB2/ PTK2/ HAMP/ TDGF1/ NOS2/ NRG1/ LTB/ NRG2/ APLN/ LTA/ TNFSF4/ NTF3/ INHA/ TNFSF9/ CCL15/ HLA-G/ HLA-F/ AMH/ CCL13/ PDGFRB |
| MF | GO:0008009 | chemokine activity | 0.04 | 202/18493 | 7.47E-11 | 1.09E-07 | 1.06E-08 | CCL8/ CCL28/ CCL15/ CXCL12/ CCL26/ CCL25/ CCL13/ CCL23/ CCL14/ CCL20/ CXCL14/ PPBP/ CKLF |
| MF | GO:0042056 | chemoattractant activity | 0.030769 | 499/18493 | 8.78E-10 | 1.29E-06 | 9.97E-08 | VEGFB/ BMP4/ NTF3/ SAA2/ PDGFB/ SAA1/ HGF/ CXCL12/ CCL15/ MIF |
| MF | GO:0003707 | steroid hormone receptor activity | 0.036923 | 293/18493 | 5.85E-09 | 8.57E-06 | 5.54E-07 | VDR/ PPARD/ THRA/ RARG/ RXRB/ NR6A1/ PPARG/ NR4A2/ ESR1/ NR4A1/ NR4A3/ NR2C2 |
| MF | GO:0005179 | hormone activity | 0.043077 | 234/18493 | 2.09E-08 | 3.07E-05 | 1.70E-06 | UTS2/ GNRH1/ STC2/ INS-IGF2/ NMB/ INHA/ VGF/ PTHLH/ AMH/ CCL25/ HAMP/ STC1/ ADM2/ APLN |
| MF | GO:0045499 | chemorepellent activity | 0.027692 | 153/18493 | 2.25E-08 | 3.30E-05 | 1.60E-06 | SEMA5B/ SEMA6B/ SEMA6C/ SEMA3G/ SEMA4F/ SEMA3F/ SEMA7A/ SEMA3B/ NRG1 |
| MF | GO:0005088 | Ras guanyl-nucleotide exchange factor activity | 0.046154 | 24/18493 | 3.17E-08 | 4.64E-05 | 2.00E-06 | FGFR4/ FGFR3/ PDGFB/ PDGFA/ KITLG/ PTK2/ PDGFRB/ SHC1/ IL2RG/ ANGPT1/ EGF/ NRG1/ NRG2/ CSF2RA/ IL3RA |
| MF | GO:0008201 | heparin binding | 0.052308 | 437/18493 | 5.56E-08 | 8.16E-05 | 3.16E-06 | BMP4/ FGFR4/ LTBP2/ PGF/ CCL8/ FGF12/ AGER/ MDK/ CCL15/ VEGFB/ NOV/ CCL23/ LPA/ SAA1/ THBS1/ BMP7/ CYR61 |
| MF | GO:0005160 | transforming growth factor beta receptor binding | 0.030769 | 158/18493 | 6.53E-08 | 9.57E-05 | 3.37E-06 | BMP4/ AMH/ GDF2/ GDF11/ INHA/ BMP7/ TGFB1/ BMP8B/ BMP5/ TGFB2 |
| MF | GO:0030215 | semaphorin receptor binding | 0.024615 | 26/18493 | 1.43E-07 | 2.09E-04 | 6.76E-06 | SEMA5B/ SEMA6B/ SEMA6C/ SEMA3G/ SEMA4F/ SEMA3F/ SEMA7A/ SEMA3B |
| MF | GO:0005031 | tumor necrosis factor-activated receptor activity | 0.024615 | 26/18493 | 1.98E-07 | 2.91E-04 | 8.67E-06 | TNFRSF21/ TNFRSF9/ TNFRSF10C/ TNFRSF11A/ TNFRSF25/ TNFRSF18/ NGFR/ TNFRSF4 |
| MF | GO:0001948 | glycoprotein binding | 0.033846 | 16/18493 | 3.30E-07 | 4.84E-04 | 1.34E-05 | HSP90AB1/ AZGP1/ HSP90AA1/ TFRC/ VIM/ CD4/ HSPA5/ CALR/ THBS1/ CANX/ TGFB1 |

GO gene ontology, BP biological process, CC cellular component, MF molecular function
