# Supplementary material for: Identification of a five-immune gene model as an independent prognostic factor in hepatocellular carcinoma
Source: BMC Cancer. 2021 Mar 16;21:278. doi: 10.1186/s12885-021-08012-2 (PMC7962305; doi:10.1186/s12885-021-08012-2)
Supplement: Supplementary file 5 — Additional file 5: Table S5. Significantly enriched KEGG terms [file 12885_2021_8012_MOESM5_ESM.docx]

**Table S5** Significantly enriched KEGG terms

| ID | Description | GeneRatio | BgRatio | pvalue | p.adjust | qvalue | geneID | Count |
| --- | --- | --- | --- | --- | --- | --- | --- | --- |
| hsa04060 | Cytokine-cytokine receptor interaction | 62/248 | 294/7946 | 1.79E-35 | 4.10E-33 | 1.94E-33 | 920/4049/5473/9547/6387/6357/6355/2826/3569/7040/3553/6364/56477/7292/6359/6358/10344/6368/6370/284340/2833/268/652/653/655/656/970/10220/2658/3589/53342/3557/9235/146433/3623/4050/7042/9966/8744/85480/94/64109/1438/2690/3590/55540/7850/3556/9173/58985/9466/3561/3563/3977/4804/8794/8792/8784/27242/8718/7293/3604 | 62 |
| hsa04612 | Antigen processing and presentation | 24/248 | 78/7946 | 4.27E-18 | 4.89E-16 | 2.31E-16 | 811/821/920/3108/3112/3134/3135/3303/3304/3305/3306/3308/3309/3310/3320/3326/4261/4800/5993/6890/6891/8625/10197/10437 | 24 |
| hsa04360 | Axon guidance | 33/248 | 181/7946 | 7.20E-17 | 5.49E-15 | 2.60E-15 | 6387/5595/2770/7474/6714/64221/5747/5881/4776/3265/4893/5296/8503/7869/6405/56920/10505/54437/10501/10500/8482/5361/5362/55558/10154/6091/655/5058/5335/5578/8440/10298/5163 | 33 |
| hsa04010 | MAPK signaling pathway | 38/248 | 295/7946 | 4.08E-14 | 2.34E-12 | 1.11E-12 | 3303/3304/3305/3306/3310/5971/7040/3553/4137/5595/1616/5605/3082/5159/5881/3265/4893/2353/8517/1950/4254/4908/5154/5155/80310/5228/7042/7423/284/2261/2264/2321/3556/4804/3164/5058/5578/836 | 38 |
| hsa01521 | EGFR tyrosine kinase inhibitor resistance | 20/248 | 79/7946 | 1.84E-13 | 8.44E-12 | 3.99E-12 | 3569/5595/6714/5605/3082/5159/3265/4893/5296/8503/1950/3084/9542/5154/5155/80310/2261/5335/6464/5578 | 20 |
| hsa04061 | Viral protein interaction with cytokine and cytokine receptor | 20/248 | 100/7946 | 2.07E-11 | 7.91E-10 | 3.75E-10 | 4049/5473/9547/6387/6357/6355/2826/3569/6364/56477/6359/6358/10344/6368/6370/2833/146433/58985/3561/8794 | 20 |
| hsa05162 | Measles | 23/248 | 138/7946 | 3.19E-11 | 1.04E-09 | 4.94E-10 | 3303/3304/3305/3306/3310/10332/57506/3569/3553/3661/9641/103/7297/7187/2353/8517/5296/8503/2213/3561/836/915/1019 | 23 |
| hsa04659 | Th17 cell differentiation | 20/248 | 107/7946 | 7.61E-11 | 2.18E-09 | 1.03E-09 | 920/3108/3112/3320/3326/3569/7040/3553/5595/7297/2353/8517/4794/53342/3556/9466/3561/6257/5335/915 | 20 |
| hsa05169 | Epstein-Barr virus infection | 27/248 | 201/7946 | 1.01E-10 | 2.58E-09 | 1.22E-09 | 811/3108/3112/3134/3135/5704/5708/5710/5971/6890/6891/10213/57506/3569/3661/9641/7297/7187/3065/7431/8517/4794/5296/8503/836/915/1019 | 27 |
| hsa04062 | Chemokine signaling pathway | 26/248 | 189/7946 | 1.35E-10 | 3.09E-09 | 1.46E-09 | 5473/9547/6387/6357/6355/2826/6364/5595/2770/6714/56477/6359/6358/10344/6368/6370/5747/5881/3265/4893/8517/5296/8503/2833/5058/6464 | 26 |
| hsa04015 | Rap1 signaling pathway | 27/248 | 210/7946 | 2.80E-10 | 5.83E-09 | 2.76E-09 | 7057/5595/2770/6714/5605/3082/5159/5881/3265/4893/5296/8503/2357/1950/4254/5154/5155/80310/5228/7423/284/2261/2264/2321/4804/5335/5578 | 27 |
| hsa04014 | Ras signaling pathway | 28/248 | 232/7946 | 5.69E-10 | 1.09E-08 | 5.14E-09 | 5595/5605/3082/5159/5881/3265/4893/8517/5296/8503/1950/4254/4908/5154/5155/80310/5228/7423/284/2261/2264/2321/4804/5058/5335/6464/5578/10298 | 28 |
| hsa04012 | ErbB signaling pathway | 17/248 | 85/7946 | 7.02E-10 | 1.24E-08 | 5.86E-09 | 5595/6714/5605/5747/3265/4893/5296/8503/1950/3084/9542/5058/5335/6464/5578/8440/10298 | 17 |
| hsa04151 | PI3K-Akt signaling pathway | 35/248 | 354/7946 | 8.94E-10 | 1.46E-08 | 6.92E-09 | 3320/3326/7057/3569/3685/5595/5605/3082/5159/5747/3265/4893/8517/5296/8503/1950/4254/4908/5154/5155/80310/5228/6696/7423/284/2261/2264/2321/2690/3561/3563/4804/3164/5578/1019 | 35 |
| hsa05170 | Human immunodeficiency virus 1 infection | 26/248 | 212/7946 | 1.73E-09 | 2.64E-08 | 1.25E-08 | 811/920/3134/3135/6890/6891/3661/5595/2770/164668/5605/5747/5881/4776/3265/4893/2353/8517/5296/8503/5058/5335/5578/836/915/10298 | 26 |
| hsa04660 | T cell receptor signaling pathway | 18/248 | 104/7946 | 2.58E-09 | 3.66E-08 | 1.73E-08 | 920/5595/5605/5133/3265/4893/2353/8517/4794/5296/8503/5058/5335/915/8440/10298/1493/1019 | 18 |
| hsa05167 | Kaposi sarcoma-associated herpesvirus infection | 24/248 | 186/7946 | 2.72E-09 | 3.66E-08 | 1.73E-08 | 3134/3135/100507436/4277/3569/3661/9641/5595/7297/7187/6714/5743/5605/4776/3265/4893/2353/8517/5296/8503/5155/5335/836/1019 | 24 |
| hsa04630 | JAK-STAT signaling pathway | 22/248 | 162/7946 | 4.91E-09 | 5.92E-08 | 2.80E-08 | 3569/9021/7297/5159/3265/5296/8503/1950/3589/53342/5154/5155/85480/64109/1438/2690/3590/58985/9466/3561/3563/3977 | 22 |
| hsa05161 | Hepatitis B | 22/248 | 162/7946 | 4.91E-09 | 5.92E-08 | 2.80E-08 | 57506/3569/7040/4318/3661/9641/5595/7297/7187/6714/5605/332/4776/3265/4893/2353/8517/5296/8503/7042/5578/836 | 22 |
| hsa05215 | Prostate cancer | 17/248 | 97/7946 | 5.94E-09 | 6.80E-08 | 3.22E-08 | 3320/3326/4318/5328/5595/5605/5159/3265/4893/8517/5296/8503/1950/5154/5155/80310/7850 | 17 |
| hsa05163 | Human cytomegalovirus infection | 26/248 | 225/7946 | 6.24E-09 | 6.81E-08 | 3.22E-08 | 811/3134/3135/6890/6891/6387/3569/3553/3661/3685/5595/2770/6714/5743/5605/5747/5881/4776/3265/4893/8517/5296/8503/5578/836/1019 | 26 |
| hsa04933 | AGE-RAGE signaling pathway in diabetic complications | 17/248 | 100/7946 | 9.62E-09 | 1.00E-07 | 4.74E-08 | 3569/7040/3553/50507/27035/5595/177/3265/4893/5296/8503/7042/7423/5335/5578/836/1019 | 17 |
| hsa04510 | Focal adhesion | 24/248 | 199/7946 | 1.06E-08 | 1.06E-07 | 5.01E-08 | 7057/7791/3685/5595/6714/3082/5159/5747/5881/3265/5296/8503/1950/5154/5155/80310/5228/6696/7423/2321/5058/6464/5578/10298 | 24 |
| hsa05166 | Human T-cell leukemia virus 1 infection | 25/248 | 219/7946 | 1.60E-08 | 1.52E-07 | 7.22E-08 | 811/821/920/3108/3112/3134/3135/4049/5971/3569/7040/5595/5605/4776/3265/4893/2353/8517/5296/8503/7042/7850/3561/915/1019 | 25 |
| hsa04657 | IL-17 signaling pathway | 16/248 | 94/7946 | 2.61E-08 | 2.39E-07 | 1.13E-07 | 3320/3326/3934/3569/4318/3553/6364/9641/5595/7187/5743/2353/8517/53342/55540/836 | 16 |
| hsa05219 | Bladder cancer | 11/248 | 41/7946 | 3.03E-08 | 2.67E-07 | 1.26E-07 | 7057/4318/5595/6714/5605/3265/4893/1890/1950/2261/1019 | 11 |
| hsa04915 | Estrogen signaling pathway | 19/248 | 138/7946 | 4.58E-08 | 3.88E-07 | 1.84E-07 | 3303/3304/3305/3306/3310/3320/3326/4318/5595/2770/6714/5605/3265/4893/2353/5296/8503/2099/6464 | 19 |
| hsa01522 | Endocrine resistance | 16/248 | 98/7946 | 4.82E-08 | 3.94E-07 | 1.86E-07 | 4318/5595/6714/54567/5605/5747/3265/4893/2353/5296/8503/182/3714/2099/6464/1019 | 16 |
| hsa05214 | Glioma | 14/248 | 75/7946 | 5.92E-08 | 4.64E-07 | 2.20E-07 | 5595/5605/5159/3265/4893/5296/8503/1950/5154/5155/5335/6464/5578/1019 | 14 |
| hsa05164 | Influenza A | 21/248 | 170/7946 | 6.08E-08 | 4.64E-07 | 2.20E-07 | 3108/3112/4261/57506/3569/3553/3661/5371/9021/9641/5595/103/7297/7187/5605/8517/5296/8503/5578/836/1019 | 21 |
| hsa04625 | C-type lectin receptor signaling pathway | 16/248 | 104/7946 | 1.14E-07 | 8.42E-07 | 3.99E-07 | 5971/10332/3569/3553/9641/5595/6714/5743/4776/3265/4893/8517/5296/8503/53342/5058 | 16 |
| hsa05323 | Rheumatoid arthritis | 15/248 | 93/7946 | 1.51E-07 | 1.08E-06 | 5.12E-07 | 3108/3112/6387/3569/7040/3553/6364/2353/3589/4050/7042/284/2321/8792/1493 | 15 |
| hsa04370 | VEGF signaling pathway | 12/248 | 59/7946 | 1.99E-07 | 1.38E-06 | 6.55E-07 | 5595/6714/5743/5605/5747/5881/3265/4893/5296/8503/5335/5578 | 12 |
| hsa05218 | Melanoma | 13/248 | 72/7946 | 2.68E-07 | 1.80E-06 | 8.54E-07 | 5595/5605/3082/5159/3265/4893/5296/8503/1950/5154/5155/80310/1019 | 13 |
| hsa05231 | Choline metabolism in cancer | 15/248 | 98/7946 | 3.08E-07 | 2.01E-06 | 9.53E-07 | 5595/5605/5159/5881/3265/4893/2353/5296/8503/1950/5154/5155/80310/5335/5578 | 15 |
| hsa05145 | Toxoplasmosis | 16/248 | 112/7946 | 3.27E-07 | 2.08E-06 | 9.85E-07 | 3108/3112/3303/3304/3305/3306/3310/4261/7040/5595/2770/7297/4843/8517/7042/836 | 16 |
| hsa05235 | PD-L1 expression and PD-1 checkpoint pathway in cancer | 14/248 | 89/7946 | 5.46E-07 | 3.38E-06 | 1.60E-06 | 920/5595/5605/5133/3265/4893/2353/8517/4794/5296/8503/1950/5335/915 | 14 |
| hsa04650 | Natural killer cell mediated cytotoxicity | 17/248 | 131/7946 | 5.69E-07 | 3.43E-06 | 1.62E-06 | 3135/100507436/4277/80328/5595/5605/5881/3265/4893/5296/8503/5058/5335/6464/51744/5578/836 | 17 |
| hsa04658 | Th1 and Th2 cell differentiation | 14/248 | 92/7946 | 8.28E-07 | 4.86E-06 | 2.30E-06 | 920/3108/3112/5595/7297/54567/2353/8517/4794/182/3714/3561/5335/915 | 14 |
| hsa05211 | Renal cell carcinoma | 12/248 | 69/7946 | 1.19E-06 | 6.81E-06 | 3.22E-06 | 7040/5595/5605/3082/3265/4893/5296/8503/5155/7042/5058/10298 | 12 |
| hsa05418 | Fluid shear stress and atherosclerosis | 17/248 | 139/7946 | 1.33E-06 | 7.37E-06 | 3.49E-06 | 3320/3326/4318/3553/27035/3685/6714/5747/5881/2353/8517/5296/8503/652/5154/5155/7850 | 17 |
| hsa05205 | Proteoglycans in cancer | 21/248 | 204/7946 | 1.35E-06 | 7.37E-06 | 3.49E-06 | 7057/7040/4318/5328/3685/5595/7474/6714/5605/3082/5747/3265/4893/5296/8503/7042/2099/5058/5335/5578/836 | 21 |
| hsa04917 | Prolactin signaling pathway | 12/248 | 70/7946 | 1.40E-06 | 7.43E-06 | 3.52E-06 | 9021/5595/6714/5605/3265/4893/2353/5296/8503/2099/8792/6464 | 12 |
| hsa04668 | TNF signaling pathway | 15/248 | 112/7946 | 1.79E-06 | 9.13E-06 | 4.32E-06 | 4049/3569/4318/3553/9021/6364/5595/7187/5743/2353/8517/5296/8503/182/836 | 15 |
| hsa05340 | Primary immunodeficiency | 9/248 | 38/7946 | 1.79E-06 | 9.13E-06 | 4.32E-06 | 920/4261/5993/6890/6891/8625/8517/3561/915 | 9 |
| hsa04640 | Hematopoietic cell lineage | 14/248 | 99/7946 | 2.06E-06 | 1.02E-05 | 4.85E-06 | 912/920/3108/3112/3569/3553/7037/3589/4254/1438/3590/7850/3563/915 | 14 |
| hsa05210 | Colorectal cancer | 13/248 | 86/7946 | 2.23E-06 | 1.09E-05 | 5.14E-06 | 7040/5595/5605/332/5881/3265/4893/2353/5296/8503/1950/7042/836 | 13 |
| hsa05220 | Chronic myeloid leukemia | 12/248 | 76/7946 | 3.45E-06 | 1.64E-05 | 7.78E-06 | 7040/5595/5605/3065/3265/4893/8517/5296/8503/7042/6464/1019 | 12 |
| hsa04919 | Thyroid hormone signaling pathway | 15/248 | 119/7946 | 3.88E-06 | 1.78E-05 | 8.41E-06 | 3685/5595/6714/5605/3065/3265/4893/5296/8503/652/2099/6257/7067/5335/5578 | 15 |
| hsa04935 | Growth hormone synthesis, secretion and action | 15/248 | 119/7946 | 3.88E-06 | 1.78E-05 | 8.41E-06 | 9021/5595/2770/5605/5747/3265/4893/2353/5296/8503/2690/2692/5335/6464/5578 | 15 |
| hsa05223 | Non-small cell lung cancer | 11/248 | 66/7946 | 5.16E-06 | 2.32E-05 | 1.10E-05 | 5595/5605/3265/4893/5296/8503/1950/6257/5335/5578/1019 | 11 |
| hsa05160 | Hepatitis C | 17/248 | 155/7946 | 6.05E-06 | 2.67E-05 | 1.26E-05 | 10197/57506/3661/9021/9641/5595/7297/7187/5605/3265/4893/8517/5296/8503/1950/836/1019 | 17 |
| hsa04380 | Osteoclast differentiation | 15/248 | 128/7946 | 9.64E-06 | 4.16E-05 | 1.97E-05 | 5971/7040/3553/27035/9021/5595/7297/5468/2353/8517/5296/8503/2213/7042/8792 | 15 |
| hsa04810 | Regulation of actin cytoskeleton | 20/248 | 213/7946 | 9.96E-06 | 4.22E-05 | 2.00E-05 | 6387/3685/5595/6714/5605/5159/5747/5881/3265/4893/5296/8503/1950/5154/5155/80310/2261/2264/5058/10298 | 20 |
| hsa04926 | Relaxin signaling pathway | 15/248 | 129/7946 | 1.06E-05 | 4.42E-05 | 2.09E-05 | 7040/4318/5595/2770/4843/6714/5605/3265/4893/2353/5296/8503/7423/6464/5578 | 15 |
| hsa04072 | Phospholipase D signaling pathway | 16/248 | 148/7946 | 1.37E-05 | 5.62E-05 | 2.66E-05 | 5595/5605/5159/3265/4893/5296/8503/1950/4254/5154/5155/80310/552/5335/6464/5578 | 16 |
| hsa05142 | Chagas disease (American trypanosomiasis) | 13/248 | 102/7946 | 1.54E-05 | 6.06E-05 | 2.87E-05 | 811/3569/7040/3553/5595/2770/4843/2353/8517/5296/8503/7042/915 | 13 |
| hsa05146 | Amoebiasis | 13/248 | 102/7946 | 1.54E-05 | 6.06E-05 | 2.87E-05 | 912/3569/7040/3553/4843/384/5747/5296/8503/7042/7850/5578/836 | 13 |
| hsa04540 | Gap junction | 12/248 | 88/7946 | 1.64E-05 | 6.35E-05 | 3.01E-05 | 5595/2770/6714/5605/5159/3265/4893/1950/5154/5155/80310/5578 | 12 |
| hsa04213 | Longevity regulating pathway - multiple species | 10/248 | 62/7946 | 1.88E-05 | 7.12E-05 | 3.37E-05 | 3303/3304/3305/3306/3310/3065/3265/4893/5296/8503 | 10 |
| hsa04620 | Toll-like receptor signaling pathway | 13/248 | 104/7946 | 1.90E-05 | 7.12E-05 | 3.37E-05 | 3569/3553/3661/9641/3663/5595/7187/5605/2353/8517/5296/8503/6696 | 13 |
| hsa04145 | Phagosome | 16/248 | 152/7946 | 1.93E-05 | 7.12E-05 | 3.37E-05 | 811/821/3108/3112/3134/3135/6890/6891/7057/10332/81035/4153/7037/3685/8685/2213 | 16 |
| hsa04066 | HIF-1 signaling pathway | 13/248 | 109/7946 | 3.16E-05 | 0.000115 | 5.44E-05 | 3569/7037/5595/4843/5605/5296/8503/1950/284/2321/5335/5578/5163 | 13 |
| hsa05165 | Human papillomavirus infection | 25/248 | 330/7946 | 3.34E-05 | 0.00012 | 5.66E-05 | 3134/3135/7057/3661/9641/3685/5595/7474/7297/7187/5743/5605/3065/5159/5747/3265/4893/8517/5296/8503/1950/182/6696/836/1019 | 25 |
| hsa04662 | B cell receptor signaling pathway | 11/248 | 82/7946 | 4.32E-05 | 0.000151 | 7.16E-05 | 5595/5605/5881/3265/4893/2353/8517/4794/5296/8503/2213 | 11 |
| hsa05152 | Tuberculosis | 17/248 | 180/7946 | 4.36E-05 | 0.000151 | 7.16E-05 | 3108/3112/4261/4800/5993/8625/10332/3569/7040/3553/5595/4843/6714/7421/2213/7042/836 | 17 |
| hsa05224 | Breast cancer | 15/248 | 147/7946 | 5.07E-05 | 0.000173 | 8.21E-05 | 5595/7474/54567/5605/3265/4893/2353/5296/8503/1950/182/3714/2099/6464/1019 | 15 |
| hsa04064 | NF-kappa B signaling pathway | 12/248 | 102/7946 | 7.31E-05 | 0.000246 | 0.000117 | 4049/5971/6387/6357/5328/3553/7187/5743/8517/4050/8792/5335 | 12 |
| hsa05135 | Yersinia infection | 13/248 | 120/7946 | 8.73E-05 | 0.00029 | 0.000137 | 3569/3553/3661/5595/6714/5605/5747/5881/2353/8517/5296/8503/5335 | 13 |
| hsa04672 | Intestinal immune network for IgA production | 8/248 | 49/7946 | 0.00012 | 0.000392 | 0.000185 | 3108/3112/6387/2826/3569/7040/56477/6370 | 8 |
| hsa05222 | Small cell lung cancer | 11/248 | 92/7946 | 0.000126 | 0.000407 | 0.000193 | 3685/4843/7187/5743/5747/8517/5296/8503/6257/836/1019 | 11 |
| hsa04929 | GnRH secretion | 9/248 | 64/7946 | 0.000149 | 0.000475 | 0.000225 | 5595/5605/3265/4893/5296/8503/2796/6696/5578 | 9 |
| hsa05221 | Acute myeloid leukemia | 9/248 | 67/7946 | 0.000214 | 0.00067 | 0.000317 | 5371/5595/5605/3265/4893/8517/5296/8503/5467 | 9 |
| hsa04664 | Fc epsilon RI signaling pathway | 9/248 | 68/7946 | 0.00024 | 0.000742 | 0.000351 | 5595/5605/5881/3265/4893/5296/8503/5335/5578 | 9 |
| hsa05206 | MicroRNAs in cancer | 22/248 | 310/7946 | 0.000259 | 0.00079 | 0.000374 | 7057/4318/5328/5595/5743/5605/3065/5159/7431/3265/4893/5296/8503/5154/5155/7042/2261/5335/6464/5578/836/10298 | 22 |
| hsa05230 | Central carbon metabolism in cancer | 9/248 | 69/7946 | 0.000268 | 0.000808 | 0.000383 | 5595/5605/5159/3265/4893/5296/8503/2261/5163 | 9 |
| hsa04722 | Neurotrophin signaling pathway | 12/248 | 119/7946 | 0.000321 | 0.000955 | 0.000452 | 5595/5605/3265/4893/4794/5296/8503/4908/4804/6272/5335/6464 | 12 |
| hsa05134 | Legionellosis | 8/248 | 57/7946 | 0.000354 | 0.001038 | 0.000491 | 3303/3304/3305/3306/3310/3569/3553/836 | 8 |
| hsa04080 | Neuroactive ligand-receptor interaction | 23/248 | 340/7946 | 0.000375 | 0.001086 | 0.000514 | 1241/1909/2357/8862/51083/2796/4828/5020/7349/10911/552/10203/8484/2690/2692/2696/4157/2847/4987/5021/5745/7067/7433 | 23 |
| hsa05133 | Pertussis | 9/248 | 76/7946 | 0.000558 | 0.001577 | 0.000747 | 3569/3553/3661/10392/5595/2770/4843/2353/836 | 9 |
| hsa05212 | Pancreatic cancer | 9/248 | 76/7946 | 0.000558 | 0.001577 | 0.000747 | 7040/5595/5881/8517/5296/8503/1950/7042/1019 | 9 |
| hsa05140 | Leishmaniasis | 9/248 | 77/7946 | 0.000615 | 0.001717 | 0.000813 | 3108/3112/7040/3553/5595/4843/5743/2353/7042 | 9 |
| hsa05020 | Prion diseases | 6/248 | 35/7946 | 0.00066 | 0.001801 | 0.000852 | 3303/3309/3569/3553/5595/5605 | 6 |
| hsa04350 | TGF-beta signaling pathway | 10/248 | 94/7946 | 0.00066 | 0.001801 | 0.000852 | 7057/57817/7040/5595/268/652/653/655/656/7042 | 10 |
| hsa05225 | Hepatocellular carcinoma | 14/248 | 168/7946 | 0.000746 | 0.001989 | 0.000942 | 7040/5595/7474/5605/3082/3265/4893/5296/8503/7042/5335/6464/5578/1019 | 14 |
| hsa05226 | Gastric cancer | 13/248 | 149/7946 | 0.000747 | 0.001989 | 0.000942 | 7040/5595/7474/5605/3082/3265/4893/5296/8503/1950/7042/6257/6464 | 13 |
| hsa05216 | Thyroid cancer | 6/248 | 37/7946 | 0.000897 | 0.002361 | 0.001118 | 5595/5605/5468/3265/4893/6257 | 6 |
| hsa04210 | Apoptosis | 12/248 | 136/7946 | 0.001075 | 0.002798 | 0.001325 | 5595/1616/5605/332/3265/4893/2353/8517/5296/8503/3563/836 | 12 |
| hsa04218 | Cellular senescence | 13/248 | 160/7946 | 0.001446 | 0.003721 | 0.001762 | 3134/3135/3569/7040/5595/5605/4776/3265/4893/5296/8503/7042/1019 | 13 |
| hsa05332 | Graft-versus-host disease | 6/248 | 41/7946 | 0.001563 | 0.003977 | 0.001883 | 3108/3112/3134/3135/3569/3553 | 6 |
| hsa04940 | Type I diabetes mellitus | 6/248 | 43/7946 | 0.002012 | 0.005063 | 0.002397 | 3108/3112/3134/3135/4049/3553 | 6 |
| hsa05213 | Endometrial cancer | 7/248 | 58/7946 | 0.002049 | 0.005099 | 0.002414 | 5595/5605/3265/4893/5296/8503/1950 | 7 |
| hsa04921 | Oxytocin signaling pathway | 12/248 | 153/7946 | 0.002933 | 0.007223 | 0.00342 | 5595/2770/6714/5743/5605/4776/3265/4893/2353/5020/5021/5578 | 12 |
| hsa04623 | Cytosolic DNA-sensing pathway | 7/248 | 63/7946 | 0.003304 | 0.008049 | 0.003811 | 57506/3569/3553/3661/9641/103/8517 | 7 |
| hsa05321 | Inflammatory bowel disease (IBD) | 7/248 | 65/7946 | 0.003944 | 0.009508 | 0.004502 | 3108/3112/3569/7040/3553/7042/3561 | 7 |
| hsa05203 | Viral carcinogenesis | 14/248 | 201/7946 | 0.004053 | 0.009669 | 0.004578 | 3134/3135/3661/5595/7187/6714/3065/3265/4893/8517/5296/8503/836/1019 | 14 |
| hsa04916 | Melanogenesis | 9/248 | 101/7946 | 0.004165 | 0.009834 | 0.004656 | 5595/2770/7474/5605/3265/4893/4254/4157/5578 | 9 |
| hsa04550 | Signaling pathways regulating pluripotency of stem cells | 11/248 | 140/7946 | 0.004274 | 0.009896 | 0.004685 | 5595/7474/5605/3265/4893/5296/8503/652/2261/2264/3977 | 11 |
| hsa04621 | NOD-like receptor signaling pathway | 13/248 | 181/7946 | 0.004278 | 0.009896 | 0.004685 | 3320/3326/57506/3569/3553/3661/9641/10392/5595/7297/7187/2634/8517 | 13 |
| hsa05144 | Malaria | 6/248 | 50/7946 | 0.004374 | 0.010017 | 0.004743 | 7057/3569/7040/3553/3082/7042 | 6 |
| hsa05202 | Transcriptional misregulation in cancer | 13/248 | 186/7946 | 0.005379 | 0.012197 | 0.005775 | 3569/4318/5328/5371/3065/5468/5747/5154/2321/7850/4804/8013/6257 | 13 |
| hsa04928 | Parathyroid hormone synthesis, secretion and action | 9/248 | 106/7946 | 0.005727 | 0.012858 | 0.006088 | 5595/2770/7421/2353/5744/4929/5745/6257/5578 | 9 |
| hsa04024 | cAMP signaling pathway | 14/248 | 216/7946 | 0.00759 | 0.016875 | 0.00799 | 5595/2770/5605/10257/5881/2353/5296/8503/1909/268/5020/2696/5021/5058 | 14 |
| hsa04068 | FoxO signaling pathway | 10/248 | 131/7946 | 0.007723 | 0.017006 | 0.008051 | 3569/7040/5595/5605/3265/4893/5296/8503/1950/7042 | 10 |
| hsa04730 | Long-term depression | 6/248 | 60/7946 | 0.010649 | 0.023006 | 0.010892 | 5595/2770/5605/3265/4893/5578 | 6 |
| hsa05416 | Viral myocarditis | 6/248 | 60/7946 | 0.010649 | 0.023006 | 0.010892 | 3108/3112/3134/3135/5881/836 | 6 |
| hsa04071 | Sphingolipid signaling pathway | 9/248 | 119/7946 | 0.011912 | 0.025494 | 0.01207 | 5595/2770/5605/5881/3265/4893/5296/8503/5578 | 9 |
| hsa03050 | Proteasome | 5/248 | 45/7946 | 0.012557 | 0.026625 | 0.012605 | 5704/5708/5710/10197/10213 | 5 |
| hsa01523 | Antifolate resistance | 4/248 | 31/7946 | 0.015026 | 0.031568 | 0.014946 | 3569/3553/10257/8517 | 4 |
| hsa05120 | Epithelial cell signaling in Helicobacter pylori infection | 6/248 | 70/7946 | 0.021588 | 0.044942 | 0.021278 | 10392/6714/8517/5058/5335/836 | 6 |
| hsa04670 | Leukocyte transendothelial migration | 8/248 | 112/7946 | 0.023566 | 0.048184 | 0.022813 | 6387/4318/2770/5747/5296/8503/5335/5578 | 8 |
| hsa04725 | Cholinergic synapse | 8/248 | 112/7946 | 0.023566 | 0.048184 | 0.022813 | 5595/2770/3265/4893/2353/5296/8503/5578 | 8 |
| hsa04144 | Endocytosis | 14/248 | 249/7946 | 0.02381 | 0.048253 | 0.022846 | 3134/3135/3303/3304/3305/3306/3310/5371/7037/6714/3265/2261/2264/3561 | 14 |
| hsa05320 | Autoimmune thyroid disease | 5/248 | 53/7946 | 0.024148 | 0.048509 | 0.022967 | 3108/3112/3134/3135/1493 | 5 |

KEGG Kyoto Encyclopedia of Genes and Genomes
