# Supplementary material for: Identification of a five-immune gene model as an independent prognostic factor in hepatocellular carcinoma
Source: BMC Cancer. 2021 Mar 16;21:278. doi: 10.1186/s12885-021-08012-2 (PMC7962305; doi:10.1186/s12885-021-08012-2)
Supplement: Supplementary file 6 — Additional file 6: Table S6. DE TFs in HCC [file 12885_2021_8012_MOESM6_ESM.docx]

**Table S6** DE TFs in HCC

| ID | logFC | pValue | FDR |
| --- | --- | --- | --- |
| ADNP | 1.111343 | 1.74E-19 | 7.91E-19 |
| ARID3A | 3.256725 | 2.49E-18 | 9.77E-18 |
| ATF3 | -1.1881 | 1.07E-11 | 2.27E-11 |
| BACH2 | -1.01294 | 4.58E-09 | 8.12E-09 |
| BATF | 1.543613 | 0.000362 | 0.000466 |
| BRCA1 | 1.502983 | 5.86E-12 | 1.27E-11 |
| BRF2 | 1.126861 | 2.05E-14 | 5.41E-14 |
| CBX2 | 3.45998 | 2.15E-24 | 2.70E-23 |
| CBX3 | 1.221811 | 2.24E-25 | 4.02E-24 |
| CBX5 | 1.22137 | 1.27E-15 | 3.72E-15 |
| CBX8 | 1.871865 | 3.25E-27 | 1.60E-25 |
| CDK2 | 1.065195 | 8.63E-12 | 1.84E-11 |
| CDK7 | 1.195768 | 4.38E-25 | 6.93E-24 |
| CEBPA | 1.298142 | 2.30E-10 | 4.46E-10 |
| CENPA | 4.889403 | 1.73E-27 | 1.07E-25 |
| CHD7 | 1.145013 | 2.61E-12 | 5.81E-12 |
| CIITA | 1.070709 | 1.61E-05 | 2.27E-05 |
| DNMT1 | 1.906233 | 7.50E-21 | 4.21E-20 |
| DNMT3A | 1.996224 | 7.94E-24 | 8.47E-23 |
| E2F1 | 4.35381 | 1.32E-27 | 8.85E-26 |
| E2F3 | 1.418712 | 2.54E-13 | 6.10E-13 |
| E2F4 | 1.206904 | 1.27E-22 | 1.00E-21 |
| E2F7 | 4.285471 | 2.60E-24 | 3.17E-23 |
| EBF1 | 2.826119 | 4.55E-25 | 7.14E-24 |
| EED | 1.255517 | 1.10E-24 | 1.52E-23 |
| EGR1 | -1.94885 | 4.40E-19 | 1.89E-18 |
| EGR2 | -1.81224 | 1.71E-15 | 4.96E-15 |
| EHF | 2.304048 | 0.0108 | 0.012552 |
| EHMT2 | 2.01215 | 4.23E-28 | 4.56E-26 |
| ELK4 | 1.015572 | 5.00E-14 | 1.28E-13 |
| EMX1 | 4.562804 | 4.54E-10 | 8.62E-10 |
| EP400 | 1.153527 | 1.31E-18 | 5.30E-18 |
| ESR1 | -1.94173 | 1.04E-22 | 8.36E-22 |
| EZH1 | 1.313501 | 7.55E-24 | 8.13E-23 |
| EZH2 | 3.104109 | 4.59E-28 | 4.65E-26 |
| FOS | -2.41925 | 6.68E-21 | 3.78E-20 |
| FOXK1 | 1.929439 | 7.21E-25 | 1.07E-23 |
| FOXM1 | 4.385308 | 4.85E-27 | 2.09E-25 |
| FOXO1 | -1.07571 | 4.28E-15 | 1.19E-14 |
| GATA2 | 1.116715 | 3.29E-05 | 4.54E-05 |
| GATAD1 | 1.13109 | 3.23E-21 | 1.94E-20 |
| GREB1 | 2.130265 | 8.75E-05 | 0.000117 |
| H2AFX | 2.172701 | 8.35E-24 | 8.86E-23 |
| HCFC1 | 1.374623 | 4.46E-24 | 5.07E-23 |
| HDAC1 | 1.026001 | 6.77E-19 | 2.84E-18 |
| HEY1 | 2.041161 | 9.46E-22 | 6.23E-21 |
| HOXB13 | 6.991439 | 3.68E-07 | 5.77E-07 |
| HOXB7 | 2.419392 | 3.55E-09 | 6.35E-09 |
| HOXC9 | 6.726338 | 3.78E-11 | 7.74E-11 |
| HSF1 | 1.456387 | 3.17E-25 | 5.33E-24 |
| HSF2 | 1.292878 | 2.69E-18 | 1.05E-17 |
| IRF3 | 1.360867 | 9.34E-24 | 9.80E-23 |
| IRF5 | 1.245307 | 4.35E-17 | 1.49E-16 |
| JARID2 | 1.102662 | 6.82E-16 | 2.06E-15 |
| JMJD6 | 1.104572 | 4.13E-17 | 1.42E-16 |
| KDM1A | 1.000926 | 5.94E-22 | 4.08E-21 |
| KDM5B | 1.473827 | 1.38E-16 | 4.48E-16 |
| KDM5C | 1.00199 | 2.49E-15 | 7.10E-15 |
| LEF1 | 3.567111 | 3.47E-17 | 1.21E-16 |
| LIN9 | 2.301167 | 1.07E-23 | 1.10E-22 |
| LMNA | 1.462122 | 2.92E-23 | 2.68E-22 |
| LMNB1 | 2.014673 | 3.52E-15 | 9.90E-15 |
| MAZ | 1.479326 | 7.22E-20 | 3.45E-19 |
| MED12 | 1.313723 | 2.94E-18 | 1.14E-17 |
| MYBL2 | 5.305148 | 3.92E-27 | 1.83E-25 |
| NCAPG | 4.54893 | 7.79E-28 | 6.35E-26 |
| NCOR2 | 1.243819 | 1.06E-19 | 4.94E-19 |
| NFYA | 1.330314 | 5.49E-19 | 2.32E-18 |
| NR2C2 | 1.219943 | 2.68E-12 | 5.96E-12 |
| NR4A1 | -1.51633 | 3.37E-14 | 8.71E-14 |
| NRF1 | 1.019899 | 3.77E-22 | 2.68E-21 |
| OGT | 1.372058 | 5.94E-21 | 3.40E-20 |
| PML | 1.061959 | 9.29E-18 | 3.44E-17 |
| POLR3A | 1.04707 | 7.34E-23 | 6.04E-22 |
| POLR3G | 1.184477 | 3.97E-13 | 9.40E-13 |
| POU2F1 | 1.09359 | 6.13E-19 | 2.58E-18 |
| POU5F1 | 2.704226 | 3.69E-18 | 1.42E-17 |
| PPARD | 1.042313 | 1.51E-11 | 3.18E-11 |
| PPARG | 1.208227 | 6.56E-11 | 1.32E-10 |
| PRKDC | 1.514472 | 2.87E-20 | 1.46E-19 |
| RARG | 1.064021 | 1.02E-07 | 1.67E-07 |
| RBP2 | 4.984714 | 4.18E-05 | 5.73E-05 |
| RFX5 | 1.812846 | 2.23E-23 | 2.11E-22 |
| RING1 | 1.074018 | 6.73E-23 | 5.60E-22 |
| SAP30 | 1.292846 | 1.01E-17 | 3.73E-17 |
| SCML2 | 2.073651 | 1.51E-18 | 6.06E-18 |
| SETDB1 | 1.469838 | 6.34E-25 | 9.57E-24 |
| SIRT6 | 1.273792 | 1.63E-23 | 1.61E-22 |
| SIX5 | 1.164564 | 3.79E-16 | 1.17E-15 |
| SMAD2 | 1.05422 | 5.27E-22 | 3.65E-21 |
| SMAD3 | 1.093508 | 8.62E-13 | 1.99E-12 |
| SMARCA4 | 1.640492 | 4.92E-27 | 2.12E-25 |
| SMARCB1 | 1.142845 | 8.60E-22 | 5.73E-21 |
| SMARCC1 | 1.173822 | 2.88E-19 | 1.27E-18 |
| SMC3 | 1.04315 | 1.84E-12 | 4.13E-12 |
| SNAI2 | 1.73855 | 1.00E-06 | 1.53E-06 |
| SNAPC2 | 1.168367 | 1.52E-23 | 1.51E-22 |
| SOX2 | 4.409603 | 0.000866 | 0.001091 |
| SOX4 | 2.015579 | 3.10E-07 | 4.89E-07 |
| SOX9 | 1.970541 | 2.70E-06 | 4.01E-06 |
| SPDEF | 3.58993 | 0.038361 | 0.043014 |
| SRC | 1.862442 | 2.16E-10 | 4.19E-10 |
| SREBF2 | 1.245609 | 4.68E-17 | 1.60E-16 |
| SSRP1 | 1.014534 | 3.35E-21 | 2.00E-20 |
| SUMO2 | 1.029261 | 8.60E-23 | 7.00E-22 |
| SUPT5H | 1.074232 | 6.42E-27 | 2.47E-25 |
| TCF21 | -1.07805 | 1.62E-16 | 5.24E-16 |
| TCF7 | 1.538831 | 1.20E-06 | 1.83E-06 |
| TEAD4 | 1.464149 | 2.02E-07 | 3.23E-07 |
| TFAP2A | 2.967708 | 5.48E-08 | 9.07E-08 |
| TP73 | 4.102942 | 2.96E-23 | 2.71E-22 |
| TRIM28 | 1.405146 | 3.70E-25 | 6.00E-24 |
| TTF2 | 1.740866 | 4.62E-18 | 1.77E-17 |
| USF1 | 1.454414 | 1.32E-28 | 3.21E-26 |
| VDR | 1.55964 | 1.87E-05 | 2.62E-05 |
| WDR5 | 1.210397 | 8.35E-24 | 8.86E-23 |
| ZBTB17 | 1.051057 | 7.65E-24 | 8.20E-23 |

DE differentially expressed, TFs transcription factors, HCC hepatocellular carcinoma, FC Fold Change, FDR false discovery rate
