# Supplementary figures and images for: Identification of a five-immune gene model as an independent prognostic factor in hepatocellular carcinoma
Source: BMC Cancer. 2021 Mar 16;21:278. doi: 10.1186/s12885-021-08012-2 (PMC7962305; doi:10.1186/s12885-021-08012-2)

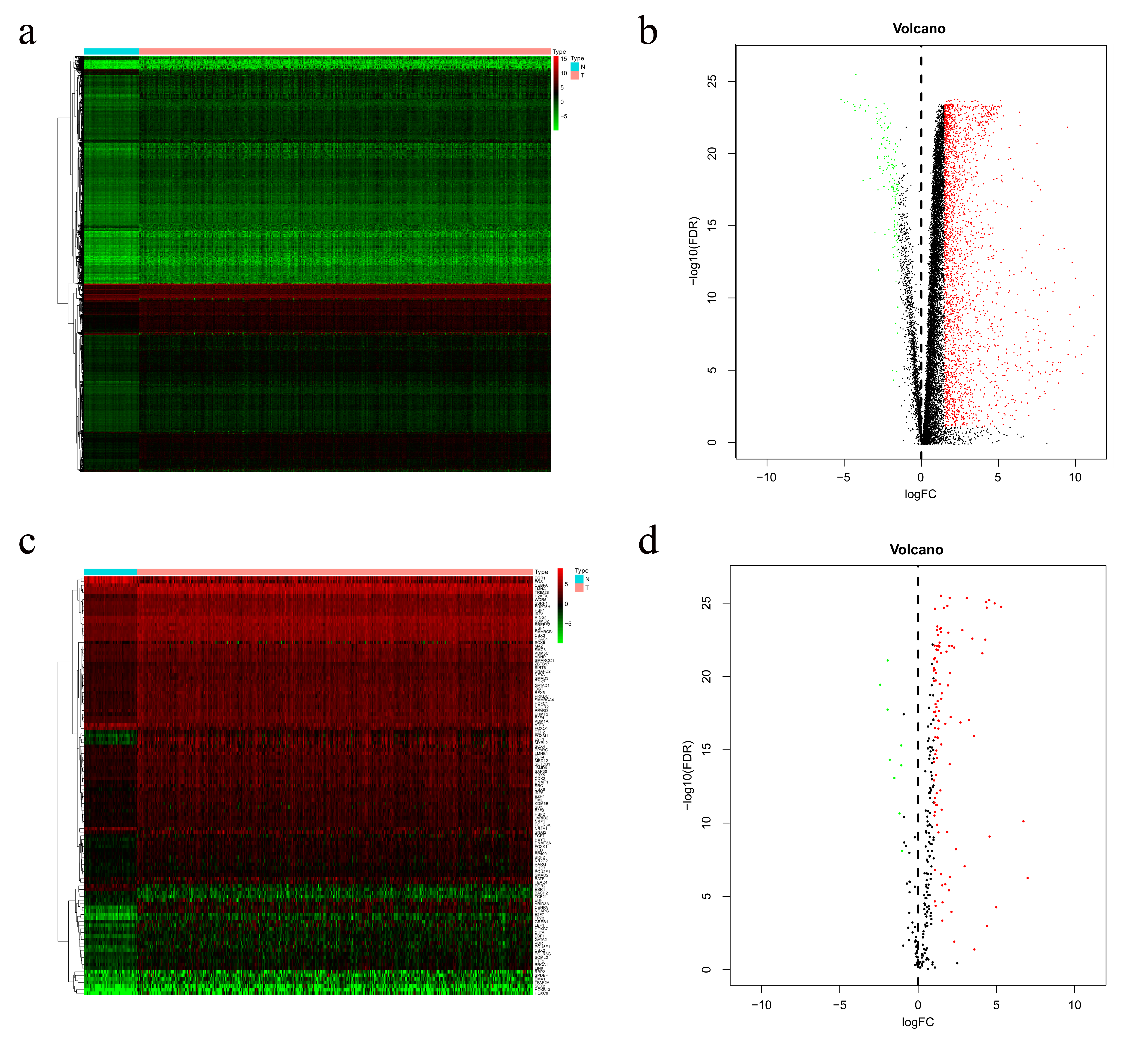

Supplement: Supplementary file 7 — Additional file 7: Supplementary Figure 1. (a) The volcano map of the DE genes. (b) The heatmap of the DE genes. (c) The volcano map of the DE TFs. (d) The heatmap of the DE TFs. [file 12885_2021_8012_MOESM7_ESM.tif]

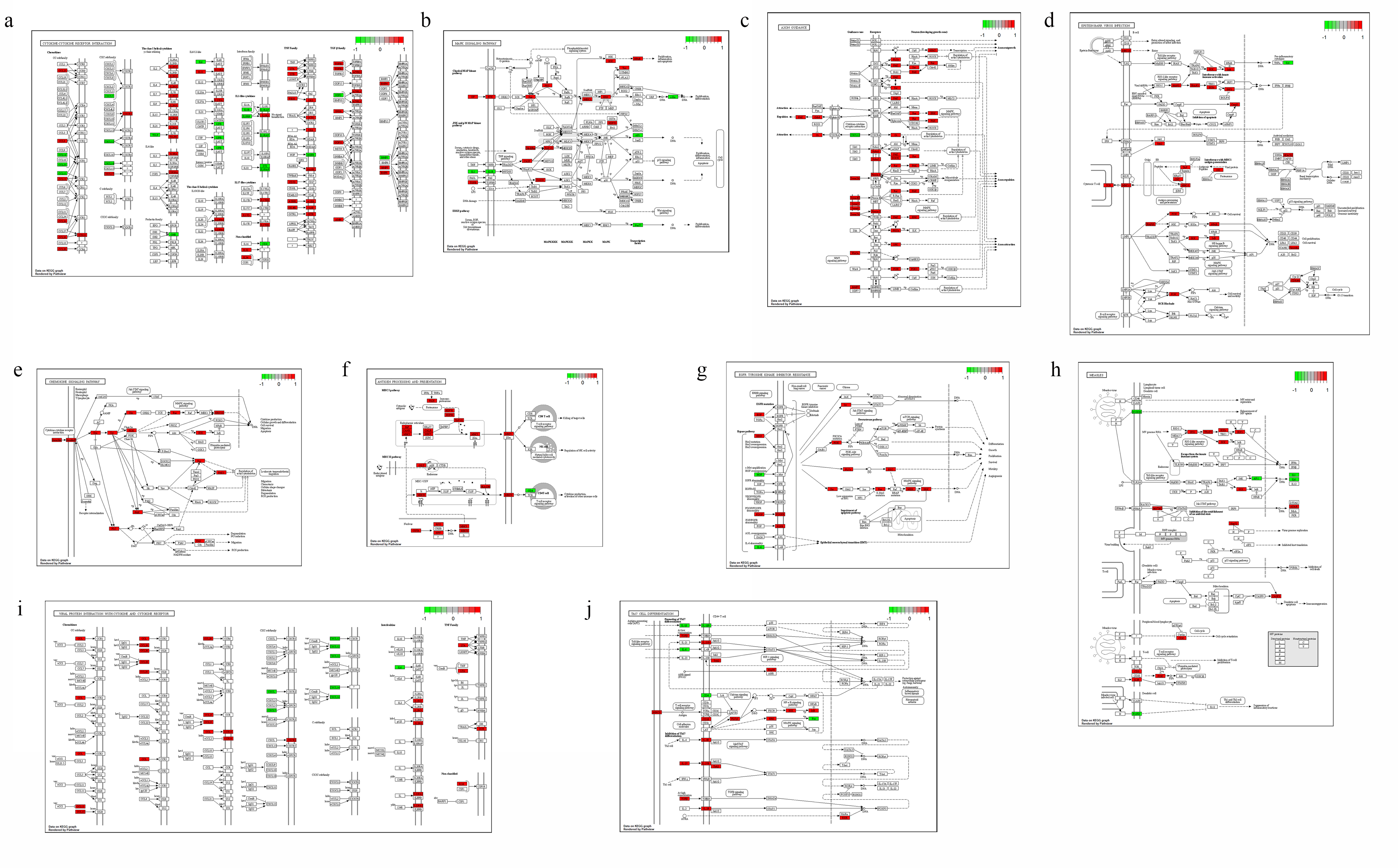

Supplement: Supplementary file 8 — Additional file 8: Supplementary Figure 2. The dysregulated genes were shown in the top ten pathways with the Pathview package. [file 12885_2021_8012_MOESM8_ESM.tif]

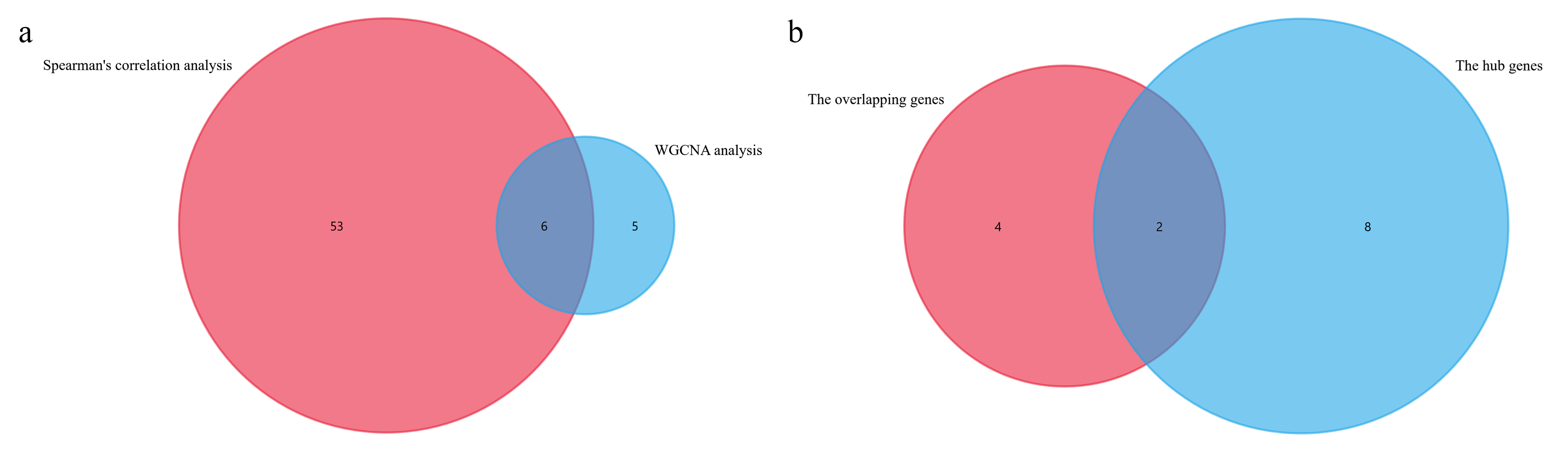

Supplement: Supplementary file 9 — Additional file 9: Supplementary Figure 3. Identification of the common genes based on WGCNA analysis, Spearman correlation analysis, and protein-protein interaction (PPI) network. [file 12885_2021_8012_MOESM9_ESM.tif]
